# Characterization of microRNAs of *Beta macrocarpa* and their responses to *Beet necrotic yellow vein virus infection*

**DOI:** 10.1371/journal.pone.0186500

**Published:** 2017-10-16

**Authors:** Jun-Ying Liu, Hui-Yan Fan, Ying Wang, Yong-Liang Zhang, Da-Wei Li, Jia-Lin Yu, Cheng-Gui Han

**Affiliations:** 1 State Key Laboratory for Agro-Biotechnology and Ministry of Agriculture Key Laboratory for Plant Pathology, China Agricultural University, Beijing, P. R. China; 2 College of Pharmacy, Zhejiang Chinese Medicine University, Hangzhou, Zhejiang, China; Institut de Biologie Moleculaire et Cellulaire, FRANCE

## Abstract

Plant microRNAs (miRNAs) are a class of non-coding RNAs that play important roles in plant development, defense, and symptom development. Here, 547 known miRNAs representing 129 miRNA families, and 282 potential novel miRNAs were identified in *Beta macrocarpa* using small RNA deep sequencing. A phylogenetic analysis was performed, and 8 *Beta* lineage-specific miRNAs were identified. Through a differential expression analysis, miRNAs associated with *Beet necrotic yellow vein virus* (BNYVV) infection were identified and confirmed using a microarray analysis and stem-loop RT-qPCR. In total, 103 known miRNAs representing 38 miRNA families, and 45 potential novel miRNAs were differentially regulated, with at least a two-fold change, in BNYVV-infected plants compared with that of the mock-inoculated control. Targets of these differentially expressed miRNAs were also predicted by degradome sequencing. These differentially expressed miRNAs were involved in hormone biosynthesis and signal transduction pathways, and enhanced axillary bud development and plant defenses. This work is the first to describe miRNAs of the plant genus *Beta* and may offer a reference for miRNA research in other species in the genus. It provides valuable information on the pathogenicity mechanisms of BNYVV.

## Introduction

Plant microRNAs (miRNAs) are a class of 20–24 nt endogenous small non-coding RNAs. Their coding genes possess their own transcriptional units [1] and are mostly located in intergenic regions, introns or inverse repeats of coding sequences [2]. These miRNA genes (MIR) are transcribed into primary miRNAs with a secondary stem-loop structure in the partial sequence by RNA polymerase II [3, 4]. Primary miRNAs are processed into mature miRNAs through two or more cleavage events, transported to the cytoplasm, methylated and despiralized by helicase. The mature miRNA guide strand enters into the RNA-induced silencing complex and regulates gene expression by target cleavage, translational inhibition and DNA methylation. The miRNA passenger strand (miRNA*) is degraded by an unclear mechanism [5, 6], and some miRNA* may also function in plant defense and development [7, 8].

There are conserved miRNAs and lineage-specific miRNAs in all plant species [9]. miRNA sequence abundance and conservation are correlated [10], and miRNAs are inherited through vertical descent [9]. Some conserved miRNA families, for example miR156, miR160, miR166 and miR172, which are generally highly expressed and ubiquitous across all terrestrial species [10, 11], often have conserved targets that play specific functions in plant development, and are probably under stringent selection pressures [12]. Lineage-specific miRNAs are diverse and common in individual species, even in *Arabidopsis thaliana* and *Arabidopsis lyrata*, and are subjected to less selection during evolution within the genus [12, 13].

Genome-wide analyses of many plants infected by viruses have proven that plant viruses often disturb host miRNA pathways, and the aberrant expression of some miRNAs could affect plant defenses or morphological development. For example, *Rice stripe virus* (RSV) blocks the defense response by significantly down-regulating the miR1870-5p- and miR1423-5p-mediated disease resistance pathways in infected rice plants [14]. Similarly, the induction of miR319 in rice by *Rice ragged stunt virus* suppresses jasmonic acid (JA)-mediated defenses to facilitate viral infection and symptom development [15]. miR159 plays an important role in eliciting the abnormal phenotype in *Arabidopsis* infected by *Cucumber mosaic virus* (strain FNY) [16]; *Turnip mosaic virus*-infected *Arabidopsis* misregulates miR167, and the down-regulated miR167 and up-regulated target Auxin Response Factor 8 are major causes for the phenotypic aberrations [17]. Thus, disturbance of the miRNA pathway is closely related to the pathogenesis of plant viruses.

*Beet necrotic yellow vein virus* (BNYVV) [18], a member of the *Benyvirus* genus, severely impacts sugar beet production by causing beet rhizomania disease. BNYVV is a multiparticle virus with four or five positive-sense, single-stranded RNAs (RNA1–5) which are individually packaged into rod-shaped virion [19]. The larger RNA1 and RNA2 genomes encode the housekeeping genes of the virus, while the smaller RNAs3, 4, and 5 are involved in pathogenicity and vector transmission during BNYVV systemic infection of beet [20–23]. The *RNA3-p25* gene encodes virulence and avirulence factors [21, 24], and amino acid changes in p25 can generate resistance-breaking variants [24]. *RNA3-p25* can target the sugar beet 26S proteasome involved in the induction of hypersensitive resistance responses through interactions with an F-box protein [25]. Additionally, other P25-interacting sugar beet proteins represent putative viral targets or components of plant resistance, but their specific functions are not clear [26]. *RNA3-p25* can also induce hormonal changes and a root branching phenotype in transgenic *Arabidopsis thaliana* [27]. In addition, a transcriptome analysis of *Beta macrocarpa* was performed to identify differentially expressed transcripts in response to BNYVV infection. Gene ontology (GO) analysis showed that these differentially expressed genes were mainly enriched in biotic stimuli and primary metabolic processes [28]. However, to date, miRNAs expression profiles in response to BNYVV infection remains unexplored. In addition, sugar beets, one of the main sugar-yielding crops, are of significant economic value, and miRNA-based biotechnology plays an important role in plant improvement [5]. Although whole-genome information of *Beta vulgaris* was available in 2014, miRNAs of the plant species in the genus *Beta* remains largely unknown.

In this study, small RNA deep sequencing was performed to investigate miRNAs from leaves of BNYVV-infected (VL) and virus-free (L) plants. The characteristics of miRNAs were systematically studied through a phylogenetic analysis, and the differential expressions of miRNAs in response to BNYVV infection were investigated and further validated by microarray analysis and stem-loop RT-qPCR. miRNAs’ targets were also predicted by degradome sequencing. To our knowledge, this is the first report that defines *B*. *macrocarpa* miRNAs, which would expand our knowledge of miRNAs in the genus *Beta*, and aid in the discovery of miRNAs related to the pathogenic mechanisms underlying beet–BNYVV interactions.

## Results and discussion

### High-throughput small RNA sequencing of BNYVV-infected and virus-free leaves of *B*. *macrocarpa*

**Fig 1:**
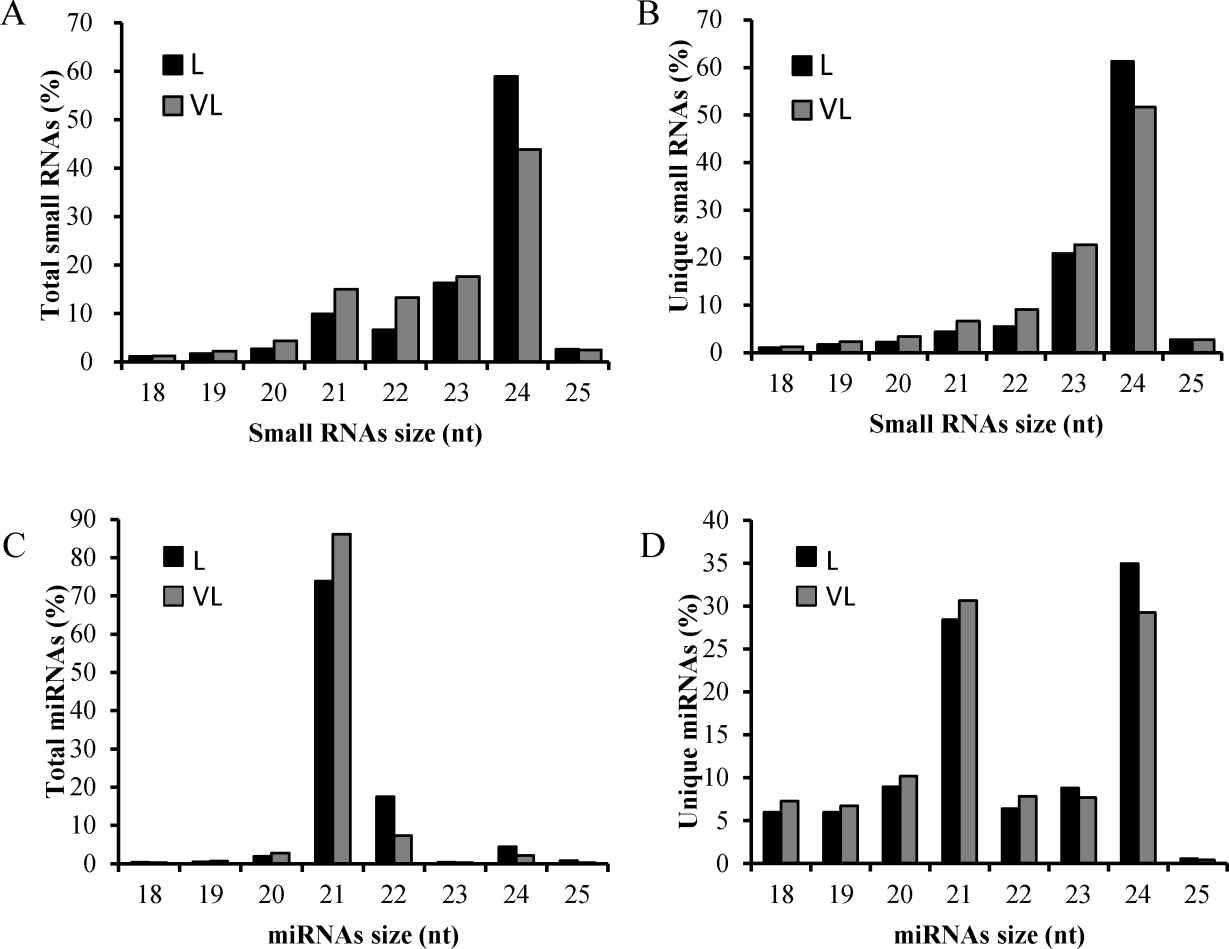
Size distribution of small RNAs. The size distribution of total (A) or unique (B) beet-derived small RNAs and total (C) or unique (D) miRNAs in two libraries. L, small RNA library from leaves; VL, from BNYVV-infected leaves; nt, nucleotide.

To investigate the *B*. *macrocarpa* miRNAs involved in BNYVV infection, two small RNA libraries were constructed from leaves of virus-free (L) and BNYVV-infected (VL) plants. The raw sequence data have been deposited in NCBI database with the accession number GSE101436. Small RNA sequencing generated 17,249,248 and 13,628,866 raw reads from the L and VL libraries, respectively (Table 1). After removal of low-quality and corrupted adaptor sequences (reads < 18 nt), 14,556,099 and 11,458,601 clean reads were obtained from the L and VL libraries, respectively (Table 1). The size distribution of sequences showed that the majority of small RNAs in our libraries belonged to the 24-nt size class (Fig 1A and 1B). In the small RNA libraries from BNYVV-infected plants, 15.36% of the high-quality reads and 5.09% of the unique reads were well matched to the BNYVV genome, whereas negligible numbers (less than 50 reads) were obtained from the healthy control (Table 1). Our sequence data are in agreement with those of many plant species, such as *Nicotiana benthamiana* [29], *Medicago truncatula* [30], *Punica granatum* [31] and *Arabidopsis* [32] in which the 24-nt size class is the most abundant of the small RNAs. Thus, these data confirmed the high quality of the small RNA libraries, indicating that they were suitable for further comparative analyses.

**Table 1:** Summary of small RNA sequencing from the BNYVV-infected and virus-free library of *B*. *macrocarpa*.

category^a^	L		VL	
	Total	Unique	Total	Unique
**Raw reads**	17249248	8641445	13628866	7375672
**Clean reads**	14906380	7767049	11783522	6494568
**Known miRNAs**^b^	255523	440	266443	449
**mapped to the virus genome**^c^			1809583	330729

^a^ L, VL represent small RNAs library from leaves or BNYVV-infected leaves, respectively.

^b^ Small RNAs matching known mature miRNAs (miRbase v21.0) with no more than two mismatches.

^c^ Small RNAs matching BNYVV genome with a maximum of one mismatch.

**Fig 2:**
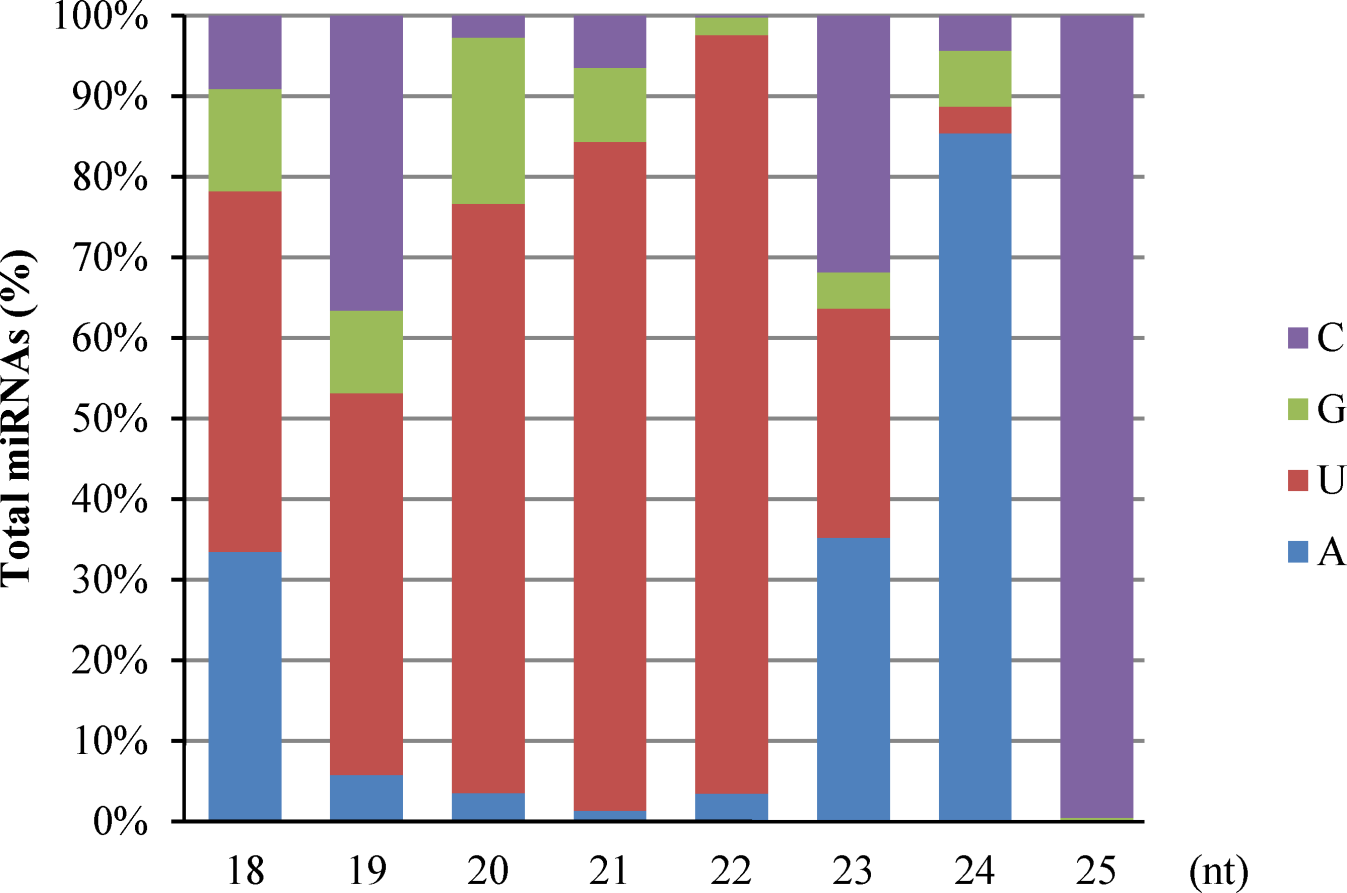
The first nucleotide bias of the known and potential miRNAs.

We then identified known miRNAs by mapping these unique small RNAs to miRBase v21.0 (http://microrna.sanger.ac.uk), allowing up to two mismatches during the alignment. Others were mapped to transcripts of *B*. *macrocarpa* [27] or reference genomes of *B*. *vulgaris* in NCBI to identify their precursors, and then each miRNA whose precursor met the criterion [minimal free energy index (MFEI) > 0.9] was defined as a potential novel miRNA. Finally, 547 miRNAs, belonging to 129 known miRNA families, and 282 potential novel miRNAs, with three or more reads in the L or VL library were identified (S1 Table). Detection of the corresponding miRNA* is an important factor in verifying the existence of miRNA. miRNA-3p and -5p were detected simultaneously in 16 of 129 known miRNA families (S2 Table) and 43 of 282 potential novel miRNAs (S3 Table). However, due to the incomplete genomic information for *B*. *macrocarpa*, pre-miRNAs of 97 of the 129 known miRNA families were not identified, including conserved miRNA 168, and miR390 (S1 Table). Thus, all of the known miRNAs identified in this study, with or without proper pre-miRNAs, were considered in the following analysis. The statistical data on the length distribution of mature miRNAs revealed that 21-nt and 22-nt miRNAs were the most abundant miRNAs, and the species of 21-nt and 24-nt miRNAs were the most abundant unique miRNAs (Fig 1C and 1D). We also determined the frequency of the first base of mature miRNAs and found that the 20-nt, 21-nt, and 22-nt miRNAs preferentially started with ‘U’ (73.14%, 83.01% and 94.09%, respectively), while 24-nt miRNAs preferred ‘A’ at the first base (Fig 2).

### Phylogenetic analysis of the microRNAome of *B*. *macrocarpa*

**Fig 3:**
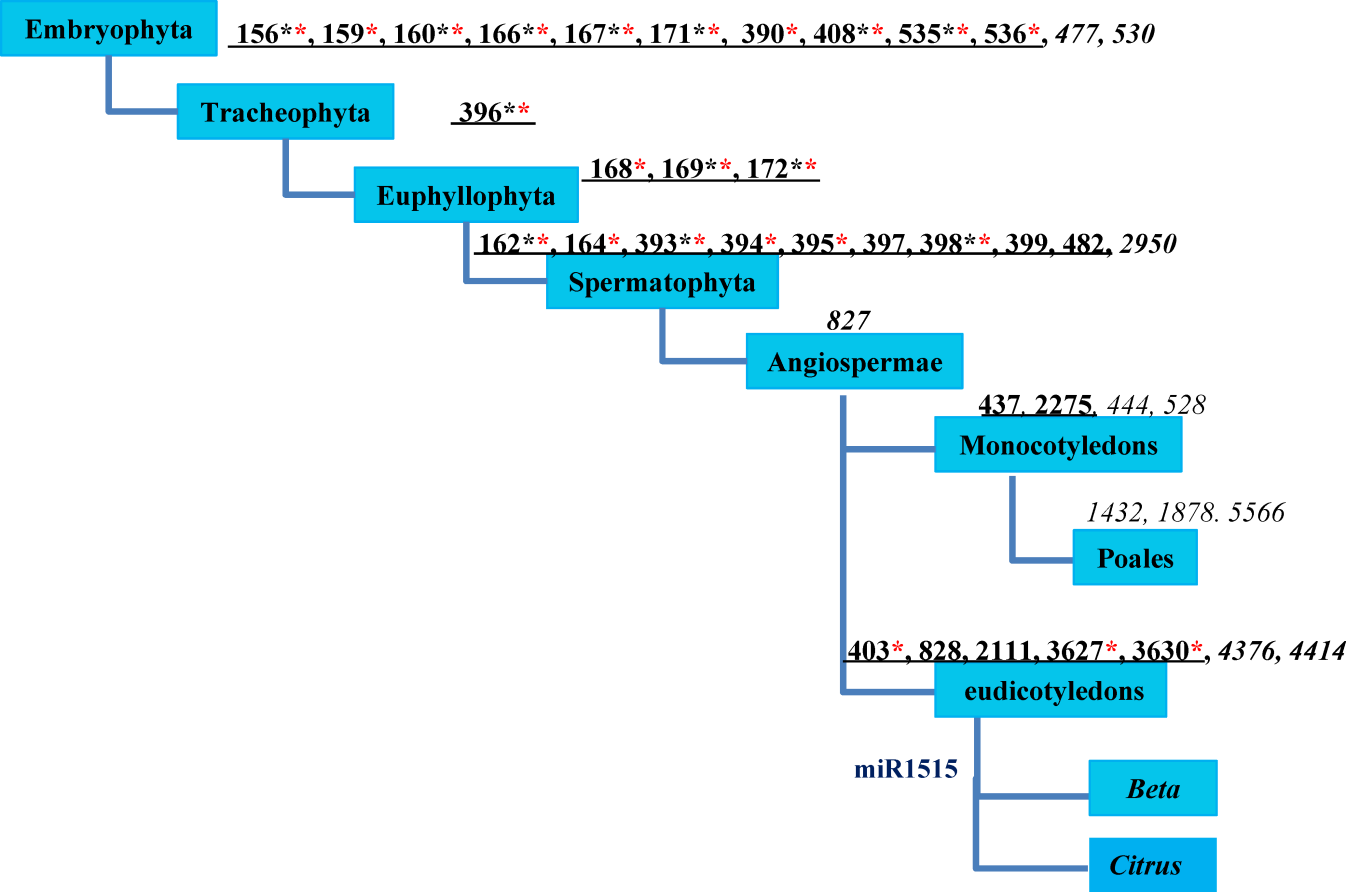
The distribution of miRNAs of *B*. *macrocarpa* within the phylogenetic perspective of plant miRNAs. The phylogenetic perspective was proposed by Taylor et al. [9]. The graphic mode has been used according to the model of Yin et al. [33]. Black underlined numbers indicate miRNA families in beet; Black italic numbers indicate missing miRNA families. Black * represents the existence of the miRNA was confirmed by detection of its corresponding miRNA*; and red * represents the expression of miRNA was confirmed by a microarray analysis. miR1515 is highlighted in blue, suggesting that it may have been generated before the separation of *Citrus* and *Beta*.

To gain a deeper understanding of the *B*. *macrocarpa* microRNAome, and to offer a reference for the microRNAome research of other species of the genus *Beta*, or even of Caryophyllales, the *B*. *macrocarpa* miRNAs’ distribution within the phylogenetic perspective of plant miRNAs proposed by Taylor et al. [9] was investigated. Through sequence analyses, we identified 28 out of 34 conserved miRNA families in Eudicotyledons, including miR482, miR535, miR536, miR828, miR3627 and miR3630 (Fig 3). They were confirmed by the detection of their corresponding miRNA*s, and a microarray analysis. In total, 13 out of 28 miRNA families were further confirmed by these analyses (S2 Table, S5 Table in bold font). Additionally, 10 of 15 other miRNA families were tested again by microarray analysis (S5 Table in bold font). miR2111, miR397, miR399, miR428 and miR828 were not tested again by microarray analysis because of their relatively low levels of expression, and the precursors of the first three miRNAs met the criteria (MFEI > 1) (S1 Table). No precursors were found for the latter two miRNAs. In healthy *B*. *macrocarpa*, the miR159 family with 22 members presented the highest expression abundance with 71,771 reads, followed by miR393. miR535 was expressed at a relatively high level, with more than 10,000 reads compared with its low expression in other species.

There are six missing miRNAs including miR477, miR530, miR2950, miR827, miR4376, and miR4414 (Fig 3). However, there were also no these missing miRNAs in *Silene latifolia* belonging to the Caryophyllaceae family of *Caryophyllales*, [10]. We cannot conclude that this is a common characteristic of plant miRNAs in Caryophyllales because of the limited number of samples.

We also found 93 reads of miR1515 (with only one mismatch), a *Citrus sinensis* species-specific miRNA [9], in the *B*. *macrocarpa* microRNAome by small RNA sequencing and a microarray analysis (S5 Table). Thus, miR1515 may have originated before the separation of *Citrus* and *Beta* (Fig 3).

**Fig 4:**
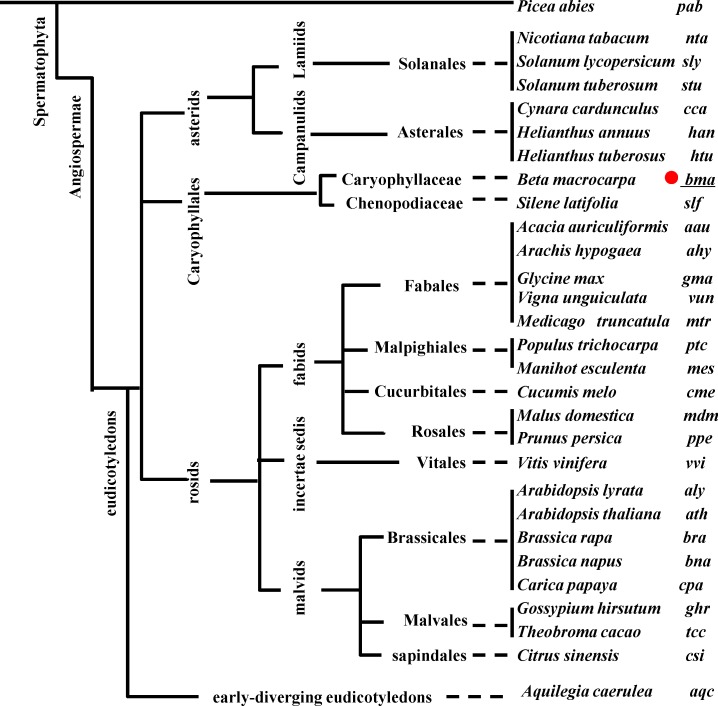
The phylogenetic distribution of the 29 plant species analyzed in this study. The phylogenetic tree was built using the common tree tool in NCBI.(https://www.ncbi.nlm.nih.gov/Taxonomy/CommonTree/). Species are presented in order using the three-letter codes to the right of the full species name.

**Fig 5:**
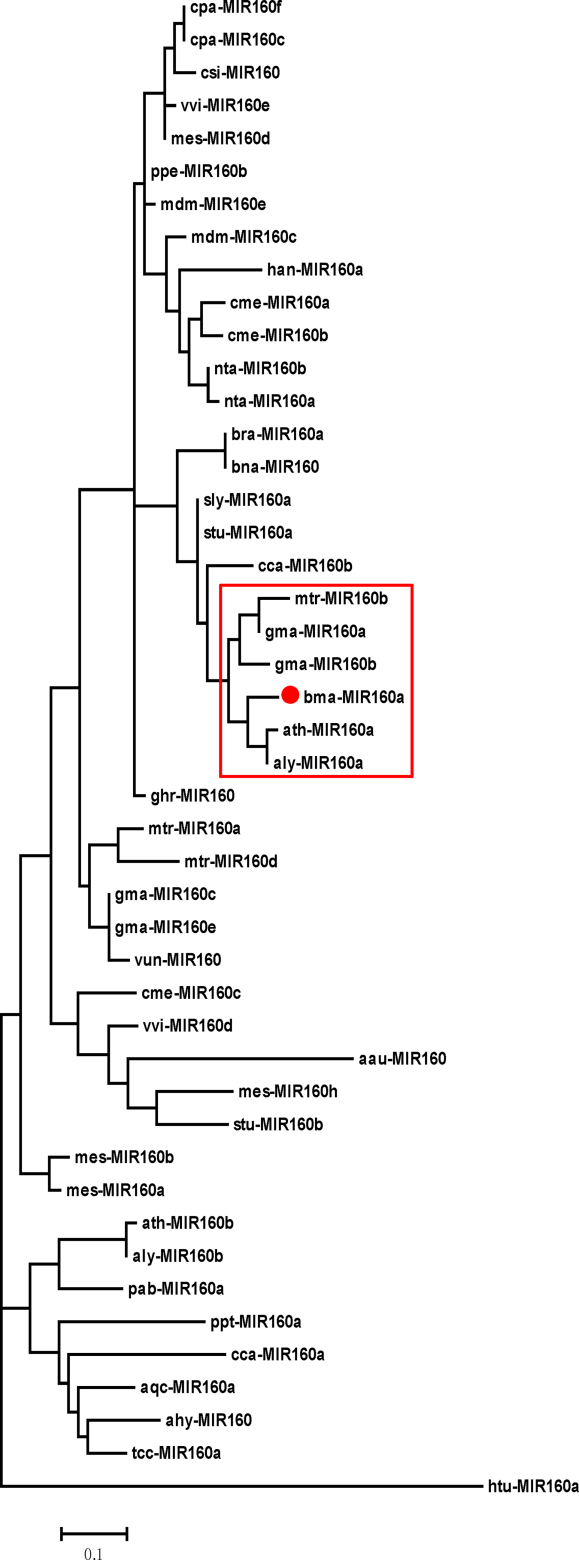
Characterization of MIR160a of *B*. *macrocarpa*. The phylogenetic tree was constructed using precursors of miRNA160 from diverse plant species obtained from miRBase v21.0 by MEGA 5.0.

To gain more evolutionary insights into the *B*. *macrocarpa* microRNAome, a phylogenetic analysis of precursors of miR160 (MIR160) with 29 species (Fig 4) used in Yin et al. [33] and a cross-species conservative analysis of known miRNAs were performed. To construct the MIR160 phylogenetic tree, ath-miR160a-5p (reads = 871) was chosen as the study object and renamed as bma-miR160a. bma-miR160a was the most abundant of three miR160s found in our small RNA sequencing, and, based on the secondary structure of its precursor, it had the canonical stem-loop structure of a processed mature miRNA (MFEI = 1.10) (S1 File). In addition, bma-miR160a* (reads = 74), which matched its precursor, was also identified. The phylogenetic tree showed that bma-MIR160a formed a subgroup with *Glycine max*, *M*. *truncatula*, *A*. *lyrata*, and *A*. *thaliana* (Fig 5), and the relationship between bma-MIR160a and gma-, mtr-, aly- or ath-MIR160 was close.

**Fig 6:**
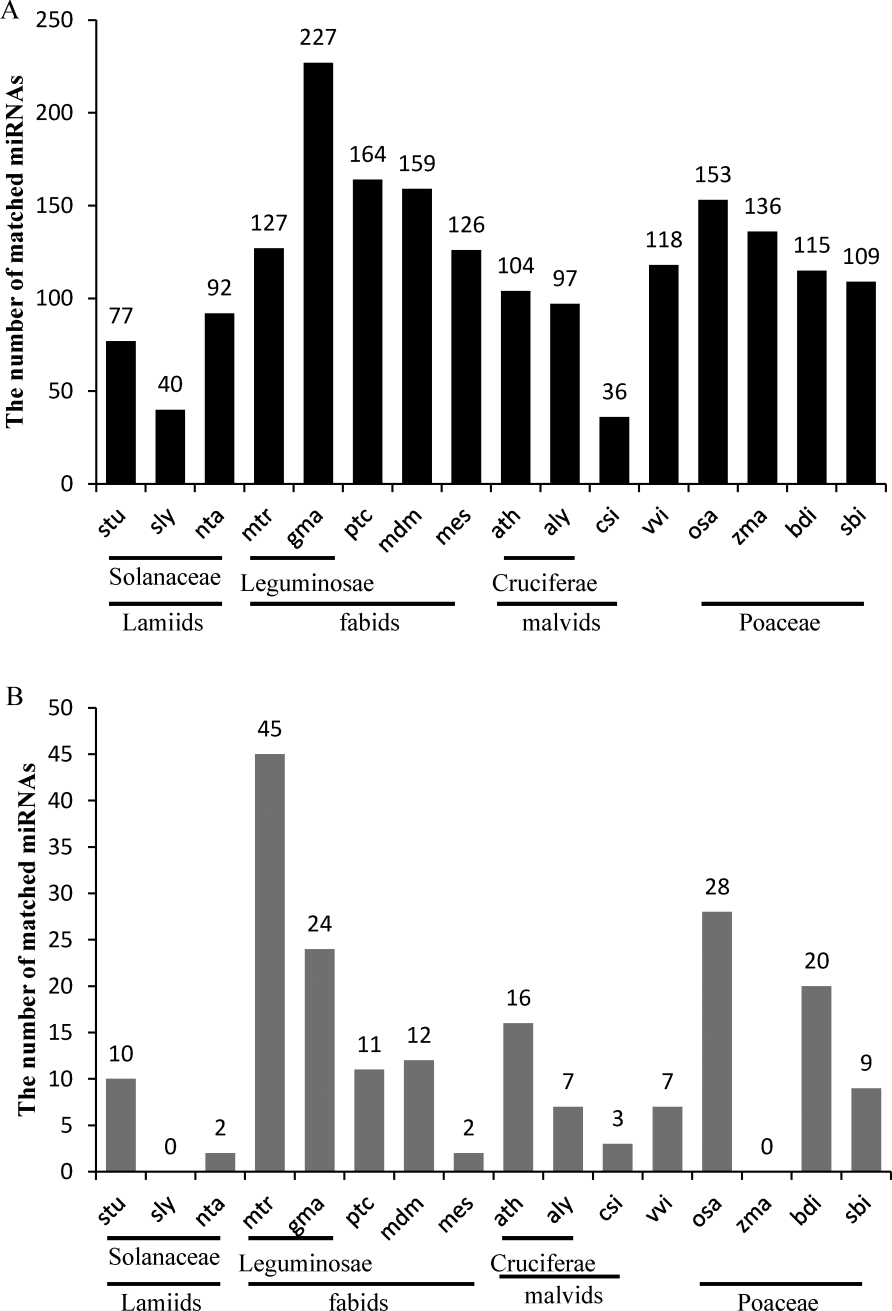
Numbers of cross-species conservative miRNAs with no more than two mismatches. Numbers of cross-species conservative miRNAs with (A) or without (B) conserved miRNAs were counted. Conserved miRNAs include miR156, miR159, miR160, miR162, miR164, miR166, miR167, miR168, miR169, miR171, miR172, miR319, miR390, miR393, miR394, miR395, miR396, miR397, miR398, miR399, miR403 (in Eudicotyledons), and miR408.

The statistical data of the cross-species conservative analysis of known miRNAs was consistent. When we aligned unique sequences to miRBase v21.0 to identify known miRNAs, these unique sequences sometimes matched precursors of multiple miRNAs of multiple species with no more than two mismatches, and these were termed as the cross-species conservative miRNAs of the corresponding *B*. *macrocarpa* miRNAs. Thus, we analyzed the similarities between the miRNAs of *B*. *macrocarpa* and those of other species. The top three species having the greatest number of similar miRNAs were *G*. *max*, *Populus trichocarpa* and *Malus domestica* (227, 164 and 159, respectively) belonging to the fabids. Additionally, there were 127 miRNAs of *M*. *truncatula*, 104 of *A*. *thaliana* and 97 of *A*. *lyrata* that were similar to those of *B*. *macrocarpa* (Fig 6A).

Most of these miRNAs were conserved miRNAs [11] that exist widely in the Eudicotyledons. Additionally, the greatest numbers of non-conserved miRNAs were found in *M*. *truncatula*, *G*. *max* and *A*. *thaliana* (45, 24 and 16, respectively) (Fig 6B). In conclusion, the relationship between the *B*. *macrocarpa* microRNAome and *M*. *truncatula*, *G*. *max* or *A*. *thaliana*, belonging to the rosids, may be close. By contrast, the relationship between the *B*. *macrocarpa* microRNAome and *Nicotiana tabacum*, *Solanum lycopersicum* or *Solanum tuberosum*, belonging to the Solanaceae, may be distant because they have few miRNAs that are similar to those of *B*. *macrocarpa* (2, 0 and 7, respectively) (Fig 6B), and there are no miRNAs enriched in the *Solanaceae* as in *B*. *macrocarpa*, such as miR1919, miR4376 and miR5300 [10].

In summary, we have uncovered useful information for miRNA research in other species of the genus *Beta* and for plant evolutionary analyses. There were also some miRNAs of *Poaceae* plants, most with low expression levels, that were similar to *B*. *macrocarpa* miRNAs, but the relationship between Monocotyledons and Eudicotyledons is complex.

The microRNAome of *B*. *macrocarpa* has species specificity. The lineage-specific miRNAs in *B*. *macrocarpa* were investigated by re-evaluating potential novel miRNAs (reads > 20 in L/VL library) with -3p and -5p according to the stringent criteria reported by Taylor et al. [9]. To validate the lineage-specific miRNAs, we aligned the precursor sequences to the public database in NCBI, and we found that no miRNA gene was supported by other plant species data sets, but most of them were found in *B*. *vulgaris* (Table 2). Finally, 8 possible lineage-specific miRNAs were identified, which could have evolved within the genus *Beta* (S2–S5 Files).

**Table 2:** Summary of lineage-specific miRNAs in *B*. *macrocarpa*.

miRNA name	Length	MFEI	Norm-L	Norm-VL	*B*. *macrocarpa*	*B*. *vulgaris*	other species	microarray
miRn14-3p	21	1.5	14	56	Found	Found	Not found	Y
miRn14-5p	21	1.5	87	192	Found	Found	Not found	Y
miRn23-3p	21	1.5	43	40	Not found	Found	Not found	Y
miRn23-5p	21	1.5	26	37	Not found	Found	Not found	N
miRn31-3p	22	1.3	1876	3606	Found	Found	Not found	Y
miRn31-5p	21	1.3	1405	4415	Found	Found	Not found	Y
miRn33-3p	21	1.1	69	29	Not found	Found	Not found	Y
miRn33-5p	21	1.1	16	10	Not found	Found	Not found	N

### The differential expression analysis of miRNAs responsive to BNYVV infection was confirmed by a microarray analysis and stem-loop RT-qPCR

Genome-wide profiling of miRNAs revealed that 103 known miRNAs (with reads > 20 in L or VL library), representing 36 miRNAs families, and 45 novel miRNAs were differentially regulated by at least a two-fold change in BNYVV-infected *B*. *macrocarpa* compared with the mock-inoculated control (|log_2_(VL/L)| > 1.0). The expression levels of 20 known and 20 novel miRNAs were inhibited during BNYVV infection (S4 Table; S6 File).

To determine the reliability of small RNA sequencing, a microarray analysis was performed with 158 probes of conserved miRNAs from the phylogenetic analysis (80), other known miRNAs (18), and potential novel miRNAs (60) with reads > 20 in L/VL library (S5 Table). The raw sequence data have been deposited in NCBI database with the accession number GSE102330. The expression of 94 miRNAs (hybrid signal > 500 in L or VL microarray library) was further analyzed. Results showed that the fold change of expression obtained by microarray analysis was not completely consistent with small RNA sequencing results, mainly due to the different principles and sensitivity of the two technologies, but the trend of 39 out of 55 significantly differential expressed miRNAs was similar (S5 Table highlighted in yellow). And the trend of the other 16 miRNAs was inconsistent (S5 Table highlighted in red) between microarray analysis and small RNA sequencing. We think there were two possible reasons for such phenomenon. One is that microarray analysis hardly accurately distinguishes the miRNA sequences at the ends of the bases, such as miR159_R+1_1ss21AT, miR159a_R-1_1ss1TG and miR156t, and the other is that two kinds of strong fluorescent signals might mask the difference between each other, such as miR166a_1ss21CT, miR166a and miR535d_1ss7CT.

**Fig 7:**
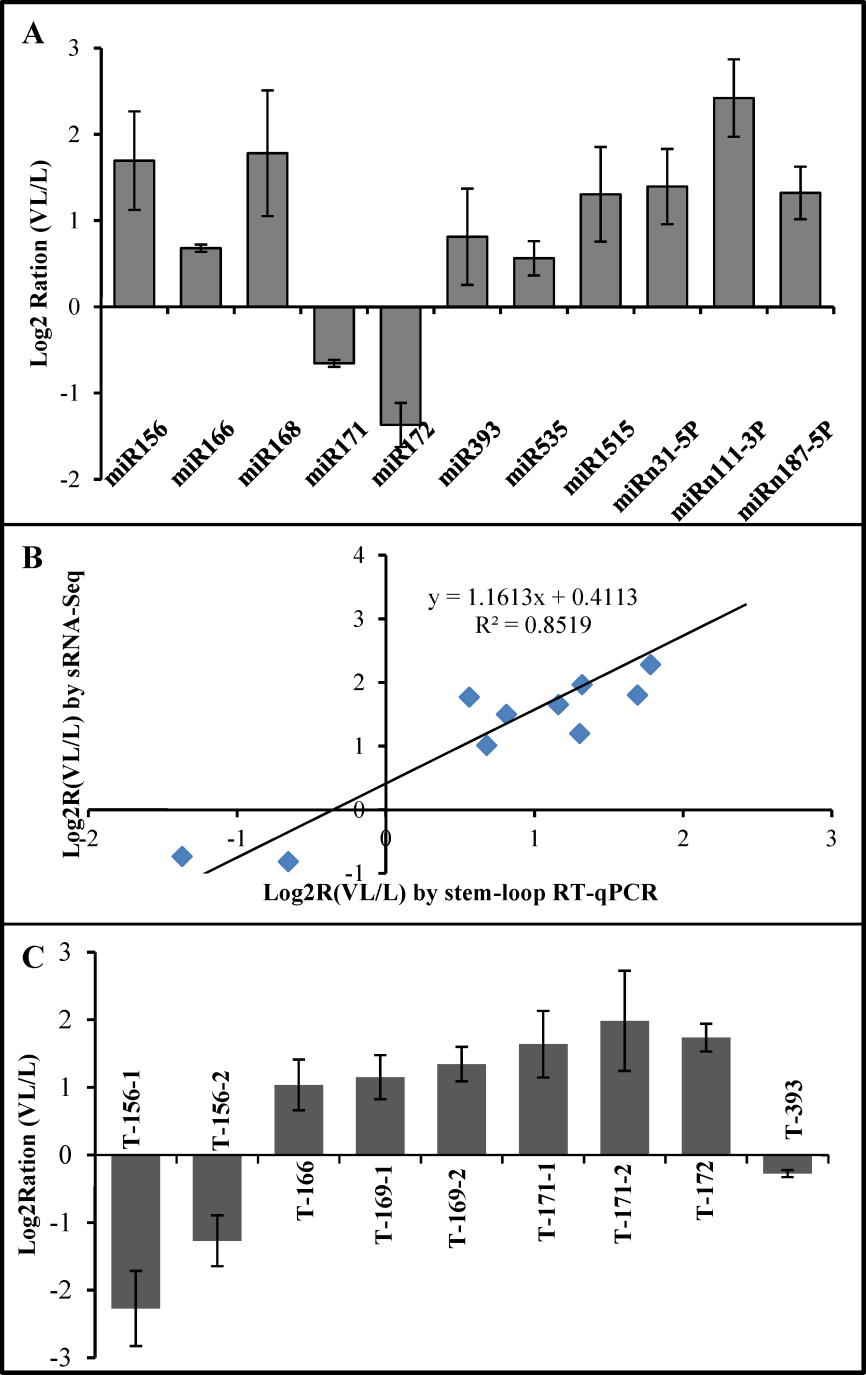
Validation of the relative expression levels of selected miRNAs and their targets by RT-qPCR. (A) Expression patterns of selected *B*. *macrocarpa* miRNAs in response to BNYVV infection by stem-loop RT-qPCR. (B) Correlation of the differential expression ratio of selected miRNAs measured by stem-loop RT-qPCR and small RNA-Seq. (C) Expression patterns of the targets of selected miRNAs in response to BNYVV infection by RT-qPCR. PP2A was used as an internal control.

Meanwhile, the stem-loop RT-qPCR was performed to validate the small RNA deep sequencing results. The expression patterns of eight known miRNAs (miR156, miR166, miR168, miR171, miR172, miR393, miR535 and miR1515) and three novel miRNAs (miRn31-5p, miR111-3p and miRn187-5p) were tested. A high correlation (R^2^ = 0.85) was found between stem-loop RT-qPCR and small RNA sequencing (Fig 7A and 7B).Thus, the alterations in miRNA expression detected by small RNA sequencing could reflect the actual miRNA expression changes between BNYVV-infected and mock-inoculated control plants. miRNAs were selectively regulated by BNYVV infection. miR169, miR172 and miR319 were inhibited, and miR156, miR160, miR390, miR162, miR168 and miR393 were induced (Table 3).

**Table 3:** Differentially expressed miRNAs of *B*. *macrocarpa* in response to BNYVV infection.

miRNA name	miRNA sequence	Length	MFEI	small RNA sequencing			microarray
				L	VL	log2(VL/L)	log2(VL/L)
miR156e	TGACAGAAGATAGAGAGCAC	20	0.9	44	154	1.8	1.21
miR156h-3p	GCTCTCTATGCTTCTGTCATC	21	0.9	679	2681	1.98	1.33
miR160a-3p	GCGTATGAGGAGCCAAGCATA	21	1.1	3	74	4.49	3.53
miR162	TCGATAAACCTCTGCATCCAG	21	0.7	410	1099	1.42	0.66
miR166e-5p	GGAATGTTGGCTGGCTCGAGG	21	0.9	143	1657	3.53	2.57
miR167d-3p	GATCATGTGGTAGCTTCACC	20	0.9	59	239	2.03	0.79
miR168b	TCGCTTGGTGCAGGTCGGGAA	21	0.6	2082	10113	2.28	1.84
miR168c-3p	CCCGCCTTGCATCAACTGAAT	21	0.8	299	5034	4.08	3.03
miR169a	CAGCCAAGGATGACTTGCCGG	21	1.3	6377	2013	-1.66	-0.64
miR171d	TGATTGAGCCGTGCCAATATC	21	1	409	232	-0.81	-0.54
miR172b	CGAATCTTGATGATGCTGCAT	21	1.1	1745	1049	-0.73	-0.81
miR172c-5p	GCAGCATCTTCAAGATTCACA	21	1.1	1143	3165	1.47	2.01
miR319a-3p	TTGGACTGAAGGGAGCTCCC	20	0.7	1341	596	-1.17	-0.20
miR390a-3p	CGCTATCTATCCTGAGTTTCA	21	0.8	52	1813	5.13	2.54
miR390a-5p	AAGCTCAGGAGGGATAGCGCC	21	1	84	551	2.72	1.63
miR393a	TCCAAAGGGATCGCATTGATT	21	1.1	31	63	1.04	1.99
miR393b-3p	ATCATGCAATCCCTTTGGATT	21	1.1	50	883	4.14	2.97
miR395a	CTGAAGTGTTTGGGGGAACTT	21	1	358	999	1.48	0.61
miR395e-5p	GTTCCTCTGAGCACTTCATT	20	1.2	21	241	3.54	1.76
miR396b-3p	GTTCAATAAAGCTGTGGGAAG	21	1.3	2452	19526	2.99	3.11

#### Analysis of the predicted target genes of differentially expressed miRNAs

Degradome sequencing [34] was performed to investigate the targets of miRNAs. One library was constructed from leaves of BNYVV-infected (VL) plants. Through the degradome sequencing approach, we obtained about 7,678,479 raw reads, of which 37,371 reads were perfectly matched to the beet genome.

After further analysis, 32 target genes for 15 known miRNA families and 4 target genes for 4 novel miRNA were identified (Table 4). The sequences of candidate targets and the results of t-plot were shown in S7 and S8 Files. The cleaved targets have been categorized into five classes (Categories 0, 1, 2, 3 and 4) according to the abundance of degradome tags at the target sites [35]. In our study, we listed Categories 0–3 targets, and omitted Category 4 targets which have only one raw read at the predicted cleavage position. Our results substantiate that conserved plant miRNAs regulate conserved targets at identical target sites in species they exist in [12]. This could be manifested by the miR156’s squamosa promoter-binding-like protein, miR162’s endoribonuclease Dicer homolog 1 and miR393’s transport inhibitor response 1.

**Table 4:** Targets of known and putatively novel miRNAs in *B*. *macrocarpa*.

miRNA family	Gene ID	Clevage Site	Score/ Category	Normal reads	Annotation
miR156/157	comp31576_c0	797	1/1	3	SPL9
	comp46911_c1	1183	2/0	3	SPL16
	comp47891_c0	1446	1/2	4	SPL12
	comp41255_c0	1062	2/2	2	SPL6
	comp39747_c0	868	3.5/2	2	isoflavone 2'-hydroxylase-like
miR159	comp26487_c0	594	3.5/0	6	transcription factor GAMYB-like
miR160	comp48868_c0	2279	0.5/0	103	auxin response factor 18-like
miR162	comp51493_c0	2516	2/2	5	DCL1
miR165/166	comp36994_c0	989	3/0	37	homeobox-leucine zipper protein REVOLUTA
	comp51129_c0	1532	2.5/0	20	homeobox-leucine zipper protein ATHB-15-like
miR169	comp41128_c0	1306	4/1	2	NF-YA-10-like
	comp45596_c1	1044	4/1	5	ARF-GAP domain 7 isoform 2
	comp41850_c0	1316	3/2	7	NF-YA-1
	comp45352_c0	691	4/2	37	methyltransferase-like protein 22
	comp32005_c0	650	4/0	4	predicted protein
miR171	comp39988_c1	551	0/0	55	scarecrow-like protein 15-like
	comp48892_c1	2105	1/0	79	scarecrow-like protein 22-like
miR172	comp24578_c0	91	3/0	2	floral homeotic protein APETALA 2
	comp41046_c0	400	3.5/2	3	hypothetical protein ARALYDRAFT
miR393	comp45777_c0	1593	2/0	1468	TRANSPORT INHIBITOR RESPONSE 1
miR394	comp40897_c0	1337	1/2	4	F-box only protein 6
miR396	comp16637_c0	111	3/0	98	Growth-regulating factor 7
	comp46504_c0	848	3.5/2	5	predicted protein, partial
	comp35011_c0	159	3.5/0	12	Growth-regulating factor 9
	comp41306_c0	949	4/1	16	Growth-regulating factor 1
	comp1228726_c0	56	4/0	69	hypothetical protein SORBIDRAFT
miR408	comp30637_c0	84	1/0	20	Basic blue protein, putative
	comp37963_c0	104	1.5/0	164	Basic blue protein, putative
	comp39319_c0	652	2.5/0	148	stellacyanin-like
MIR1103	comp40908_c0	403	4/2	3	chlorophyll a/b-binding protein CP29
MIR1436	comp44948_c2	846	3.5/3	1	predicted protein
miR5205	comp14269_c0	157	3.5/1	2	No annotation
miRn14-5p	comp39238_c0	197	4/2	3	E3 ubiquitin-protein ligase
miRn111-3p	comp45717_c0	422	4/2	2	predicted protein
miRn31-3p	comp17946_c0	248	2/0	1923	disease resistance protein RGA3-like
miRn235-5p	comp301241_c0	59	2.5/1	2	No annotation

To investigate the functions of these miRNAs in response to BNYVV infection, the differential expression patterns of the target genes of miR156, miR166, miR169, miR171, miR172 and miR393 were also examined by RT-qPCR (Fig 7C). And the results showed that the differential expression of these target genes appeared to be negatively related to their respective miRNAs.

In addition, miRNAs regulate gene expression, not only by cleaving targets, but also by inhibiting translation and DNA methylation [6]. Tombusviruses infections enhance the accumulation of miR168, a regulator of ARGONAUTE1 (AGO1) mRNA, and, in parallel, induce the expression of AGO1 mRNA, which has an inhibited translational capacity, and the infection decreases the AGO1 content, resulting in disturbance of its anti-viral function [36].

#### Roles of the miRNA targets’ gene-mediated expressions in plant defenses

BNYVV infections also disturbed *B*. *macrocarpa’*s defense system. The RNA silencing pathway is an efficient way to protect plants from viral infection. miR162-controlled endoribonuclease Dicer homolog 1, miR168-controlled AGO1 and miR403-controlled AGO2 are positive factors participating in the RNA silencing pathway [36]. In BNYVV infected *B*. *macrocarpa*, miR162 and miR168 were induced (VL/L > 1.8), thus impairing the plant’s antiviral capability and enhancing viral infection and proliferation (Table 3).

#### Interactions of miRNA-target-mediated gene expression levels responsible for plant morphological changes

BNYVV infections lead to chlorosis, dwarfism and enhanced axillary bud development [28]. miR395 mediated regulation of sulfate accumulation and allocation in *Arabidopsis thaliana*. Overexpression of miR395 inhibited the process of sulfur assimilation, and led plants display sulfur deficiency symptoms. In BNYVV-infected *B*. *macrocarpa*, miR395 was significantly up-regulated, and might play a role in the formation of chlorosis and dwarfism symptoms [37]. The abnormal development of axillary buds may result from the increased abundance of miR156. Up-regulated miR156 can disturb gibberellin levels [38], and exhibit enhanced branching from axillary buds resulting in a bushy appearance [39–42]. Additionally, miR156 was induced by the BNYVV infection and may be responsible for abnormal axillary bud development. miR156 is a switch between vegetative and reproductive growth in plants through its regulation of the ‘miR156-squamosa promoter-binding-like protein–miR172- AP2-like factor’ pathway. The function of the miR172’s target is to inhibit flowering. The up-regulated miR156 (VL/L > 4) and the corresponding down-regulated miR172 (VL/L < 0.6) may retain the vegetative state by delaying the flowering time [43, 44]. Up-regulated miR169 plays a key role in stress-induced early flowering in *A*. *thaliana* [45], and in BNYVV-infected *B*. *macrocarpa*, miR169 (VL/L < 0.3) was inhibited, delaying flowering, which correlated with the miR156 regulatory pathway.

#### BNYVV infection altered miRNAs participating in plant hormone synthesis and signal transduction pathways

The plant hormone signaling pathway plays an important role in plant growth and defense. Auxin is related to plant height, root development and apical dominance. miR160, miR390 and miR393 control plant growth by regulating the auxin signaling pathway [46, 47]. miR390 and miR393 were up-regulated (VL/L > 2.2) by BNYVV infection, and the target of miR393, transport inhibitor response 1, was inhibited. All of these may function in the formation of dwarf symptoms. The JA signaling pathway functions in plant disease resistance and insect resistance. miR319-controlled TEOSINTE BRANCHED/CYCLOIDEA/PCF transcription factors control the biosynthesis of the hormone JA by affecting the expression levels of JA biosynthetic genes and is proposed to coordinate two sequential processes in leaf development: leaf growth, which they negatively regulate, and leaf senescence, which they positively regulate [15, 48]. In BNVV-infected *B*. *macrocarpa*, miR319 was inhibited (VL/L < 0.4), which might enhance plant defense by increase the JA content, and might also take part in leaf area reduction.

## Conclusions

miRNA expression profiles in *B*. *macrocarpa* during viral infection were investigated by small RNA sequencing, and were further validated by microarray analysis and stem-loop RT-qPCR. In *B*. *macrocarpa*, 547 known miRNAs, representing 129 miRNA families, and 282 potential novel miRNAs were identified, and a phylogenetic analysis showed 8 lineage-specific miRNAs in the genus *Beta*. The differential expressions of 103 known miRNAs, representing 38 miRNA families, and 45 potential novel miRNAs had at least a two-fold change in response to BNYVV infection, and most of the miRNAs were involved in the auxin signal pathway, jasmonate biosynthesis, and plant defense. Thus, the BNYVV infection disturbed the miRNA pathway of *B*. *macrocarpa*, which may be favorable for viral symptom development. Our results revealed miRNAs involved in the BNYVV infection, which would be helpful for further study of the molecular mechanisms underlying BNYVV-plant interactions.

## Materials and methods

### Plants, viral inoculations and detection

*B*. *macrocarpa* plants were grown in a controlled-environment chamber at 24 ± 1°C with 16 h of illumination and 8 h darkness per day. BNYVV (RNAs 1+2+3+4+5) was a mixture of total RNAs (2 μg) from ‘BN34’ (RNAs 1+2+3+4) preserved in our laboratory [49], and *in vitro* transcript of RNA5 (1 μg). BNYVV RNAs were then mixed with equal volume of inoculation buffer (50 mM glycine, 30 mM K_2_HPO_4_, 1% bentonite, and 1% celite, pH 9.2), and rubbed onto one leaf of *B*. *macrocarpa*. Inoculation buffer without the addition of RNAs served as the control. Three leaves per plant were inoculated. At 15 days post inoculation (dpi), western blot analysis of the BNYVV coat protein (CP) was performed using symptomatic leaves of BNYVV-inoculated *B*. *macrocarpa* or the healthy control plants (S1 Fig). Rabbit polyclonal antibodies against BNYVV CP had been preserved in our laboratory [28].

### Total RNA extraction

Total RNAs were extracted from systemic leaves of virus-free and BNYVV-infected *B*. *macrocarpa* at 15 dpi using according to previously described methods [28]. RNA integrity and size distribution were examined using a Bioanalyzer 2100 (Agilent Technologies, Palo Alto, CA, USA) and agarose gel electrophoresis (1%). For each group, the RNA pool was prepared by mixing RNA samples (12 μg per sample) from five individual plants.

### Construction and sequencing of small RNA libraries

Small RNA fragments (10–40 nt) were isolated from total RNA sample (2 μg), and two respective small RNA libraries were constructed using IlluminaTruSeqTM Small RNA Sample Preparation Kit (Illumina, San Diego, CA, USA) according to the manufacturer’s instructions. 5he performdaptors was added to the short RNA fragments of each sample followed by RT-PCR. The products were purified and assessed using the Agilent 2100 Bioanalyzer (Agilent Technologies). A clustering of two samples was performed using a cBot Cluster Generation System using TruSeqSE Cluster Kit (Illumina) according to the manufacturer’s instructions. Finally, deep sequencing was performed using the Illumina Hiseq 2000 (Illumina) according to the protocol of the manufacturer.

### Sequencing data analysis

The raw data were retrieved by Illumina’s Sequencing Control Studio software version 2.8 (SCS v 2.8, LC-Bio, Houston, TX, USA), and extracted from the image files generated by Illumina Genome Analyzer Pipeline software (LC Sciences, Hangzhou, China). The clean reads (18–25 nt) were collected after removing junk sequences, error length sequences (< 18 nt and > 25 nt), and sequences mapped to RFam (http://rfam.janelia.org), RepBase (http://www.girinst.org/repbase) and mRNA (http://www.ncbi.nlm.nih.gov/). Additionally, the unique clean reads mapped to miRNA precursors found using a BLAST algorithm-based search against the miRBase v21.0 (htt://mirbase.org) were identified as known miRNAs, allowing up to two mismatches during the alignment. Others were mapped to genomes of *B*. *macrocarpa* [28] or reference genomes of *B*. *vulgaris* in NCBI to identify their precursors. Those with precursors that met the criteria (MFEI > 0.9) were marked as potential miRNAs. RNAfold software (http://rna.tbi.univie.ac.at/cgi-bin/RNAfold.cgi) was used to investigate their secondary hairpin structures. Other criteria are listed in S1 Table.

### Phylogenetic analysis

The MIR160 (precursor of miR160) phylogenetic tree with 34 MIR160s from 29 plant species was constructed by MEGA5.0 using the Maximum Likelihood method according to the manual [50]. These validated MIR160s from various plant species were adapted as reported [9, 10, 33], and their sequences were downloaded from miRBase v21.0. The phylogenetic distribution of the 29 plant species was analyzed by the common tree tool in NCBI (https://www.ncbi.nlm.nih.gov/Taxonomy/CommonTree/). The three-letter codes of the full species names are listed.

### Microarray analysis

The miRNA microarray experiment was performed according to the protocol provided by LC Sciences (LC Sciences, Hangzhou, China). The small RNAs (< 300-nt) were size-fractionated using YM-100 microcon centrifugal filters (Millipore) from 2 μg of total RNA samples. These small RNAs were 3′ extended with a poly(A) tail using poly(A) polymerase and then an oligonucleotide tag was ligated to the poly(A) tail for later fluorescent dye staining. Antisense detection probes for each of the 158 chosen identified miRNAs were made by *in situ* synthesis using photo-generated reagent chemistry. Hybridization was performed overnight on a μParaflo microfluidic chip using a micro-circulation pump (Atactic Technologies, Houston, TX, USA).

Each probe was used three times, arranged in different places on one chip, and three biological replicates were performed. Tag-specific Cy3 and Cy5 dyes were used to label virus-free and BNYVV-infected RNA samples, respectively, and were exchanged during the double-color microarray assays. After RNA hybridization, tag-conjugating Cy3 and Cy5 dyes were circulated through the microfluidic chip for dye staining. Fluorescence images were collected using a laser scanner (GenePix 4000B, Molecular Device, Union City, CA, USA) and digitized using Array-Pro image analysis software (Media Cybernetics, Silver Spring, MD, USA). Background signals were subtracted from the original data followed by normalization of the signals using the LOWESS filter (Locally-weighted Regression).

### Degradome sequencing and analysis

One degradome library was constructed from leaves of BNYVV-infected (VL) plants based on published methods [34]. The cDNA library was sequenced on an Illumina HiSeq 2000 (LC Sciences, Hangzhou, China). CleaveLand 3.0 pipeline and ACGT301-DGEv1.0 program (LC Sciences) were employed for data analysis. The alignment scores ≤ 4 were used as the criteria. Furthermore, based on the abundance of degradome tags at the target sites, the miRNA targets were classified into 5 categories (0, 1, 2, 3, and 4) according to previously described methods [35].

### Reverse-transcription quantitative real-time PCR (RT-qPCR)

To validate the small RNA sequencing results, the relative expression levels of 11 selected miRNAs from BNYVV-infected or mock-inoculated leaves were validated by stem-loop RT-qPCR. Primers for the stem–loop RT-qPCR were designed using methods as previously described [51]. Total RNA (3 μg) was used in the reverse transcription reaction (30 μl). qPCR was performed in 96-well plates using the CFX96 real-time PCR detection system (Bio-Rad, Hercules, CA, USA) with the following temperature program: 95°C for 15 s, followed by 40 cycles of 95°C for 15 s, and then annealing at 60°C for 30 s. Each reaction mixture consisted of 1 μl cDNA, 7 μl SoFast EyaGreen Supermix (Bio-Rad, Hercules, CA, USA), 1.5 μl (3 pmol/μl) of both forward and reverse primers, and 3 μl PCR-grade water [28].

The transcript levels of several target genes of six selected miRNAs were also assayed by RT-qPCR. Reverse transcription of RNA was conducted by oligo (dT_20_). qPCR was conducted as described above. PP2A was used as an internal control. All reactions were performed using three biological replicates for each treatment, and each biological sample of three pooled plants was evaluated with three technical replicates. All primers used in this study are listed in S6 Table.

## Supporting information

S1 Table The expression levels of miRNA in *B*. *macrocarpa* (reads > 3). (XLSX)

S2 Table The list of known miRNAs-3p and -5p. (XLSX)

S3 Table The list of potential novel miRNAs-3p and -5p. (XLSX)

S4 Table Differential expression profiles of miRNAs between mock-, and BNYVV-infected plants (p < 0.05, Fold change > 2, reads more than 20). (XLSX)

S5 Table The results of microarray analysis of 158 miRNAs compared with small RNA deep sequencing. (XLSX)

S6 Table Primers used in RT-qPCR. (XLSX)

S1 File The secondary structure of bma-miR160a (PDF)

S2 File The secondary structure of miRn14-3p and miRn14-5p. (PDF)

S3 File The secondary structure of miRn23-3p and miR23-5p. (PDF)

S4 File The secondary structure of miRn31-3p and miRn31-5p. (PDF)

S5 File The secondary structure of miRn33-3p and miRn33-5p. (PDF)

S6 File Heatmap of differentially expressed miRNAs (P-value < 0.05). (PDF)

S7 File Targets' sequences database. (ZIP)

S8 File t-plot of miRNA-target. (ZIP)

S1 Fig The western blot analysis of BNYVV CP expression in systemic leaves. Mock indicates healthy *B*. *macrocarpa* inoculated with buffer only.
(TIFF)
