# Supplementary figures and images for: Characterization of microRNAs of Beta macrocarpa and their responses to Beet necrotic yellow vein virus infection
Source: PLoS One. 2017 Oct 16;12(10):e0186500. doi: 10.1371/journal.pone.0186500 (PMC5643120; doi:10.1371/journal.pone.0186500)

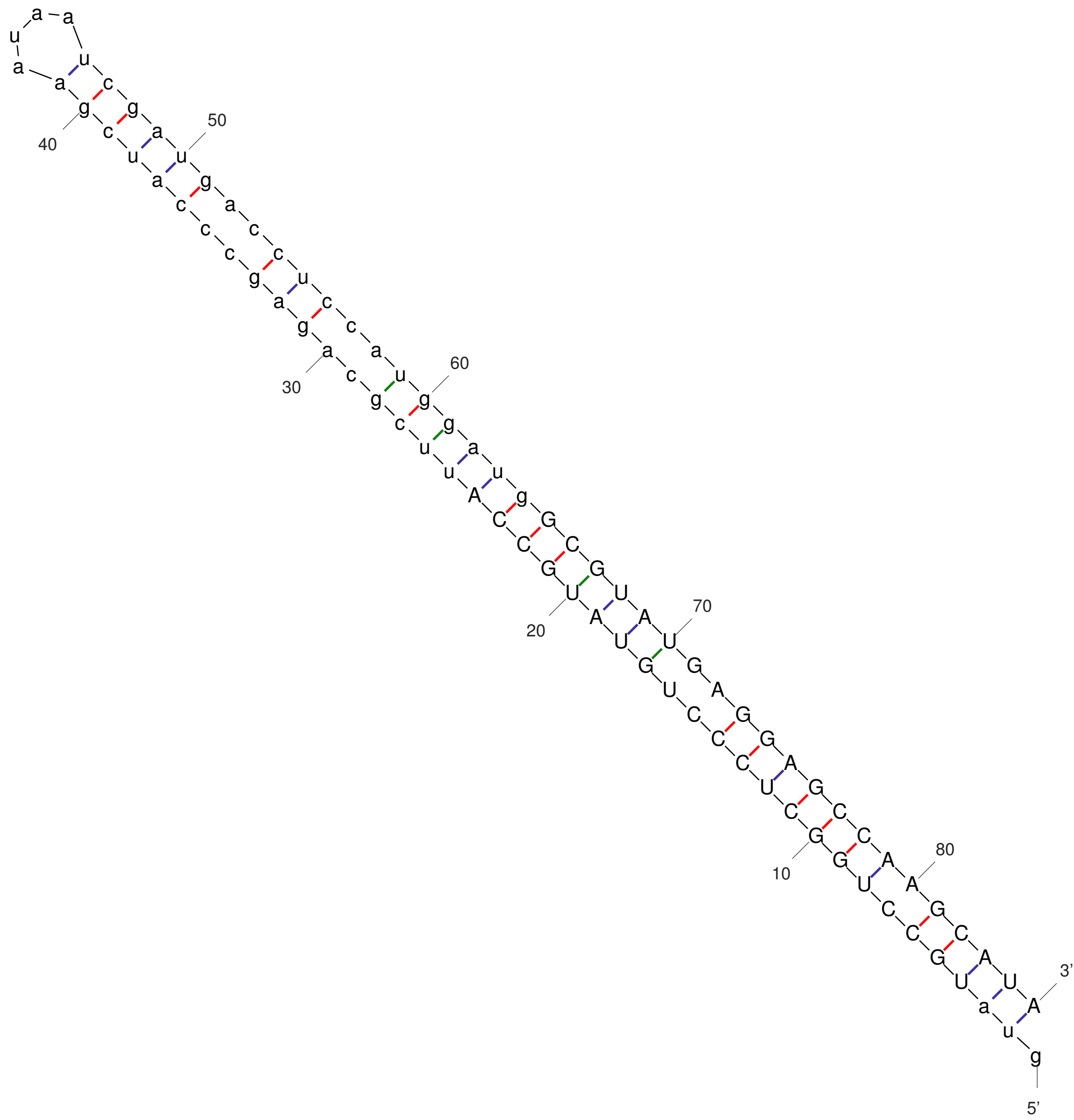

Supplement: S1 File — (PDF) [file pone.0186500.s007.pdf]

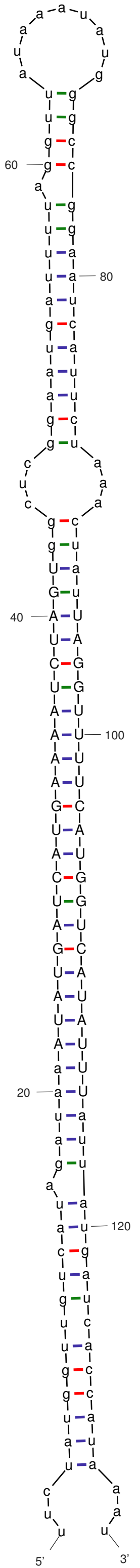

Supplement: S2 File — (PDF) [file pone.0186500.s008.pdf]

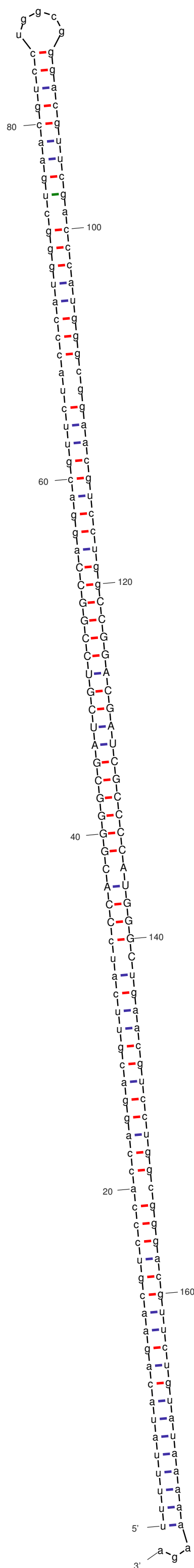

$dG = -162.10$  [Initially -162.10] 17Mar19-05-36-25

Supplement: S3 File — (PDF) [file pone.0186500.s009.pdf]

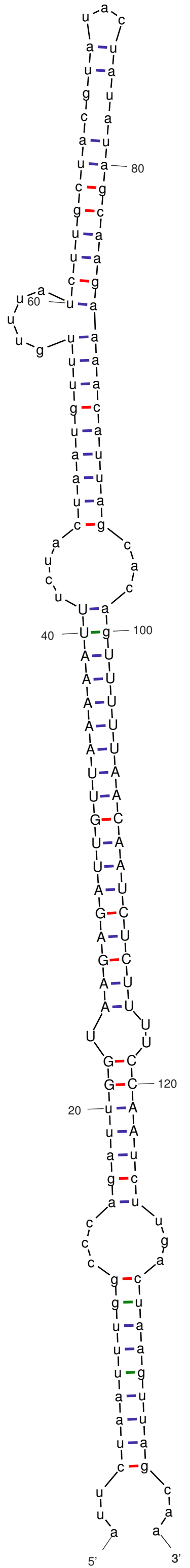

Supplement: S4 File — (PDF) [file pone.0186500.s010.pdf]

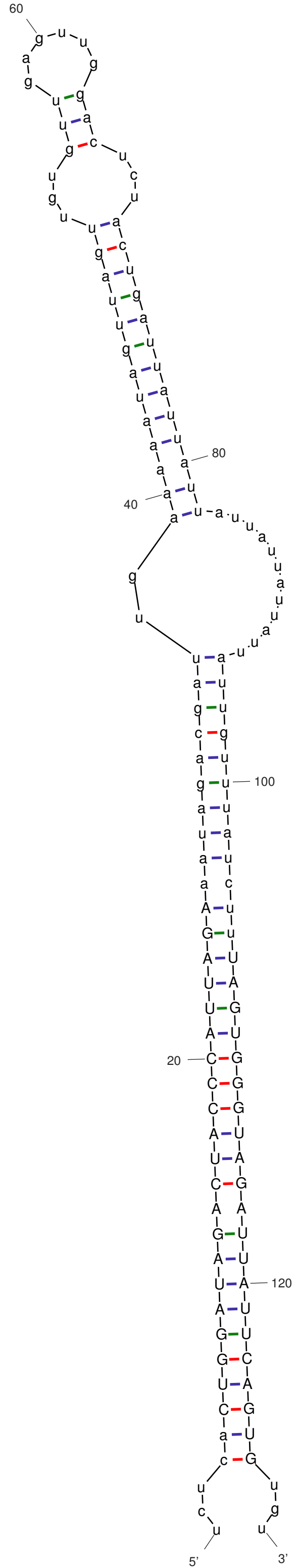

Supplement: S5 File — (PDF) [file pone.0186500.s011.pdf]

# PC-3p-121\_4699 slicing comp17946\_c0 at nt 248

alignment score=2 , category=0 , p=0.00302387622887079

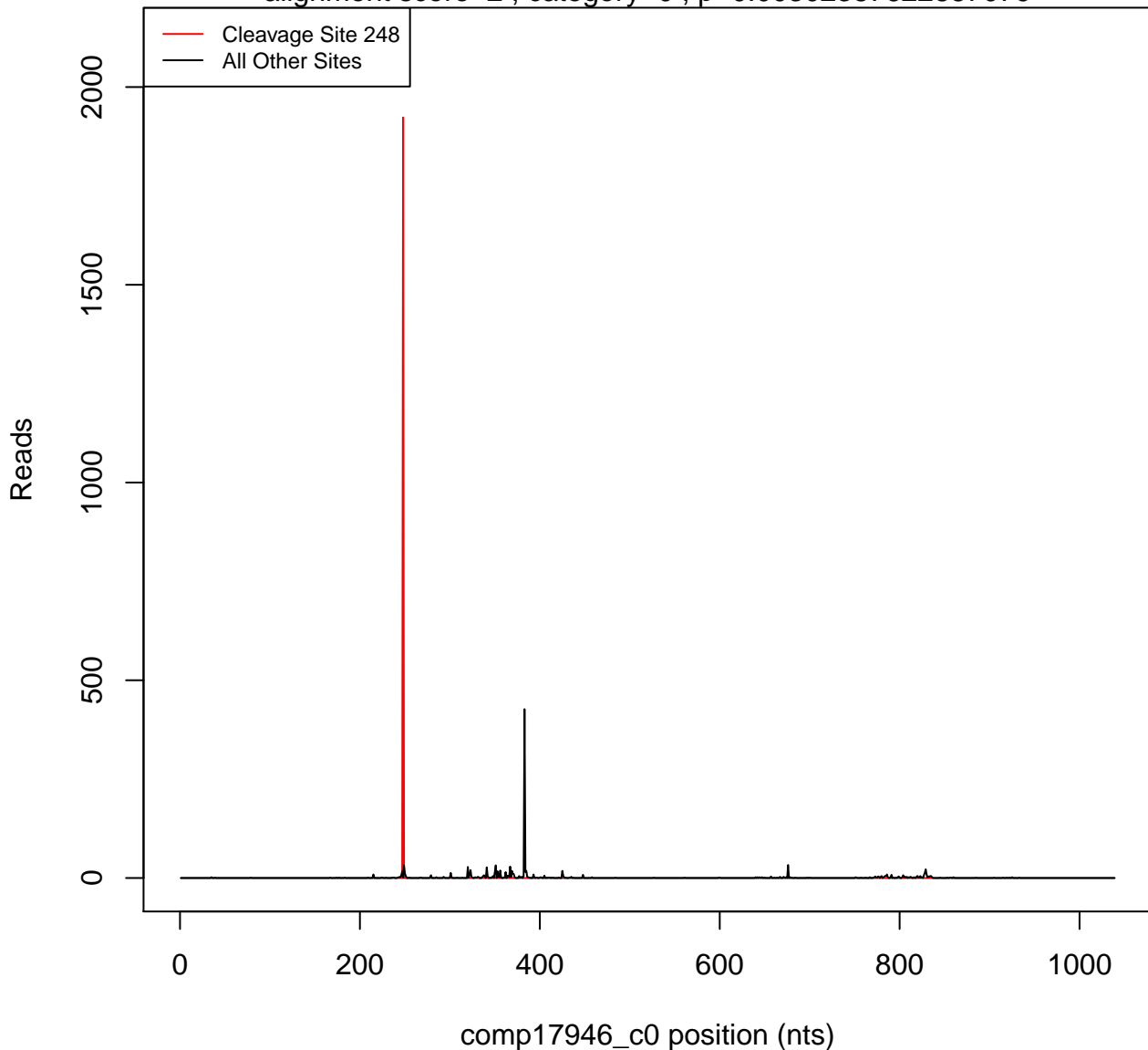

Supplement: S8 File — (ZIP) [file pone.0186500.s014.zip › S8 t-plot of miRNA-target/1comp17946_c0--248--miRn31-3p_degradome.pdf]

# PC-5p-26147\_57 slicing comp14269\_c0 at nt 157

alignment score=3.5 , category=1 , p=0.00803539649841167

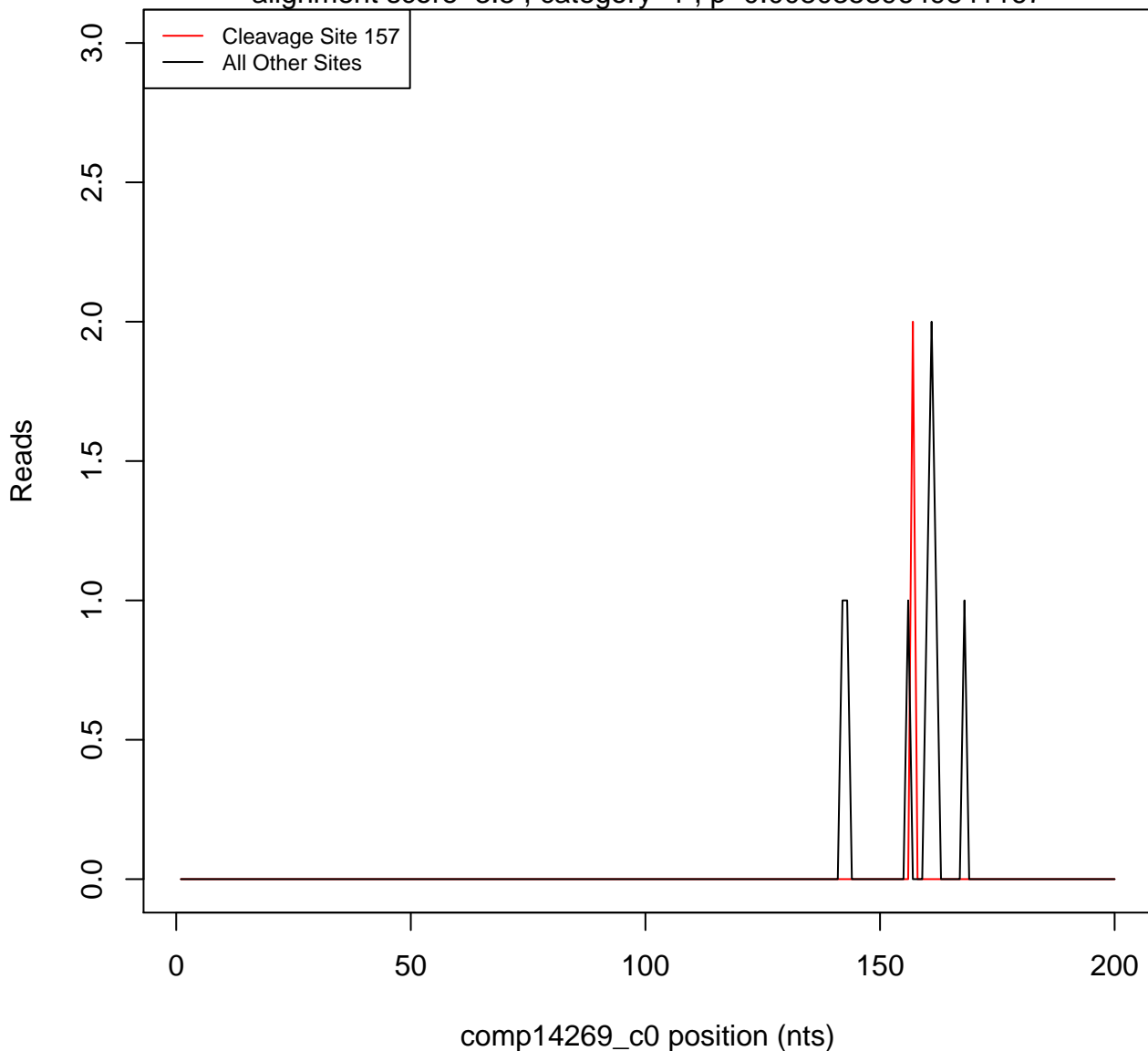

Supplement: S8 File — (ZIP) [file pone.0186500.s014.zip › S8 t-plot of miRNA-target/2comp14269_c0--157--mtr-miR5205a_L-3R+1_1ss6AT_degradome.pdf]

# PC-5p-4730\_207 slicing comp39238\_c0 at nt 197

alignment score=4 , category=2 , p=0.303033976979963

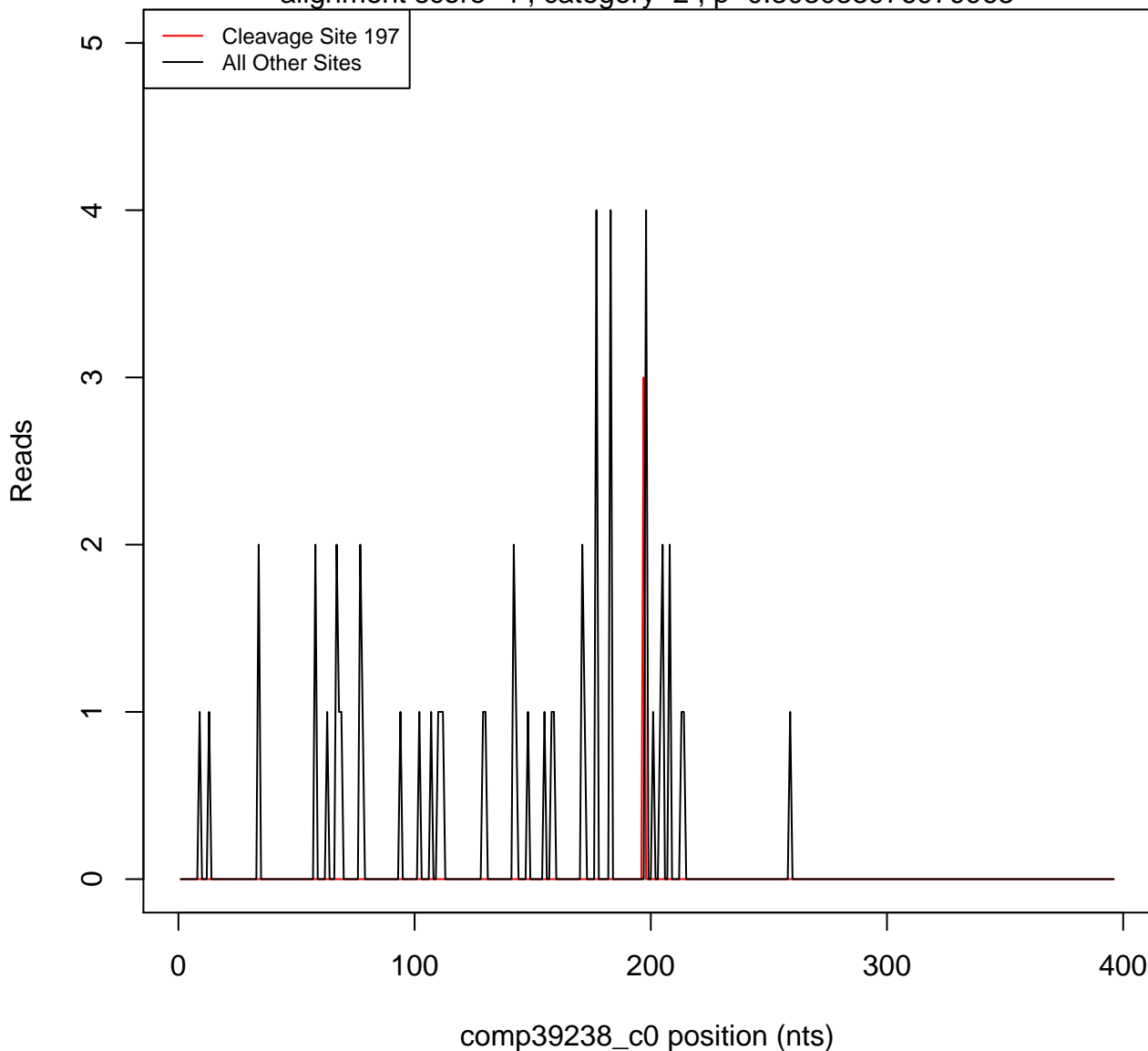

Supplement: S8 File — (ZIP) [file pone.0186500.s014.zip › S8 t-plot of miRNA-target/3comp39238_c0--197--miRn14-5p_degradome.pdf]

# PC-3p-1184\_602 slicing comp45717\_c0 at nt 422

alignment score=4 , category=2 , p=0.872923183999369

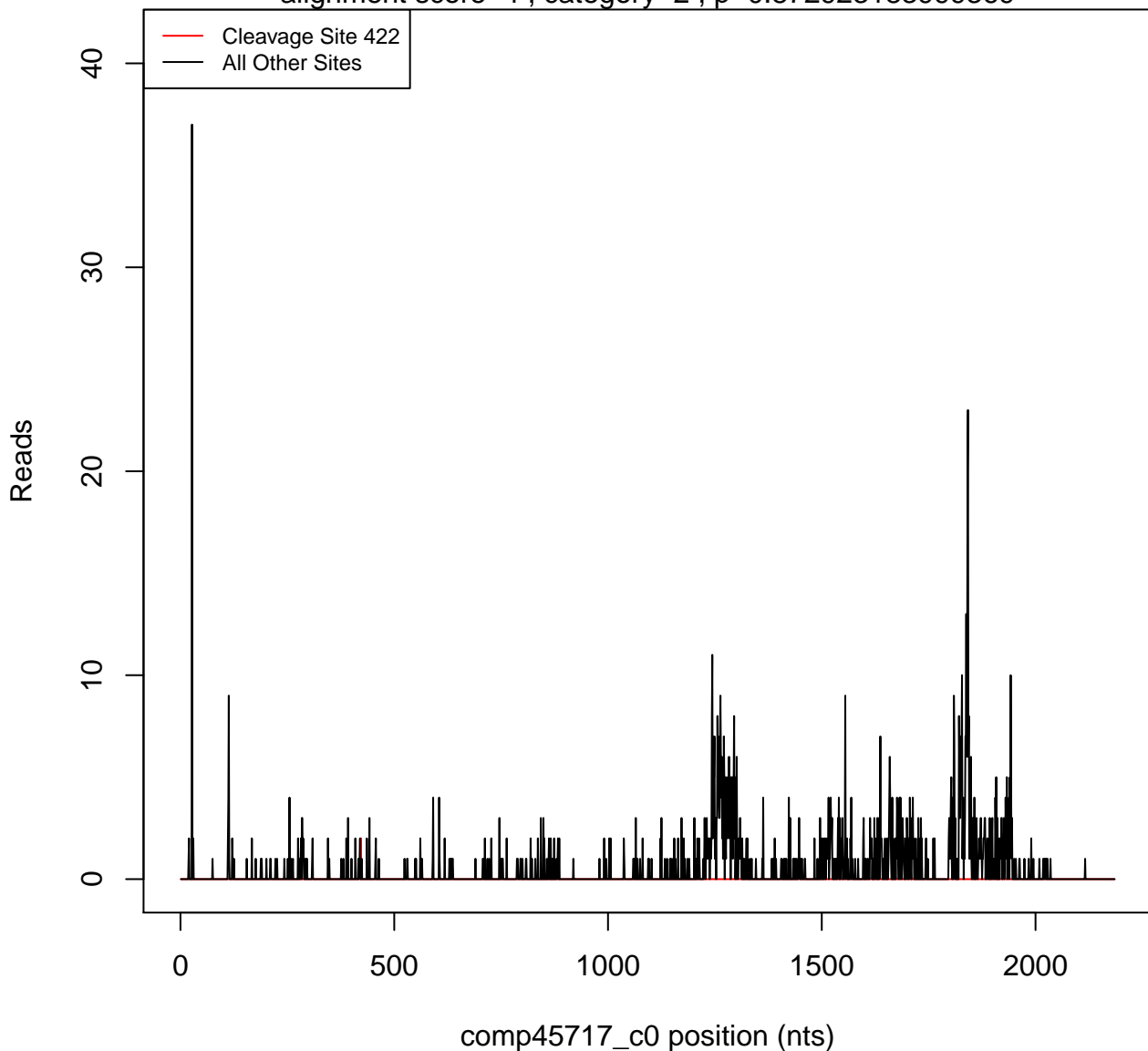

Supplement: S8 File — (ZIP) [file pone.0186500.s014.zip › S8 t-plot of miRNA-target/4comp45717_c0--422--miRn111-3p_degradome.pdf]

# PC-5p-103354\_22 slicing comp301241\_c0 at nt 59

alignment score=2.5 , category=1 , p=0.013356424497778

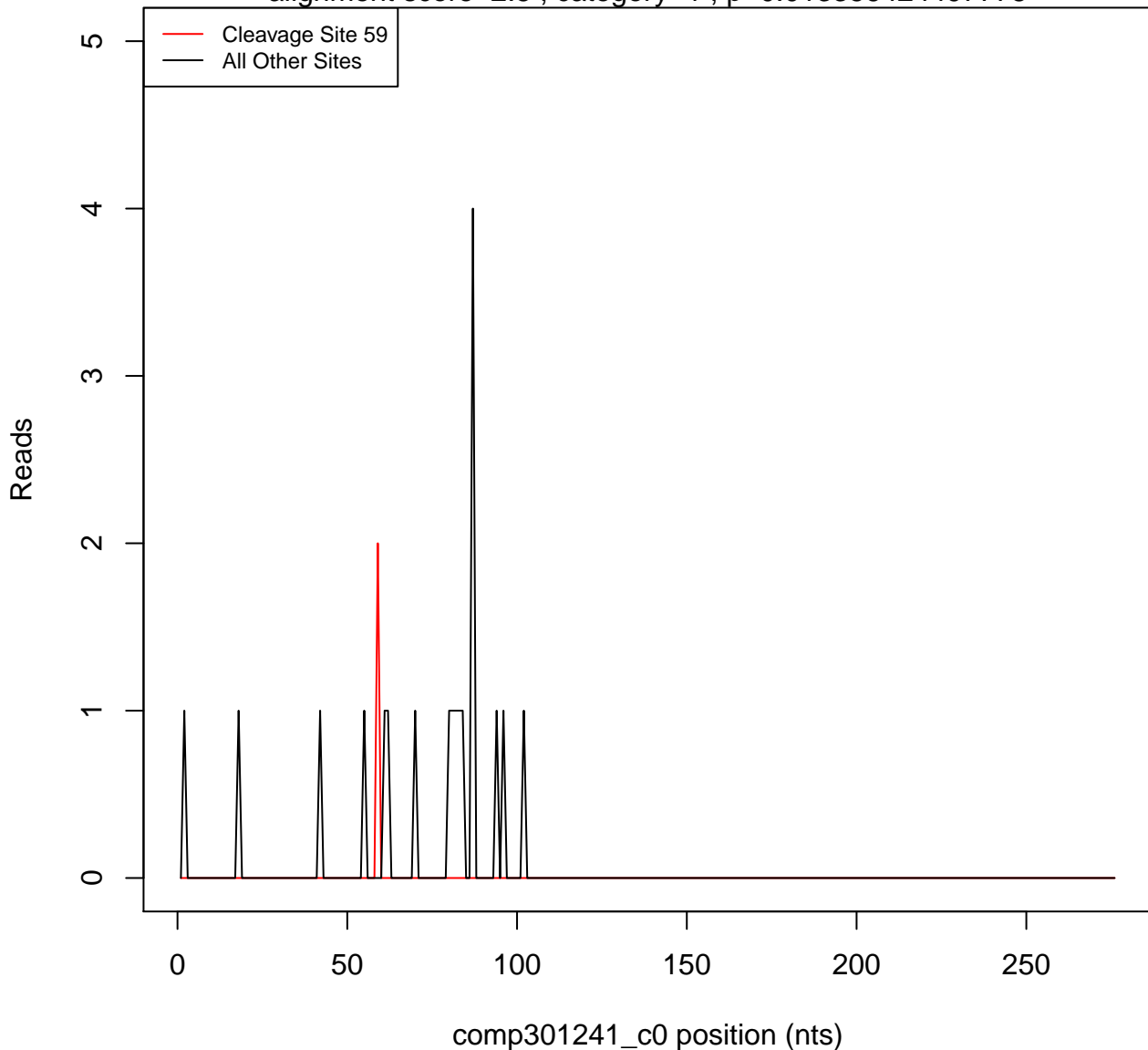

Supplement: S8 File — (ZIP) [file pone.0186500.s014.zip › S8 t-plot of miRNA-target/5comp301241_c0--59--miRn235-5p_degradome.pdf]

# bcy-miR156\_L-1\_2ss13TG16AT slicing comp47891\_c0 at nt 1446

alignment score=1 , category=2 , p=0.174330658653354

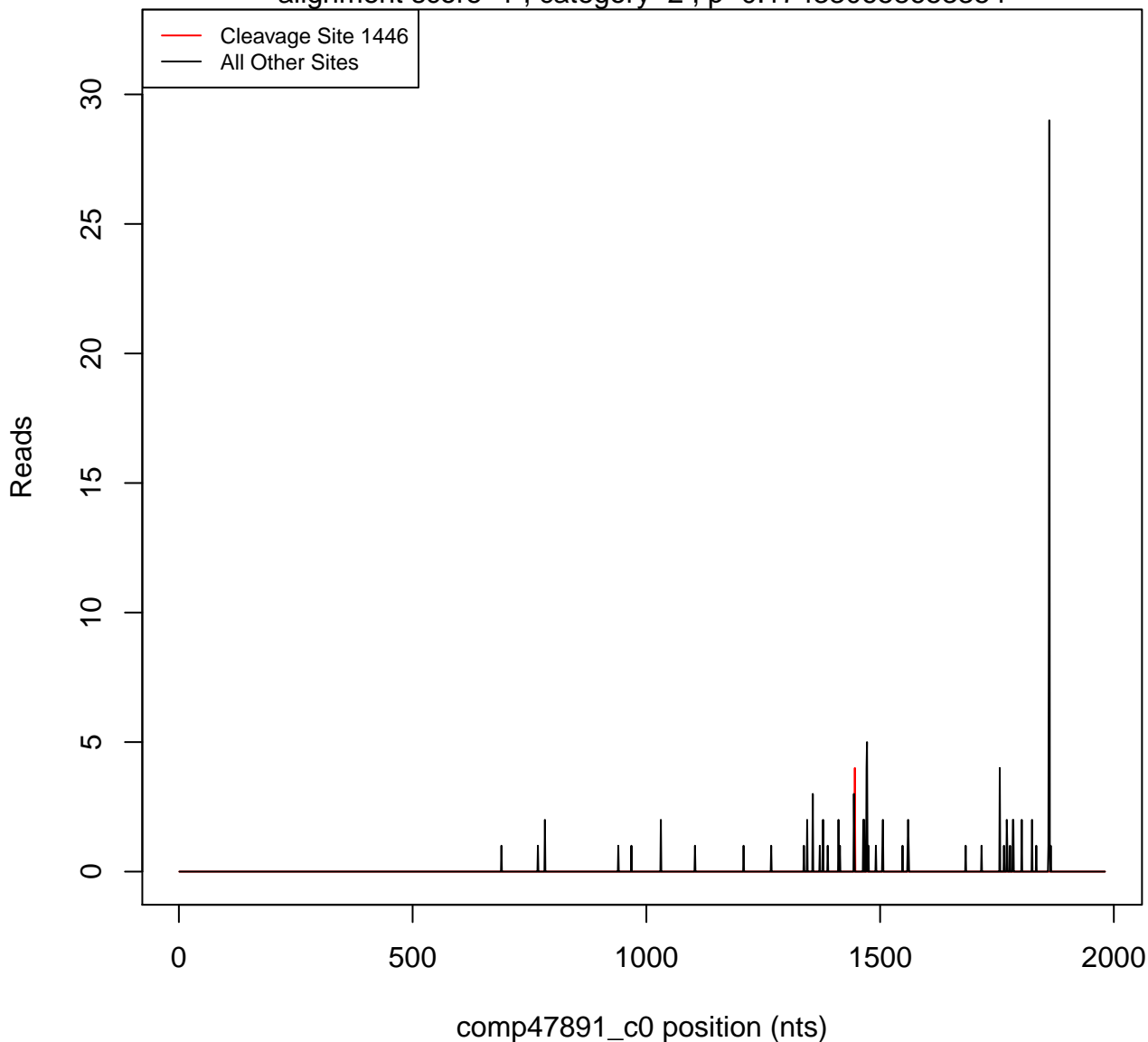

Supplement: S8 File — (ZIP) [file pone.0186500.s014.zip › S8 t-plot of miRNA-target/6comp47891_c0--1446--bcy-miR156_L-1_2ss13TG16AT_degradome.pdf]

# aqc-miR156a slicing comp41255\_c0 at nt 1062

alignment score=2 , category=2 , p=0.333173227452032

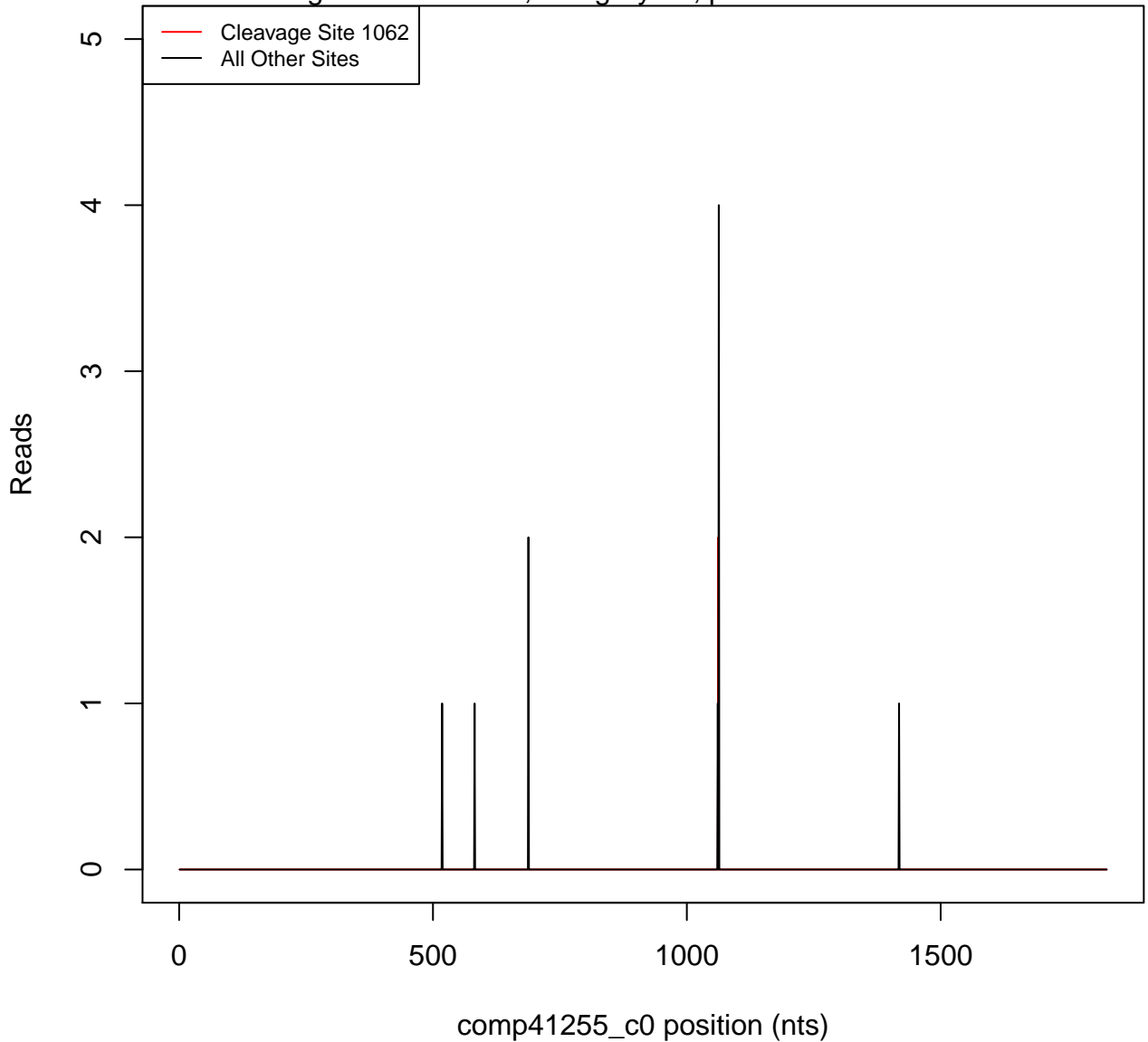

Supplement: S8 File — (ZIP) [file pone.0186500.s014.zip › S8 t-plot of miRNA-target/7comp41255_c0--1062--aqc-miR156a_degradome.pdf]

# ath-miR172b-5p\_R+1\_1ss7CT slicing comp41046\_c0 at nt 400

alignment score=3.5 , category=2 , p=0.162073964060704

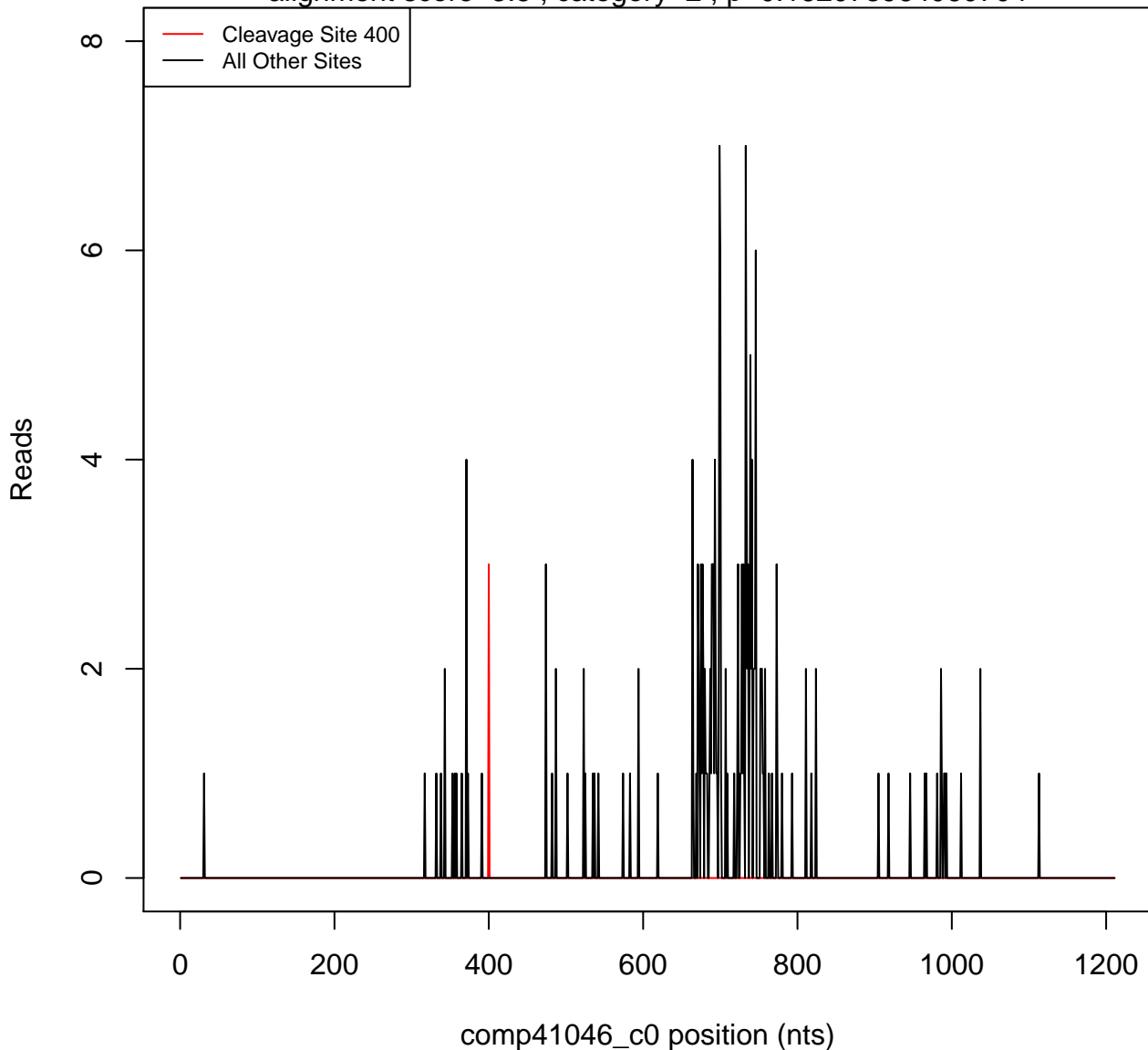

Supplement: S8 File — (ZIP) [file pone.0186500.s014.zip › S8 t-plot of miRNA-target/8comp41046_c0--400--ath-miR172b-5p_R+1_1ss7CT_degradome.pdf]

# ath-miR169b slicing comp41850\_c0 at nt 1316

alignment score=3 , category=2 , p=0.180391609715968

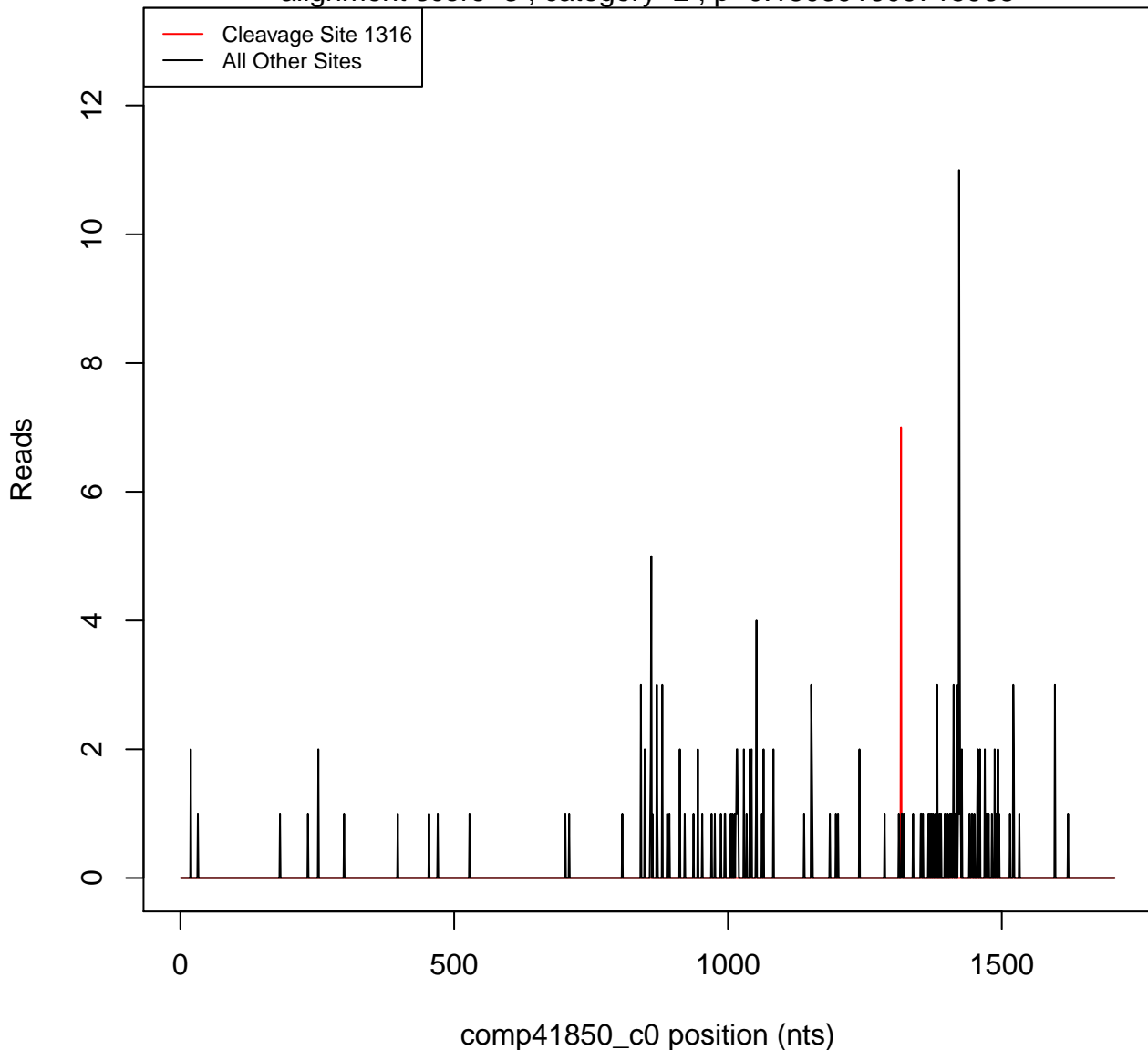

Supplement: S8 File — (ZIP) [file pone.0186500.s014.zip › S8 t-plot of miRNA-target/8comp41850_c0--1316--ath-miR169b_degradome.pdf]

# gma-miR396a-5p\_R+2 slicing comp408434\_c0 at nt 104

alignment score=3 , category=2 , p=0.0641588625669407

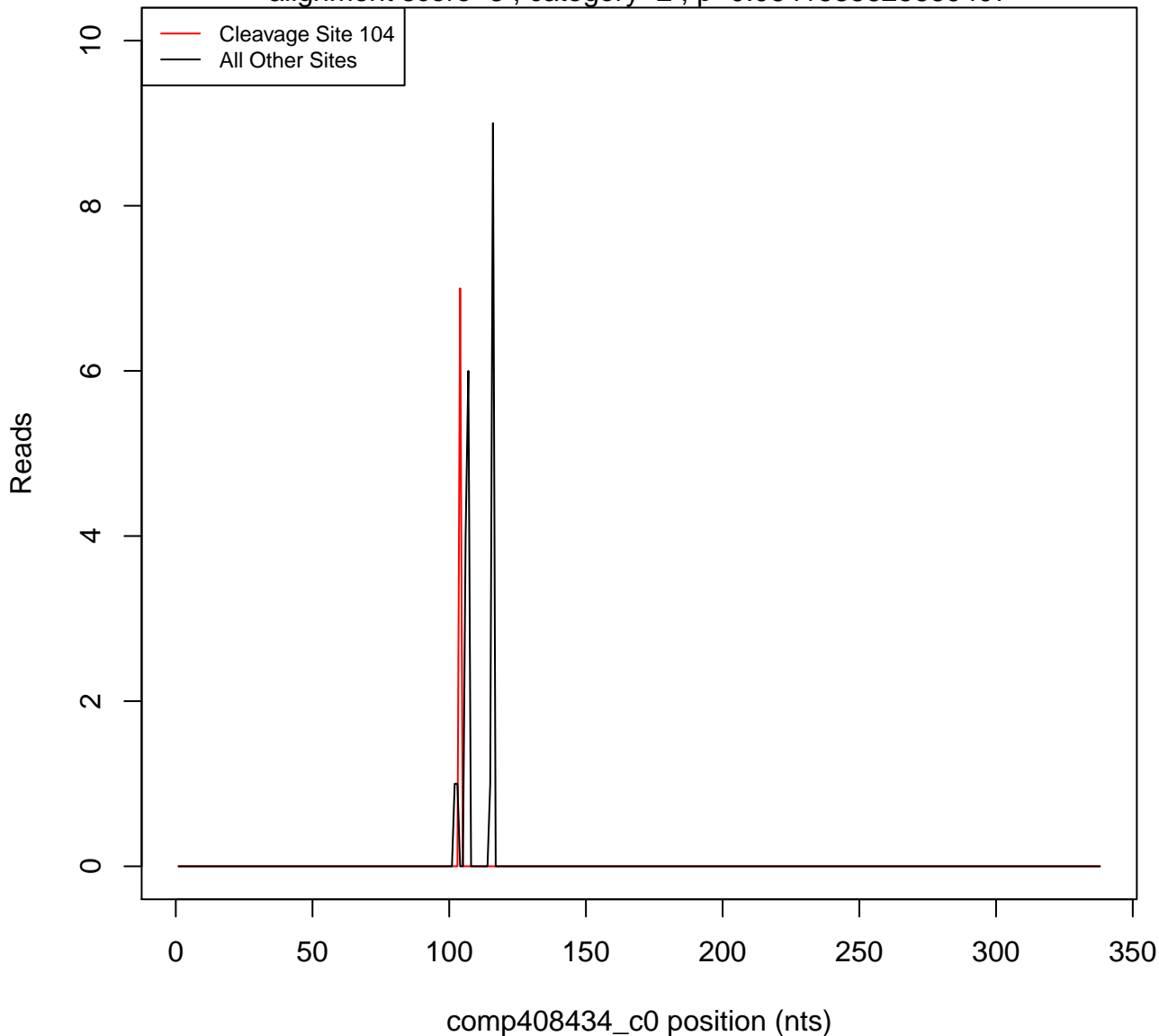

Supplement: S8 File — (ZIP) [file pone.0186500.s014.zip › S8 t-plot of miRNA-target/9comp408434_c0--104--gma-miR396a-5p_R+2_degradome.pdf]

# ath-miR394a slicing comp40897\_c0 at nt 1337

alignment score=1 , category=2 , p=0.0913365081909329

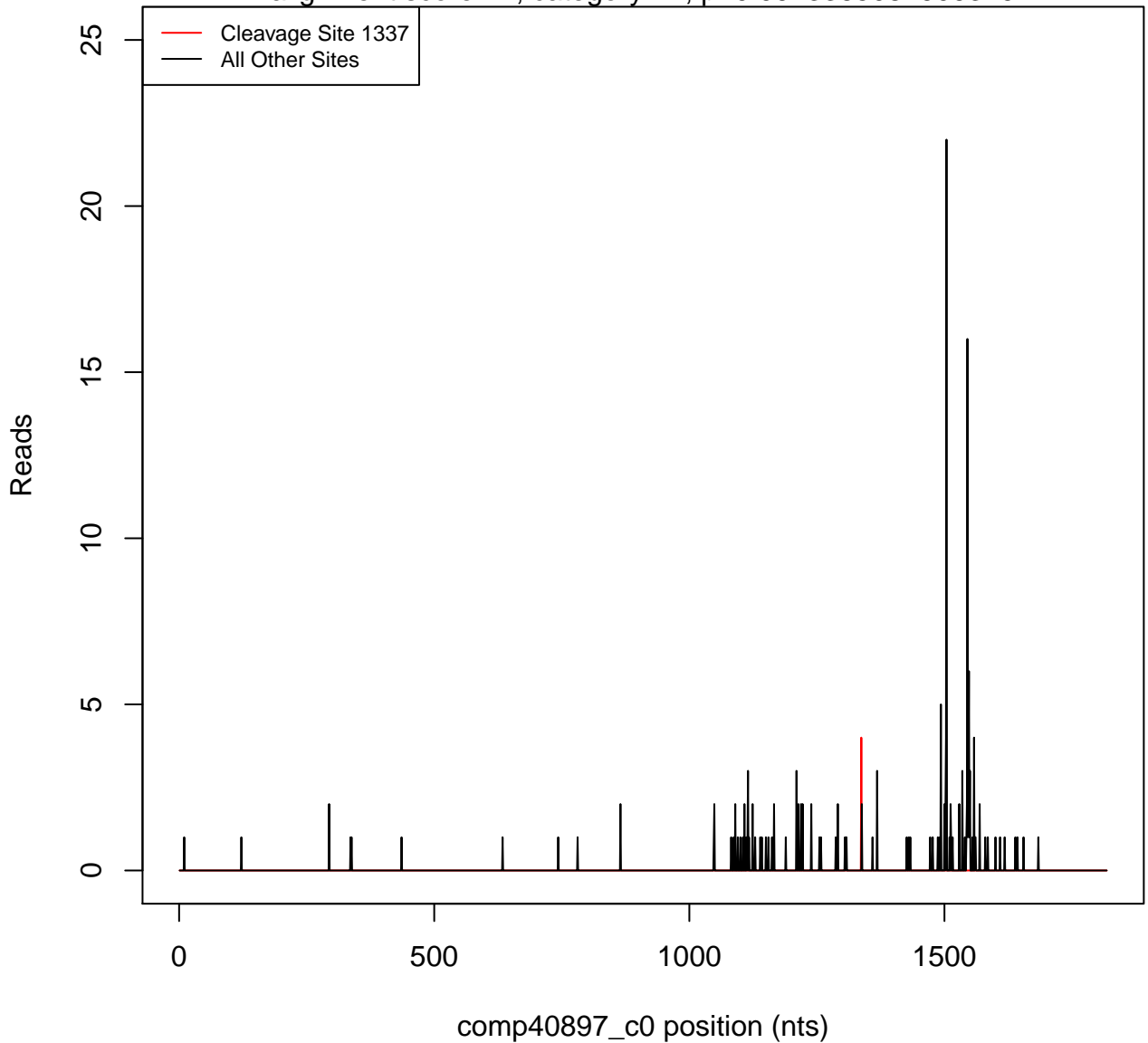

Supplement: S8 File — (ZIP) [file pone.0186500.s014.zip › S8 t-plot of miRNA-target/9comp40897_c0--1337--ath-miR394a_degradome.pdf]

# ath-miR159a\_R-1\_1ss6AG slicing comp10647\_c0 at nt 108

alignment score=3.5 , category=2 , p=0.0572383778530186

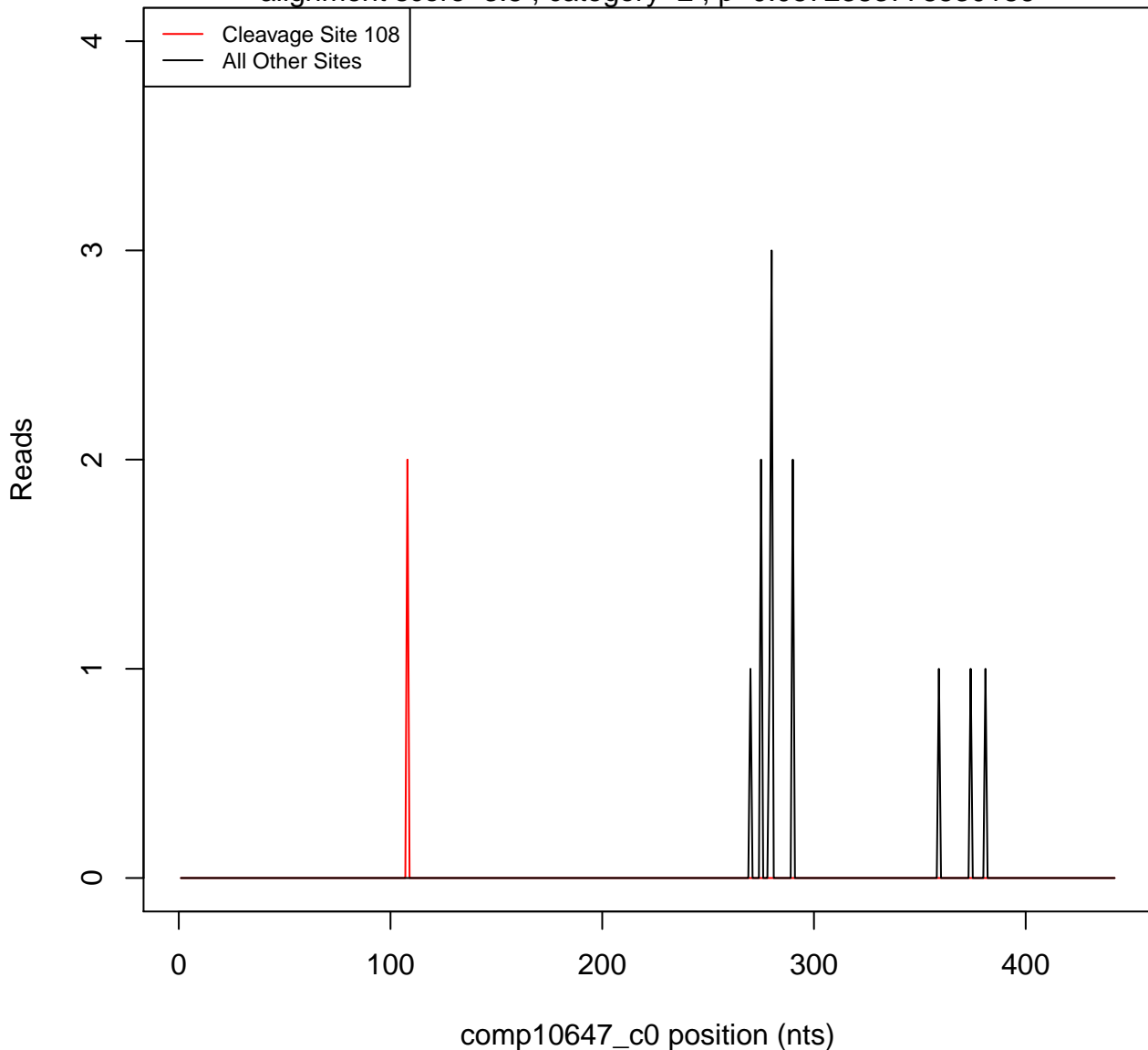

Supplement: S8 File — (ZIP) [file pone.0186500.s014.zip › S8 t-plot of miRNA-target/comp10647_c0--108--ath-miR159a_R-1_1ss6AG_degradome.pdf]

# nta-miR396b\_2ss20TA21TA slicing comp1228726\_c0 at nt 56

alignment score=4 , category=0 , p=0.0114966040365749

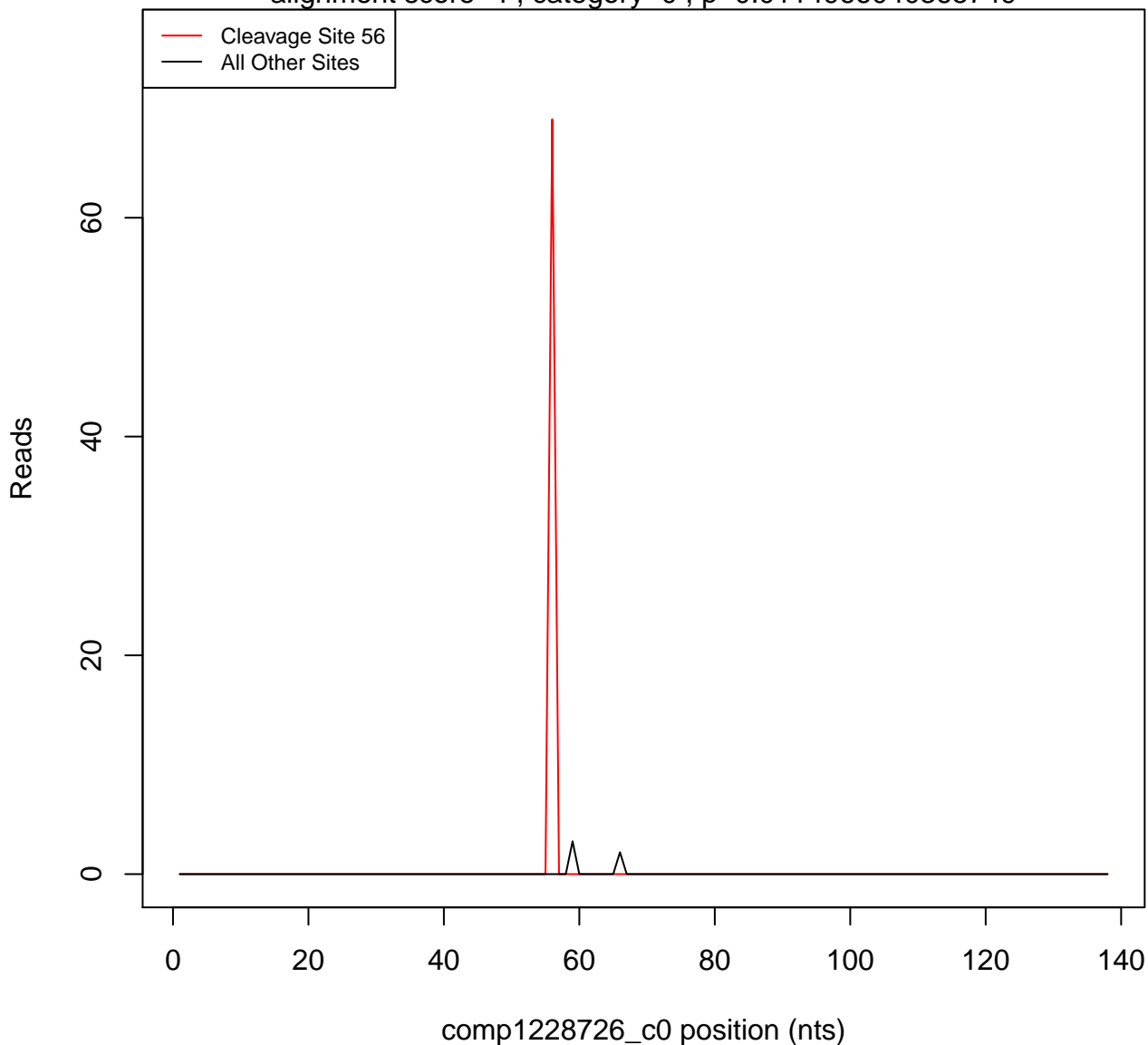

Supplement: S8 File — (ZIP) [file pone.0186500.s014.zip › S8 t-plot of miRNA-target/comp1228726_c0--56--nta-miR396b_2ss20TA21TA_degradome.pdf]

# nta-MIR6164b-p5\_1ss5CT slicing comp14512\_c0 at nt 12

alignment score=3.5 , category=0 , p=0.0324979322530617

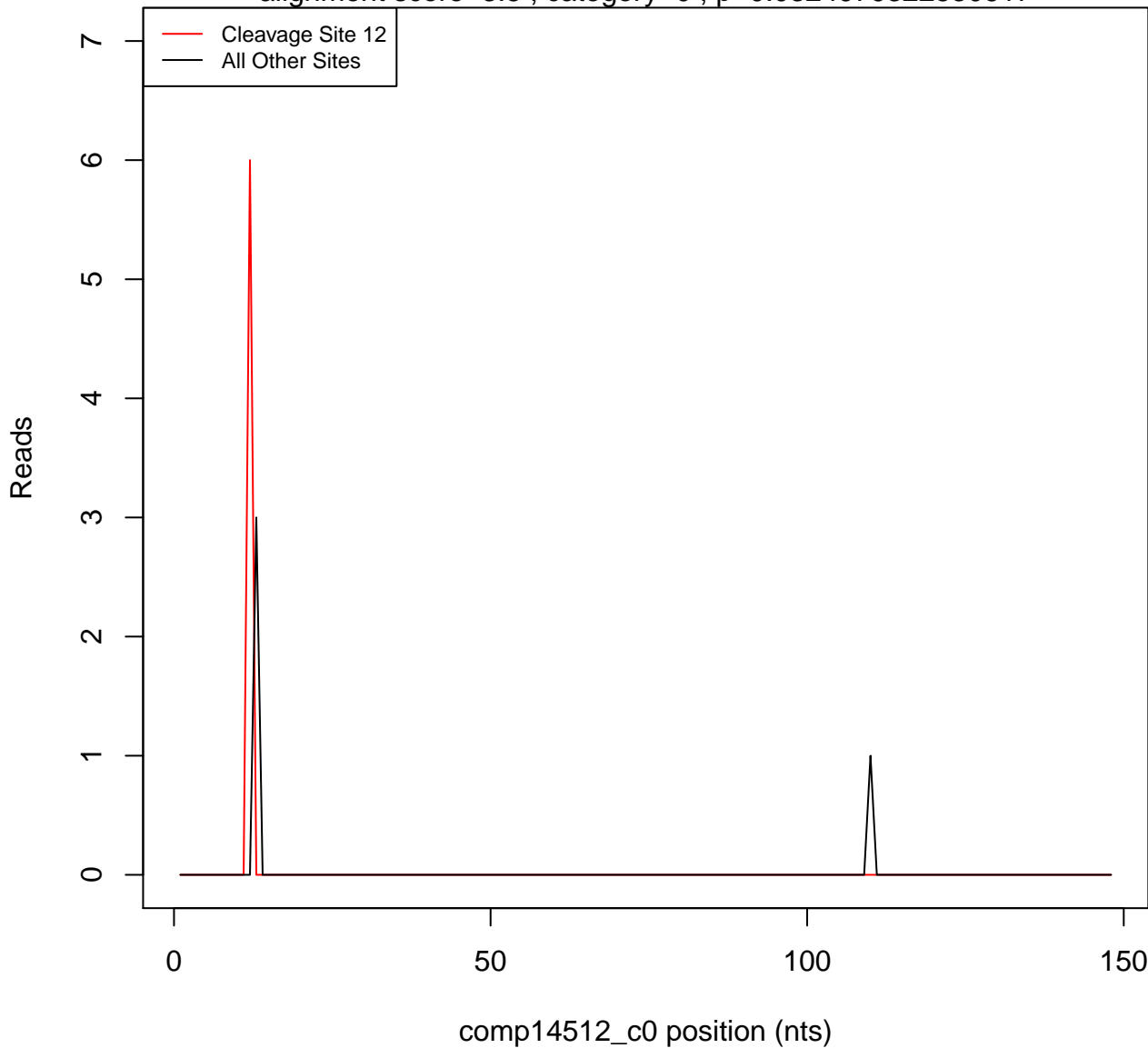

Supplement: S8 File — (ZIP) [file pone.0186500.s014.zip › S8 t-plot of miRNA-target/comp14512_c0--12--nta-MIR6164b-p5_1ss5CT_degradome.pdf]

# ath-miR396a slicing comp16637\_c0 at nt 111

alignment score=3 , category=0 , p=0.00494339851921732

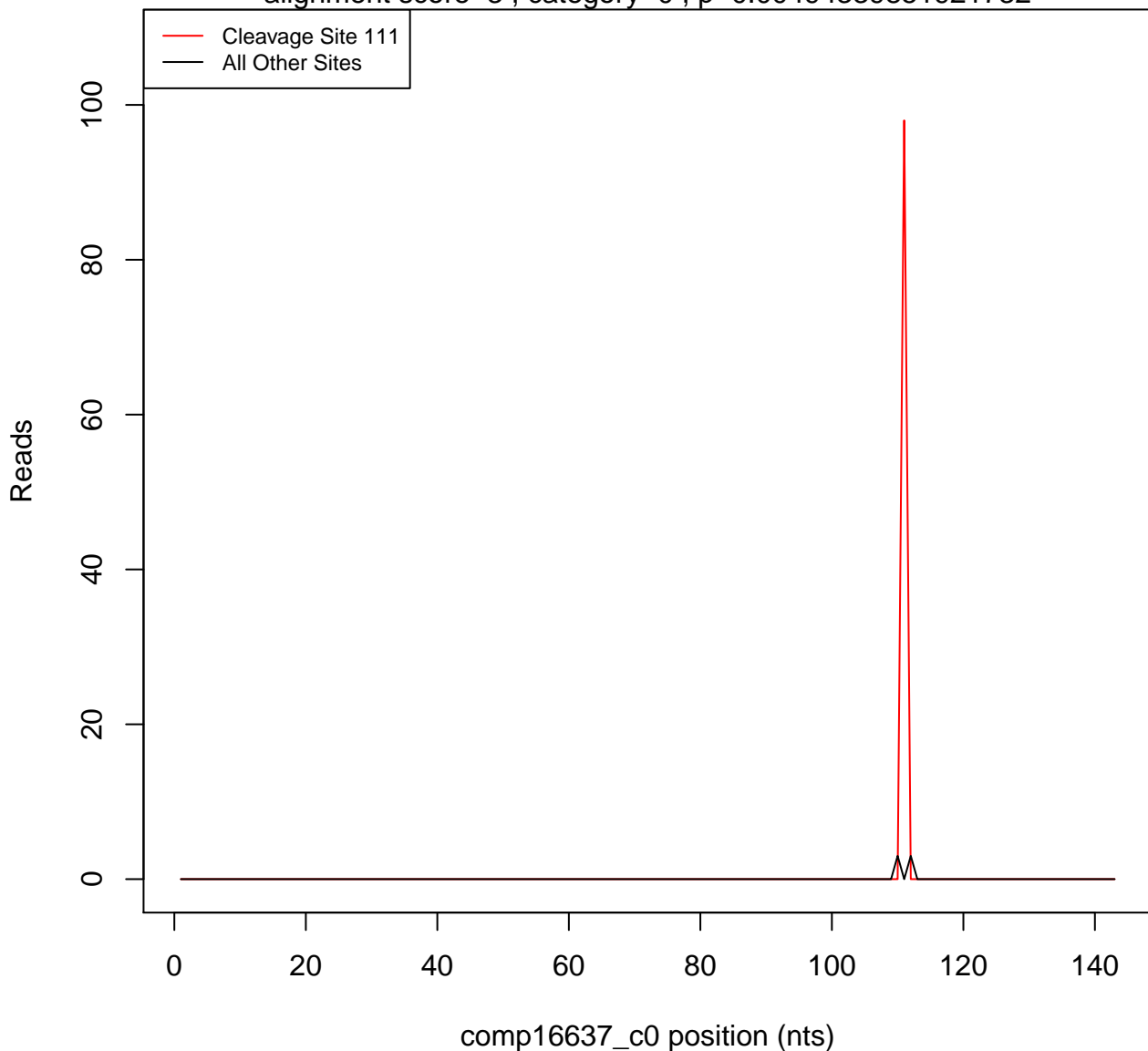

Supplement: S8 File — (ZIP) [file pone.0186500.s014.zip › S8 t-plot of miRNA-target/comp16637_c0--111--ath-miR396a_degradome.pdf]

# ath-miR396b slicing comp16637\_c0 at nt 111

alignment score=4 , category=0 , p=0.0190874837324402

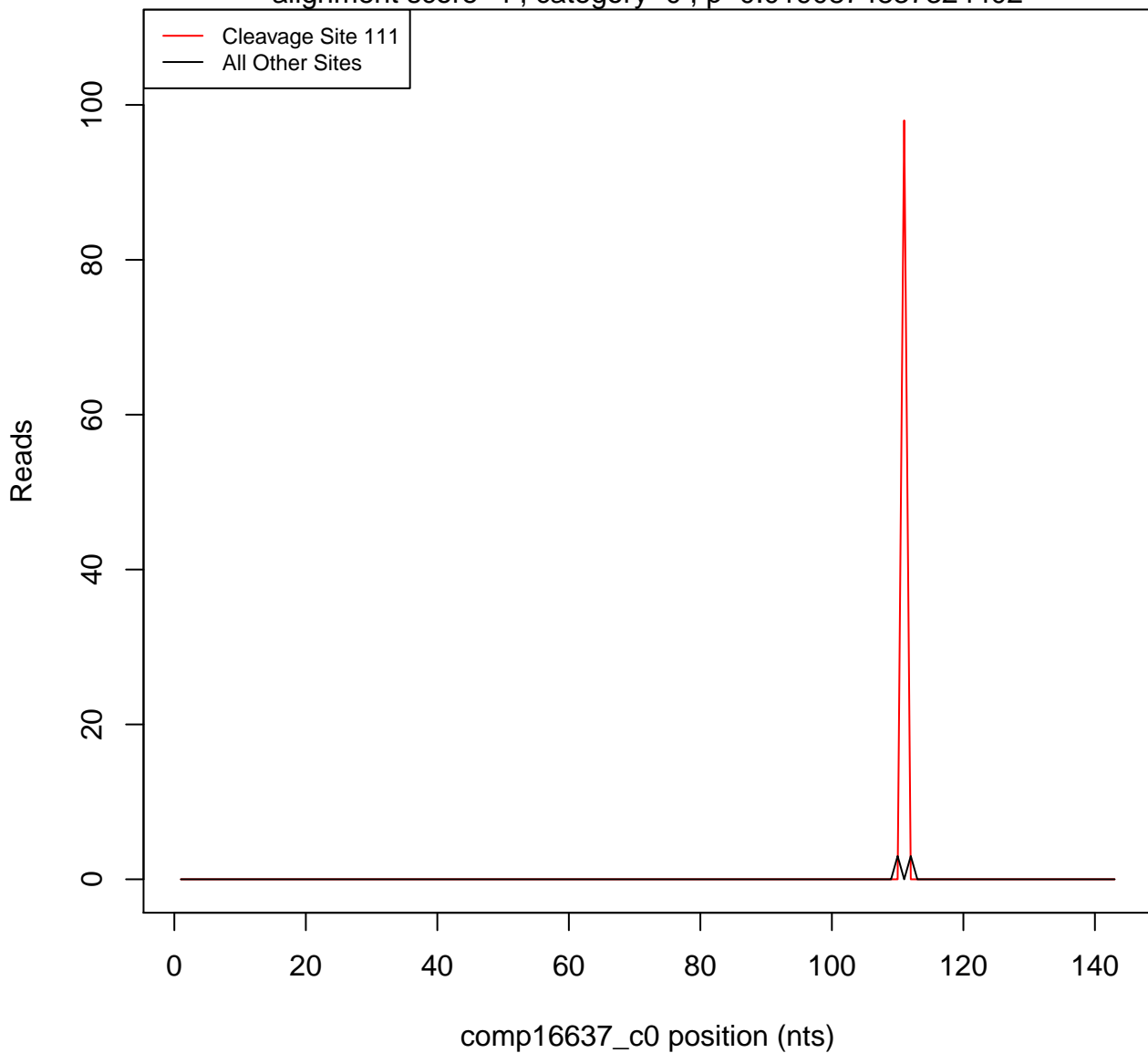

Supplement: S8 File — (ZIP) [file pone.0186500.s014.zip › S8 t-plot of miRNA-target/comp16637_c0--111--ath-miR396b_degradome.pdf]

# gma-miR396a-5p\_R+2 slicing comp16637\_c0 at nt 111

alignment score=4 , category=0 , p=0.00192534429318714

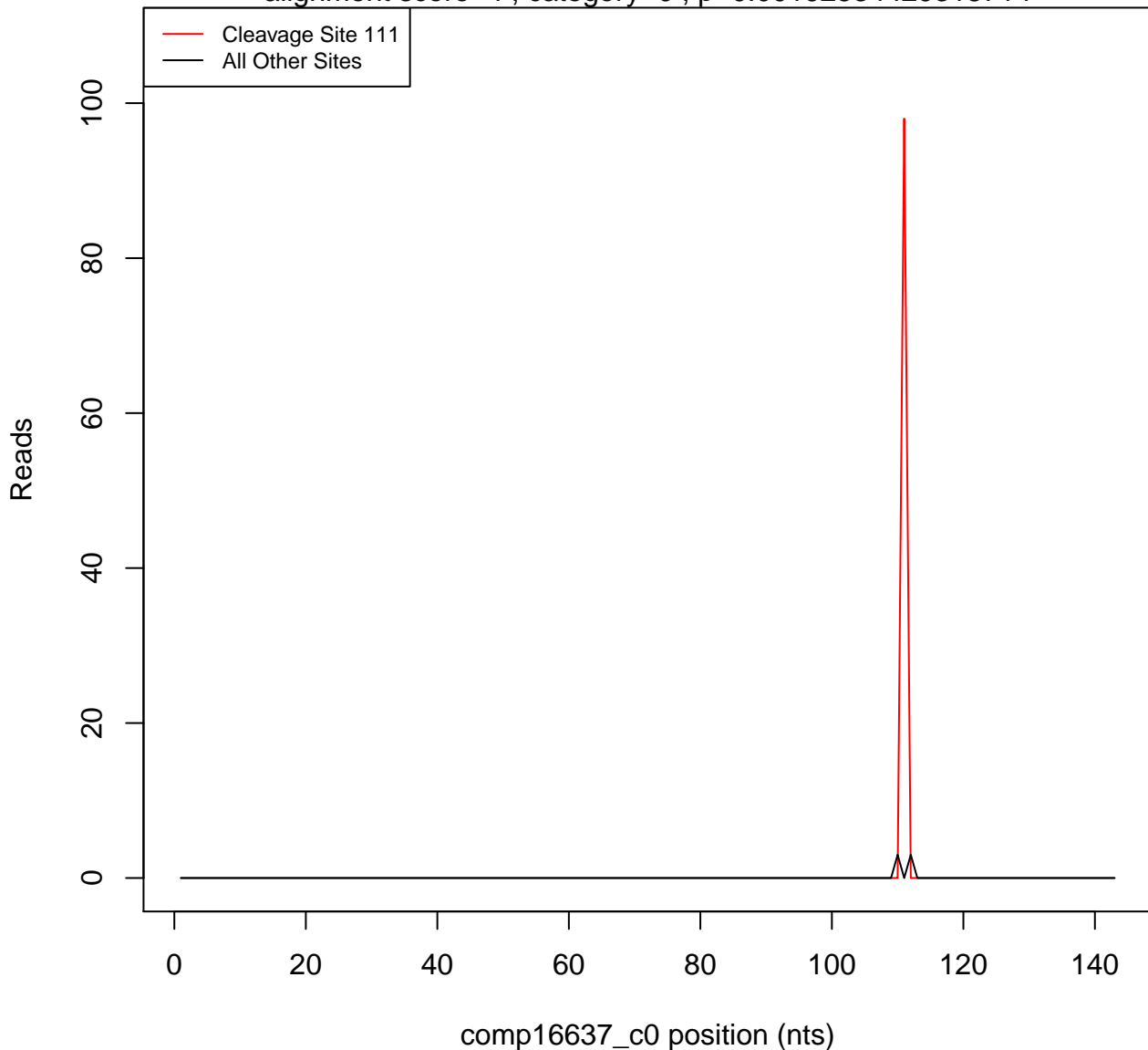

Supplement: S8 File — (ZIP) [file pone.0186500.s014.zip › S8 t-plot of miRNA-target/comp16637_c0--111--gma-miR396a-5p_R+2_degradome.pdf]

**nta-miR396b\_2ss20TA21TA slicing comp16637\_c0 at nt 111**

alignment score=4 , category=0 , p=0.0114966040365749

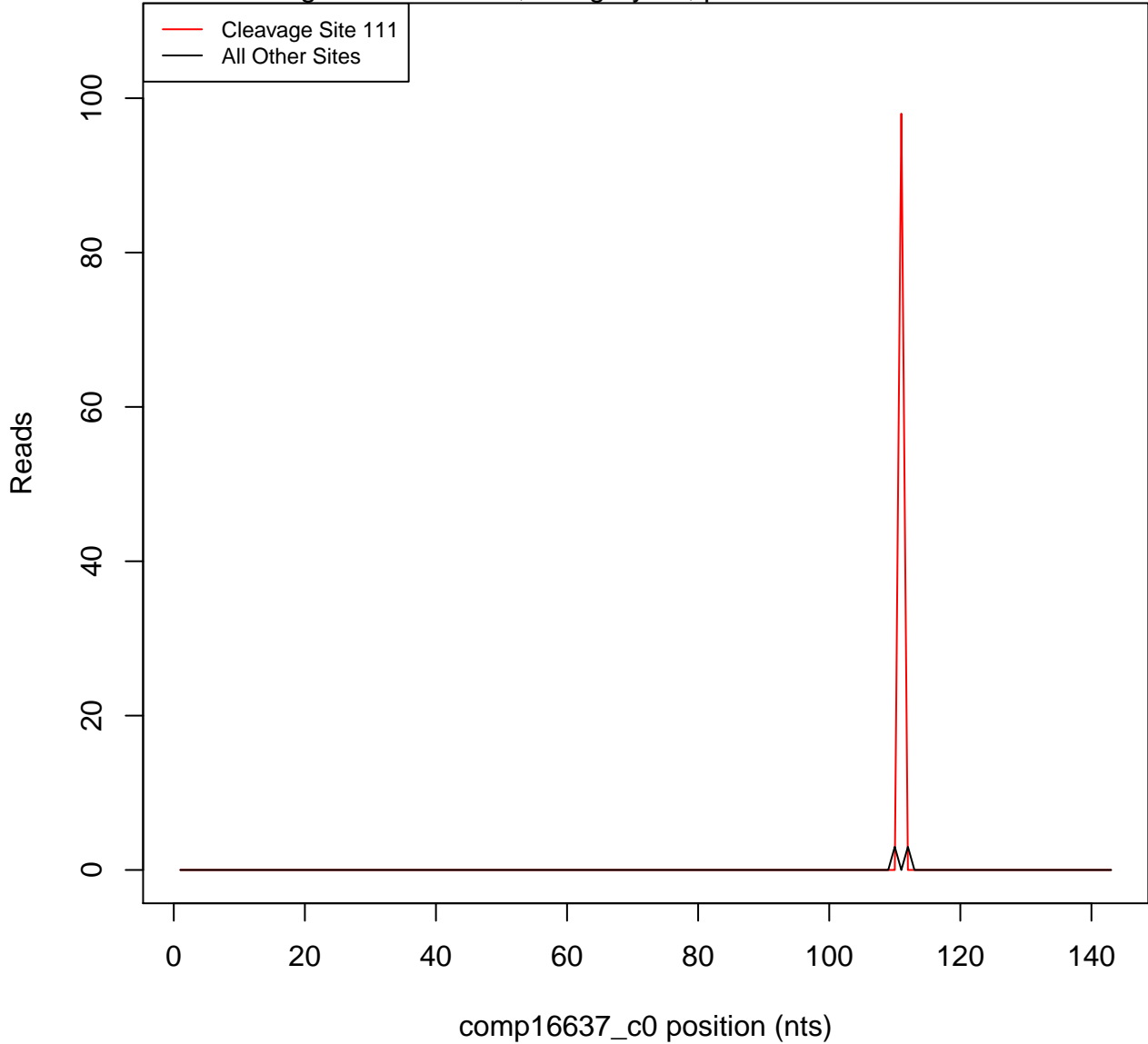

Supplement: S8 File — (ZIP) [file pone.0186500.s014.zip › S8 t-plot of miRNA-target/comp16637_c0--111--nta-miR396b_2ss20TA21TA_degradome.pdf]

# ath-miR172a\_1ss1AC slicing comp24578\_c0 at nt 91

alignment score=3 , category=0 , p=0.00494339851921732

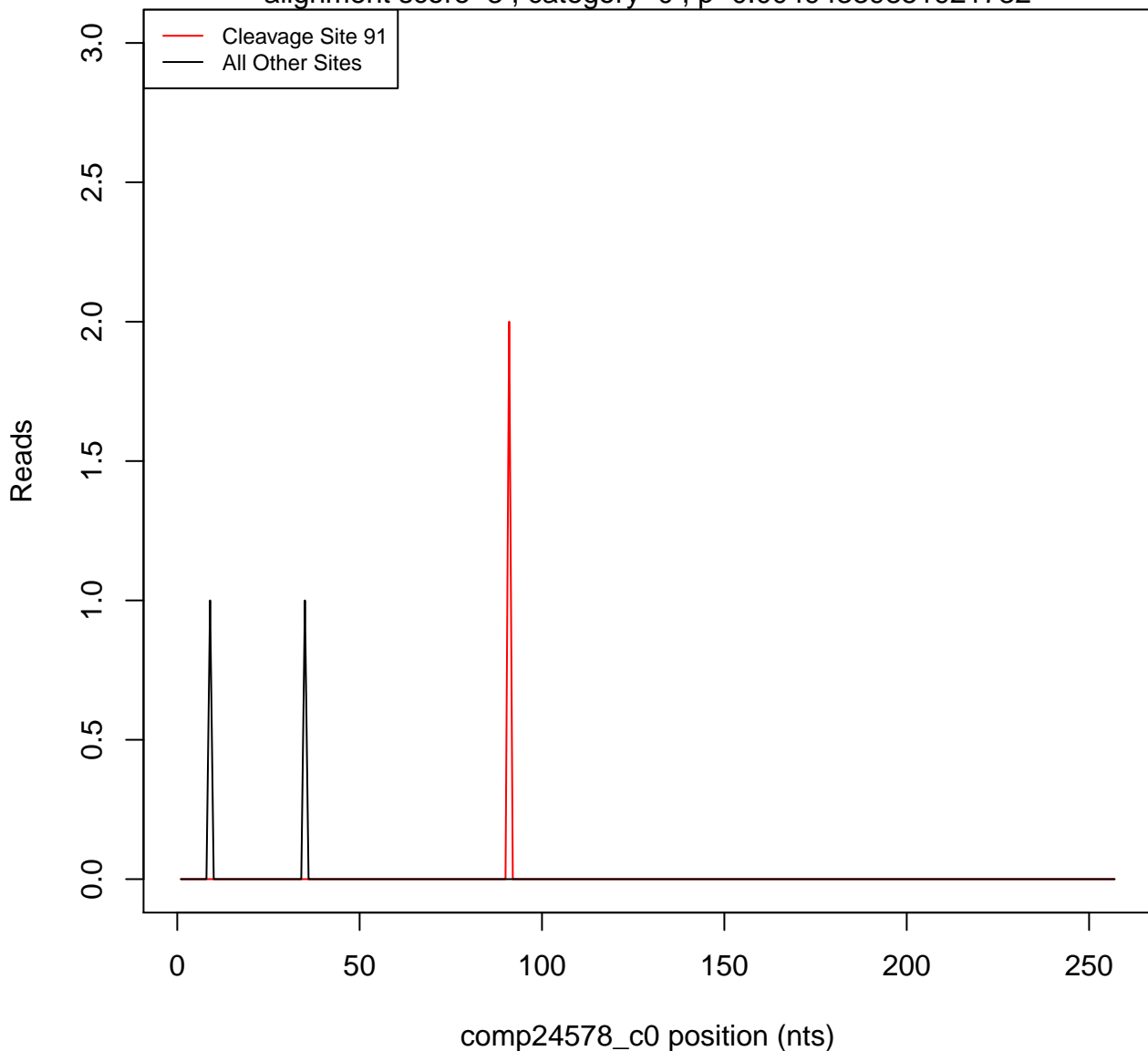

Supplement: S8 File — (ZIP) [file pone.0186500.s014.zip › S8 t-plot of miRNA-target/comp24578_c0--91--ath-miR172a_1ss1AC_degradome.pdf]

# ath-miR172c\_R+1 slicing comp24578\_c0 at nt 91

alignment score=2 , category=0 , p=0.00302387622887079

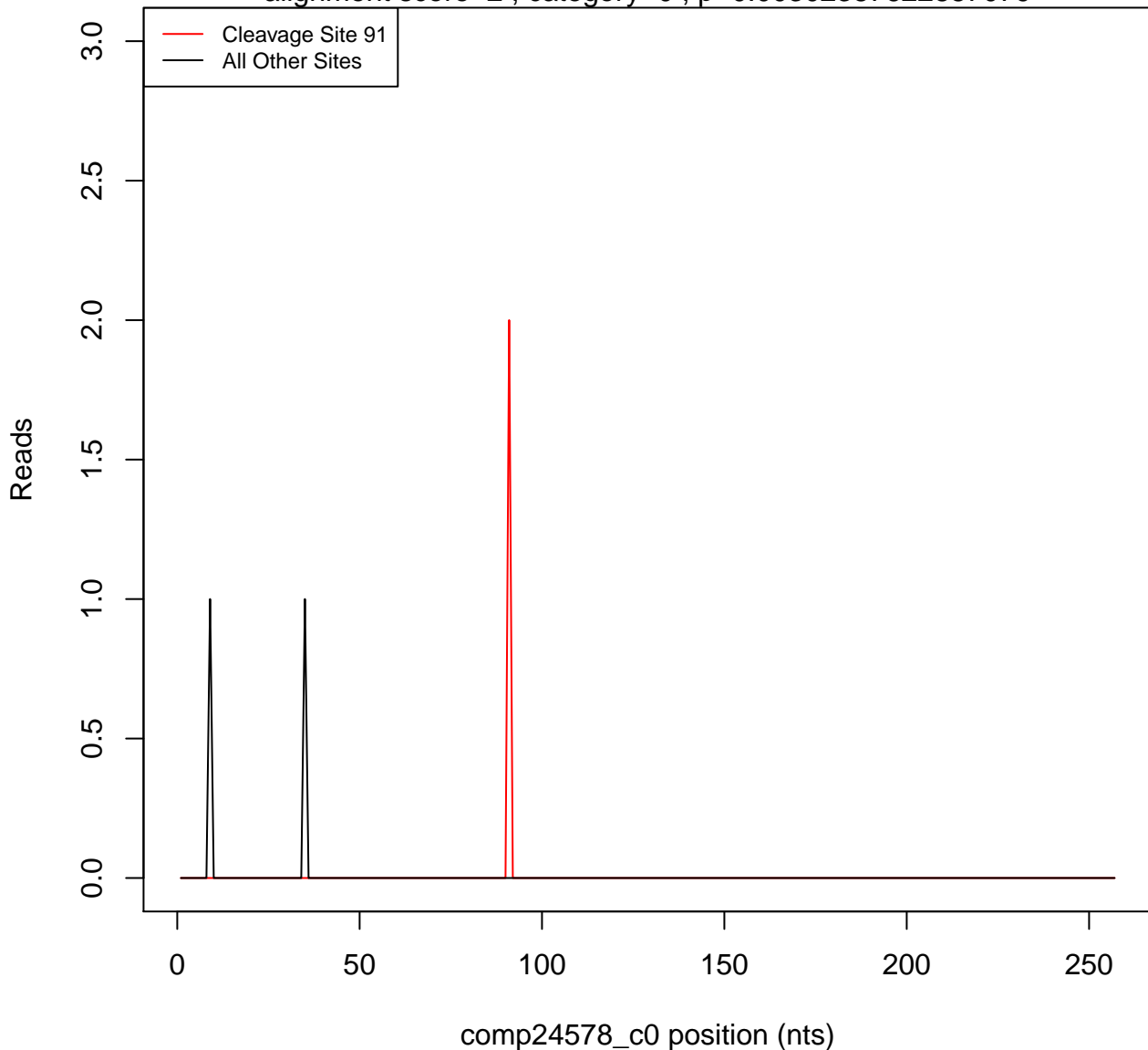

Supplement: S8 File — (ZIP) [file pone.0186500.s014.zip › S8 t-plot of miRNA-target/comp24578_c0--91--ath-miR172c_R+1_degradome.pdf]

# ath-miR396a slicing comp257104\_c0 at nt 221

alignment score=3.5 , category=0 , p=0.00658576156130786

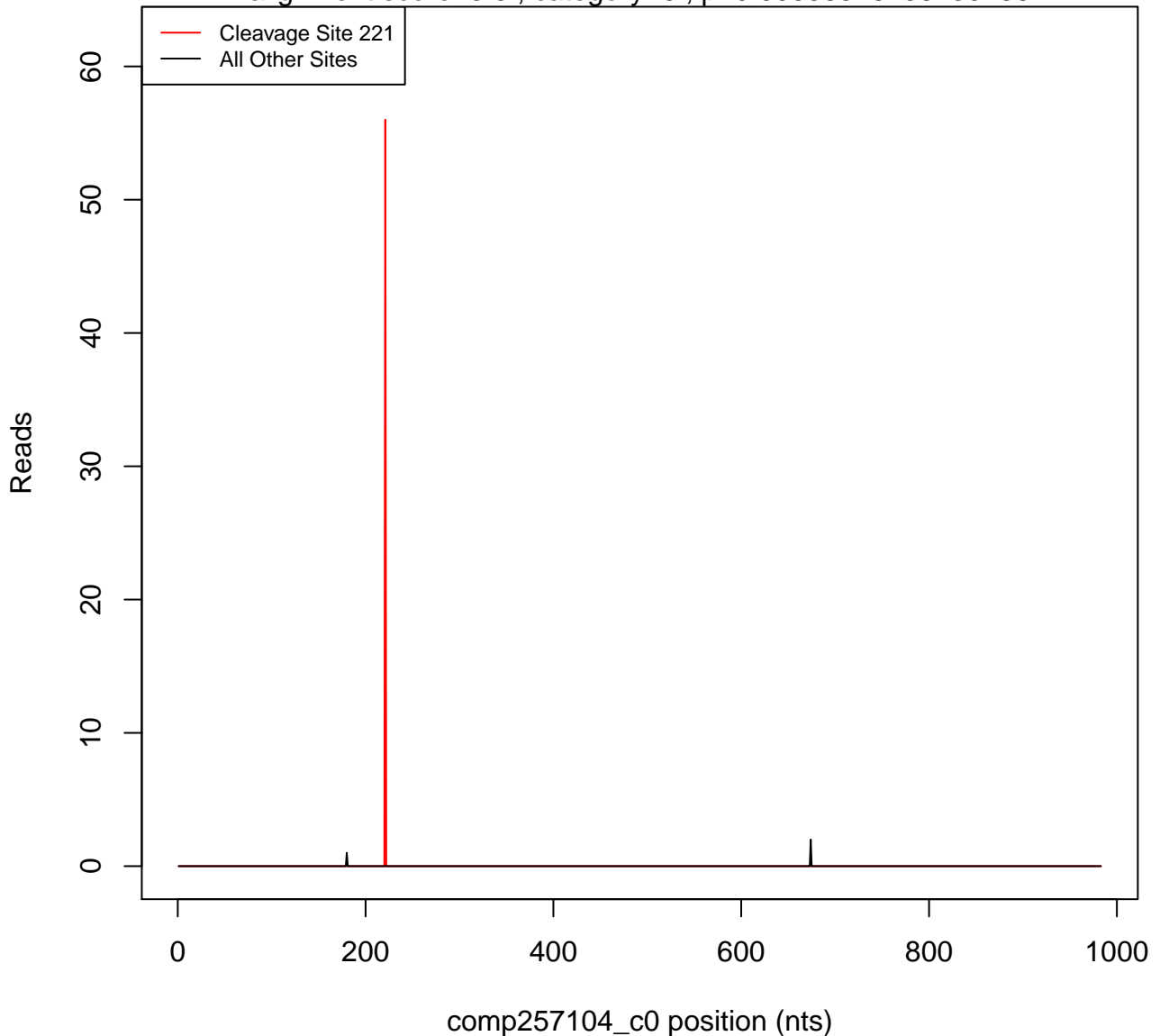

Supplement: S8 File — (ZIP) [file pone.0186500.s014.zip › S8 t-plot of miRNA-target/comp257104_c0--221--ath-miR396a_degradome.pdf]

# ath-miR396b\_1ss2TC slicing comp257104\_c0 at nt 221

alignment score=3 , category=0 , p=0.00247476148180459

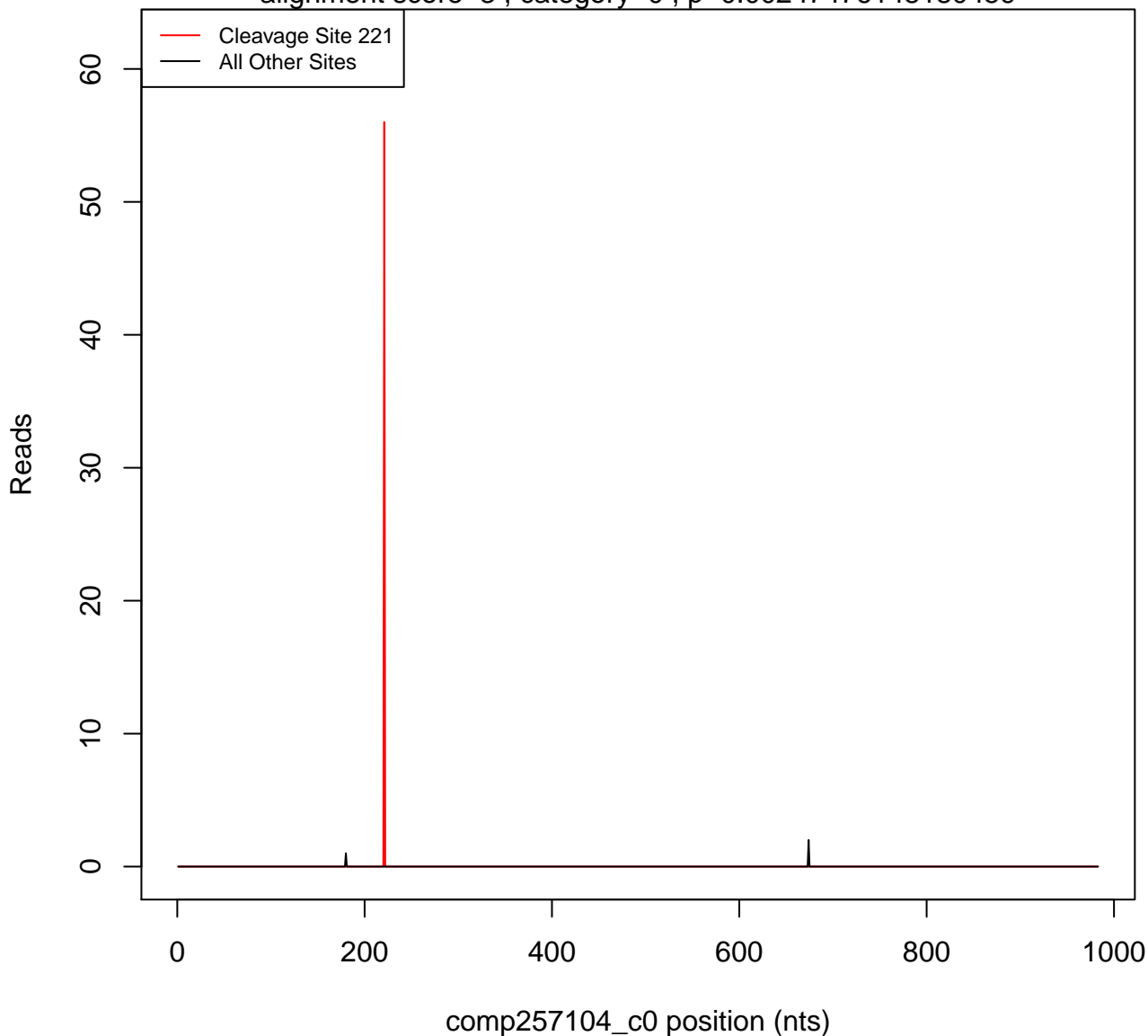

Supplement: S8 File — (ZIP) [file pone.0186500.s014.zip › S8 t-plot of miRNA-target/comp257104_c0--221--ath-miR396b_1ss2TC_degradome.pdf]

# ath-miR396b slicing comp257104\_c0 at nt 221

alignment score=4 , category=0 , p=0.0190874837324402

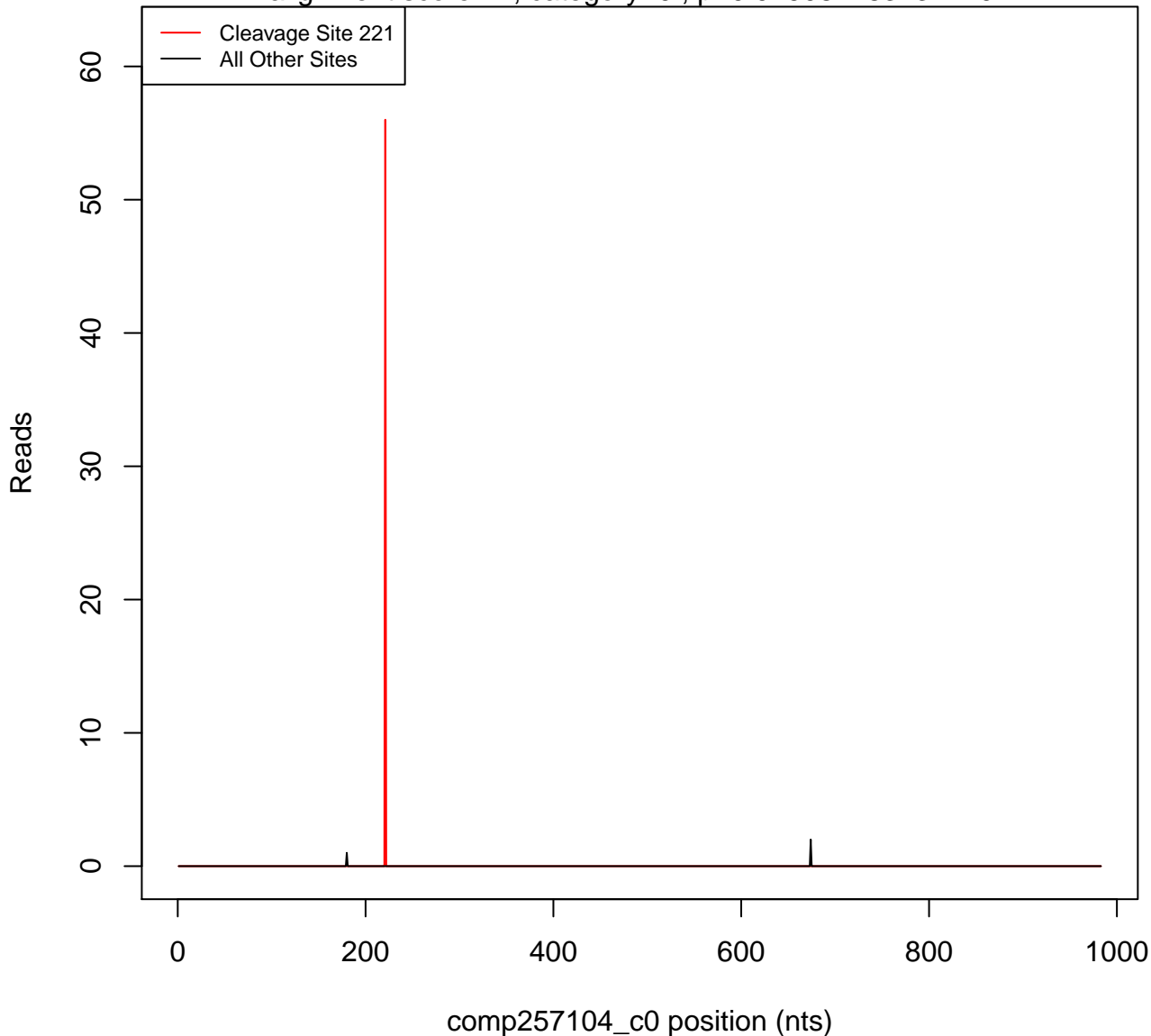

Supplement: S8 File — (ZIP) [file pone.0186500.s014.zip › S8 t-plot of miRNA-target/comp257104_c0--221--ath-miR396b_degradome.pdf]

# ath-miR159a\_R-1\_1ss1TG slicing comp26487\_c0 at nt 594

alignment score=3 , category=0 , p=0.00740592626877734

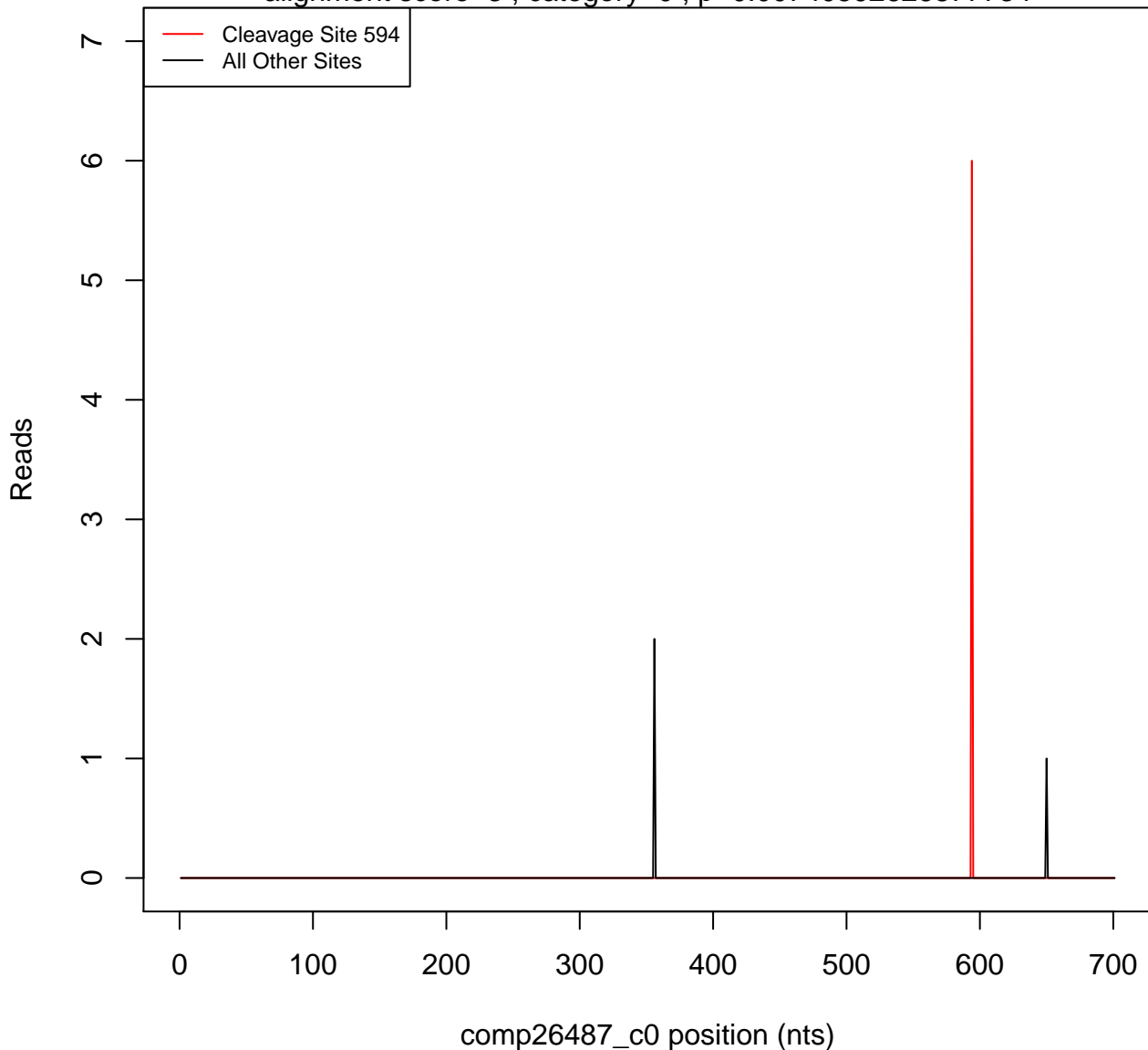

Supplement: S8 File — (ZIP) [file pone.0186500.s014.zip › S8 t-plot of miRNA-target/comp26487_c0--594--ath-miR159a_R-1_1ss1TG_degradome.pdf]

# ath-miR159a\_R-1\_1ss20TA slicing comp26487\_c0 at nt 594

alignment score=4 , category=0 , p=0.0247423650964395

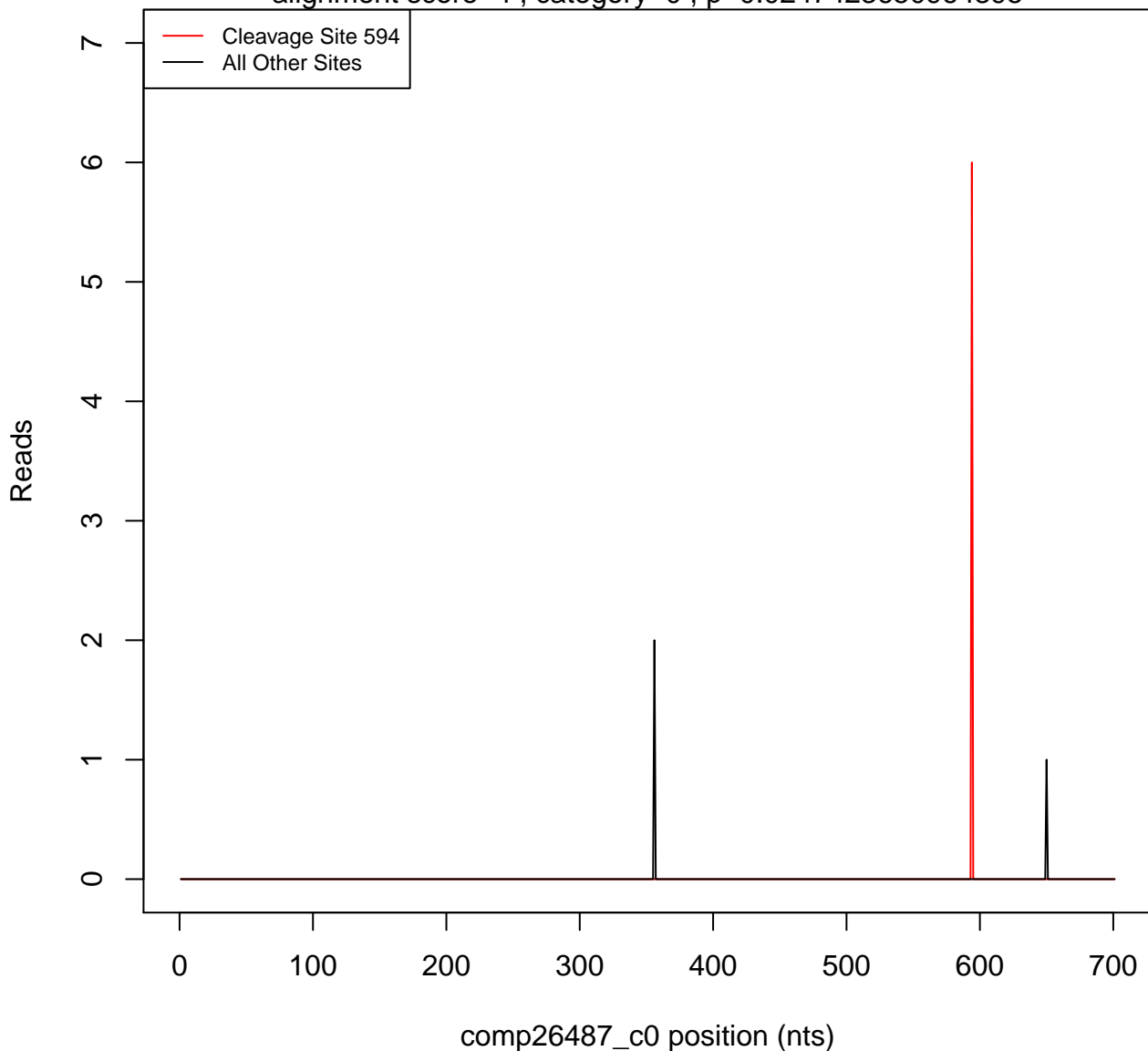

Supplement: S8 File — (ZIP) [file pone.0186500.s014.zip › S8 t-plot of miRNA-target/comp26487_c0--594--ath-miR159a_R-1_1ss20TA_degradome.pdf]

# ath-miR159a\_R-1\_1ss20TC slicing comp26487\_c0 at nt 594

alignment score=3 , category=0 , p=0.00740592626877734

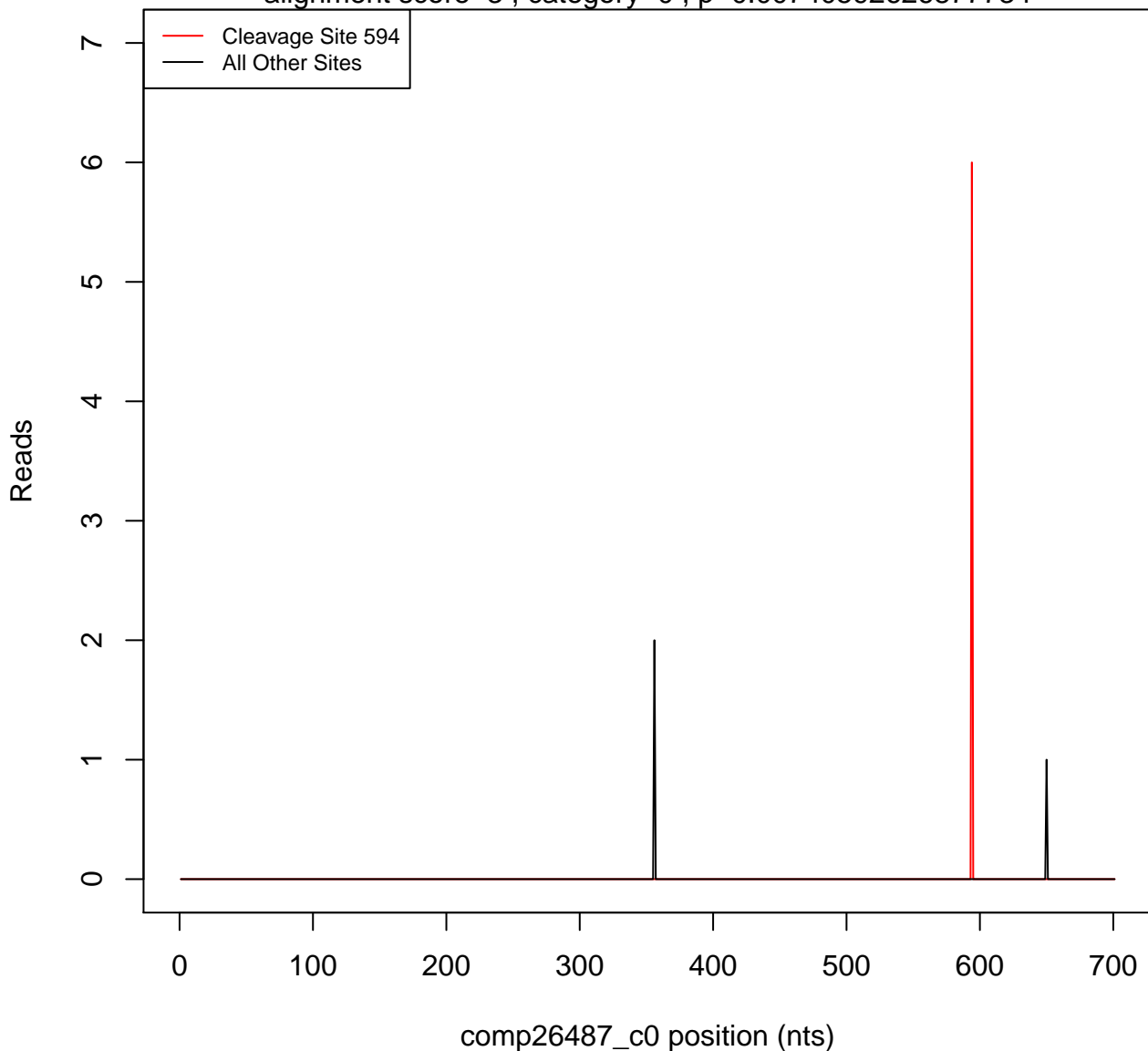

Supplement: S8 File — (ZIP) [file pone.0186500.s014.zip › S8 t-plot of miRNA-target/comp26487_c0--594--ath-miR159a_R-1_1ss20TC_degradome.pdf]

# ath-miR159a\_R-2\_1ss19CT slicing comp26487\_c0 at nt 594

alignment score=3.5 , category=0 , p=0.0303646465264888

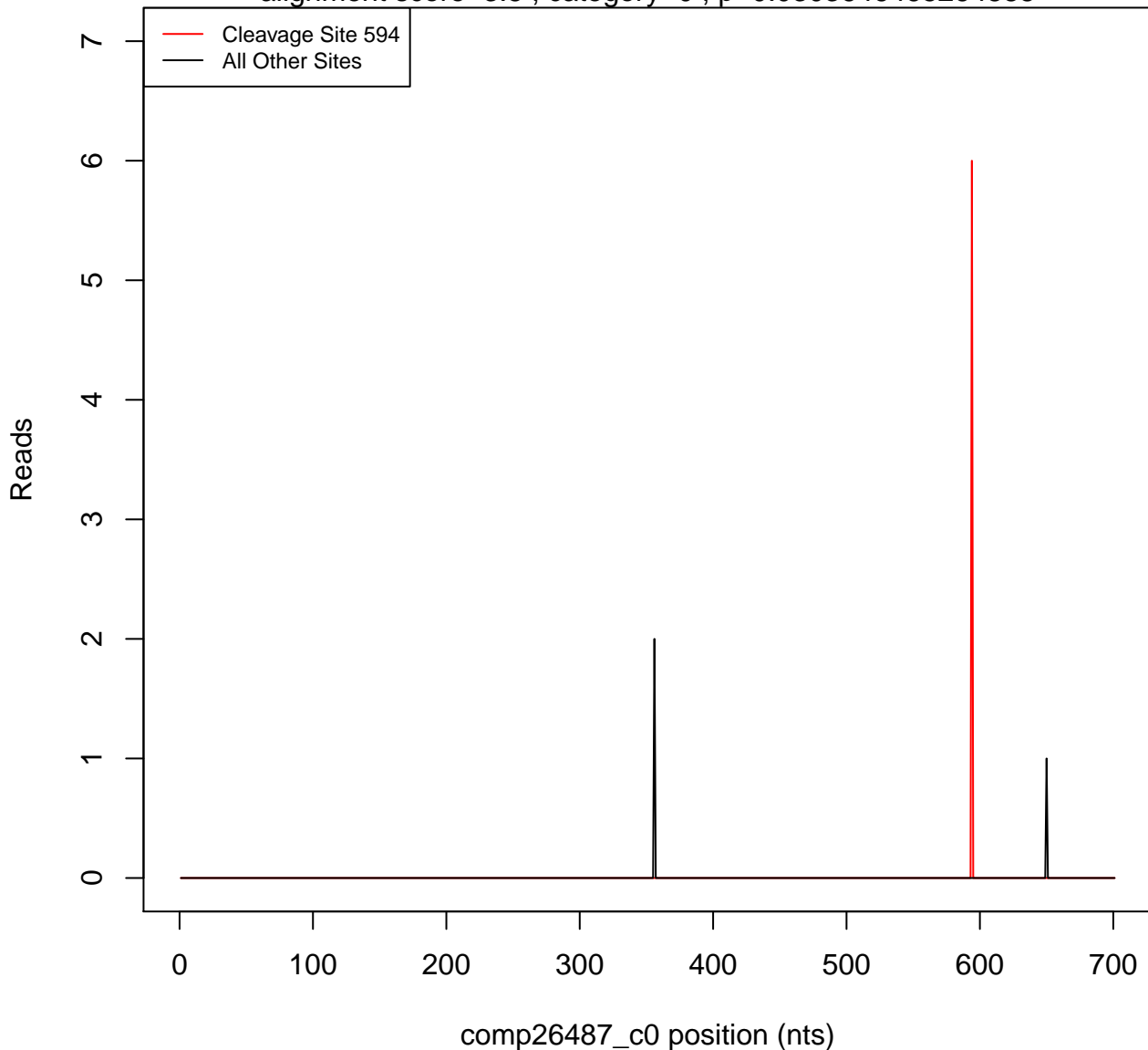

Supplement: S8 File — (ZIP) [file pone.0186500.s014.zip › S8 t-plot of miRNA-target/comp26487_c0--594--ath-miR159a_R-2_1ss19CT_degradome.pdf]

# ath-miR159a\_R-3 slicing comp26487\_c0 at nt 594

alignment score=3 , category=0 , p=0.0196274535572283

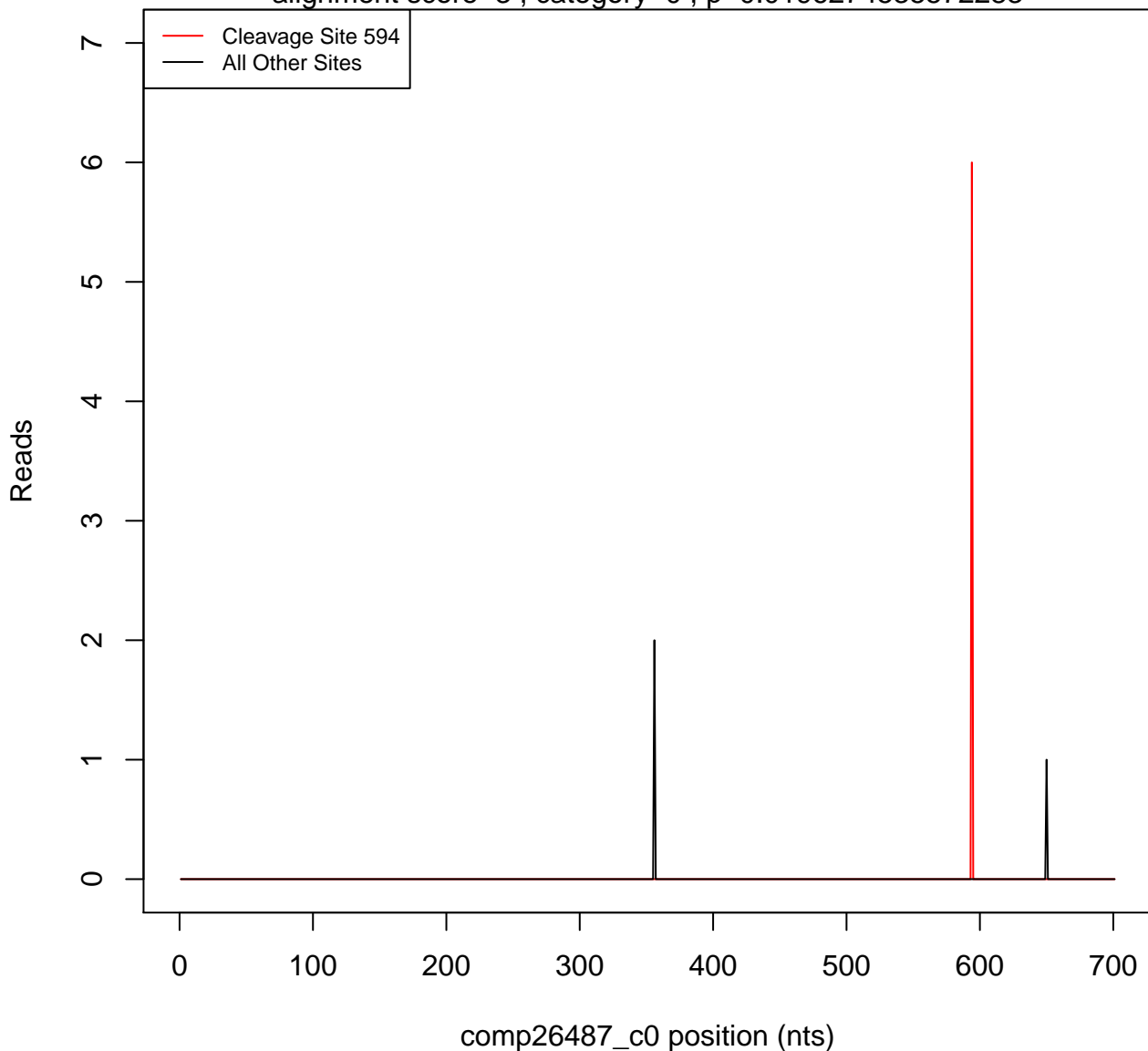

Supplement: S8 File — (ZIP) [file pone.0186500.s014.zip › S8 t-plot of miRNA-target/comp26487_c0--594--ath-miR159a_R-3_degradome.pdf]

# ath-miR159a slicing comp26487\_c0 at nt 594

alignment score=3.5 , category=0 , p=0.00439534100709571

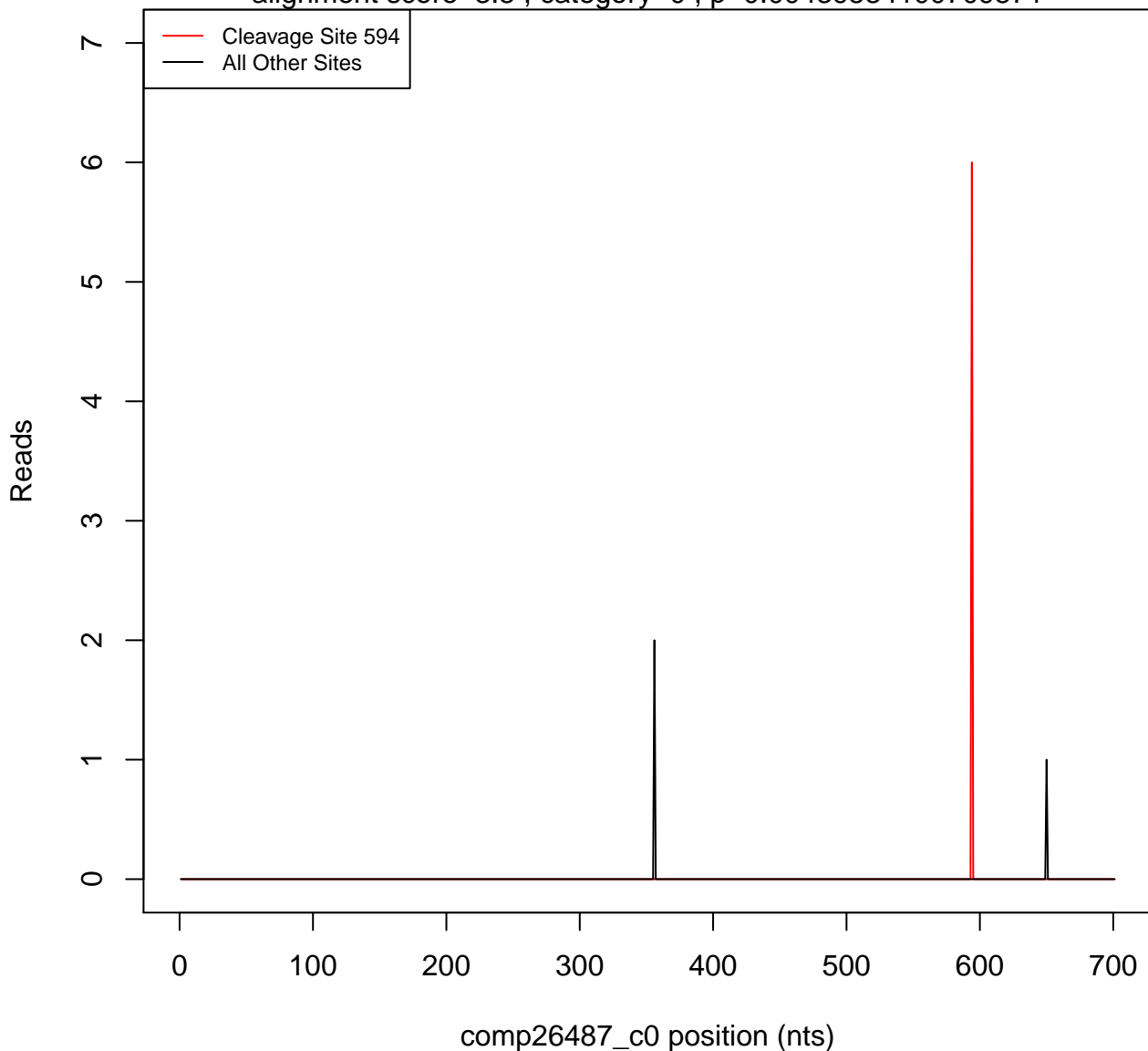

Supplement: S8 File — (ZIP) [file pone.0186500.s014.zip › S8 t-plot of miRNA-target/comp26487_c0--594--ath-miR159a_degradome.pdf]

# ath-miR408\_L-1R+1 slicing comp30637\_c0 at nt 84

alignment score=1 , category=0 , p=0.00357268870087279

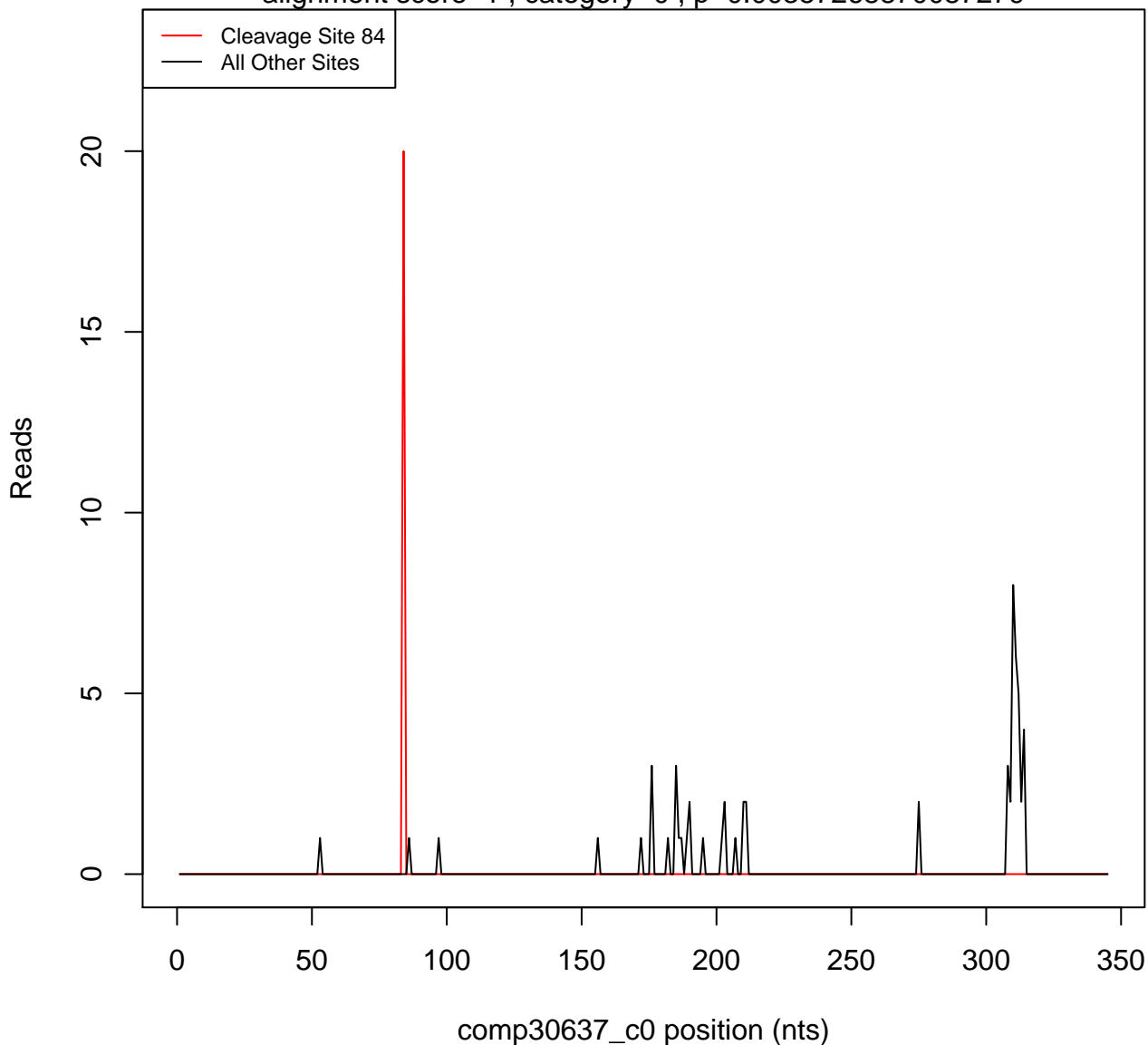

Supplement: S8 File — (ZIP) [file pone.0186500.s014.zip › S8 t-plot of miRNA-target/comp30637_c0--84--ath-miR408_L-1R+1_degradome.pdf]

# ath-miR157a\_R+1\_1ss21CT slicing comp31576\_c0 at nt 797

alignment score=3.5 , category=1 , p=0.00536413091816301

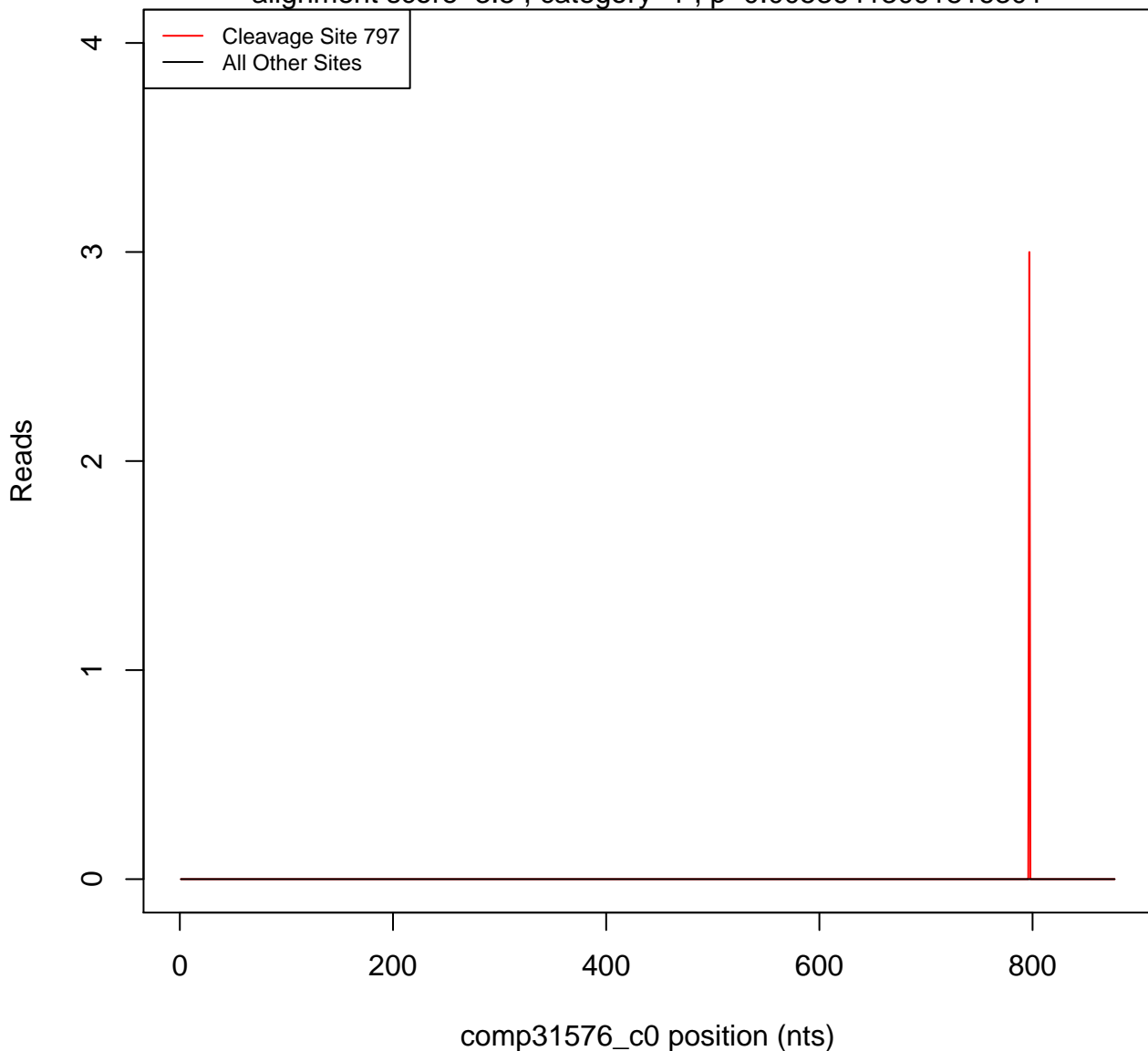

Supplement: S8 File — (ZIP) [file pone.0186500.s014.zip › S8 t-plot of miRNA-target/comp31576_c0--797--ath-miR157a_R+1_1ss21CT_degradome.pdf]

# ath-miR157d\_L+1R-1 slicing comp31576\_c0 at nt 797

alignment score=3 , category=1 , p=0.0209554359377904

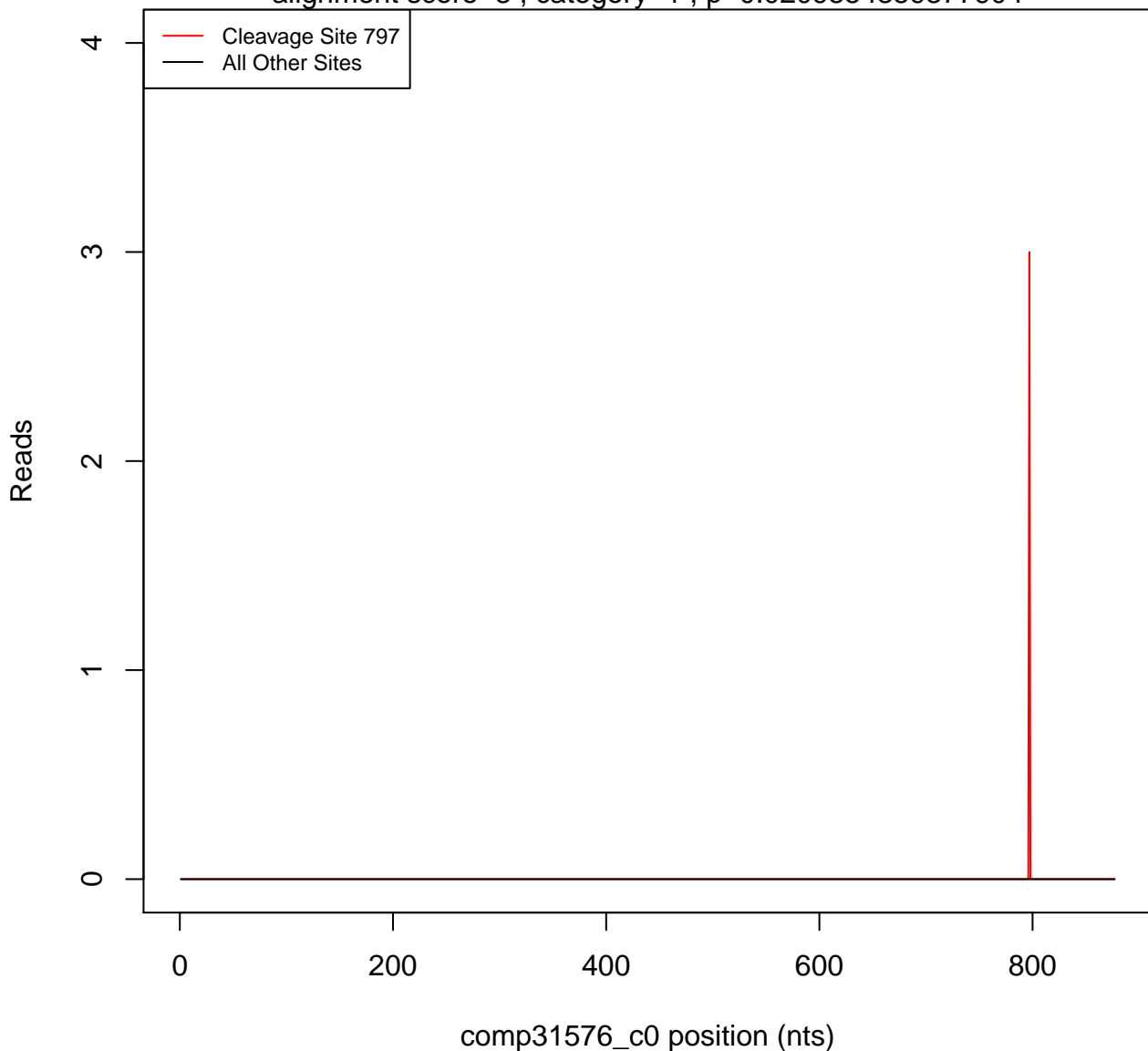

Supplement: S8 File — (ZIP) [file pone.0186500.s014.zip › S8 t-plot of miRNA-target/comp31576_c0--797--ath-miR157d_L+1R-1_degradome.pdf]

# ath-miR157d\_L+1 slicing comp31576\_c0 at nt 797

alignment score=2 , category=1 , p=0.00736825343301251

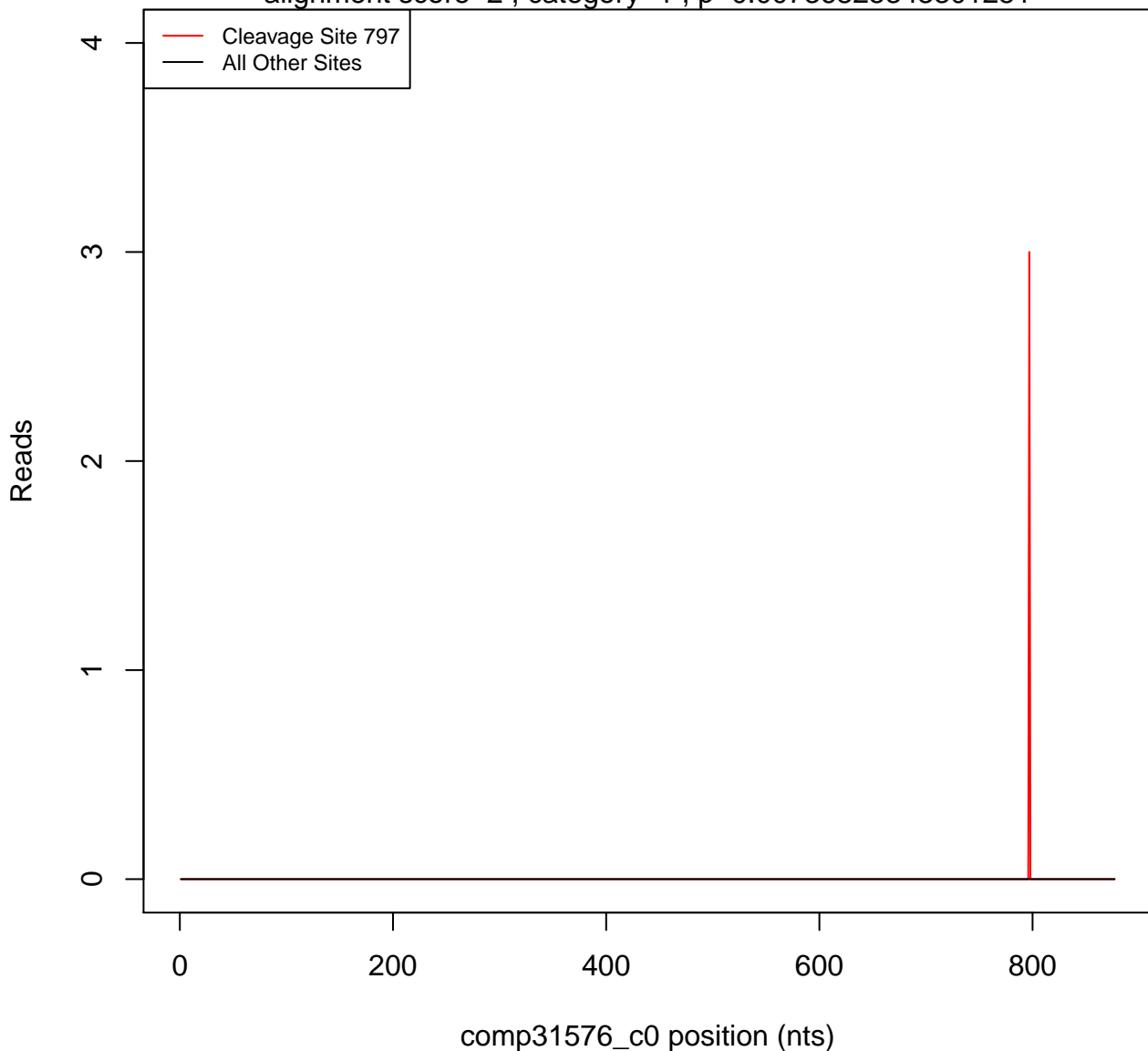

Supplement: S8 File — (ZIP) [file pone.0186500.s014.zip › S8 t-plot of miRNA-target/comp31576_c0--797--ath-miR157d_L+1_degradome.pdf]

# bcy-miR156\_L-1\_2ss13TG16AT slicing comp31576\_c0 at nt 797

alignment score=1 , category=1 , p=0.00870209118013676

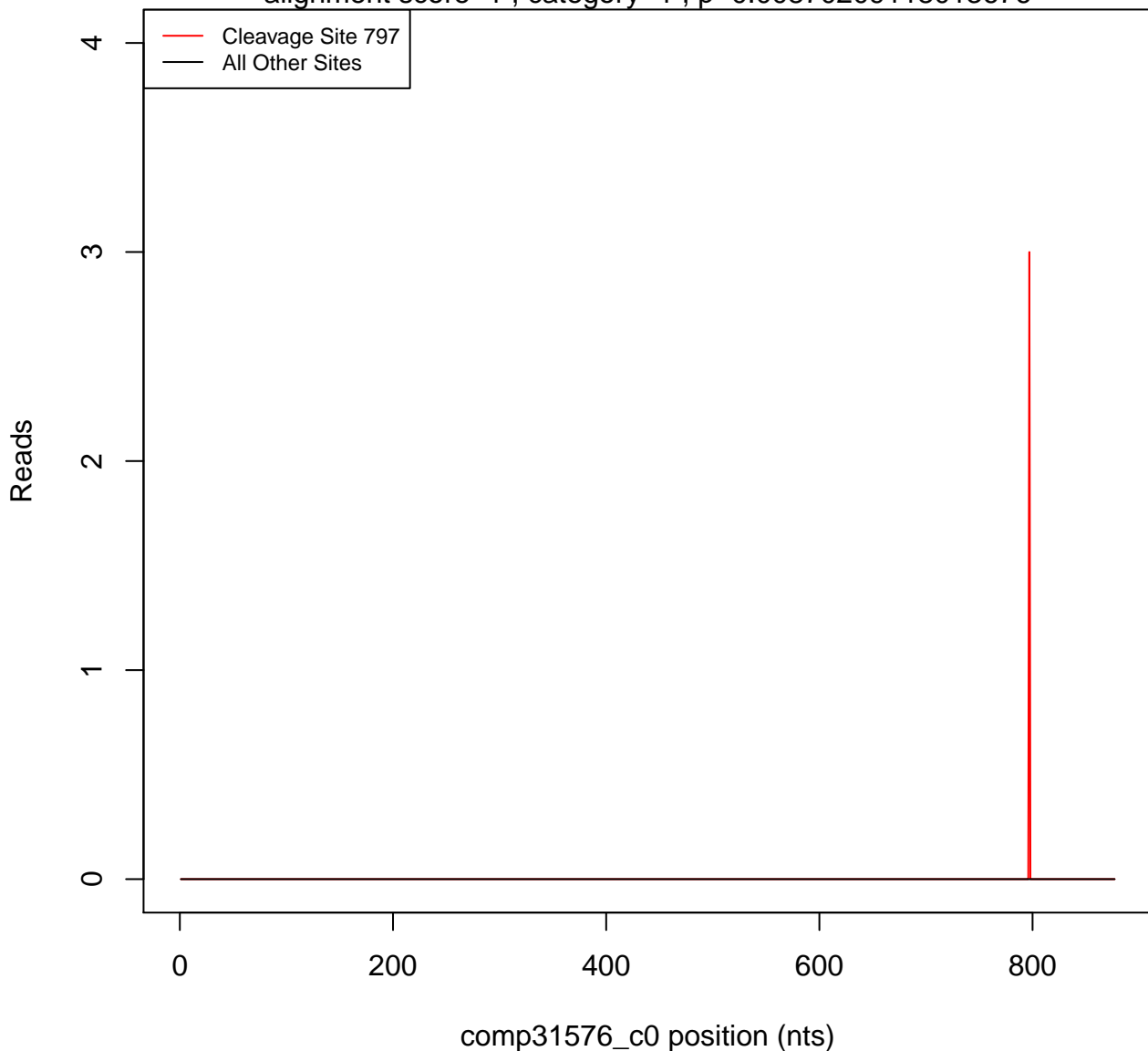

Supplement: S8 File — (ZIP) [file pone.0186500.s014.zip › S8 t-plot of miRNA-target/comp31576_c0--797--bcy-miR156_L-1_2ss13TG16AT_degradome.pdf]

# mdm-miR156t slicing comp31576\_c0 at nt 797

alignment score=0 , category=1 , p=0.00502971732708901

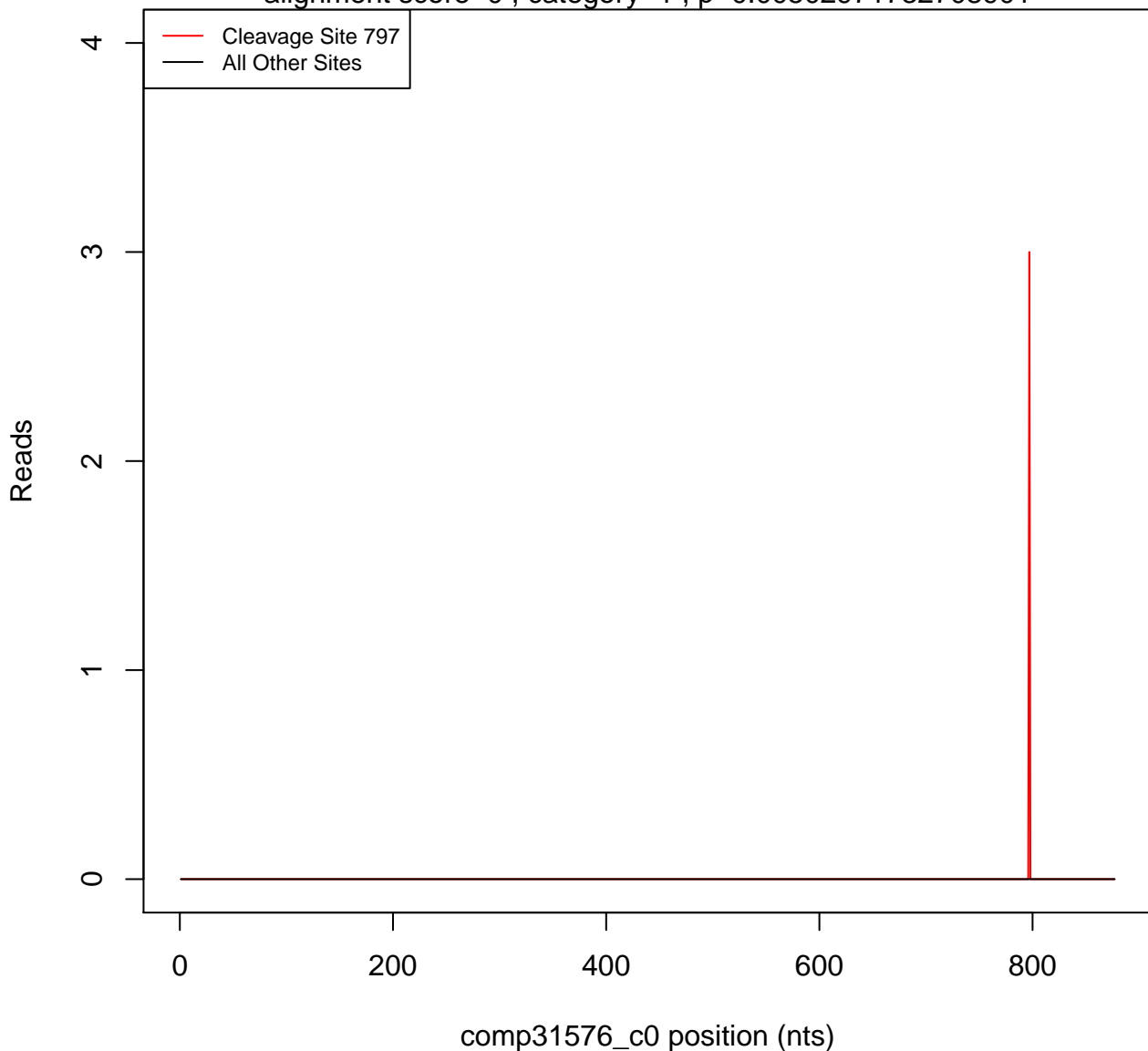

Supplement: S8 File — (ZIP) [file pone.0186500.s014.zip › S8 t-plot of miRNA-target/comp31576_c0--797--mdm-miR156t_degradome.pdf]

**mtr-miR156e\_1ss2TA slicing comp31576\_c0 at nt 797**

alignment score=4 , category=1 , p=0.00469519130044582

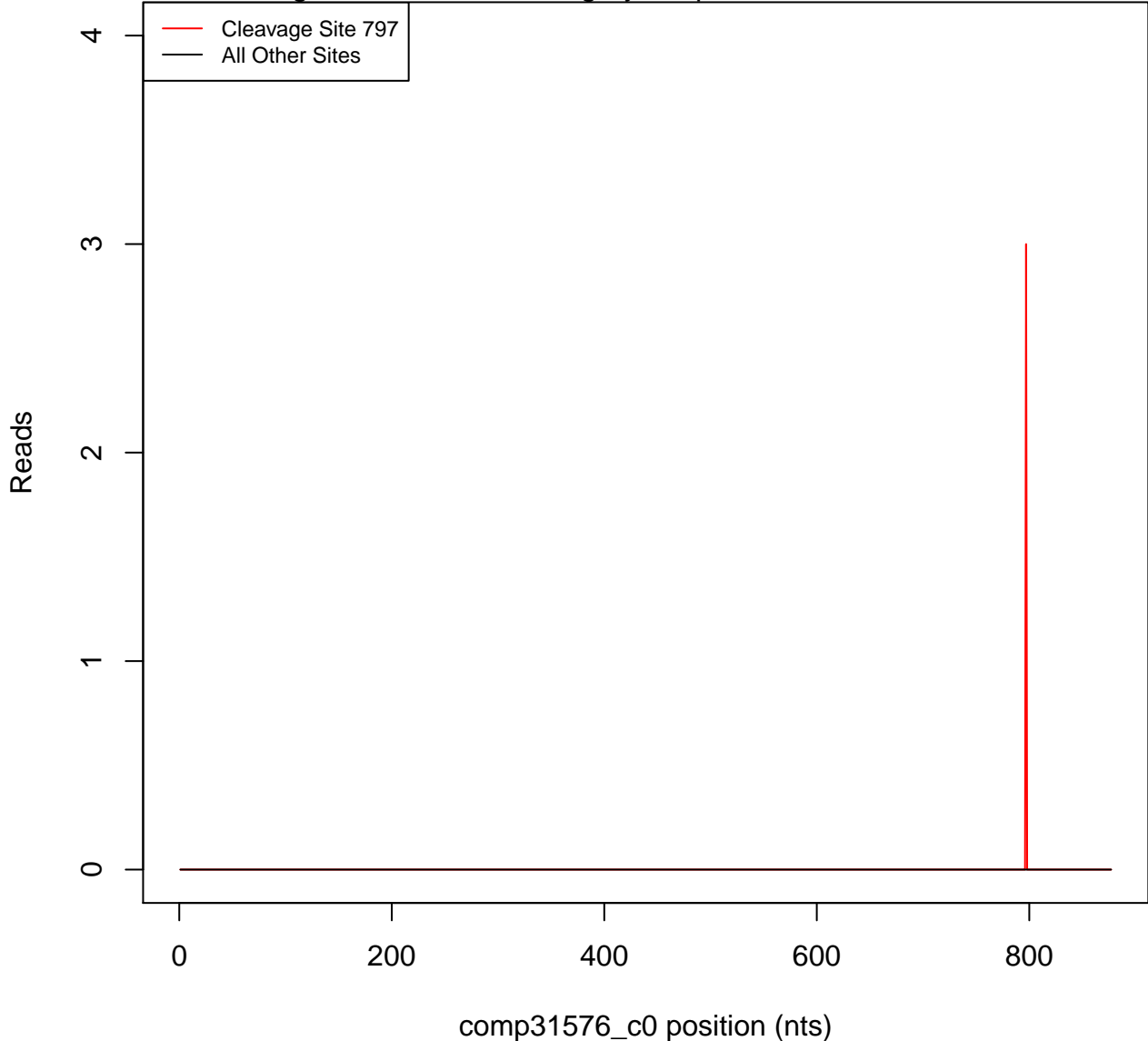

Supplement: S8 File — (ZIP) [file pone.0186500.s014.zip › S8 t-plot of miRNA-target/comp31576_c0--797--mtr-miR156e_1ss2TA_degradome.pdf]

# ath-miR169b slicing comp32005\_c0 at nt 650

alignment score=3 , category=0 , p=0.00740592626877734

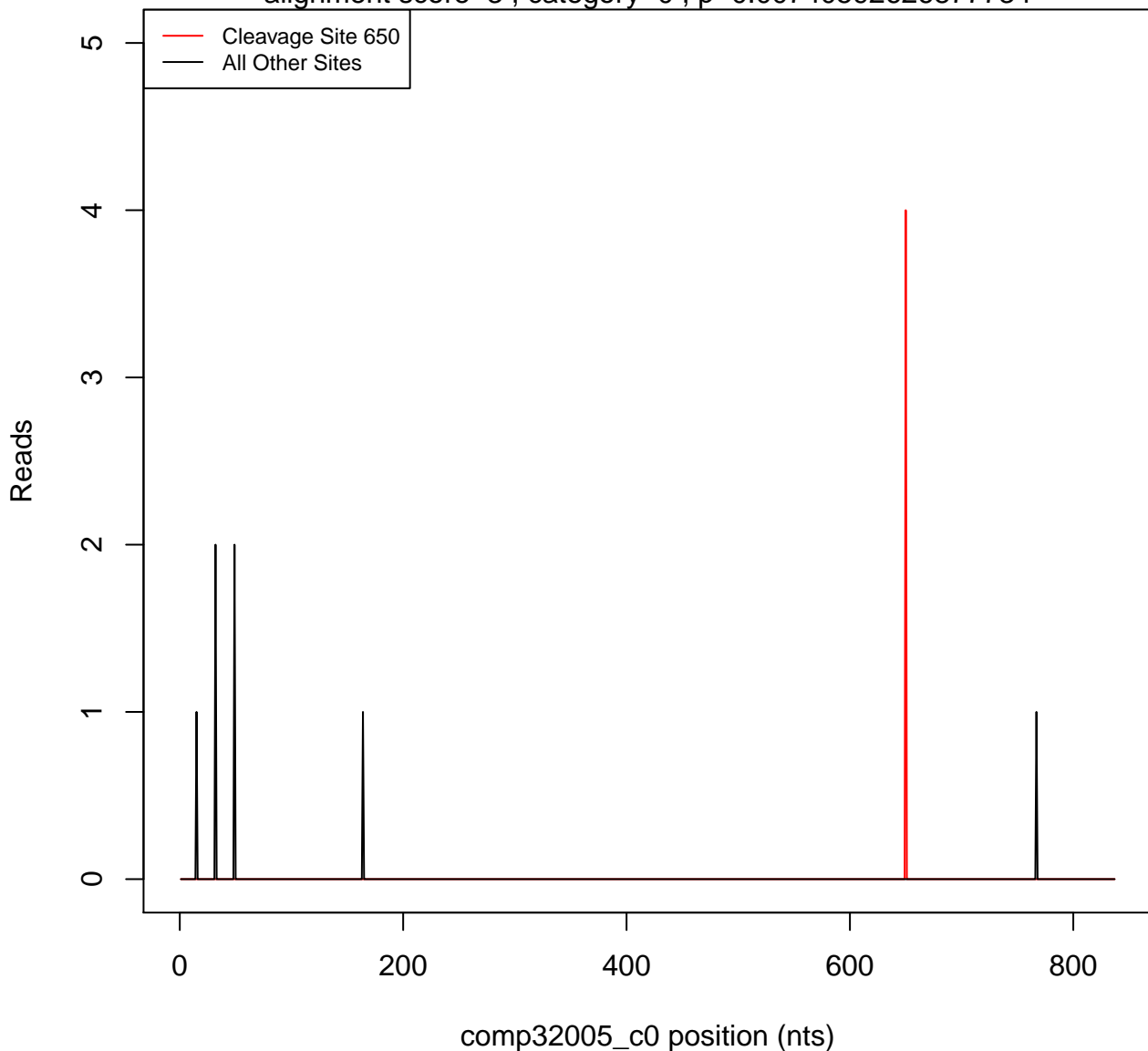

Supplement: S8 File — (ZIP) [file pone.0186500.s014.zip › S8 t-plot of miRNA-target/comp32005_c0--650--ath-miR169b_degradome.pdf]

# ath-miR169h\_1ss21GA slicing comp32005\_c0 at nt 650

alignment score=3 , category=0 , p=0.00494339851921732

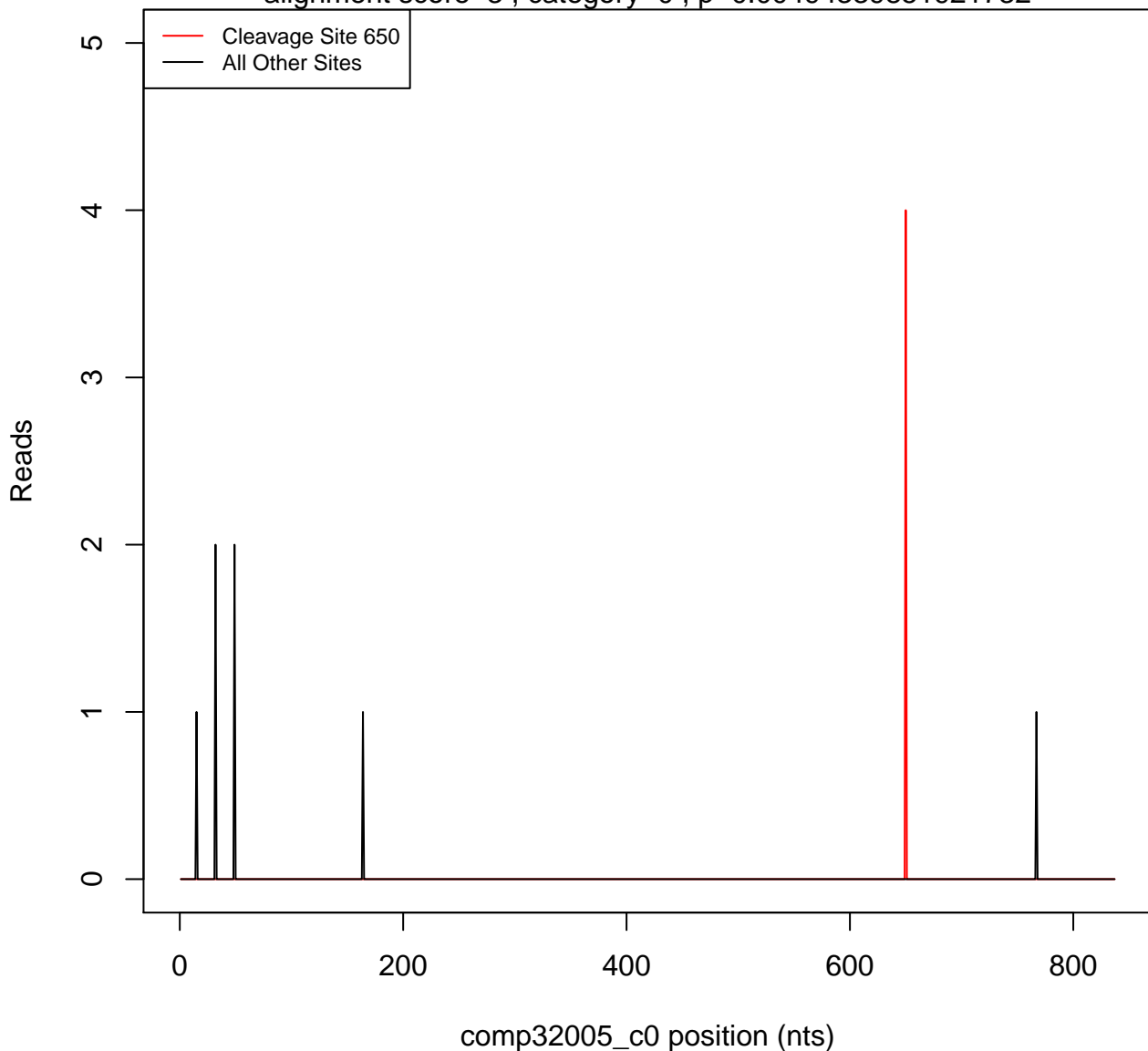

Supplement: S8 File — (ZIP) [file pone.0186500.s014.zip › S8 t-plot of miRNA-target/comp32005_c0--650--ath-miR169h_1ss21GA_degradome.pdf]

# ath-miR169h\_R-1 slicing comp32005\_c0 at nt 650

alignment score=2 , category=0 , p=0.00302387622887079

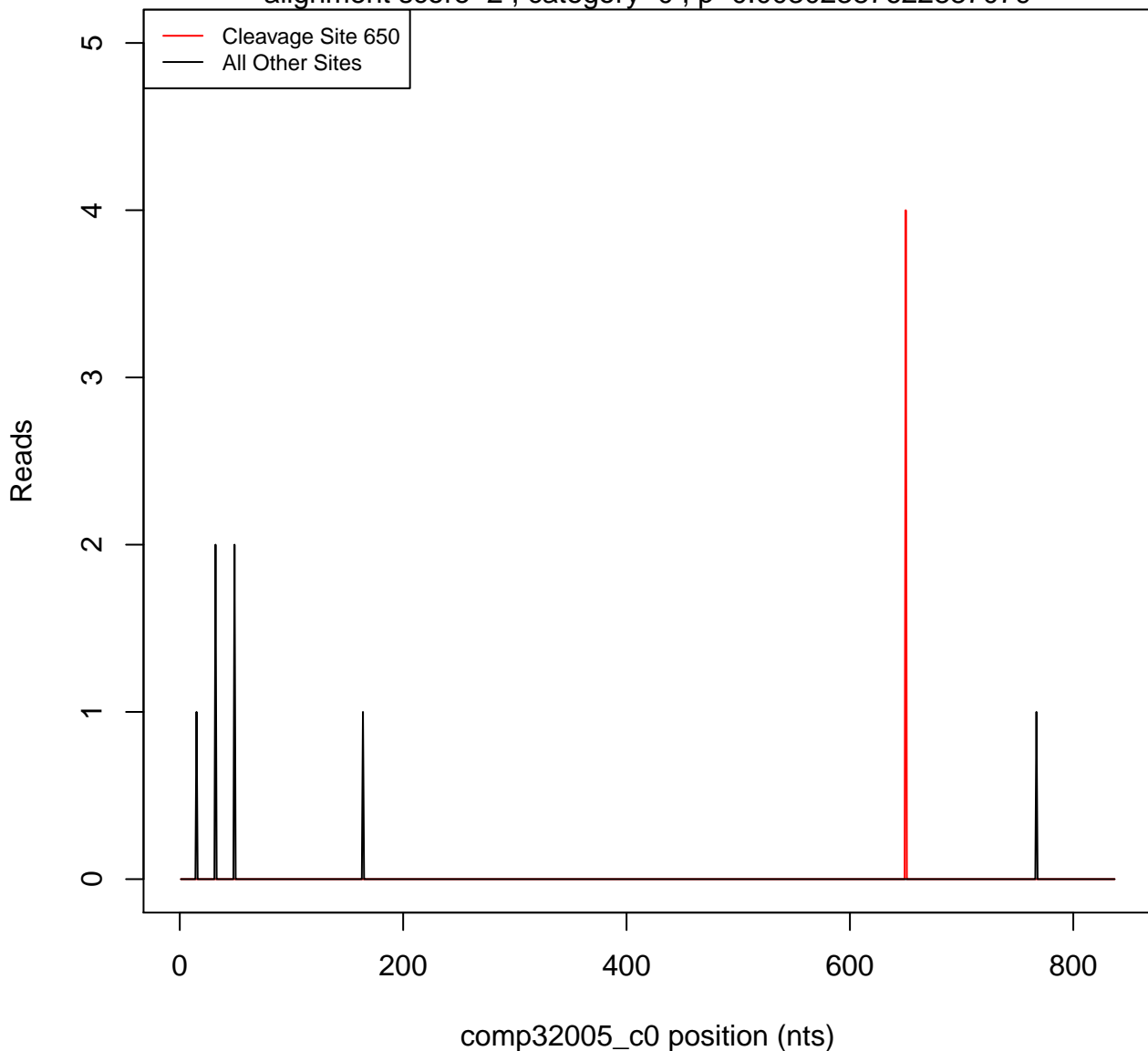

Supplement: S8 File — (ZIP) [file pone.0186500.s014.zip › S8 t-plot of miRNA-target/comp32005_c0--650--ath-miR169h_R-1_degradome.pdf]

# ath-miR169h\_R-3 slicing comp32005\_c0 at nt 650

alignment score=2 , category=0 , p=0.012040752466398

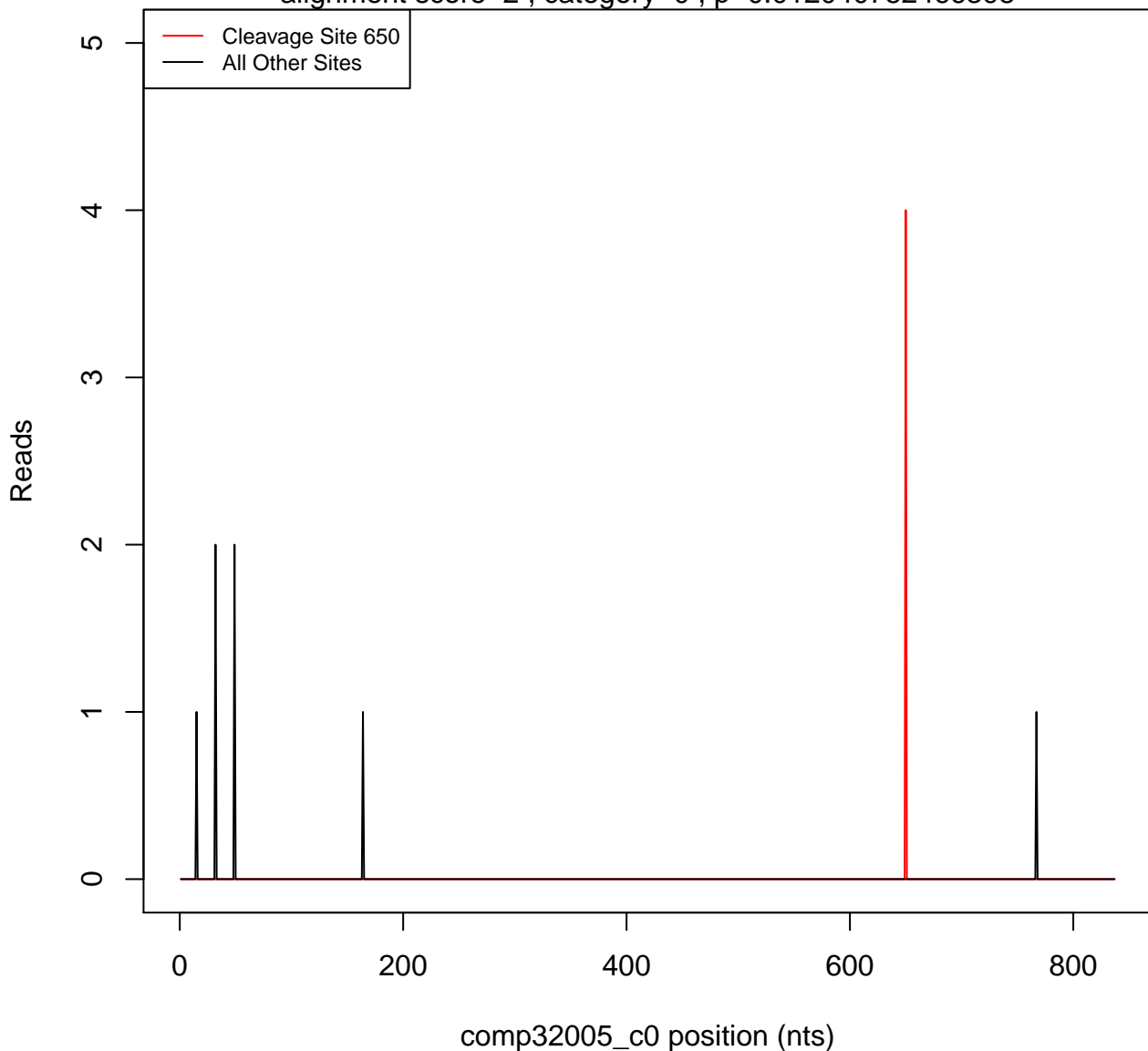

Supplement: S8 File — (ZIP) [file pone.0186500.s014.zip › S8 t-plot of miRNA-target/comp32005_c0--650--ath-miR169h_R-3_degradome.pdf]

# ath-miR169h slicing comp32005\_c0 at nt 650

alignment score=2 , category=0 , p=0.00302387622887079

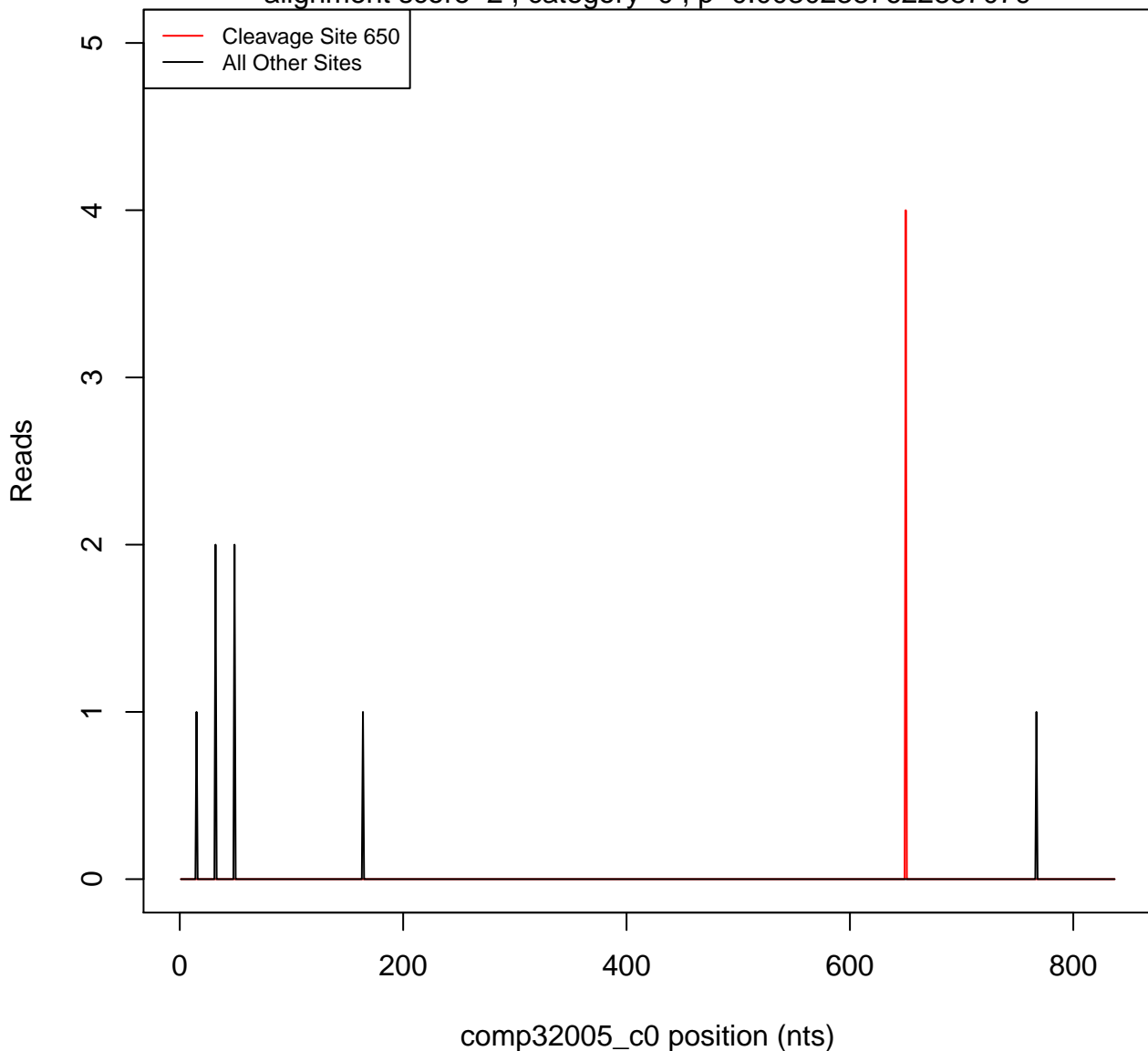

Supplement: S8 File — (ZIP) [file pone.0186500.s014.zip › S8 t-plot of miRNA-target/comp32005_c0--650--ath-miR169h_degradome.pdf]

# bna-miR169g\_1ss22CA slicing comp32005\_c0 at nt 650

alignment score=3 , category=0 , p=0.00247476148180459

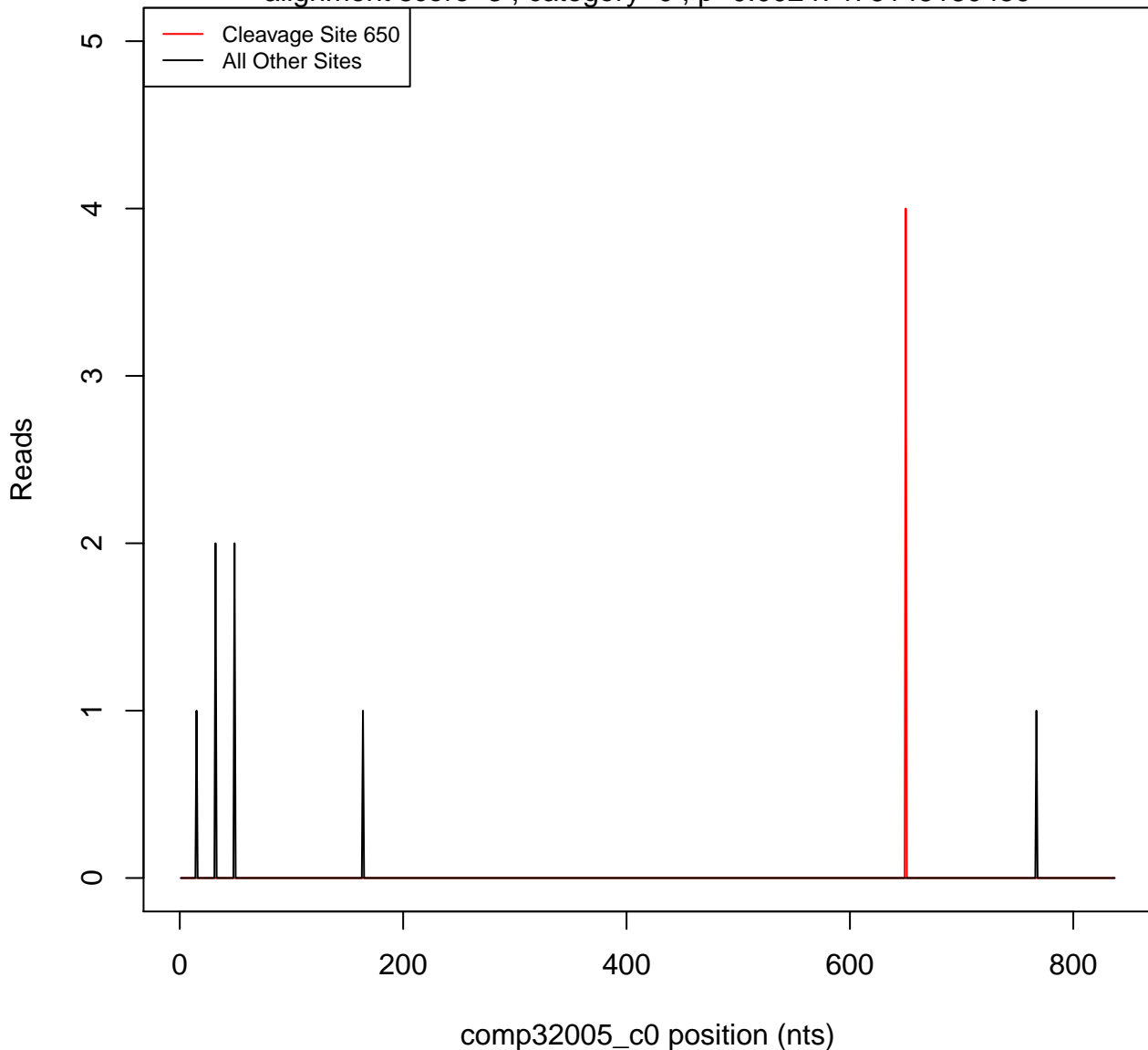

Supplement: S8 File — (ZIP) [file pone.0186500.s014.zip › S8 t-plot of miRNA-target/comp32005_c0--650--bna-miR169g_1ss22CA_degradome.pdf]

# nta-miR169t\_R+1 slicing comp32005\_c0 at nt 650

alignment score=4 , category=0 , p=0.00576491916477573

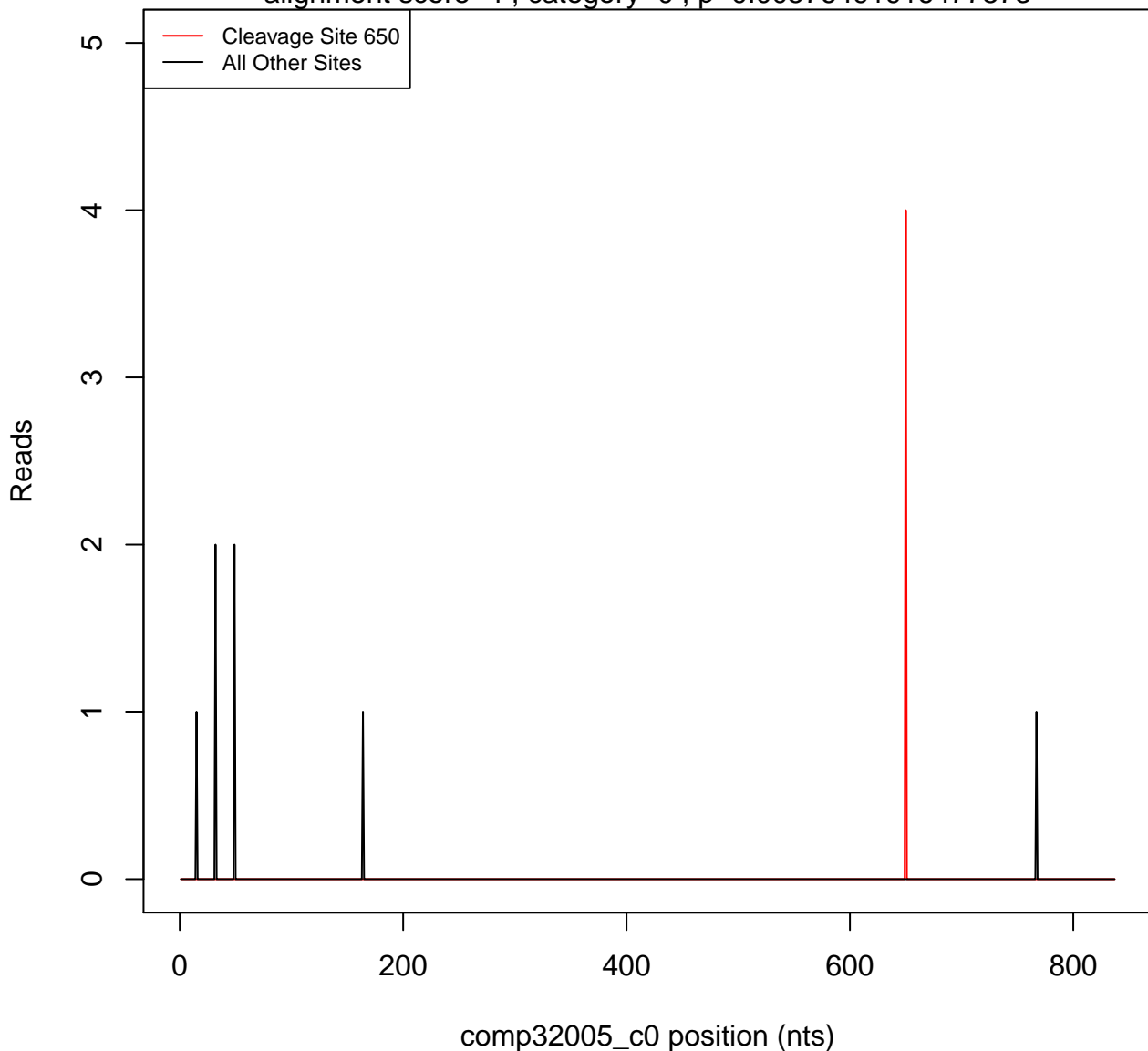

Supplement: S8 File — (ZIP) [file pone.0186500.s014.zip › S8 t-plot of miRNA-target/comp32005_c0--650--nta-miR169t_R+1_degradome.pdf]

# ath-miR396b slicing comp35011\_c0 at nt 159

alignment score=3.5 , category=0 , p=0.00658576156130786

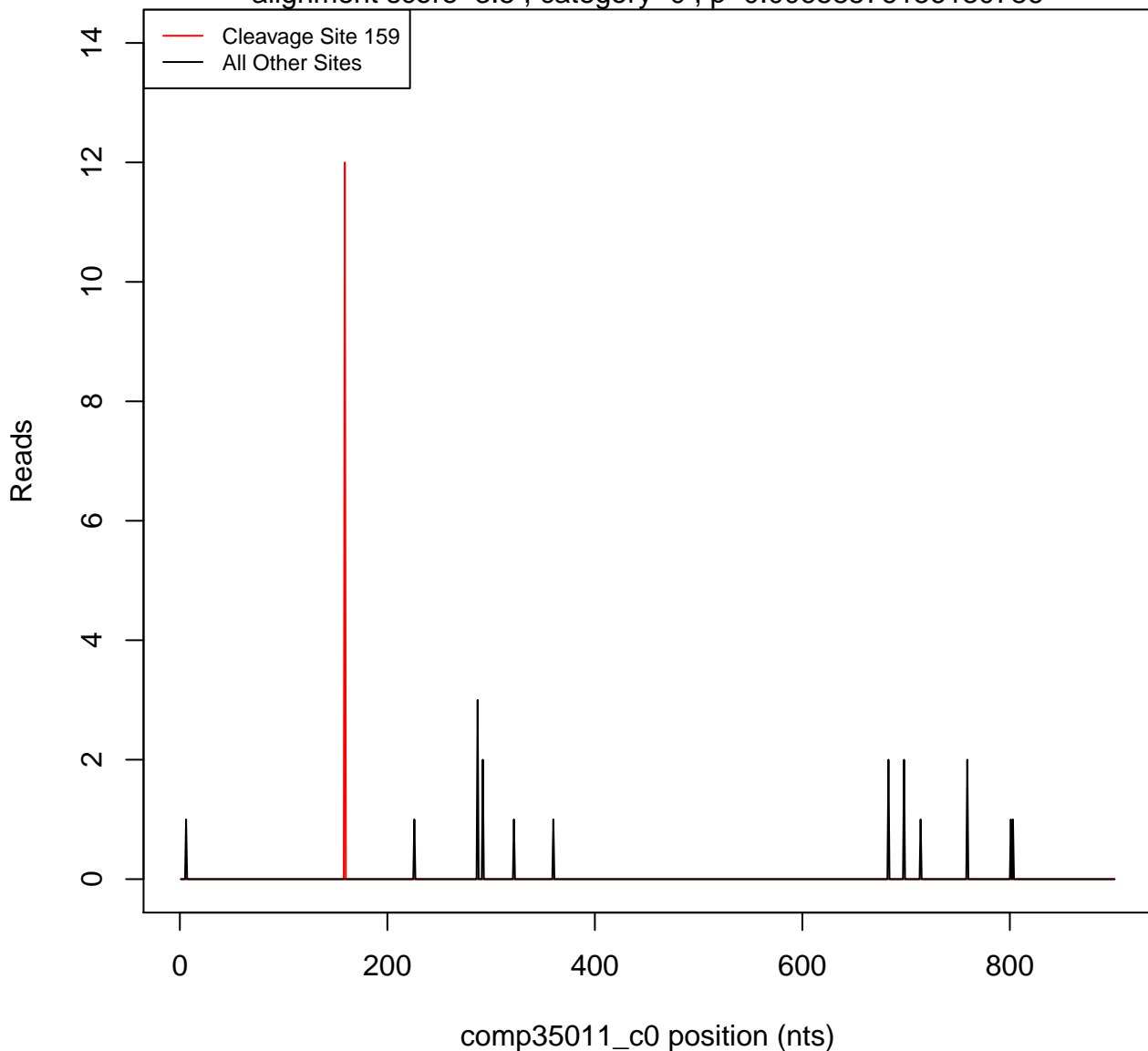

Supplement: S8 File — (ZIP) [file pone.0186500.s014.zip › S8 t-plot of miRNA-target/comp35011_c0--159--ath-miR396b_degradome.pdf]

nta-miR396b\_2ss20TA21TA slicing comp35011\_c0 at nt 159

alignment score=4 , category=0 , p=0.0114966040365749

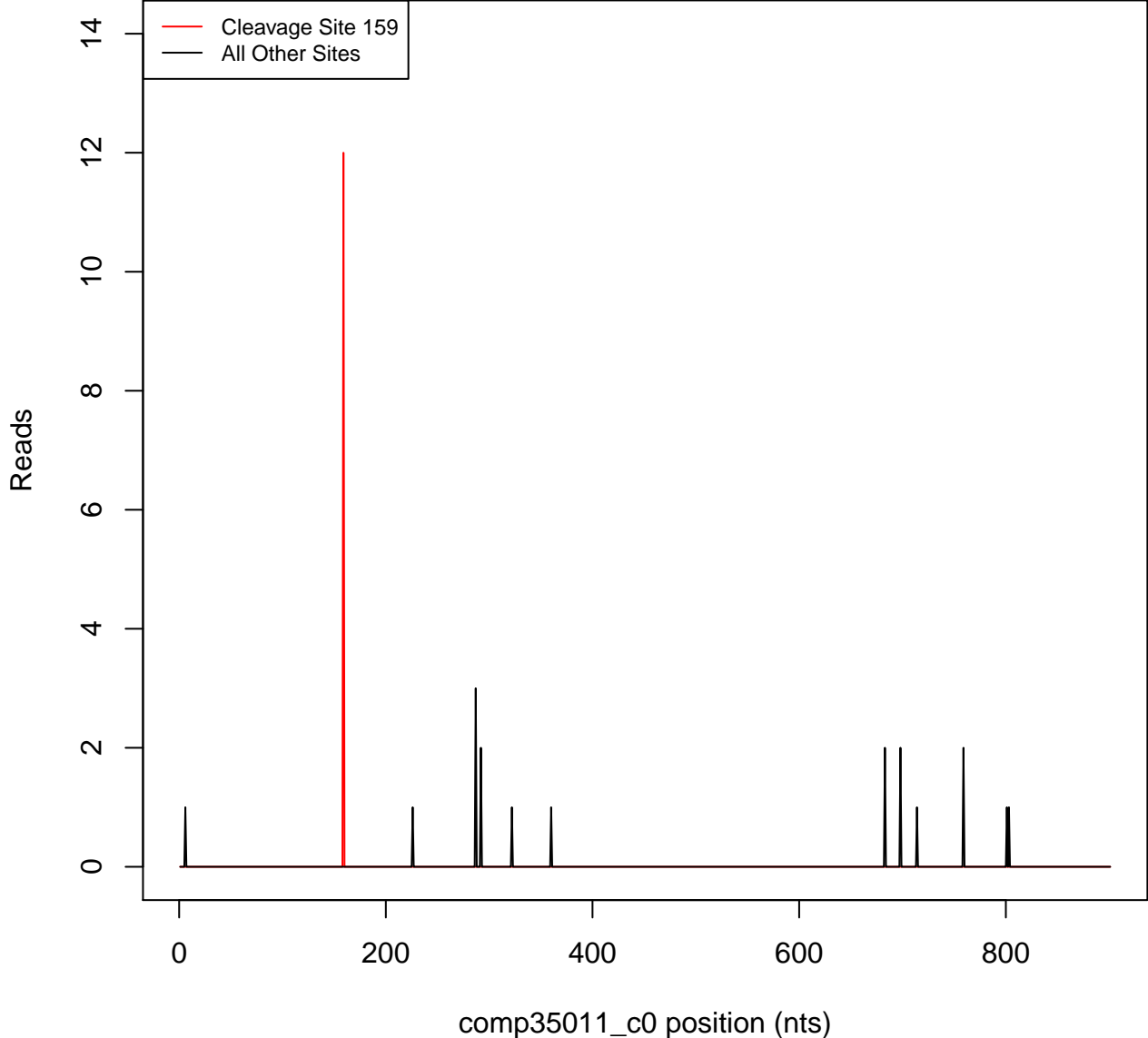

Supplement: S8 File — (ZIP) [file pone.0186500.s014.zip › S8 t-plot of miRNA-target/comp35011_c0--159--nta-miR396b_2ss20TA21TA_degradome.pdf]

**ath-miR165a\_2ss17CT21CT slicing comp36994\_c0 at nt 989**

alignment score=3 , category=0 , p=0.00247476148180459

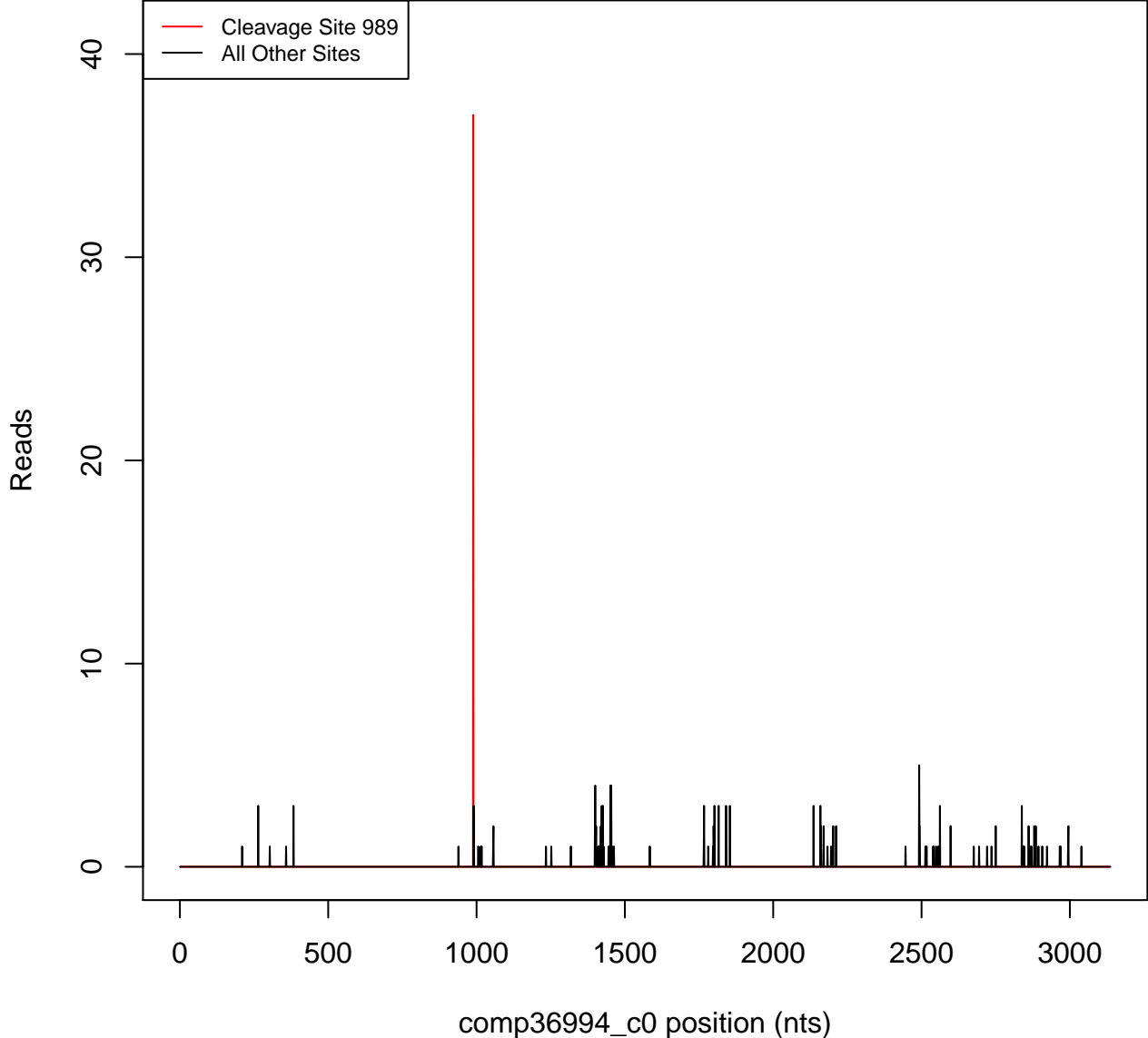

Supplement: S8 File — (ZIP) [file pone.0186500.s014.zip › S8 t-plot of miRNA-target/comp36994_c0--989--ath-miR165a_2ss17CT21CT_degradome.pdf]

# ath-miR166a\_1ss17TC slicing comp36994\_c0 at nt 989

alignment score=2.5 , category=0 , p=0.00274935665012399

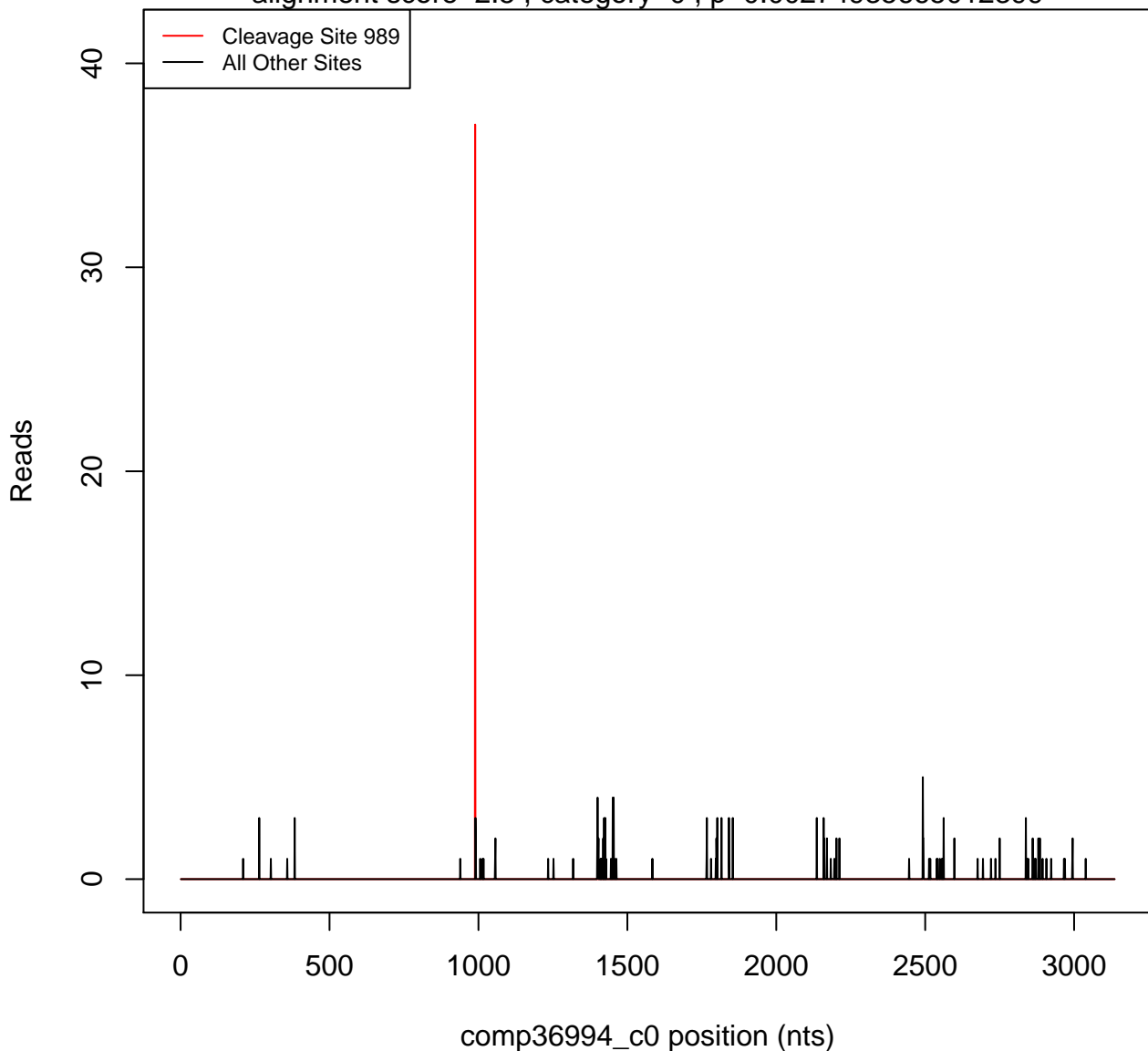

Supplement: S8 File — (ZIP) [file pone.0186500.s014.zip › S8 t-plot of miRNA-target/comp36994_c0--989--ath-miR166a_1ss17TC_degradome.pdf]

# ath-miR166a\_1ss20CT slicing comp36994\_c0 at nt 989

alignment score=3 , category=0 , p=0.00247476148180459

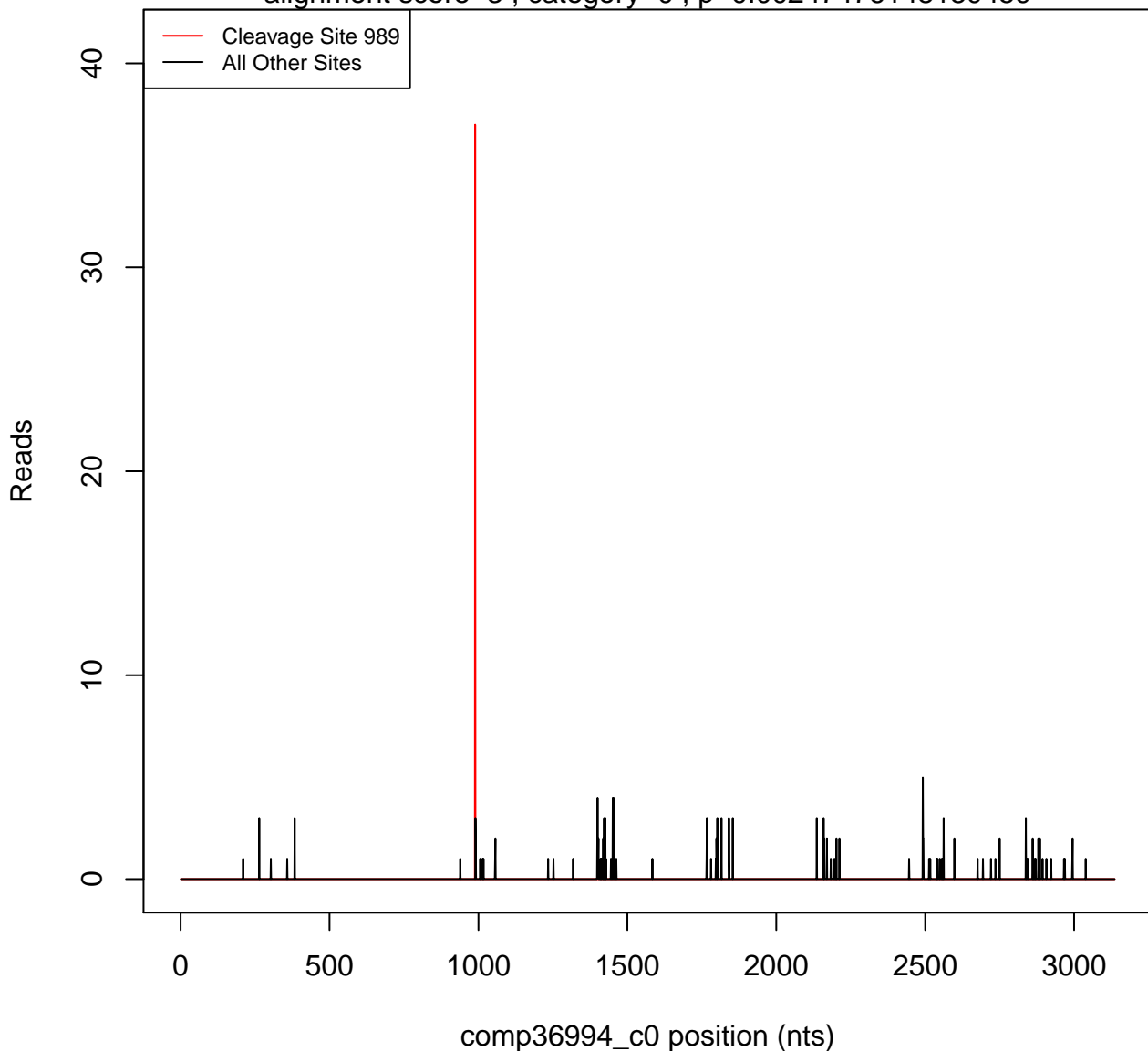

Supplement: S8 File — (ZIP) [file pone.0186500.s014.zip › S8 t-plot of miRNA-target/comp36994_c0--989--ath-miR166a_1ss20CT_degradome.pdf]

# ath-miR166a slicing comp36994\_c0 at nt 989

alignment score=3 , category=0 , p=0.00247476148180459

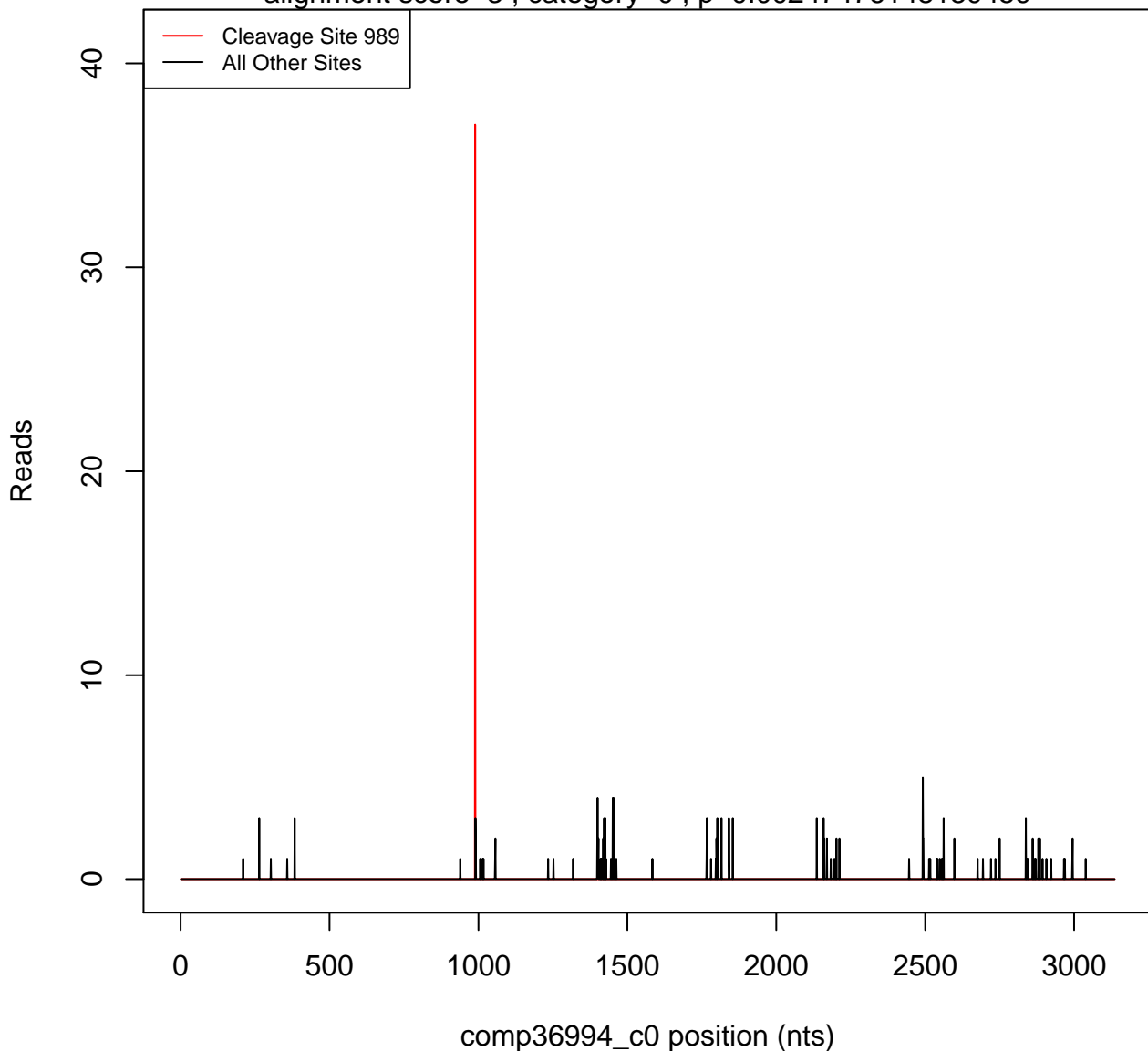

Supplement: S8 File — (ZIP) [file pone.0186500.s014.zip › S8 t-plot of miRNA-target/comp36994_c0--989--ath-miR166a_degradome.pdf]

# cme-MIR166i-p3\_1ss18CT slicing comp36994\_c0 at nt 989

alignment score=2 , category=0 , p=0.00302387622887079

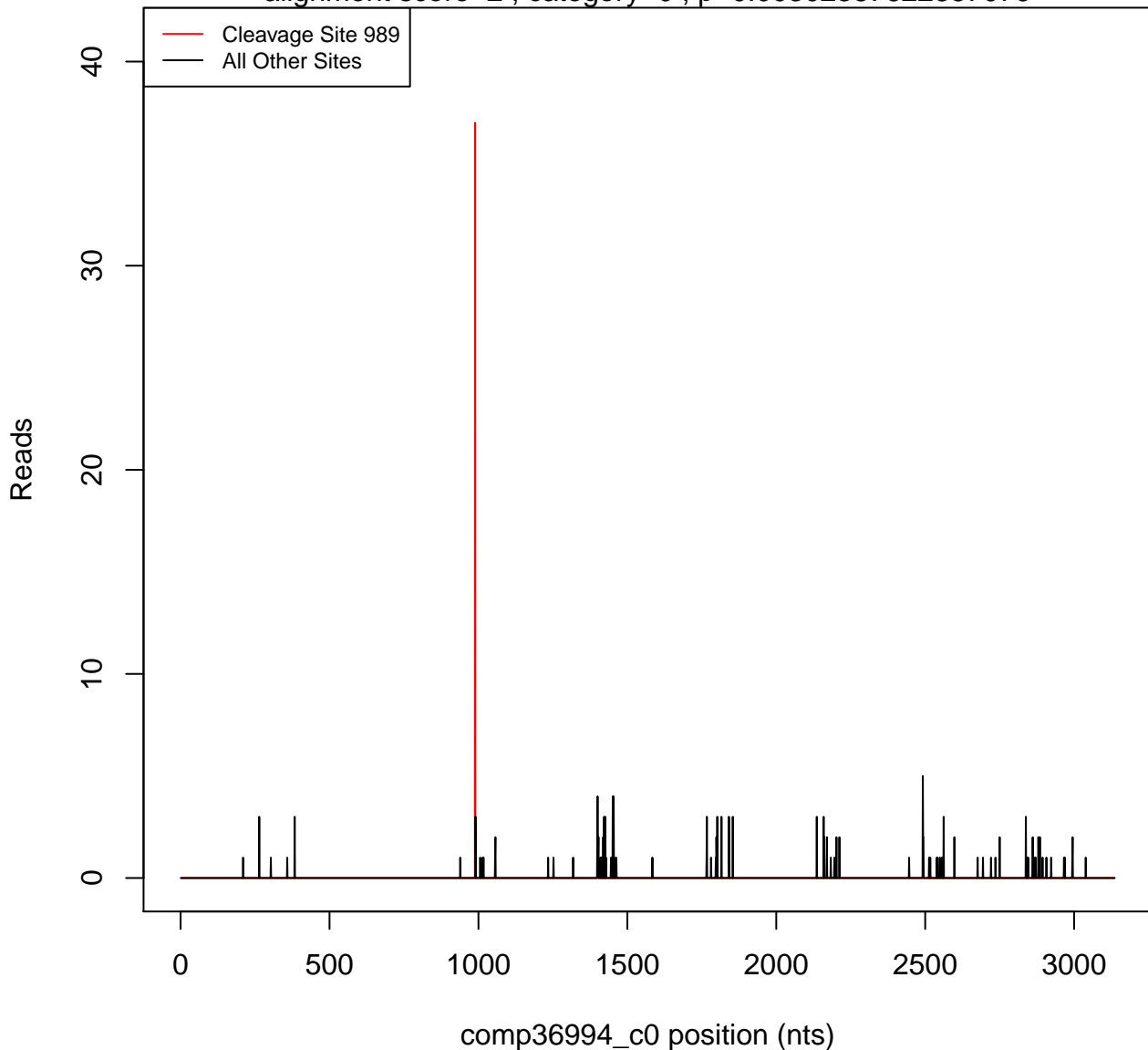

Supplement: S8 File — (ZIP) [file pone.0186500.s014.zip › S8 t-plot of miRNA-target/comp36994_c0--989--cme-MIR166i-p3_1ss18CT_degradome.pdf]

# ath-miR166a\_L+1R-1 slicing comp36994\_c0 at nt 990

alignment score=2.5 , category=2 , p=0.0710285464129122

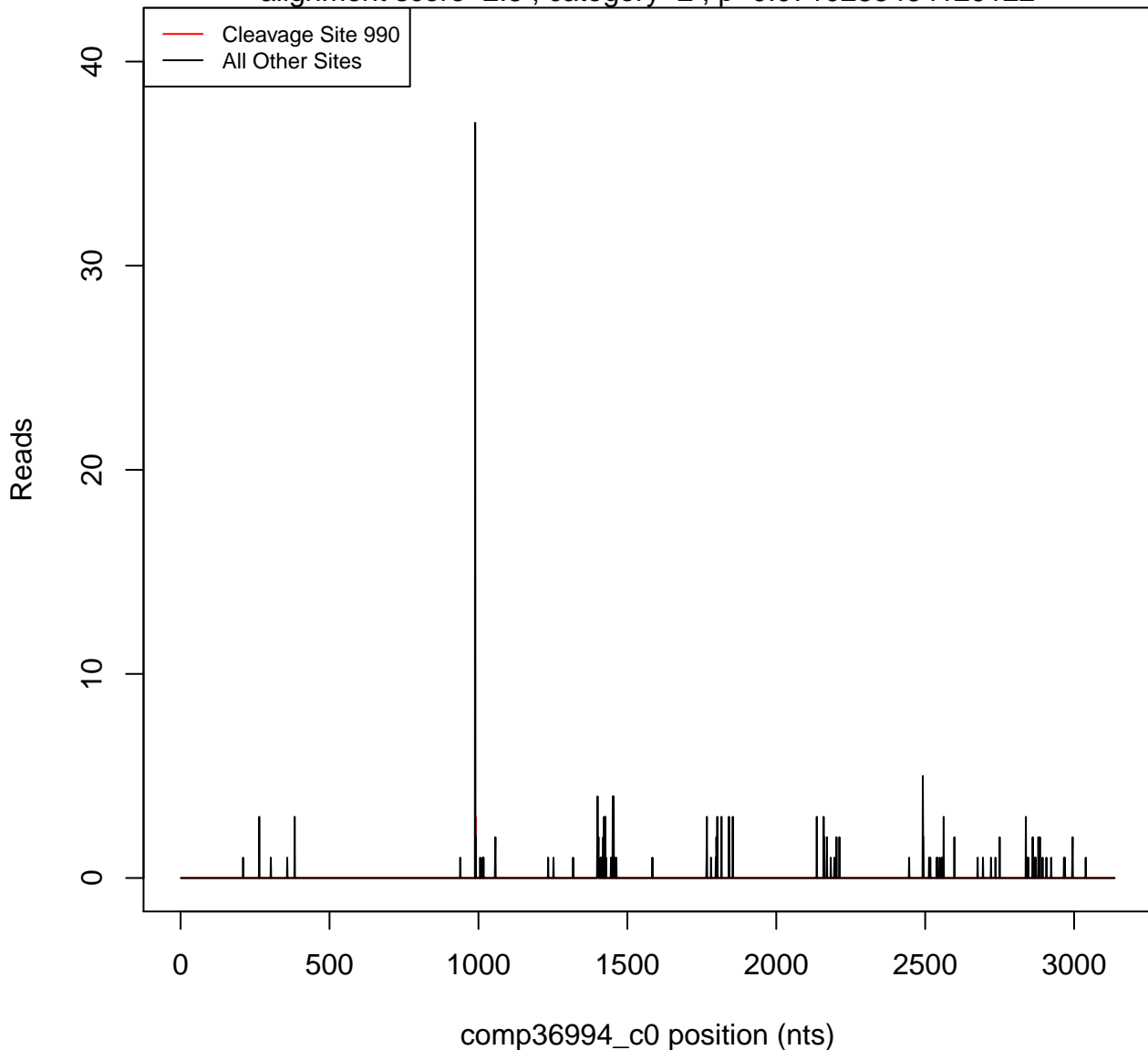

Supplement: S8 File — (ZIP) [file pone.0186500.s014.zip › S8 t-plot of miRNA-target/comp36994_c0--990--ath-miR166a_L+1R-1_degradome.pdf]

# ath-miR408\_L-1R+1 slicing comp37963\_c0 at nt 104

alignment score=1.5 , category=0 , p=0.0032983202388529

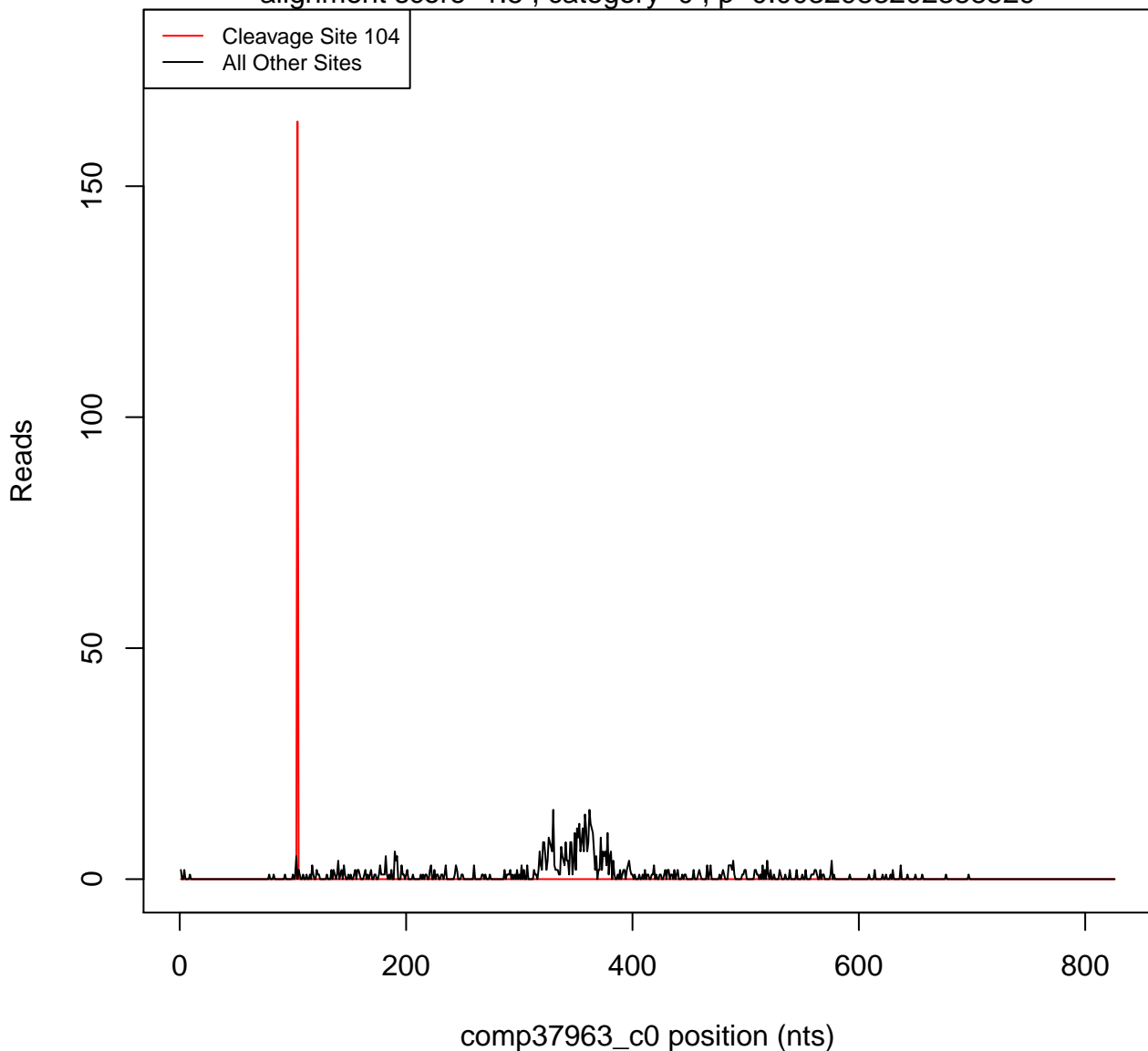

Supplement: S8 File — (ZIP) [file pone.0186500.s014.zip › S8 t-plot of miRNA-target/comp37963_c0--104--ath-miR408_L-1R+1_degradome.pdf]

# ath-miR408\_L-1R+1 slicing comp39319\_c0 at nt 652

alignment score=2.5 , category=0 , p=0.00274935665012399

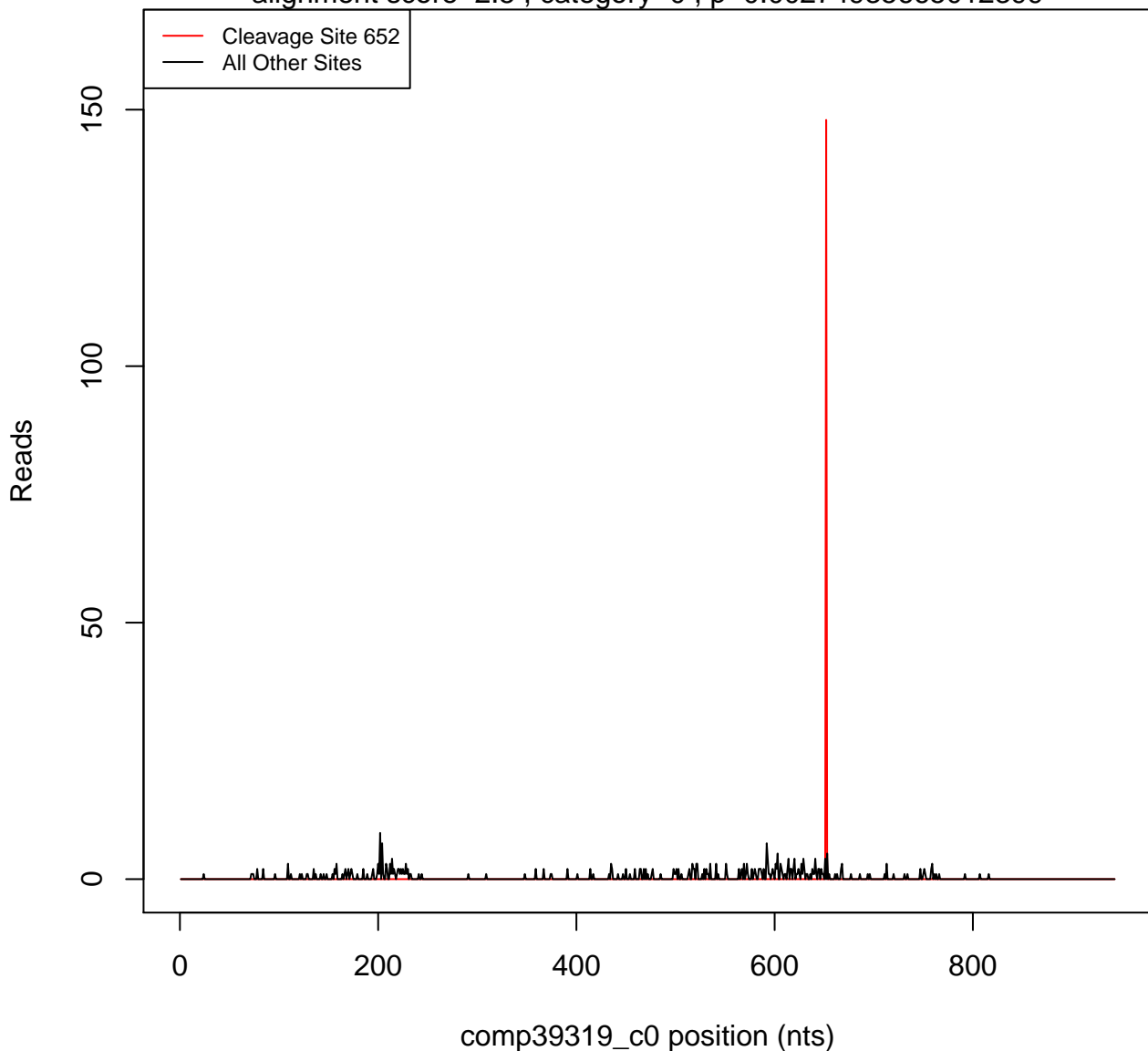

Supplement: S8 File — (ZIP) [file pone.0186500.s014.zip › S8 t-plot of miRNA-target/comp39319_c0--652--ath-miR408_L-1R+1_degradome.pdf]

**aly-miR157a-3p\_R-1\_2ss9GT10CG slicing comp39747\_c0 at nt 868**

alignment score=3.5 , category=2 , p=0.0572383778530186

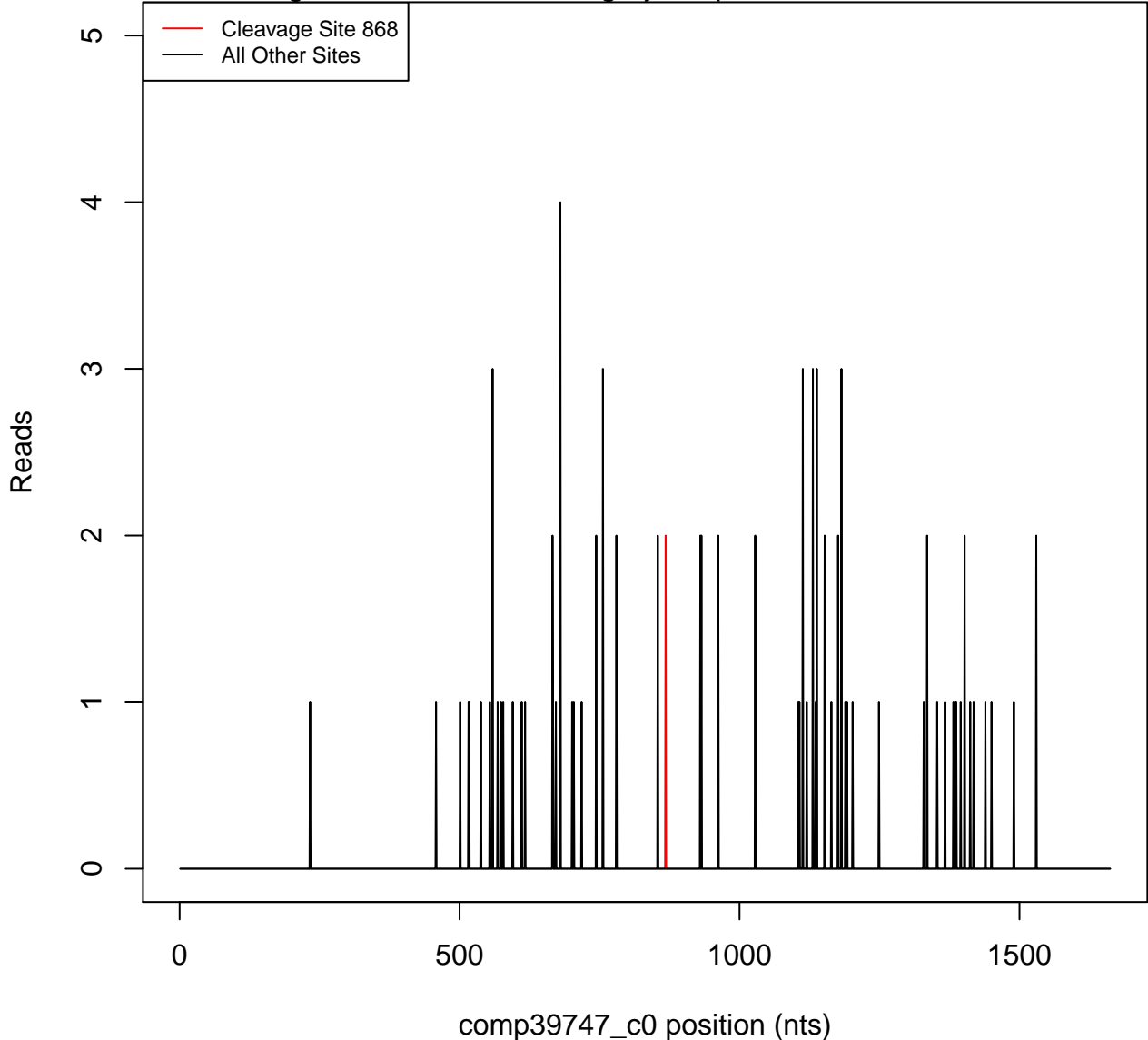

Supplement: S8 File — (ZIP) [file pone.0186500.s014.zip › S8 t-plot of miRNA-target/comp39747_c0--868--aly-miR157a-3p_R-1_2ss9GT10CG_degradome.pdf]

# gma-miR171k-3p slicing comp39988\_c1 at nt 548

alignment score=3 , category=2 , p=0.0641588625669407

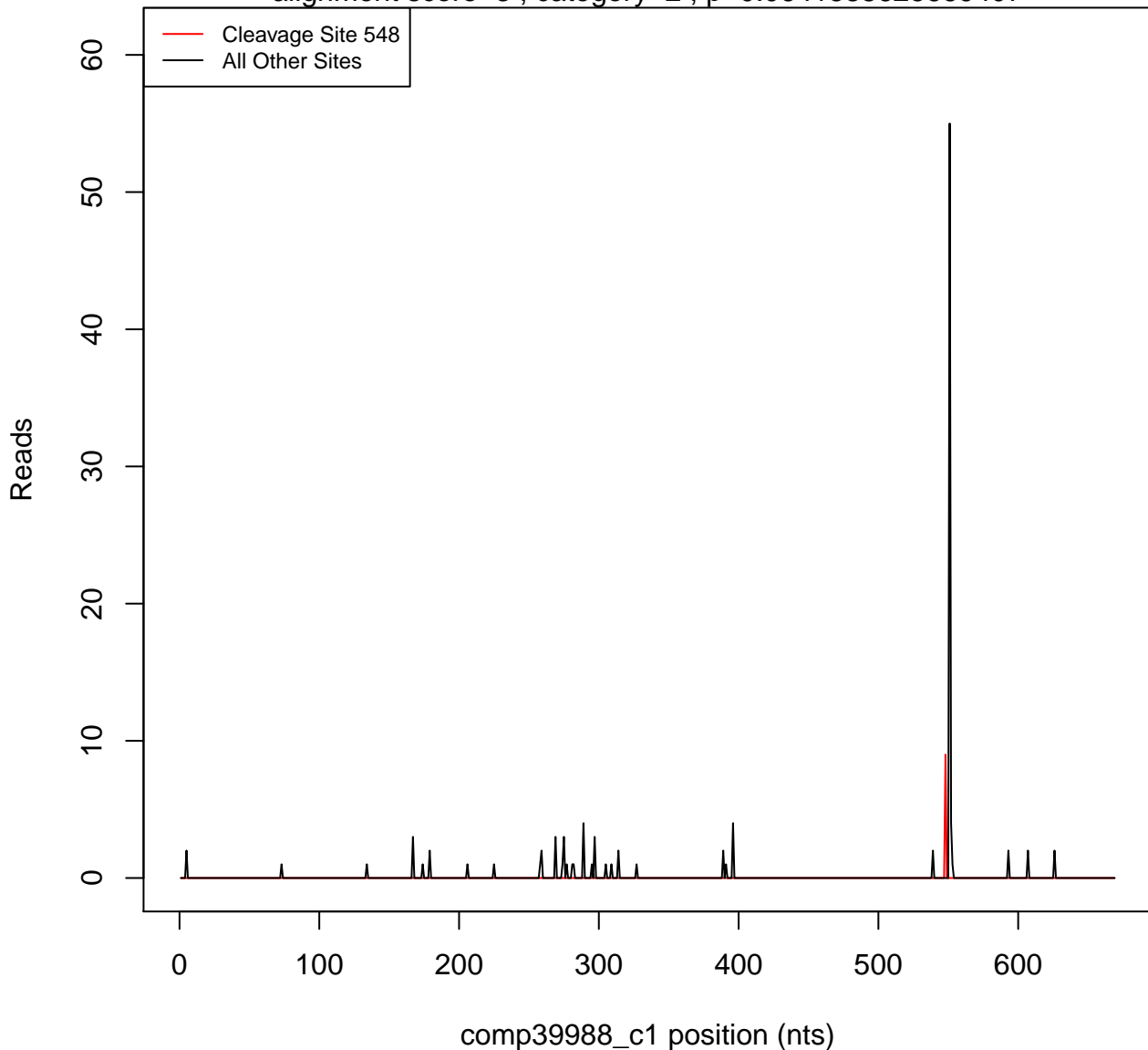

Supplement: S8 File — (ZIP) [file pone.0186500.s014.zip › S8 t-plot of miRNA-target/comp39988_c1--548--gma-miR171k-3p_degradome.pdf]

# aqc-miR171a slicing comp39988\_c1 at nt 551

alignment score=0 , category=0 , p=0.00412119906420627

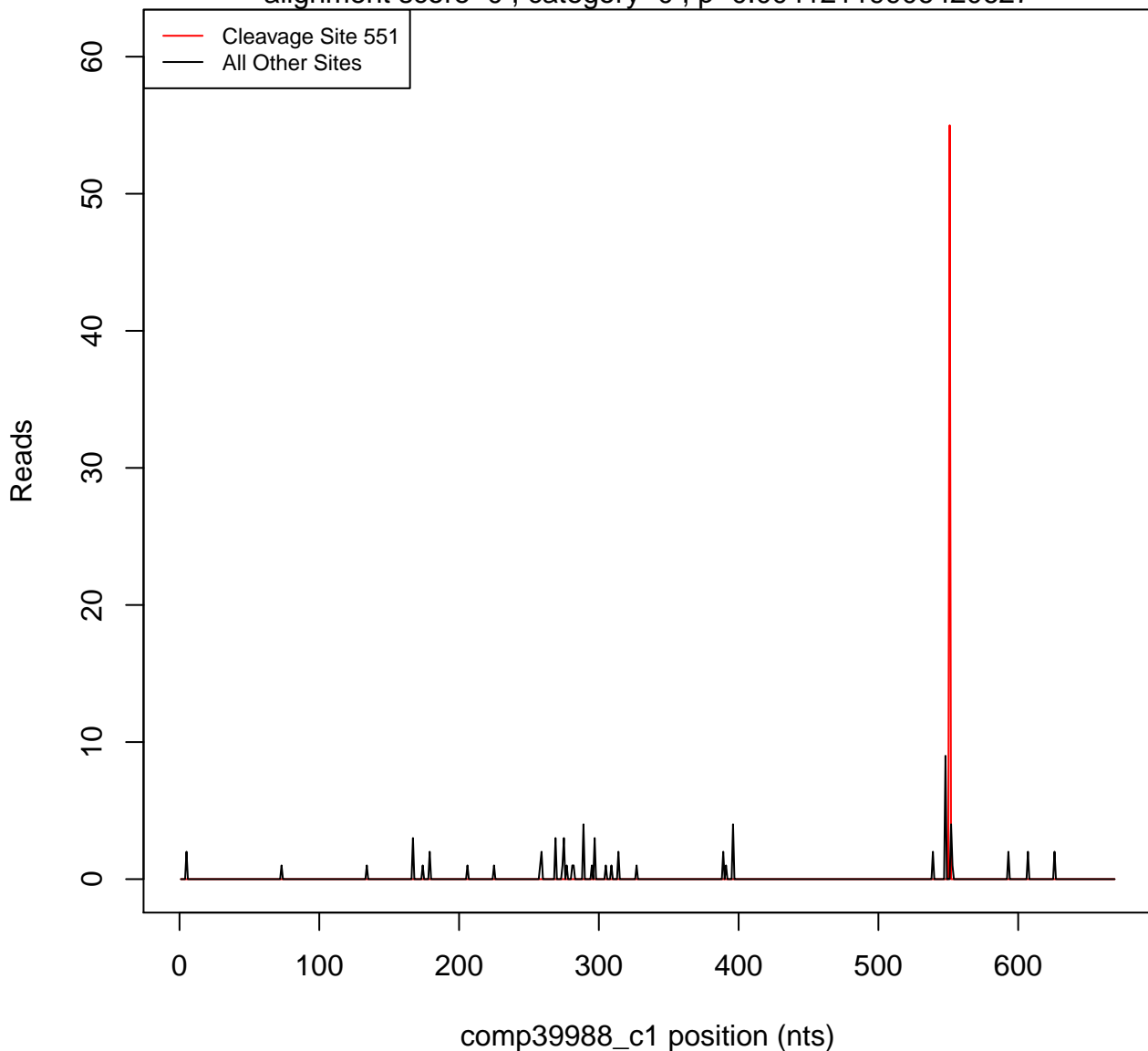

Supplement: S8 File — (ZIP) [file pone.0186500.s014.zip › S8 t-plot of miRNA-target/comp39988_c1--551--aqc-miR171a_degradome.pdf]

# ath-miR171a\_2ss12CT21CT slicing comp39988\_c1 at nt 551

alignment score=0.5 , category=0 , p=0.00384698163572694

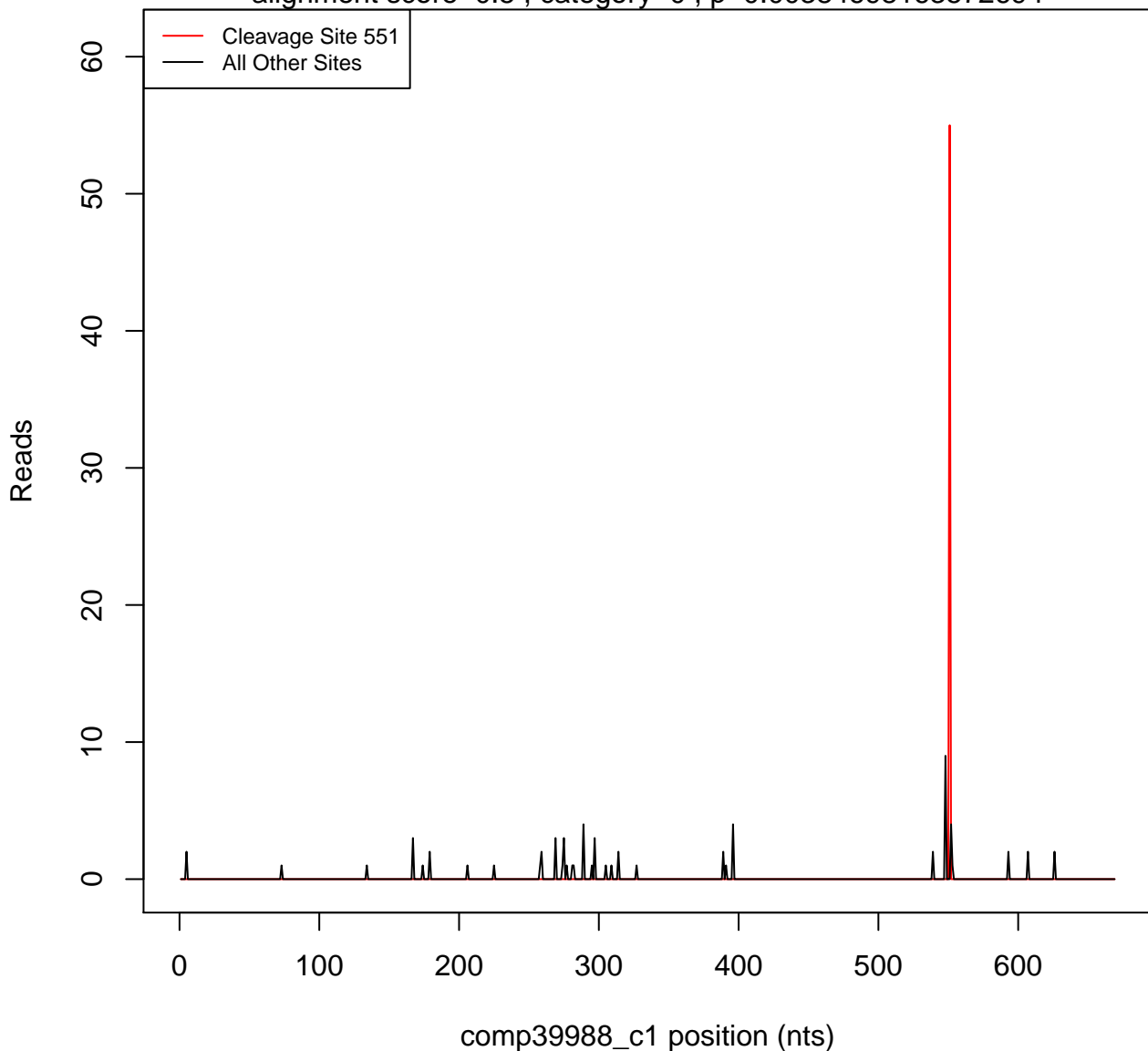

Supplement: S8 File — (ZIP) [file pone.0186500.s014.zip › S8 t-plot of miRNA-target/comp39988_c1--551--ath-miR171a_2ss12CT21CT_degradome.pdf]

# ath-miR396b slicing comp408434\_c0 at nt 104

alignment score=1 , category=2 , p=0.0913365081909329

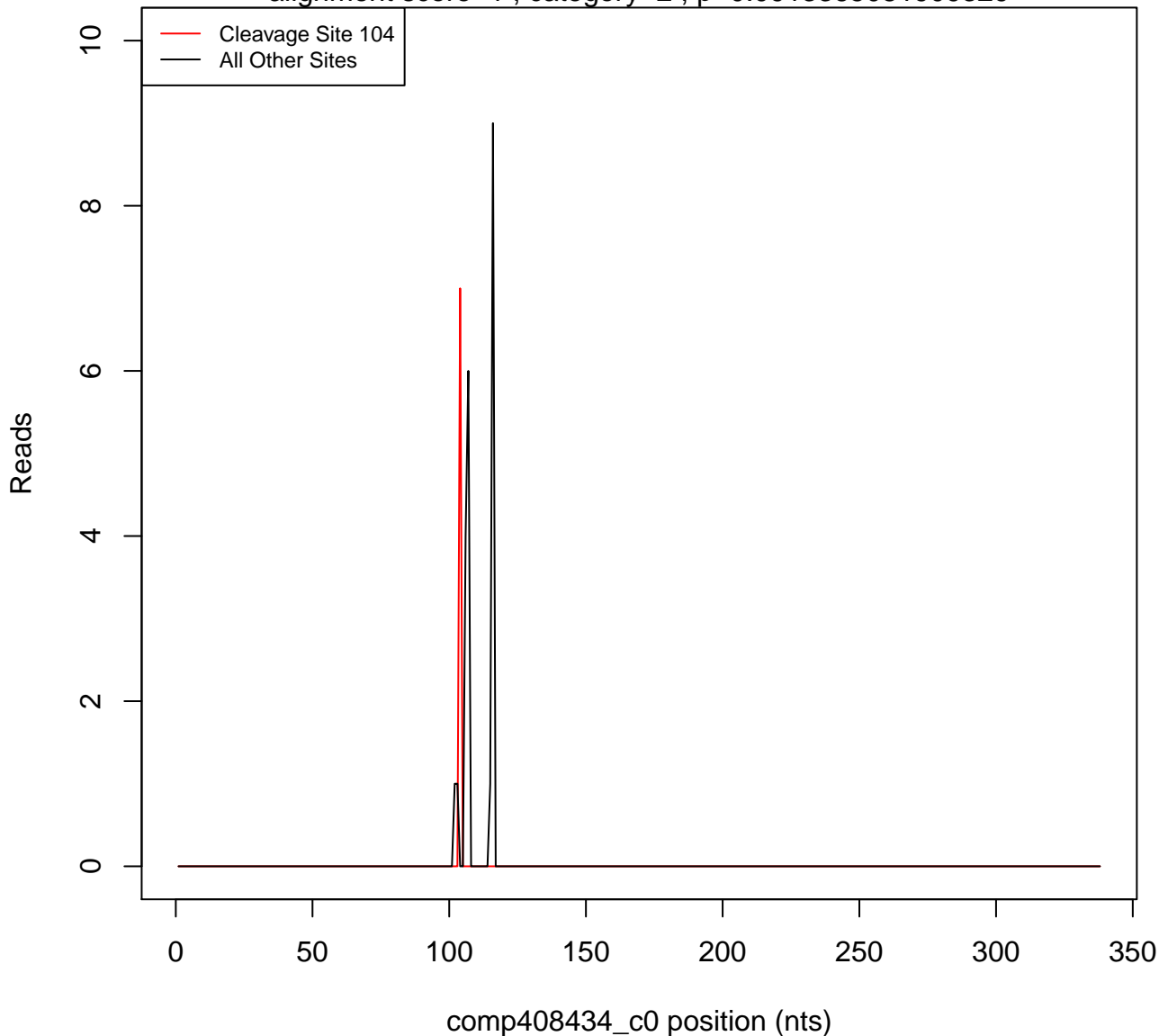

Supplement: S8 File — (ZIP) [file pone.0186500.s014.zip › S8 t-plot of miRNA-target/comp408434_c0--104--ath-miR396b_degradome.pdf]

# gma-miR396a-5p\_R+2 slicing comp408434\_c0 at nt 104

alignment score=3 , category=2 , p=0.0641588625669407

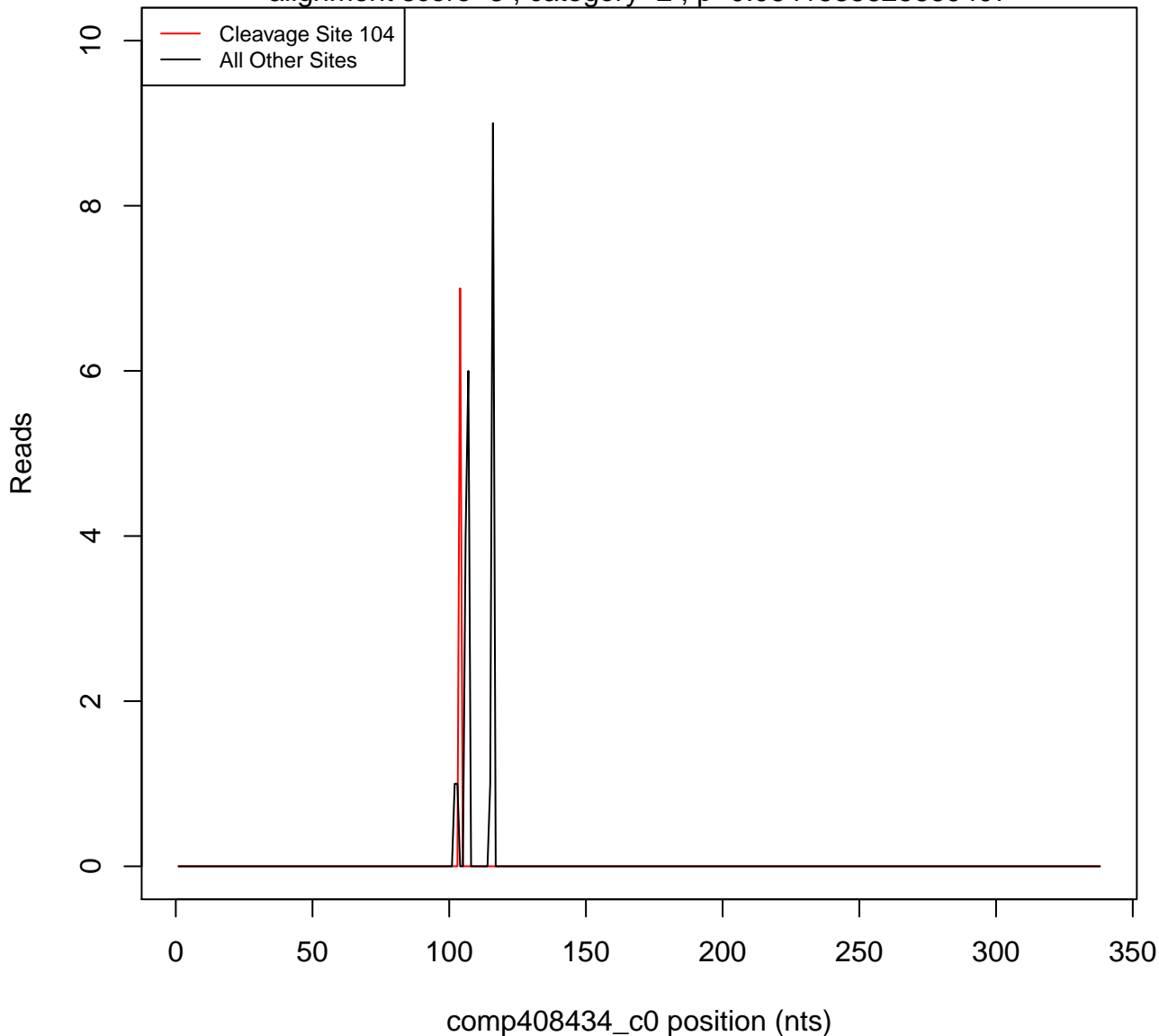

Supplement: S8 File — (ZIP) [file pone.0186500.s014.zip › S8 t-plot of miRNA-target/comp408434_c0--104--gma-miR396a-5p_R+2_degradome.pdf]

# ath-miR394a slicing comp40897\_c0 at nt 1337

alignment score=1 , category=2 , p=0.0913365081909329

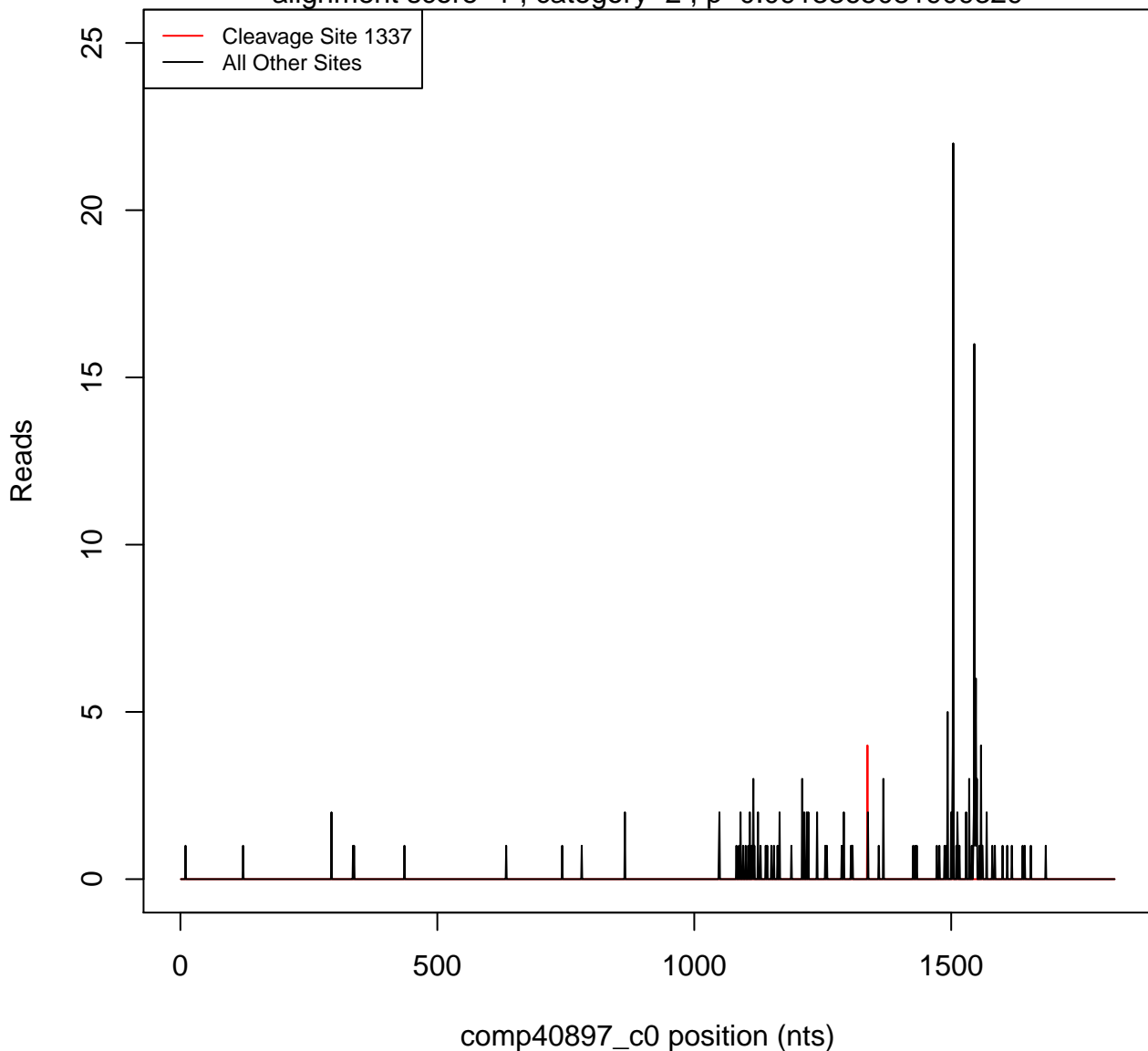

Supplement: S8 File — (ZIP) [file pone.0186500.s014.zip › S8 t-plot of miRNA-target/comp40897_c0--1337--ath-miR394a_degradome.pdf]

# smo-MIR1103-p3\_1ss13CT slicing comp40908\_c0 at nt 403

alignment score=4 , category=2 , p=0.303033976979963

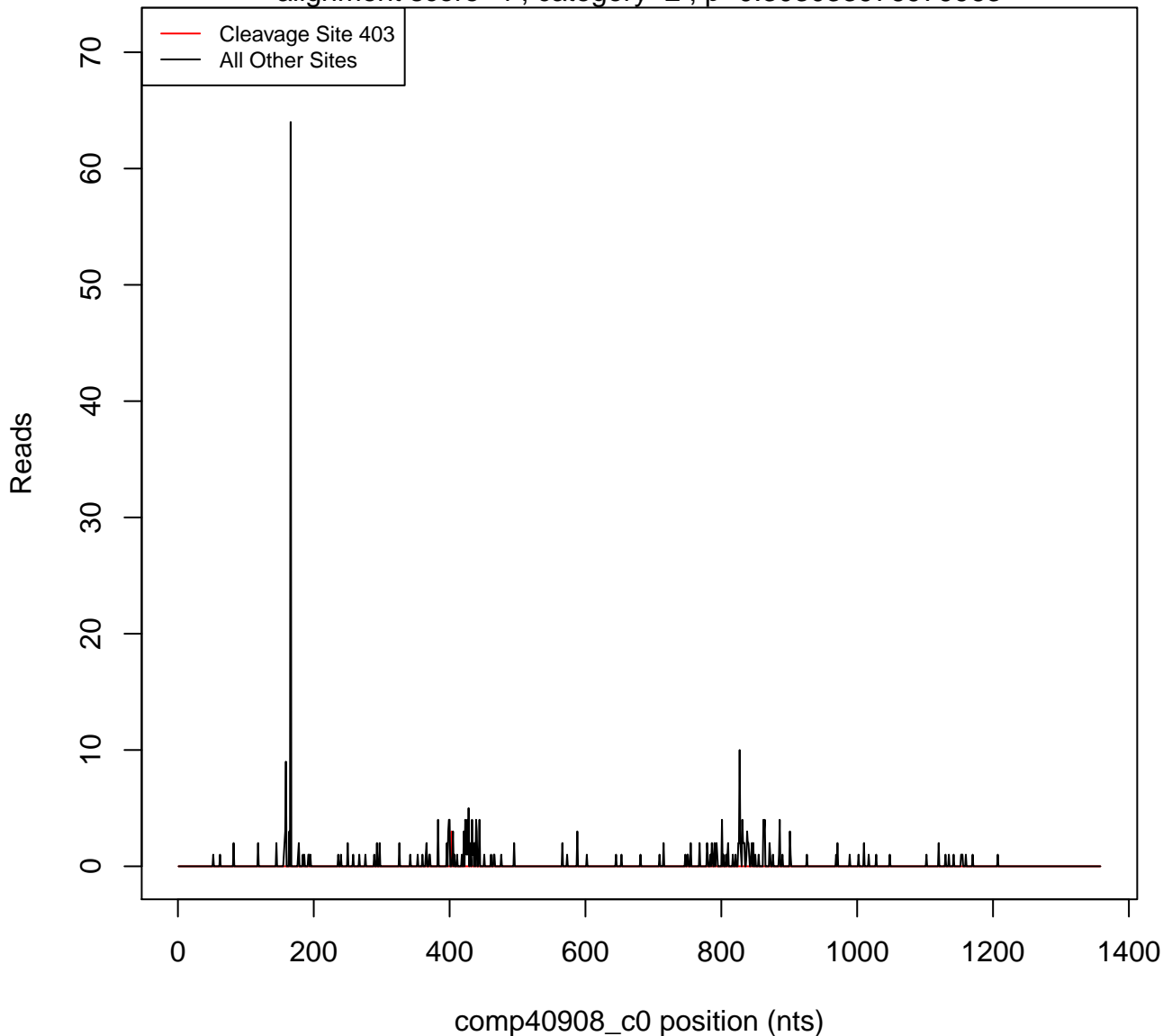

Supplement: S8 File — (ZIP) [file pone.0186500.s014.zip › S8 t-plot of miRNA-target/comp40908_c0--403--smo-MIR1103-p3_1ss13CT_degradome.pdf]

# ath-miR169h\_1ss21GA slicing comp41128\_c0 at nt 1306

alignment score=4 , category=1 , p=0.00469519130044582

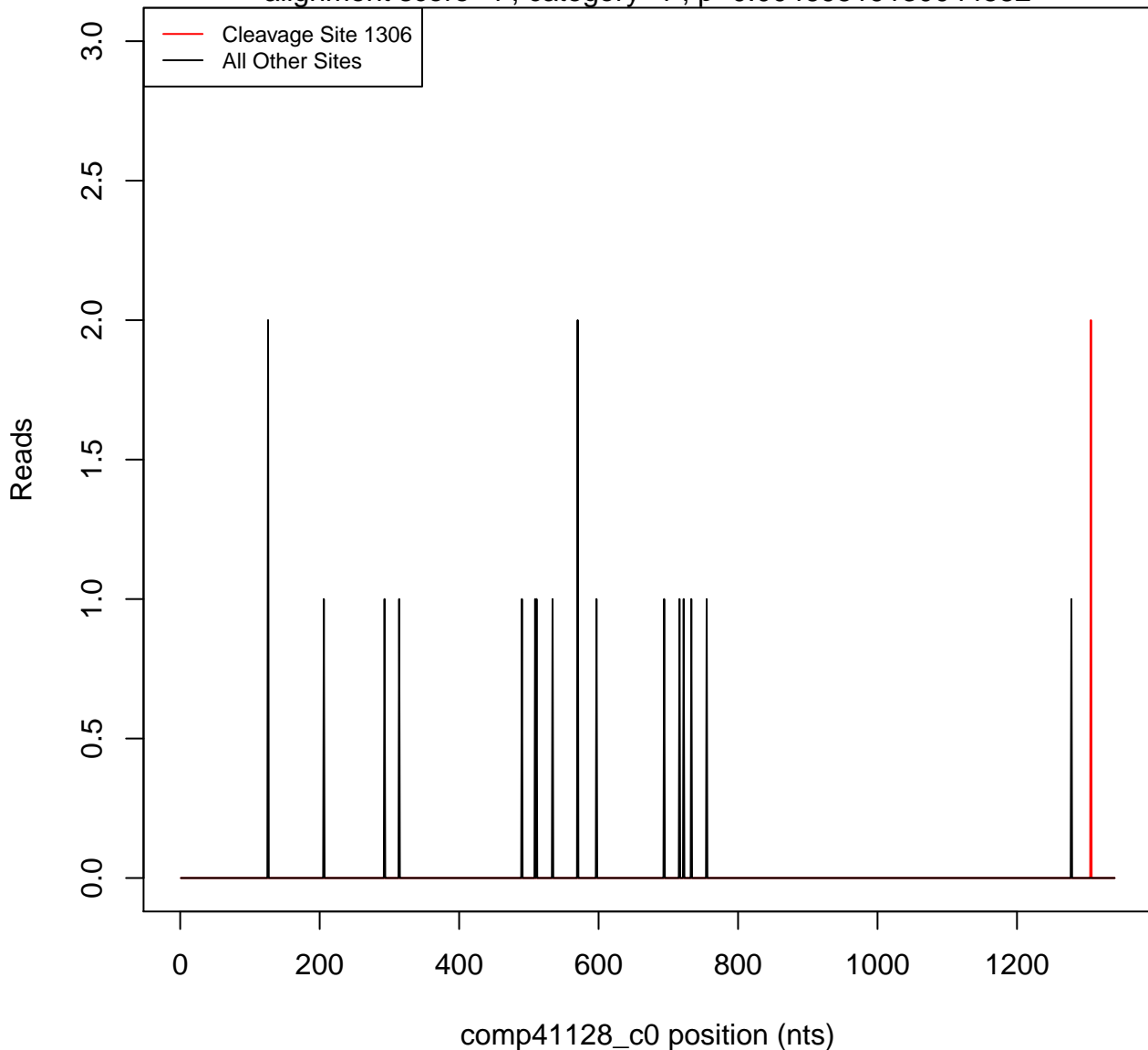

Supplement: S8 File — (ZIP) [file pone.0186500.s014.zip › S8 t-plot of miRNA-target/comp41128_c0--1306--ath-miR169h_1ss21GA_degradome.pdf]

# ath-miR169h\_R-1 slicing comp41128\_c0 at nt 1306

alignment score=3 , category=1 , p=0.00903527047130315

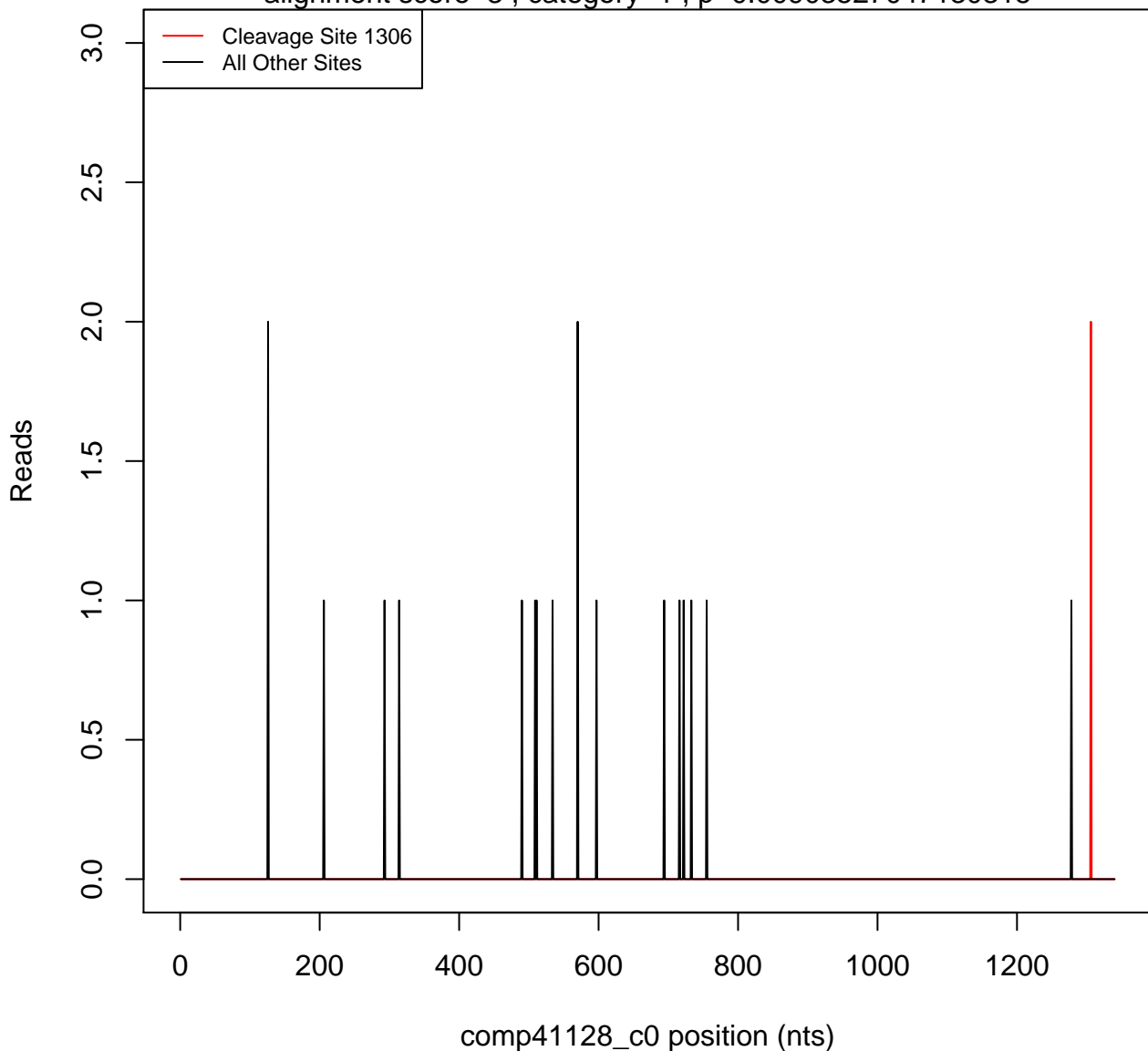

Supplement: S8 File — (ZIP) [file pone.0186500.s014.zip › S8 t-plot of miRNA-target/comp41128_c0--1306--ath-miR169h_R-1_degradome.pdf]

# ath-miR169h\_R-3 slicing comp41128\_c0 at nt 1306

alignment score=2 , category=1 , p=0.0146822157073718

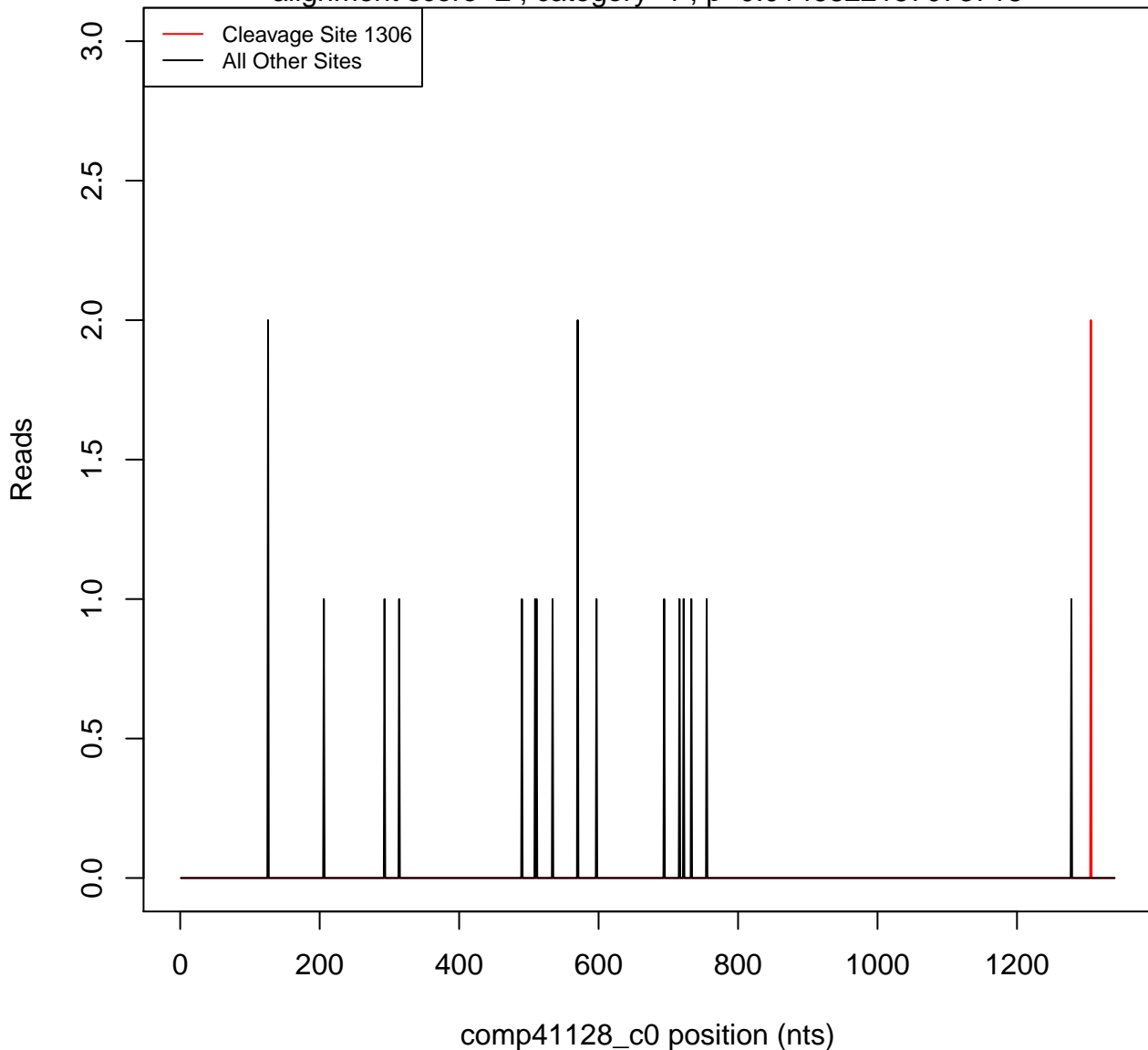

Supplement: S8 File — (ZIP) [file pone.0186500.s014.zip › S8 t-plot of miRNA-target/comp41128_c0--1306--ath-miR169h_R-3_degradome.pdf]

# ath-miR169h slicing comp41128\_c0 at nt 1306

alignment score=4 , category=1 , p=0.00703451366220564

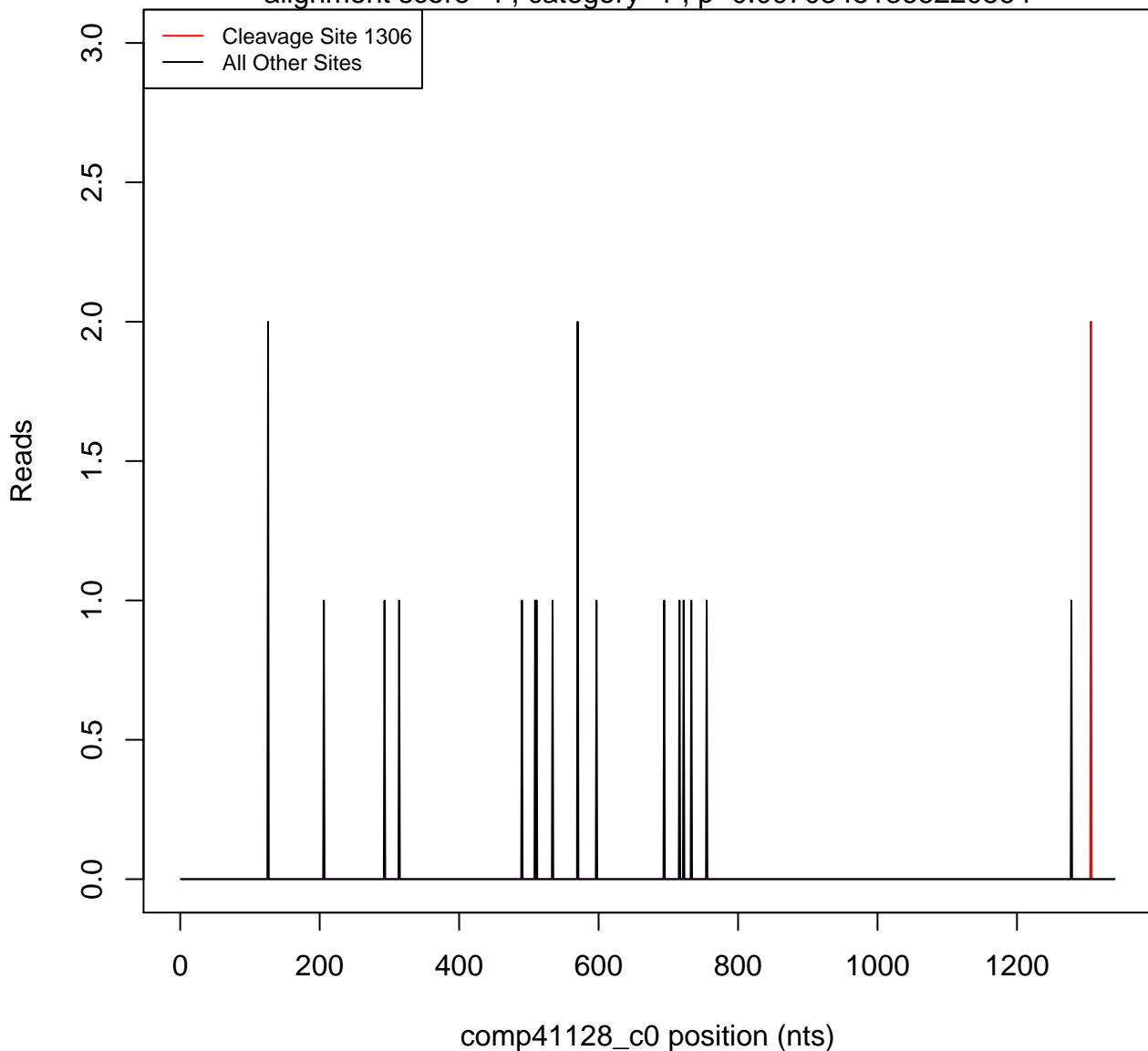

Supplement: S8 File — (ZIP) [file pone.0186500.s014.zip › S8 t-plot of miRNA-target/comp41128_c0--1306--ath-miR169h_degradome.pdf]

# gma-miR169a\_R+1\_1ss21GT slicing comp41128\_c0 at nt 1306

alignment score=4 , category=1 , p=0.00469519130044582

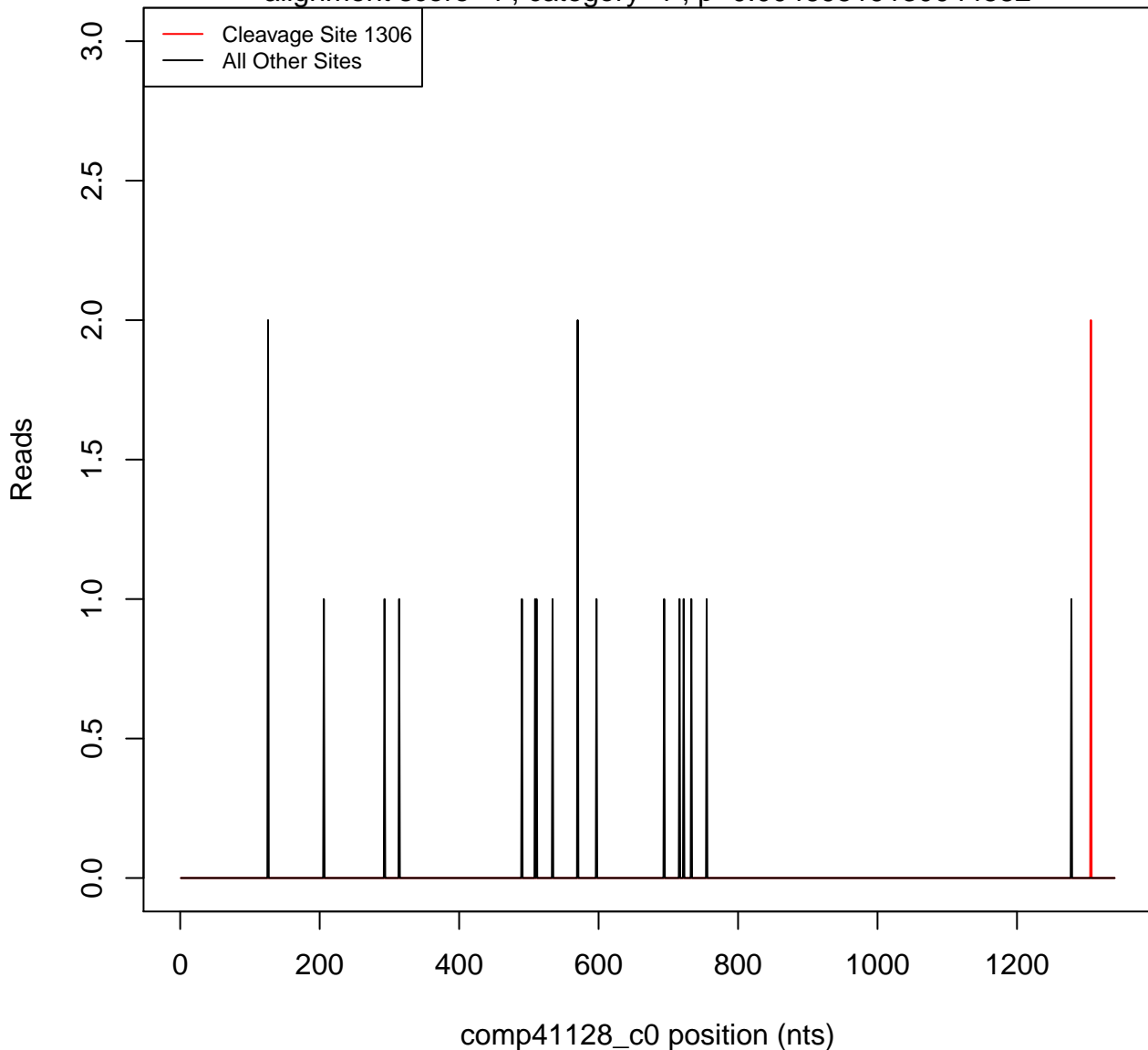

Supplement: S8 File — (ZIP) [file pone.0186500.s014.zip › S8 t-plot of miRNA-target/comp41128_c0--1306--gma-miR169a_R+1_1ss21GT_degradome.pdf]

**ath-miR157a\_R+1\_1ss21CT slicing comp41255\_c0 at nt 1063**

alignment score=4 , category=0 , p=0.00384698163572694

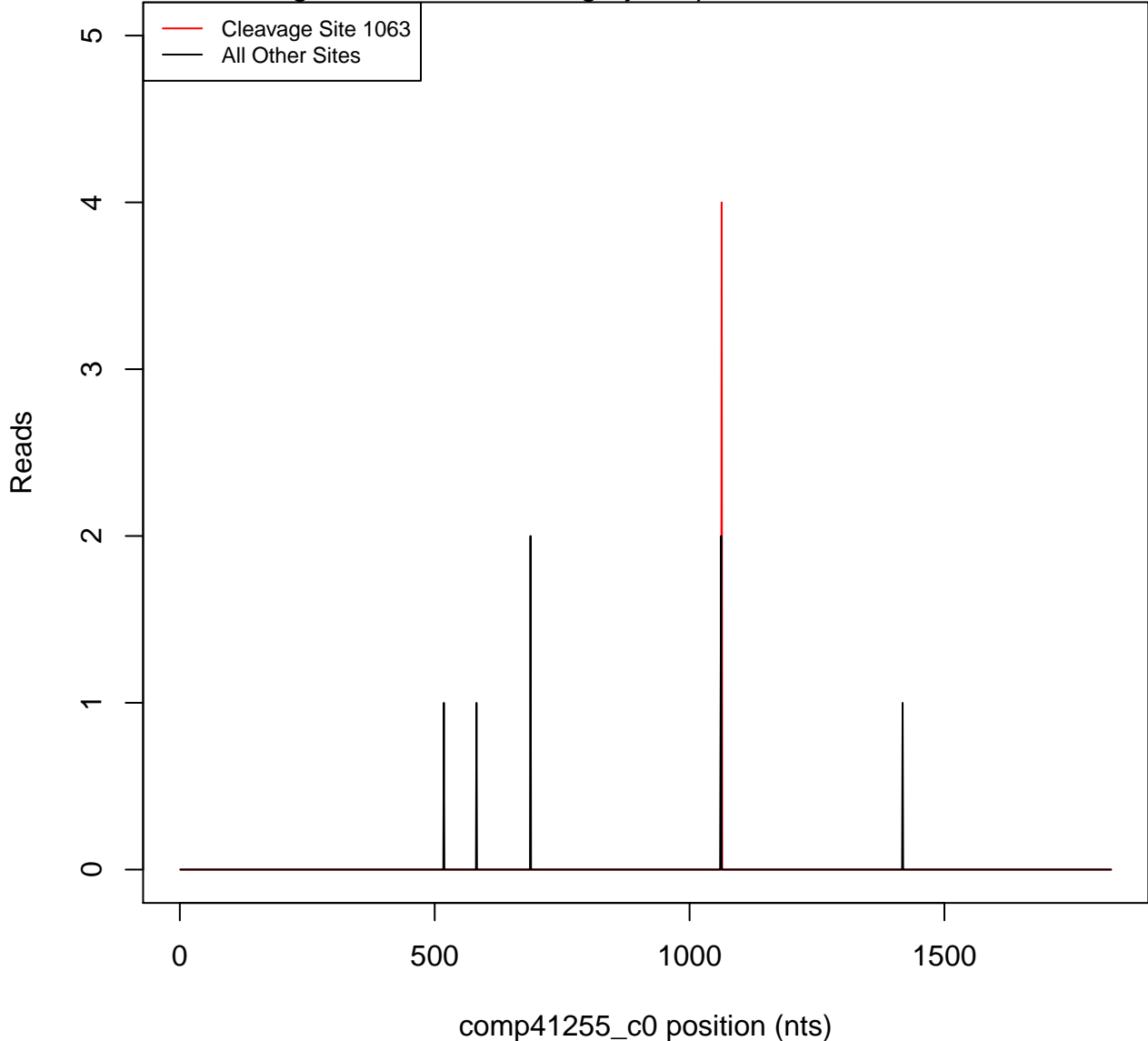

Supplement: S8 File — (ZIP) [file pone.0186500.s014.zip › S8 t-plot of miRNA-target/comp41255_c0--1063--ath-miR157a_R+1_1ss21CT_degradome.pdf]

**ath-miR157d\_L+1R-1 slicing comp41255\_c0 at nt 1063**

alignment score=2 , category=0 , p=0.00603860863029393

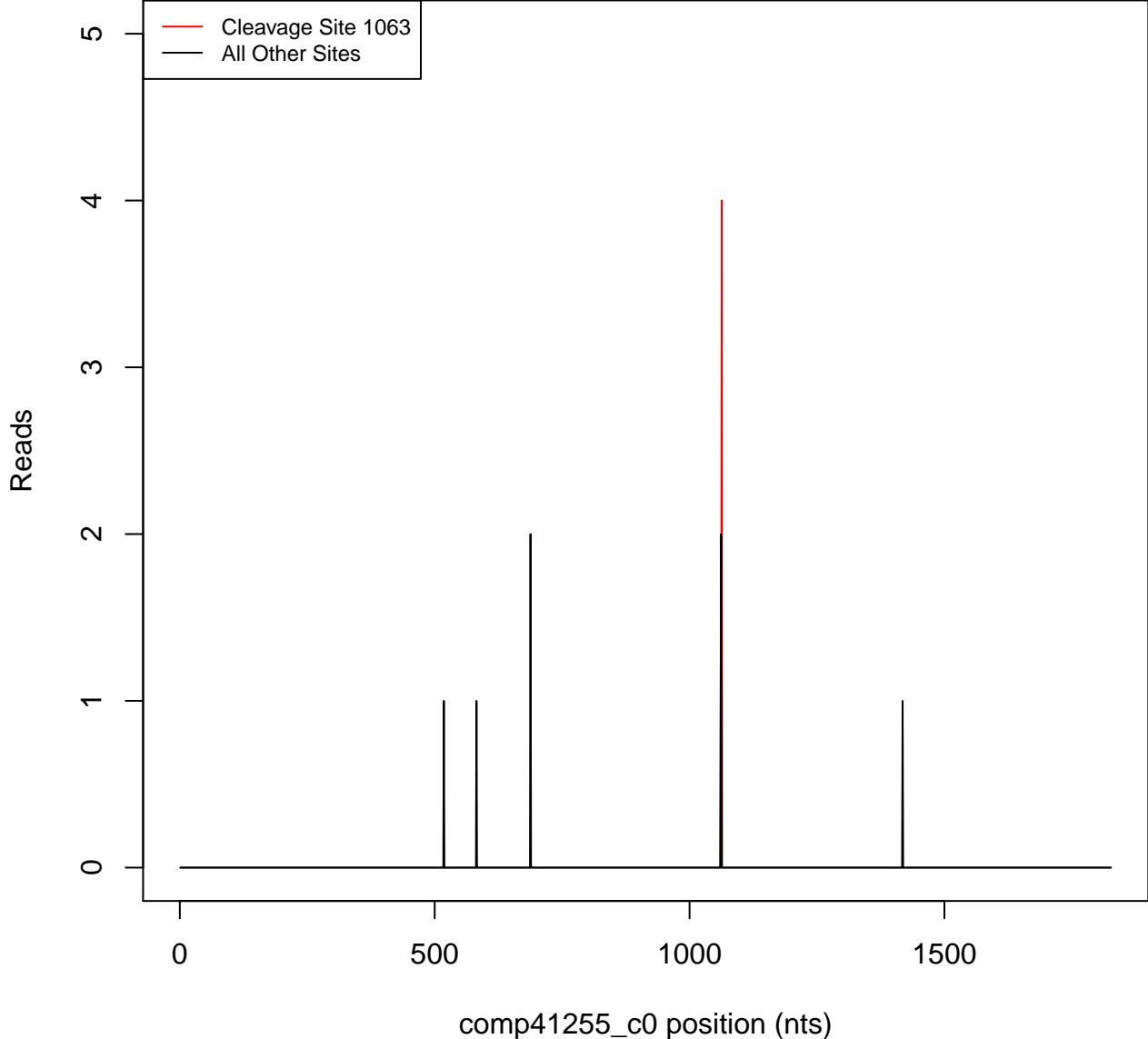

Supplement: S8 File — (ZIP) [file pone.0186500.s014.zip › S8 t-plot of miRNA-target/comp41255_c0--1063--ath-miR157d_L+1R-1_degradome.pdf]

# ath-miR157d\_L+1 slicing comp41255\_c0 at nt 1063

alignment score=2.5 , category=0 , p=0.00274935665012399

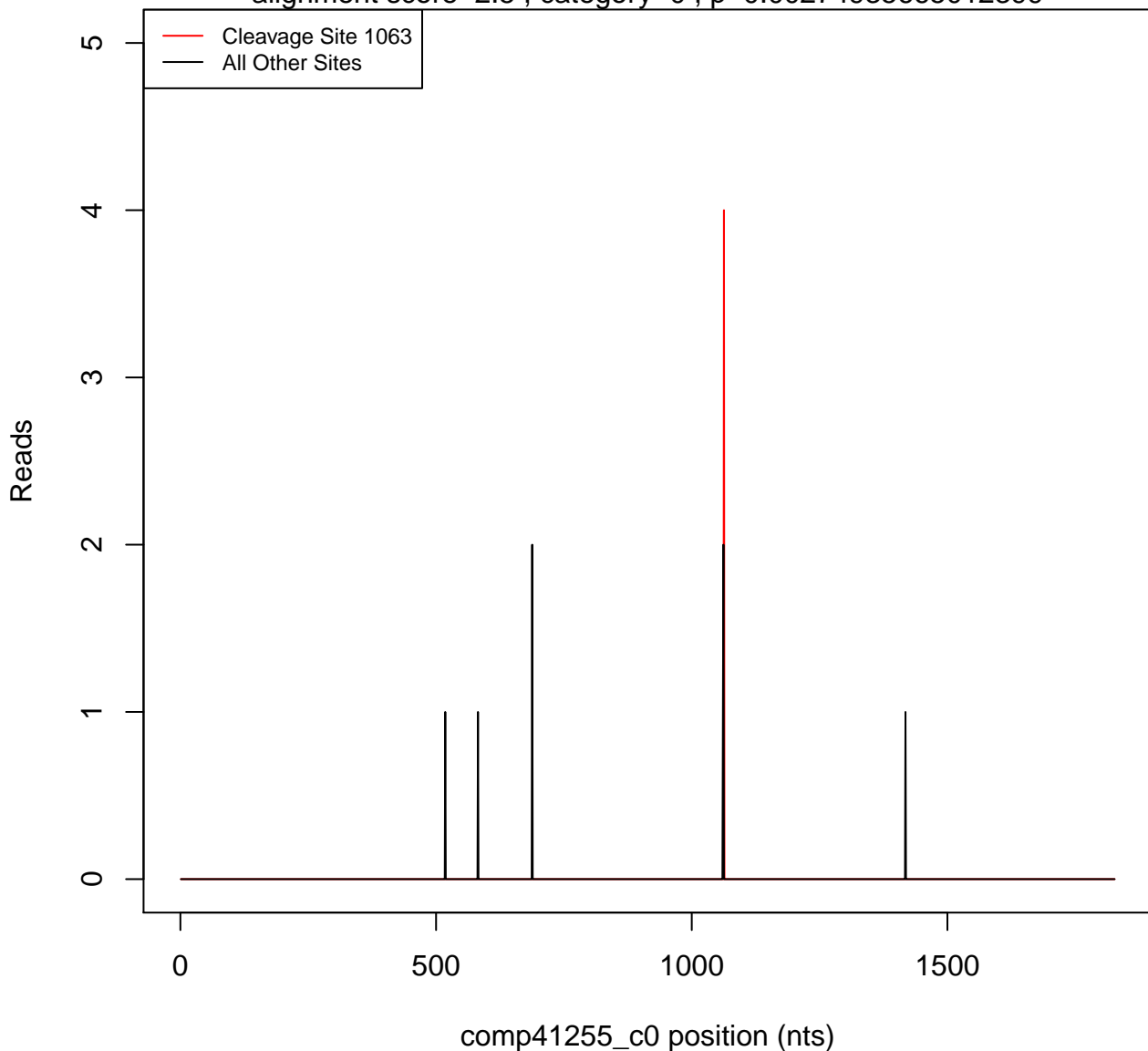

Supplement: S8 File — (ZIP) [file pone.0186500.s014.zip › S8 t-plot of miRNA-target/comp41255_c0--1063--ath-miR157d_L+1_degradome.pdf]

# ath-miR396b slicing comp41306\_c0 at nt 949

alignment score=4 , category=1 , p=0.0232565409076966

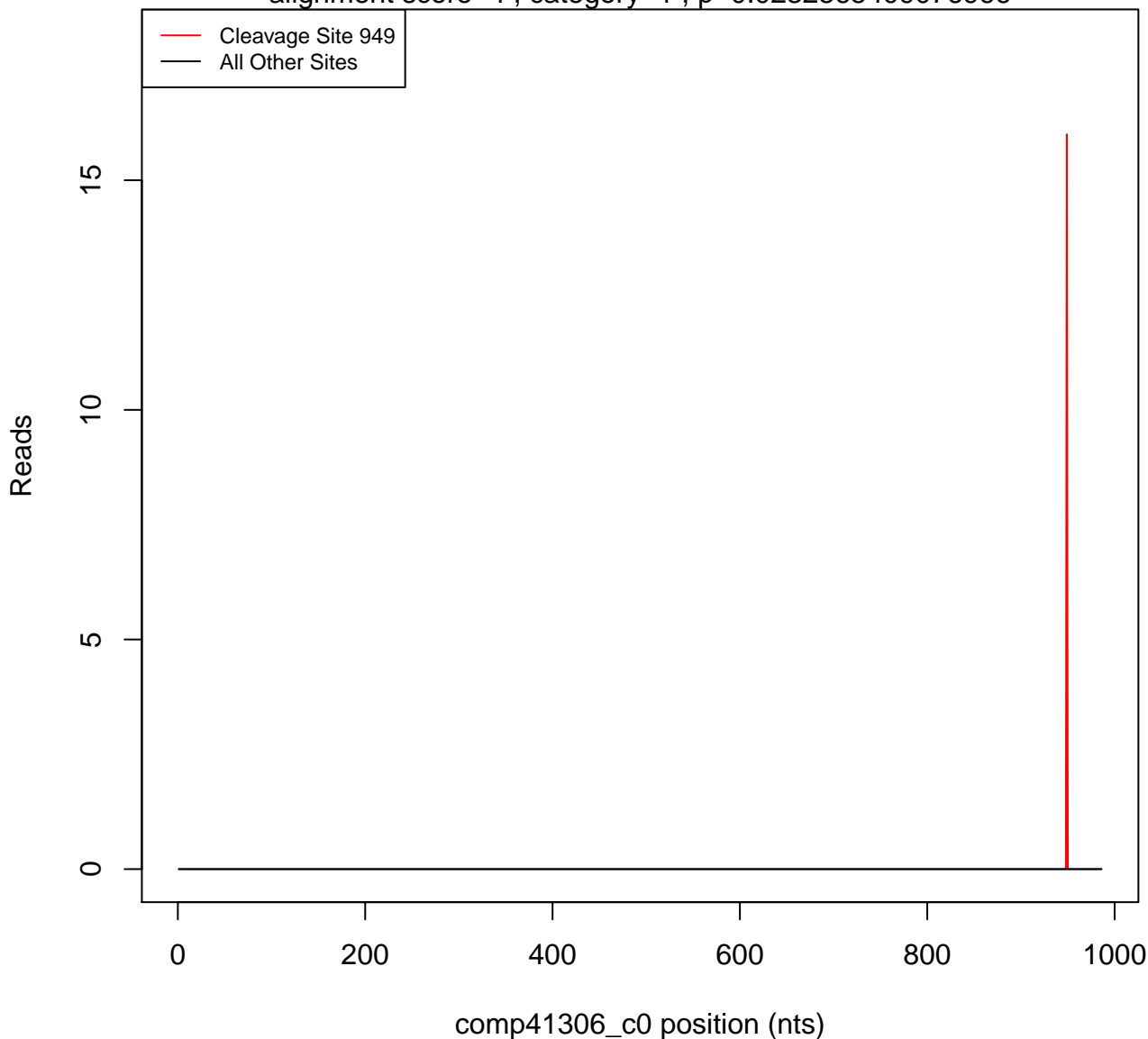

Supplement: S8 File — (ZIP) [file pone.0186500.s014.zip › S8 t-plot of miRNA-target/comp41306_c0--949--ath-miR396b_degradome.pdf]

# bdi-MIR5164-p5\_1ss1GA slicing comp421505\_c0 at nt 90

alignment score=2 , category=1 , p=0.0916649133196143

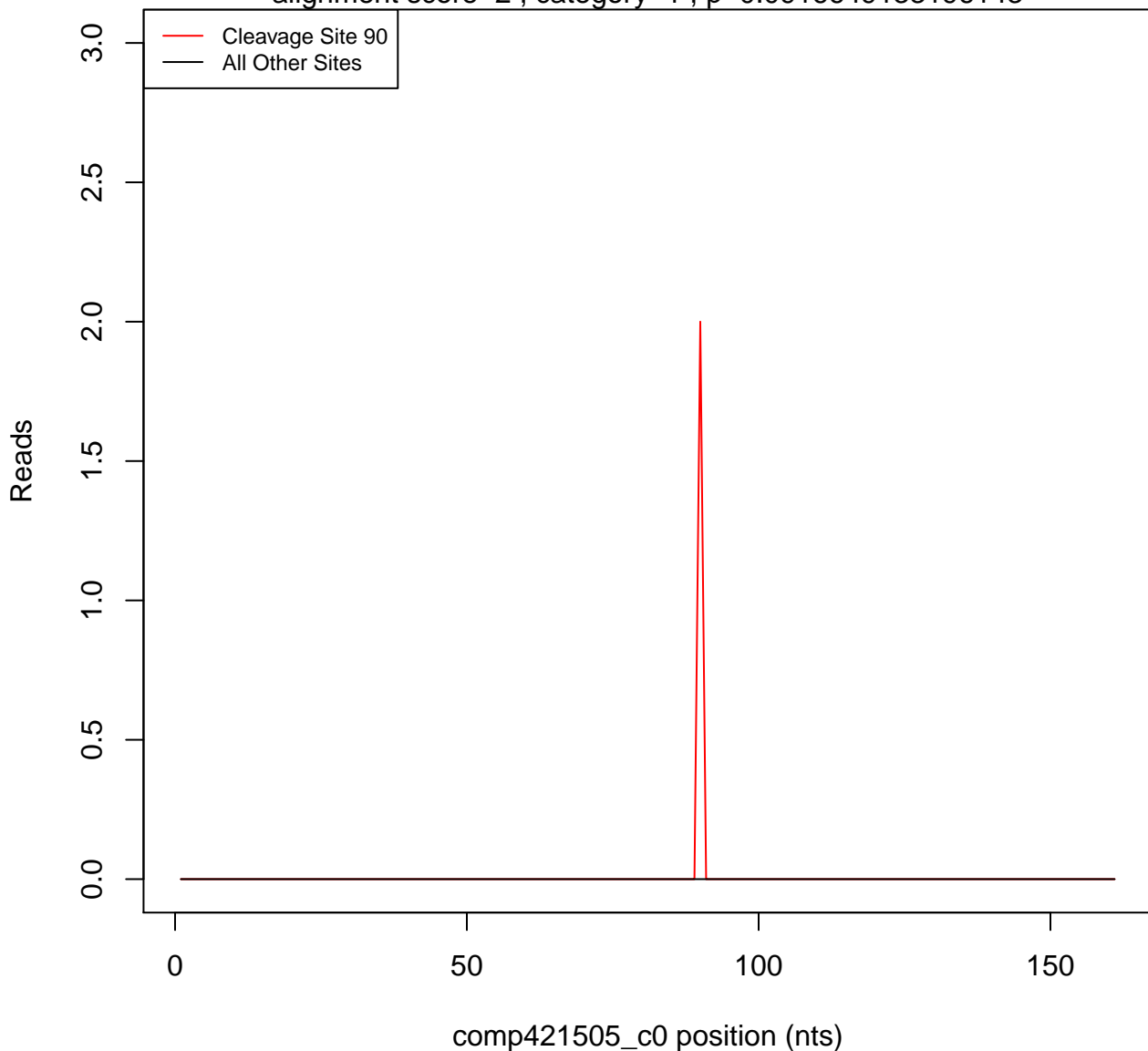

Supplement: S8 File — (ZIP) [file pone.0186500.s014.zip › S8 t-plot of miRNA-target/comp421505_c0--90--bdi-MIR5164-p5_1ss1GA_degradome.pdf]

# osa-MIR1436-p3\_1ss20CA slicing comp44948\_c2 at nt 846

alignment score=3.5 , category=3 , p=0.0258883479330546

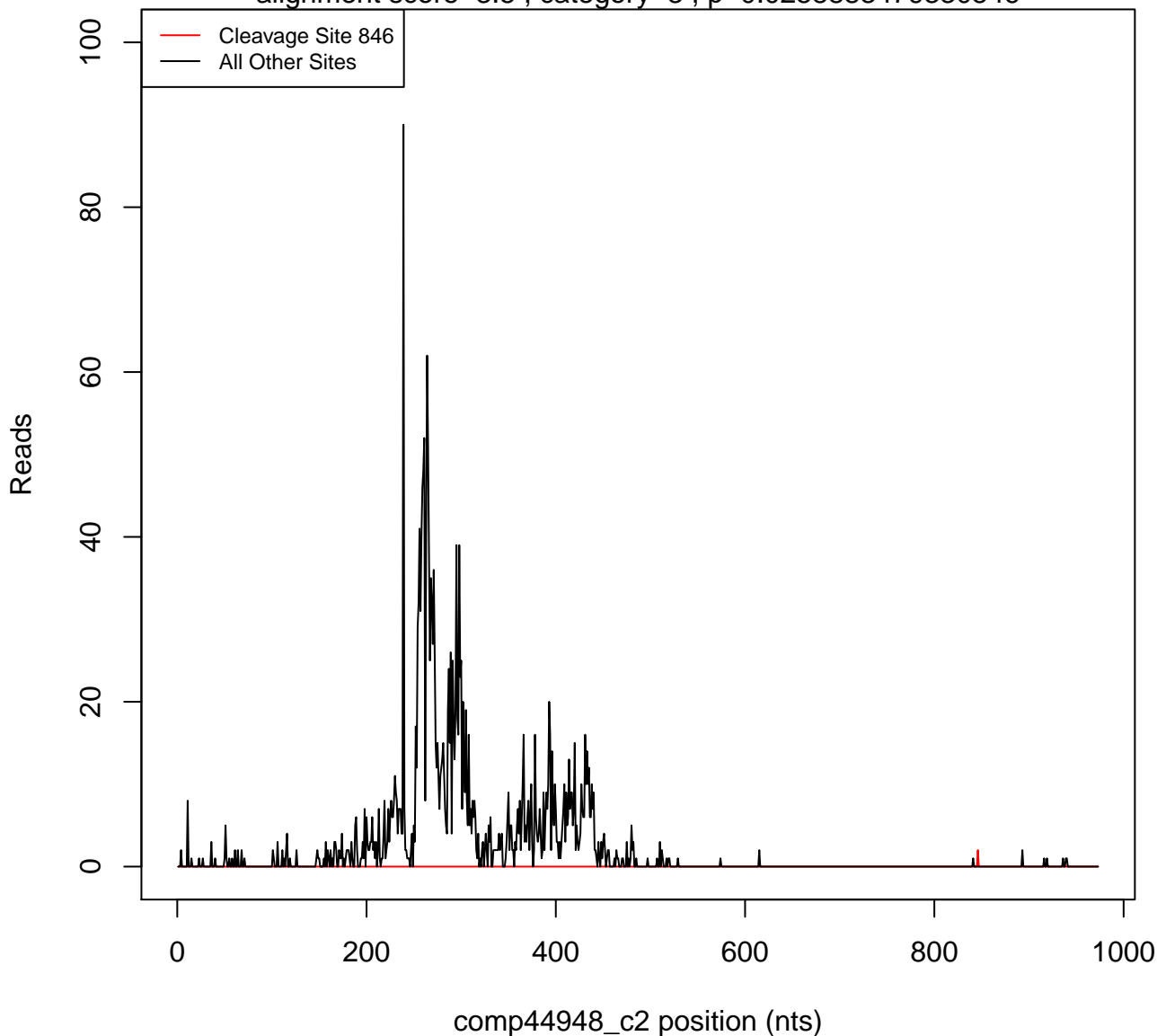

Supplement: S8 File — (ZIP) [file pone.0186500.s014.zip › S8 t-plot of miRNA-target/comp44948_c2--846--osa-MIR1436-p3_1ss20CA_degradome.pdf]

# ath-miR398a\_1ss15AG slicing comp455853\_c0 at nt 26

alignment score=4 , category=3 , p=0.00572124118747341

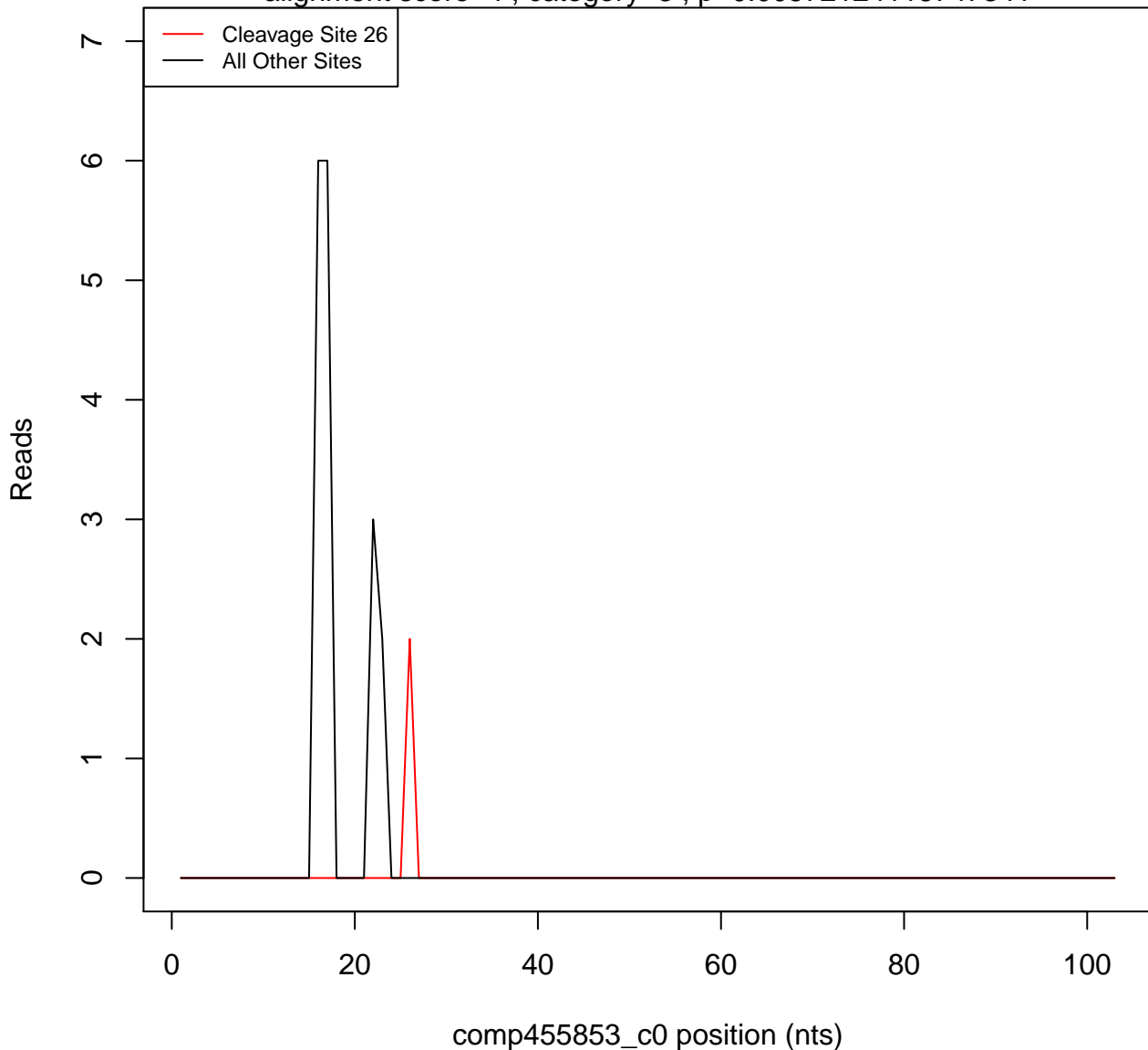

Supplement: S8 File — (ZIP) [file pone.0186500.s014.zip › S8 t-plot of miRNA-target/comp455853_c0--26--ath-miR398a_1ss15AG_degradome.pdf]

# ath-miR398b\_1ss15AG slicing comp455853\_c0 at nt 26

alignment score=3 , category=3 , p=0.00368170809051804

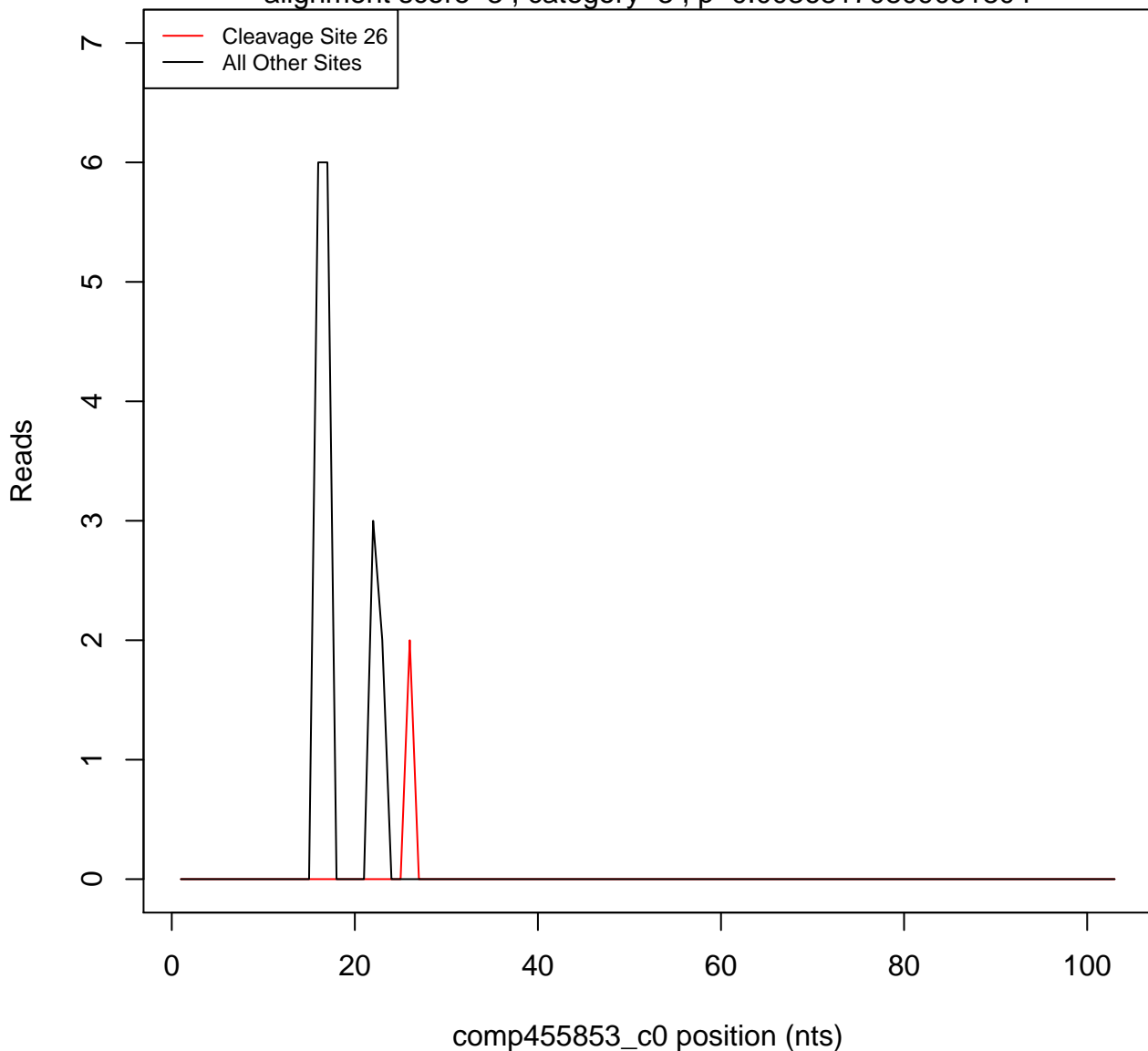

Supplement: S8 File — (ZIP) [file pone.0186500.s014.zip › S8 t-plot of miRNA-target/comp455853_c0--26--ath-miR398b_1ss15AG_degradome.pdf]

# ath-miR169b slicing comp45596\_c1 at nt 1044

alignment score=3.5 , category=1 , p=0.00268567187579372

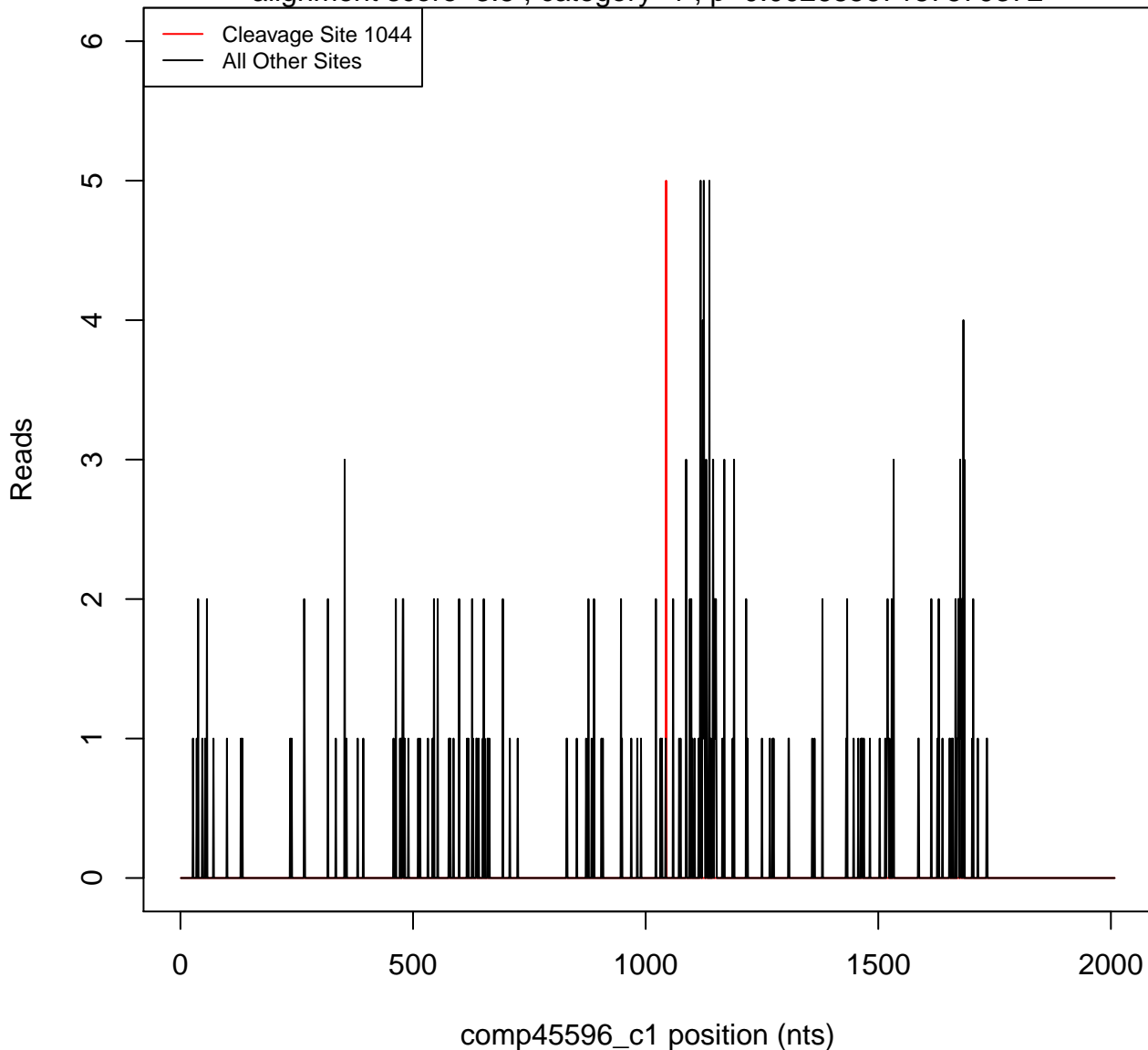

Supplement: S8 File — (ZIP) [file pone.0186500.s014.zip › S8 t-plot of miRNA-target/comp45596_c1--1044--ath-miR169b_degradome.pdf]

# ath-miR169h\_R-3 slicing comp45596\_c1 at nt 1044

alignment score=3.5 , category=1 , p=0.0239130055308171

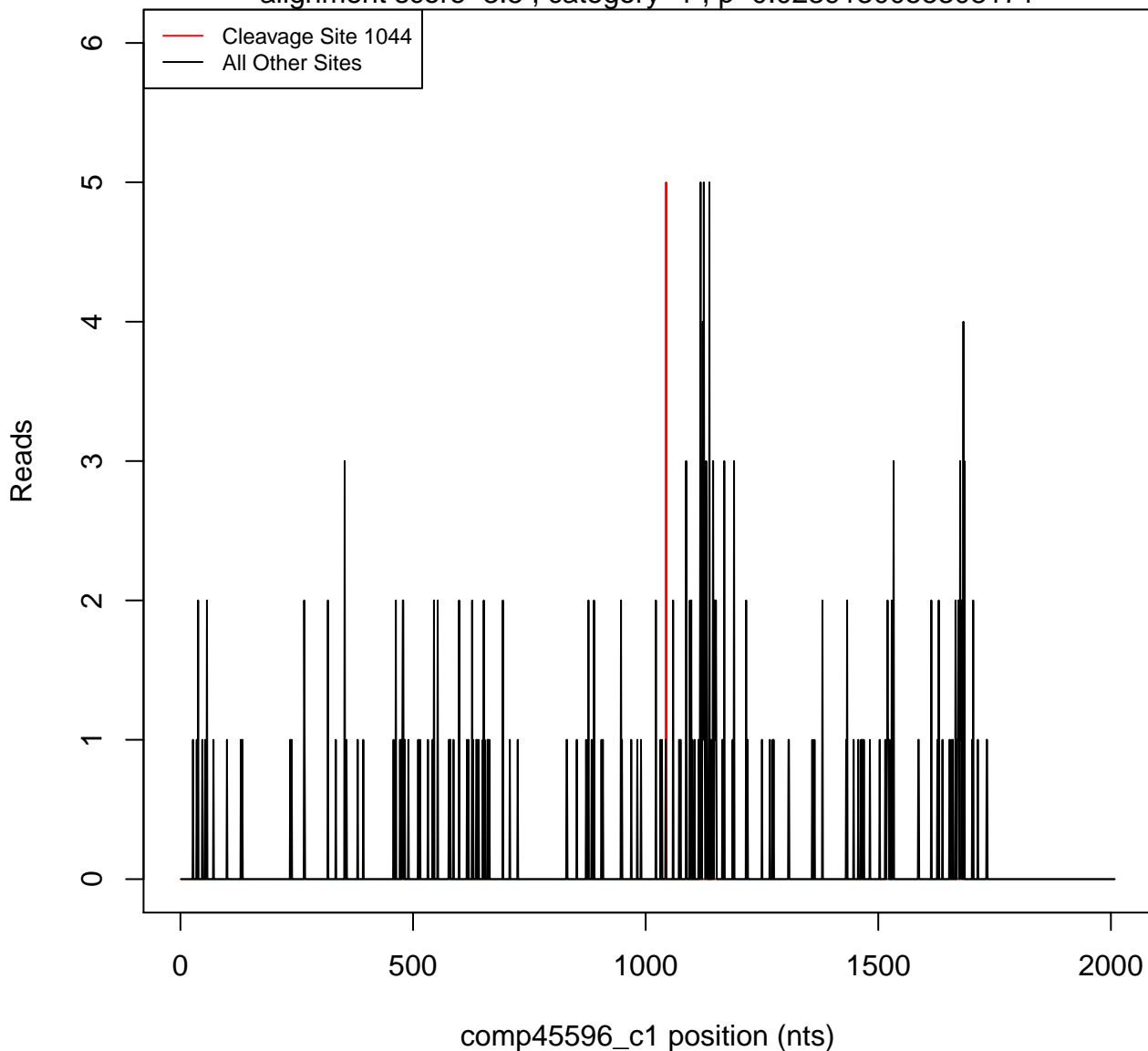

Supplement: S8 File — (ZIP) [file pone.0186500.s014.zip › S8 t-plot of miRNA-target/comp45596_c1--1044--ath-miR169h_R-3_degradome.pdf]

# gma-miR169a\_R+1\_1ss21GT slicing comp45596\_c1 at nt 1044

alignment score=4 , category=1 , p=0.00469519130044582

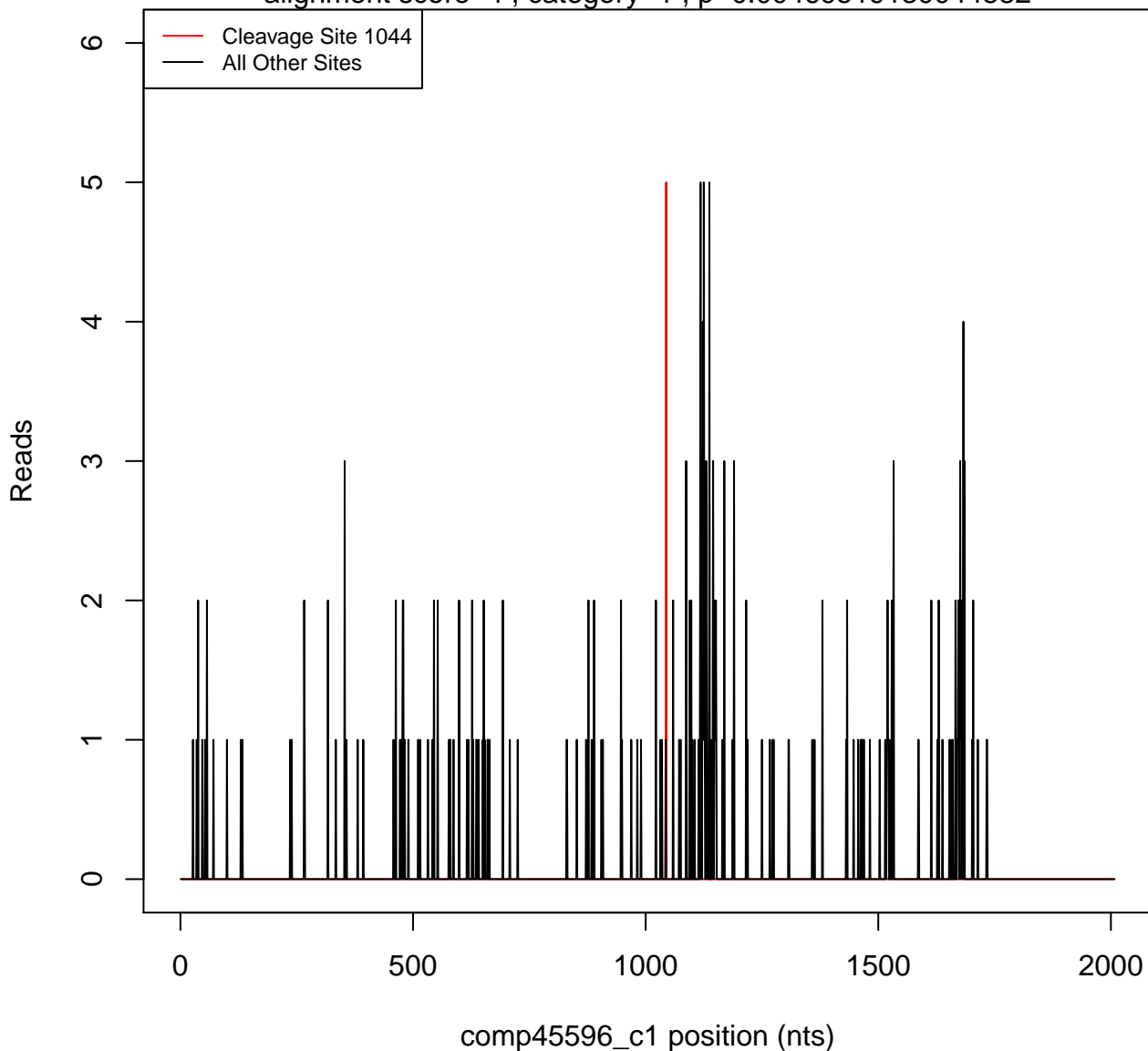

Supplement: S8 File — (ZIP) [file pone.0186500.s014.zip › S8 t-plot of miRNA-target/comp45596_c1--1044--gma-miR169a_R+1_1ss21GT_degradome.pdf]

# ath-miR393a slicing comp45777\_c0 at nt 1593

alignment score=2 , category=0 , p=0.00302387622887079

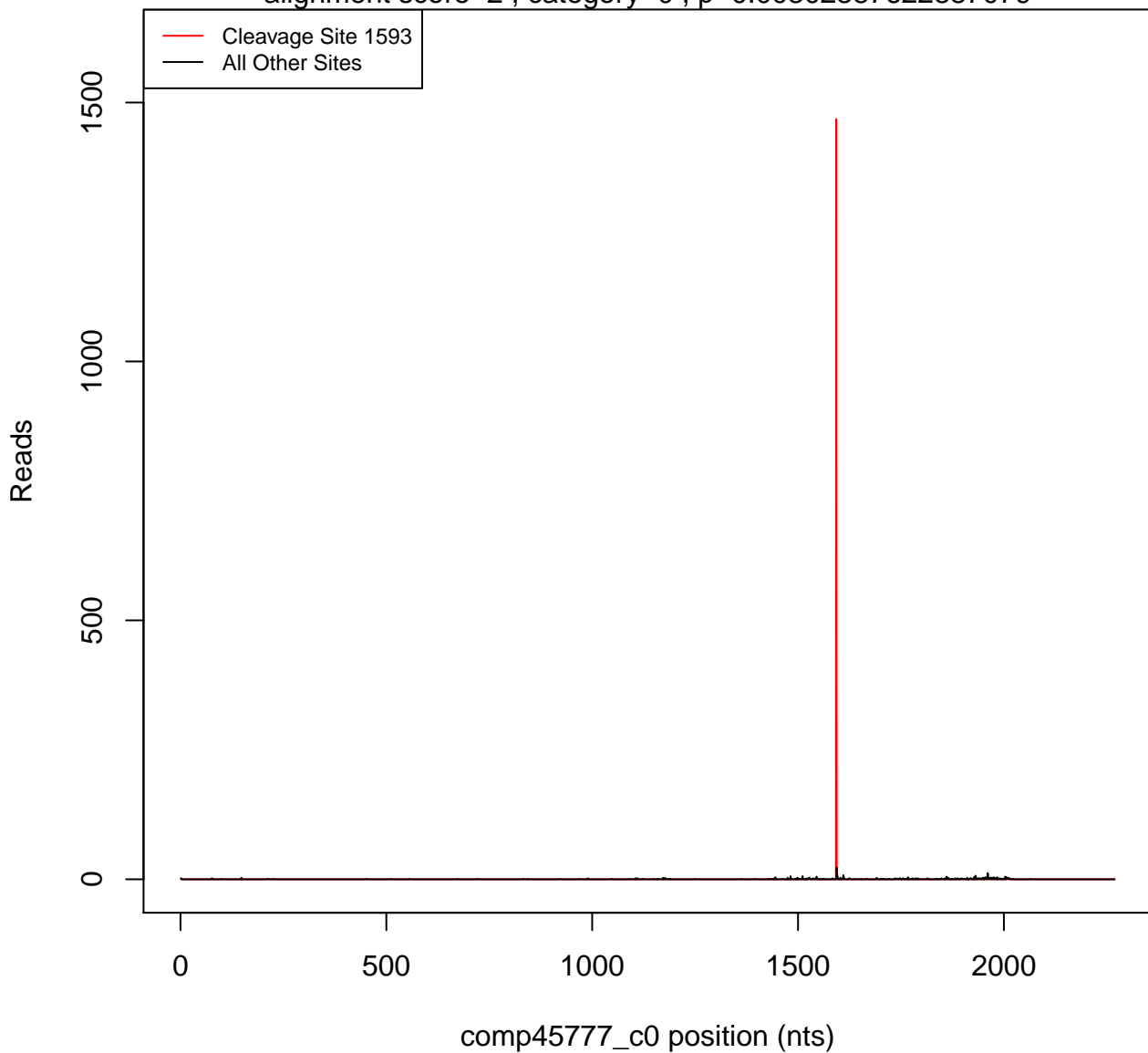

Supplement: S8 File — (ZIP) [file pone.0186500.s014.zip › S8 t-plot of miRNA-target/comp45777_c0--1593--ath-miR393a_degradome.pdf]

# gma-miR396a-5p\_R+2 slicing comp46504\_c0 at nt 848

alignment score=3.5 , category=2 , p=0.0572383778530186

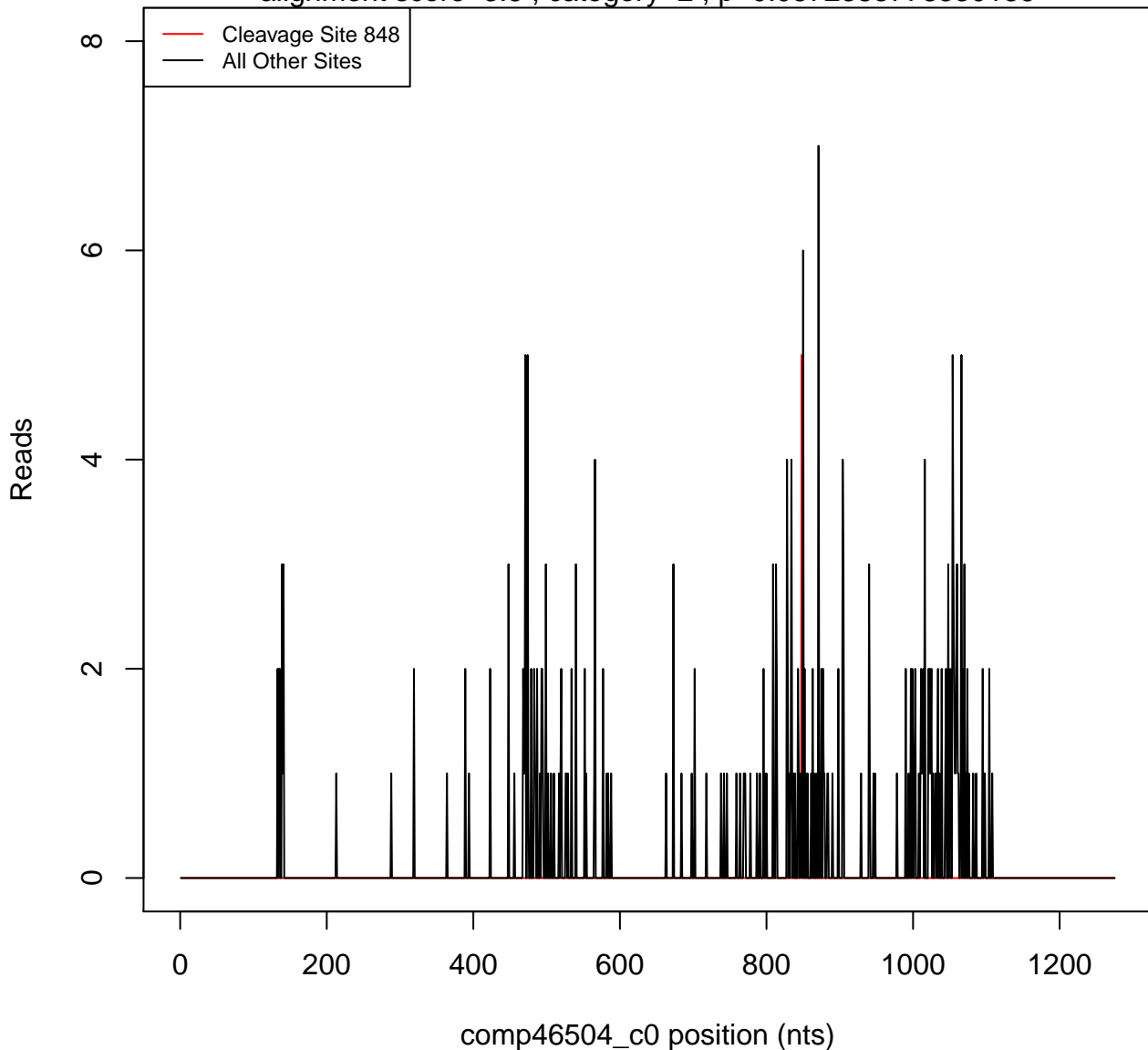

Supplement: S8 File — (ZIP) [file pone.0186500.s014.zip › S8 t-plot of miRNA-target/comp46504_c0--848--gma-miR396a-5p_R+2_degradome.pdf]

# osa-MIR812a-p3\_1ss2CT slicing comp46699\_c0 at nt 1573

alignment score=3 , category=3 , p=0.0110045092536222

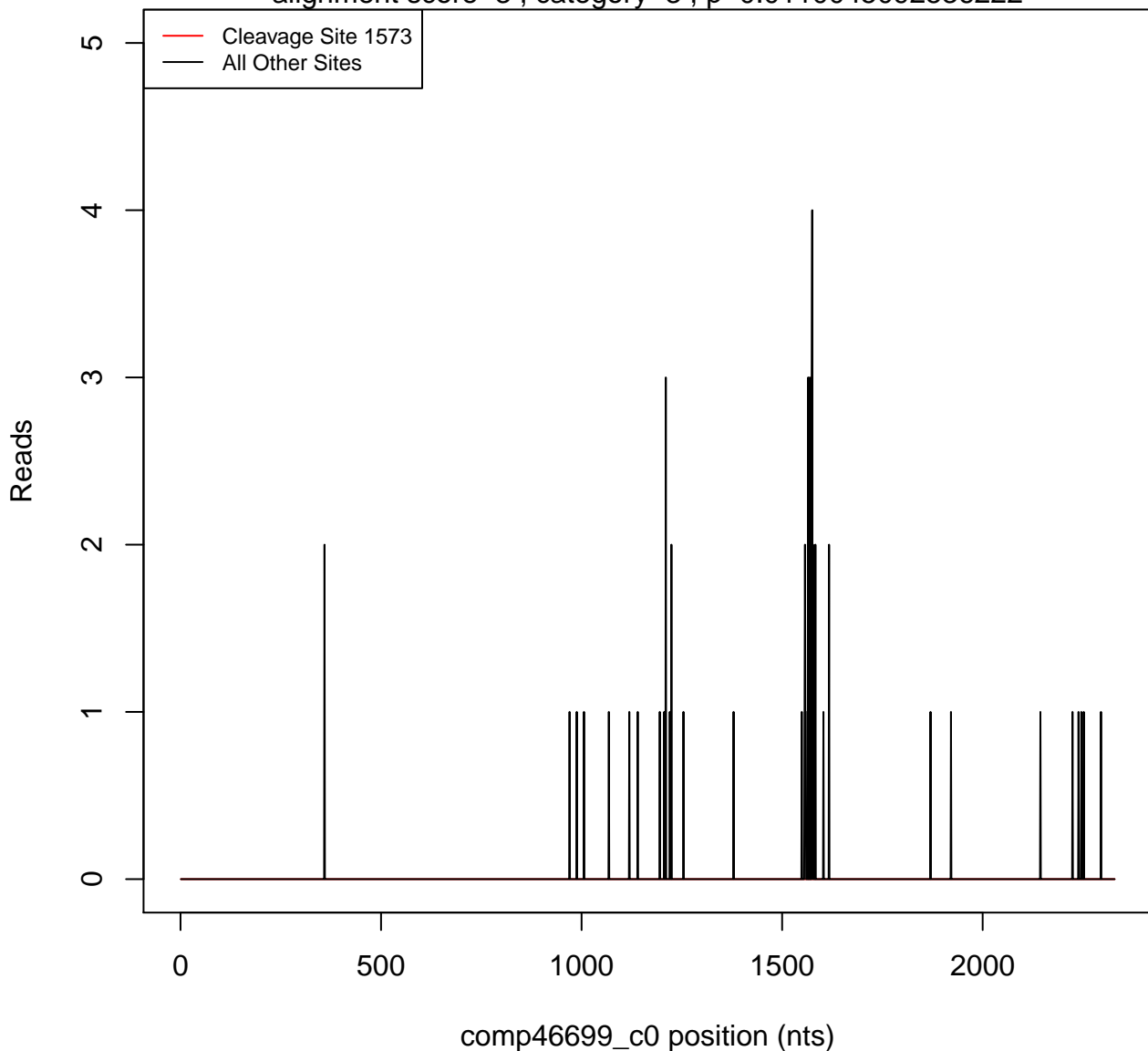

Supplement: S8 File — (ZIP) [file pone.0186500.s014.zip › S8 t-plot of miRNA-target/comp46699_c0--1573--osa-MIR812a-p3_1ss2CT_degradome.pdf]

**ath-miR157d\_L+1R-1 slicing comp46911\_c1 at nt 1183**

alignment score=3 , category=0 , p=0.0171952462084104

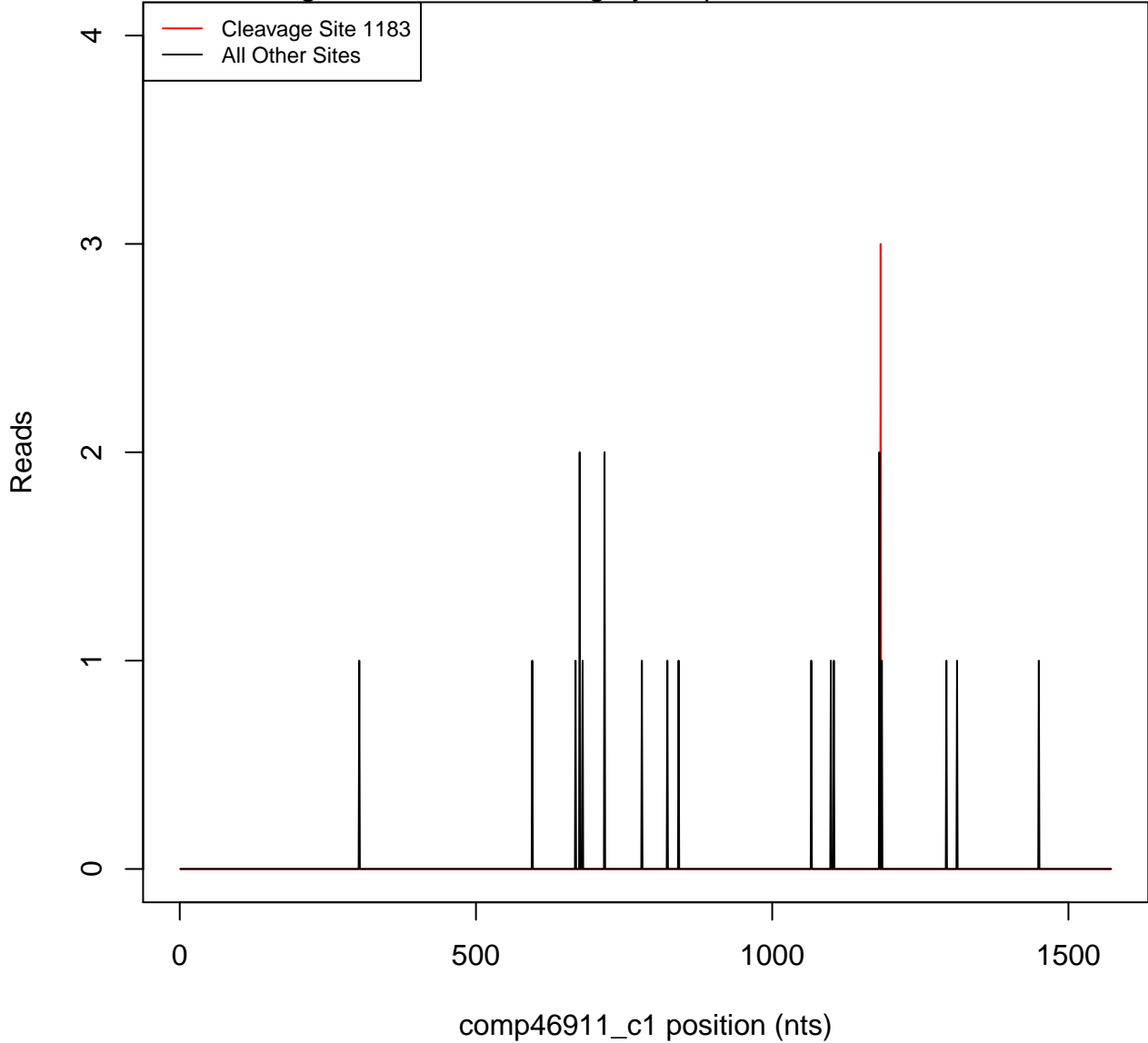

Supplement: S8 File — (ZIP) [file pone.0186500.s014.zip › S8 t-plot of miRNA-target/comp46911_c1--1183--ath-miR157d_L+1R-1_degradome.pdf]

# ath-miR157d\_L+1 slicing comp46911\_c1 at nt 1183

alignment score=3 , category=0 , p=0.00494339851921732

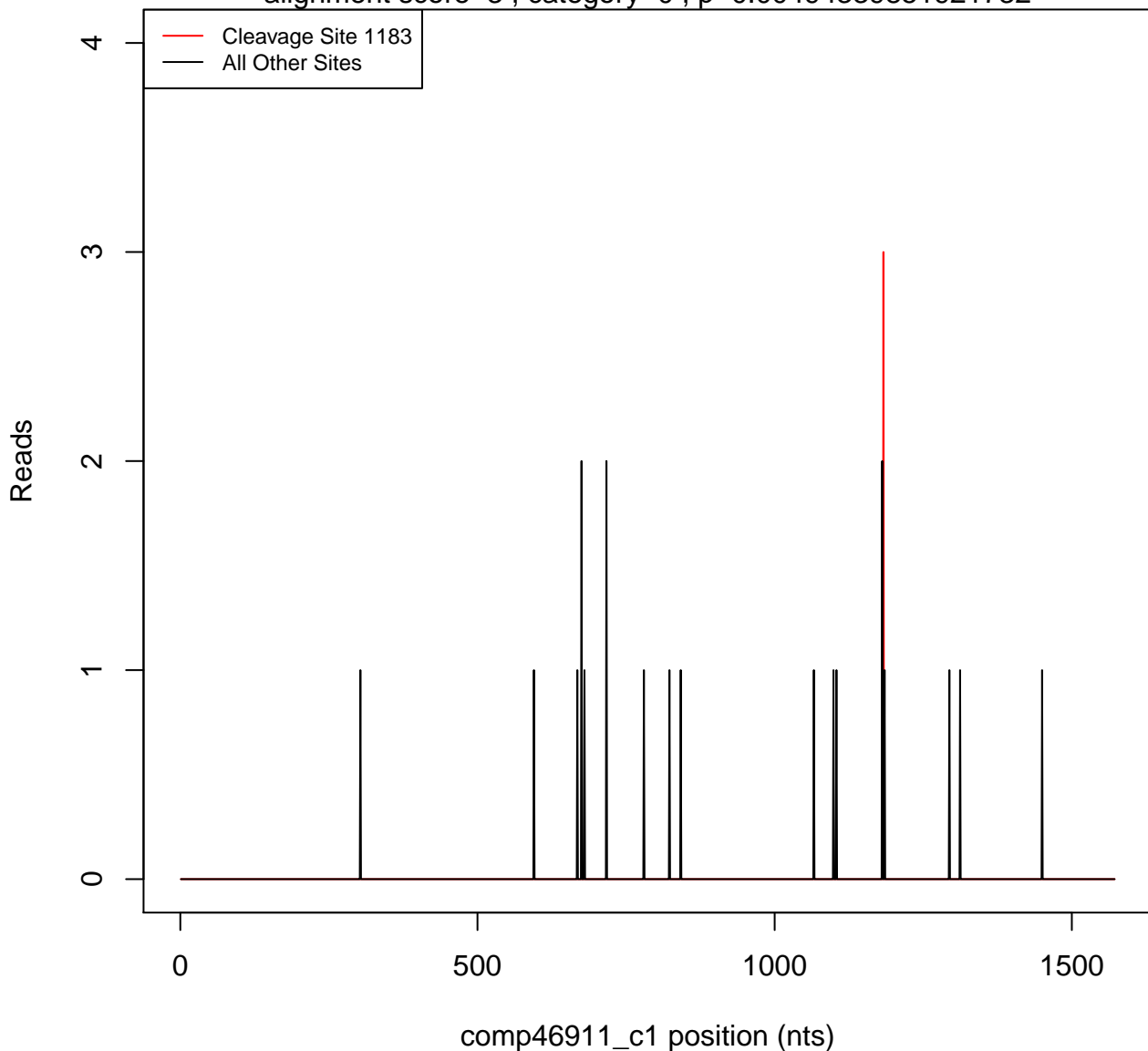

Supplement: S8 File — (ZIP) [file pone.0186500.s014.zip › S8 t-plot of miRNA-target/comp46911_c1--1183--ath-miR157d_L+1_degradome.pdf]

# bcy-miR156\_L-1\_2ss13TG16AT slicing comp46911\_c1 at nt 1183

alignment score=2 , category=0 , p=0.00603860863029393

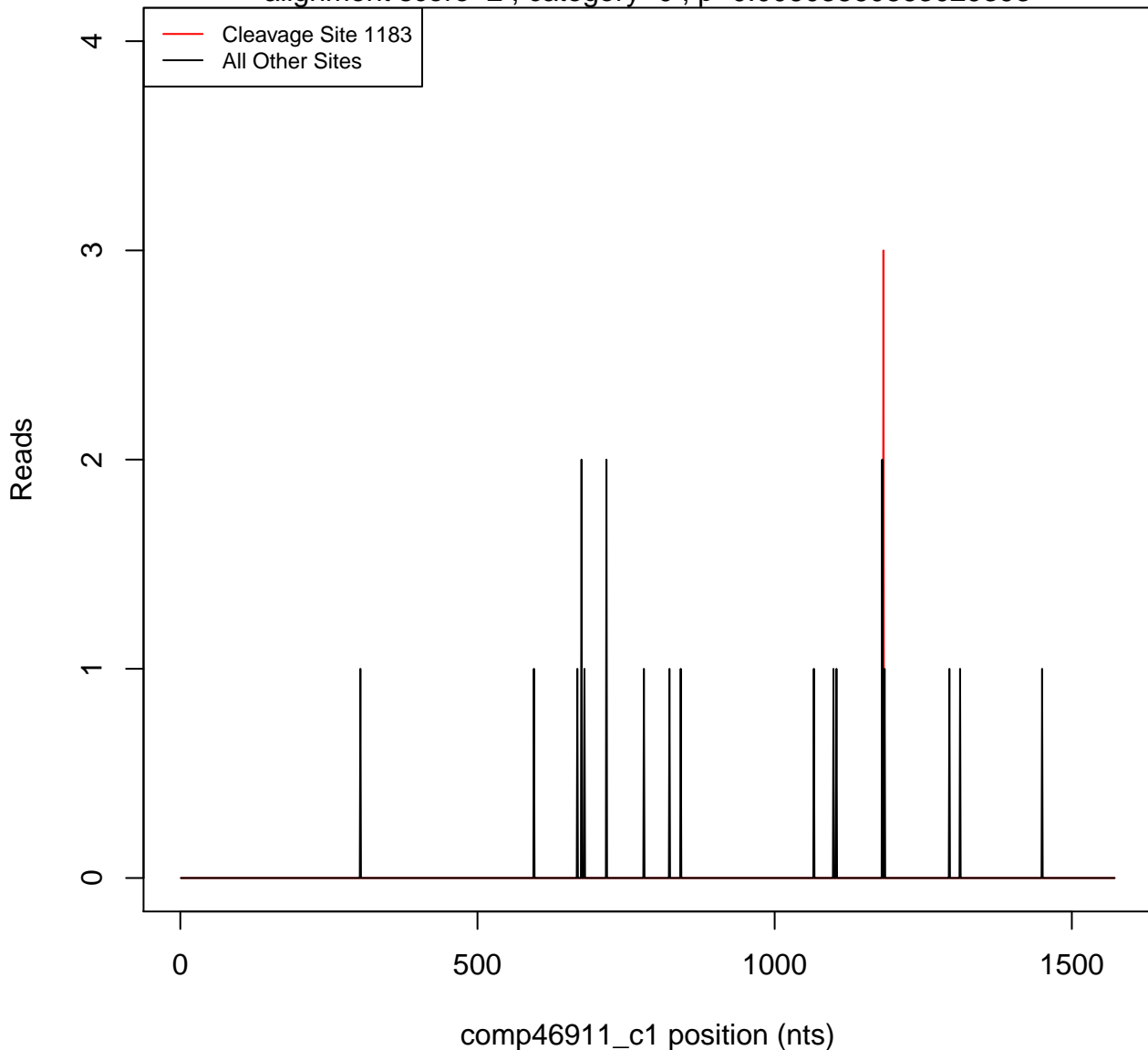

Supplement: S8 File — (ZIP) [file pone.0186500.s014.zip › S8 t-plot of miRNA-target/comp46911_c1--1183--bcy-miR156_L-1_2ss13TG16AT_degradome.pdf]

**mdm-miR156t slicing comp46911\_c1 at nt 1183**

alignment score=1 , category=0 , p=0.00357268870087279

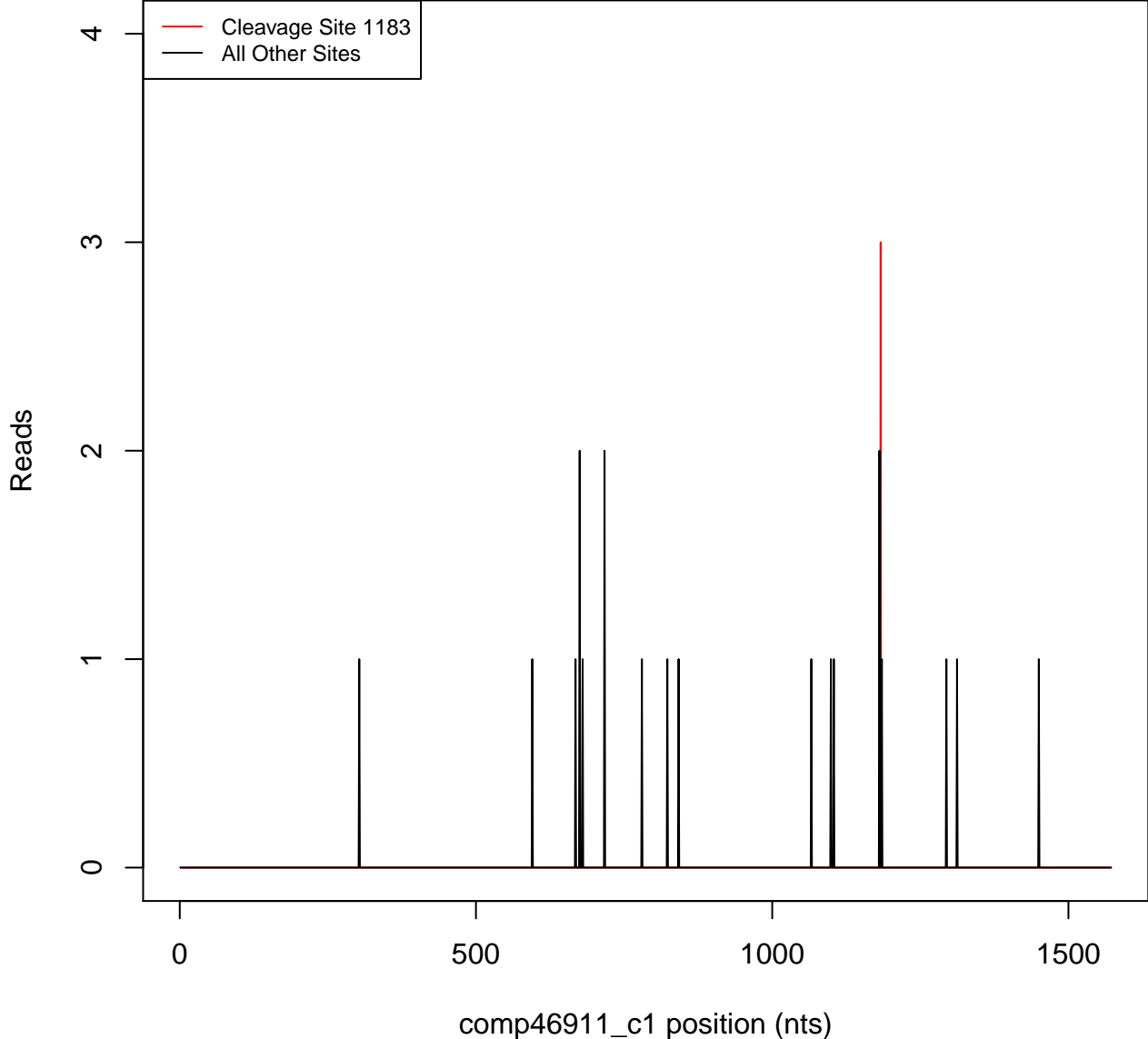

Supplement: S8 File — (ZIP) [file pone.0186500.s014.zip › S8 t-plot of miRNA-target/comp46911_c1--1183--mdm-miR156t_degradome.pdf]

# ath-miR160a\_1ss21AT slicing comp48868\_c0 at nt 2279

alignment score=1.5 , category=0 , p=0.0032983202388529

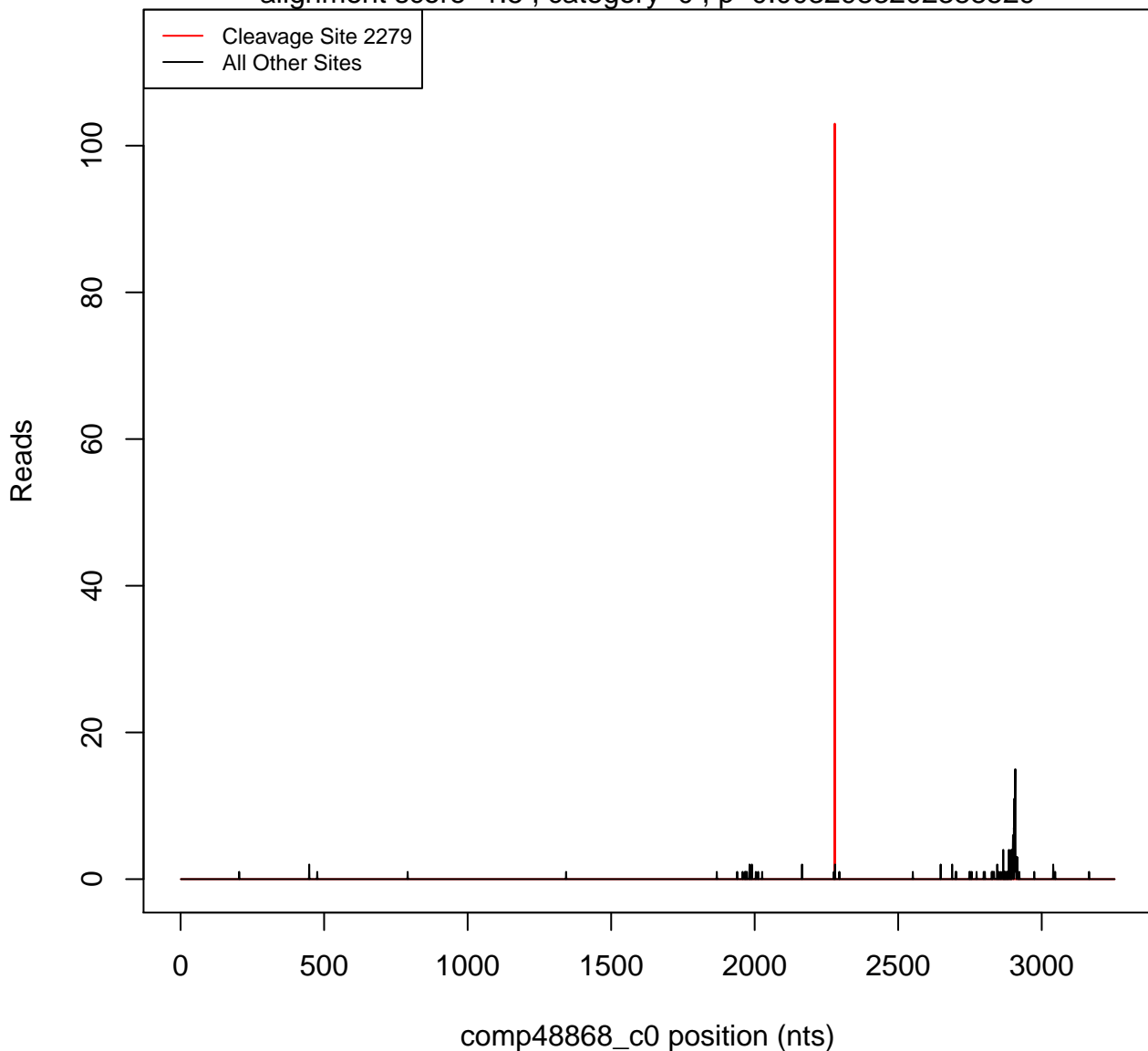

Supplement: S8 File — (ZIP) [file pone.0186500.s014.zip › S8 t-plot of miRNA-target/comp48868_c0--2279--ath-miR160a_1ss21AT_degradome.pdf]

# mdm-miR160a slicing comp48868\_c0 at nt 2279

alignment score=0.5 , category=0 , p=0.00384698163572694

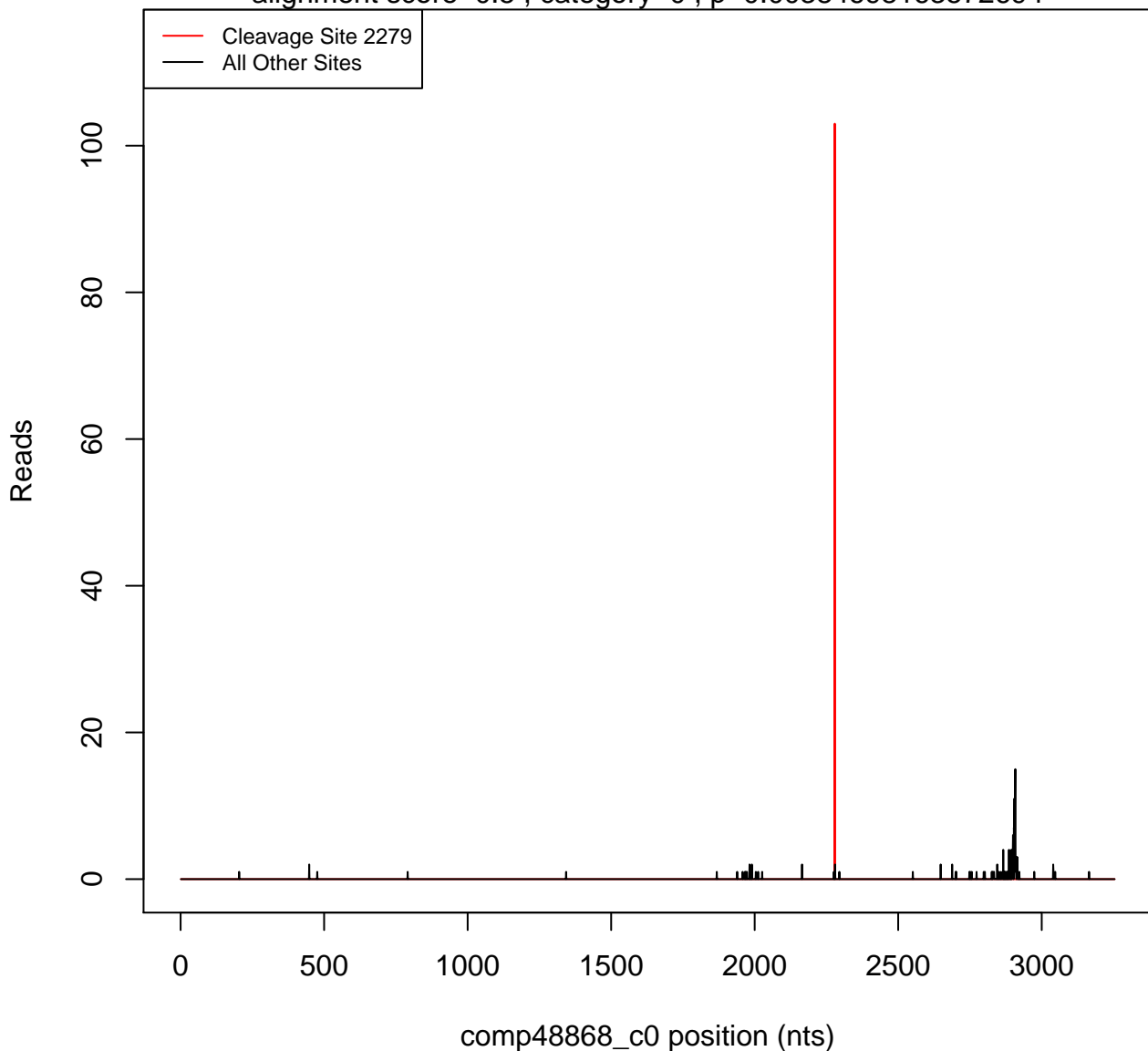

Supplement: S8 File — (ZIP) [file pone.0186500.s014.zip › S8 t-plot of miRNA-target/comp48868_c0--2279--mdm-miR160a_degradome.pdf]

# ath-miR171b\_1ss19AC slicing comp48892\_c1 at nt 2102

alignment score=3 , category=2 , p=0.0641588625669407

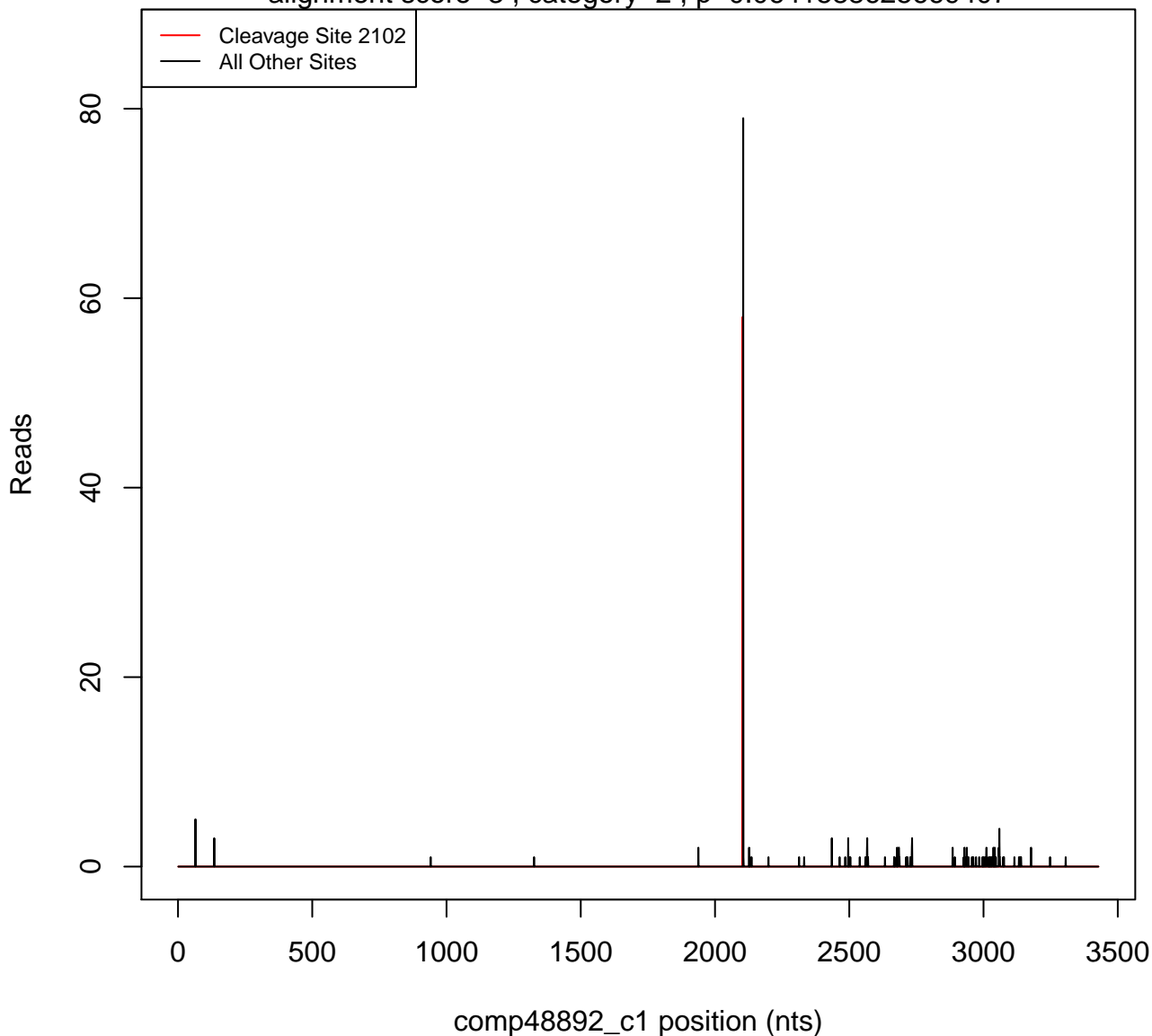

Supplement: S8 File — (ZIP) [file pone.0186500.s014.zip › S8 t-plot of miRNA-target/comp48892_c1--2102--ath-miR171b_1ss19AC_degradome.pdf]

# aqc-miR171a slicing comp48892\_c1 at nt 2105

alignment score=1 , category=0 , p=0.00357268870087279

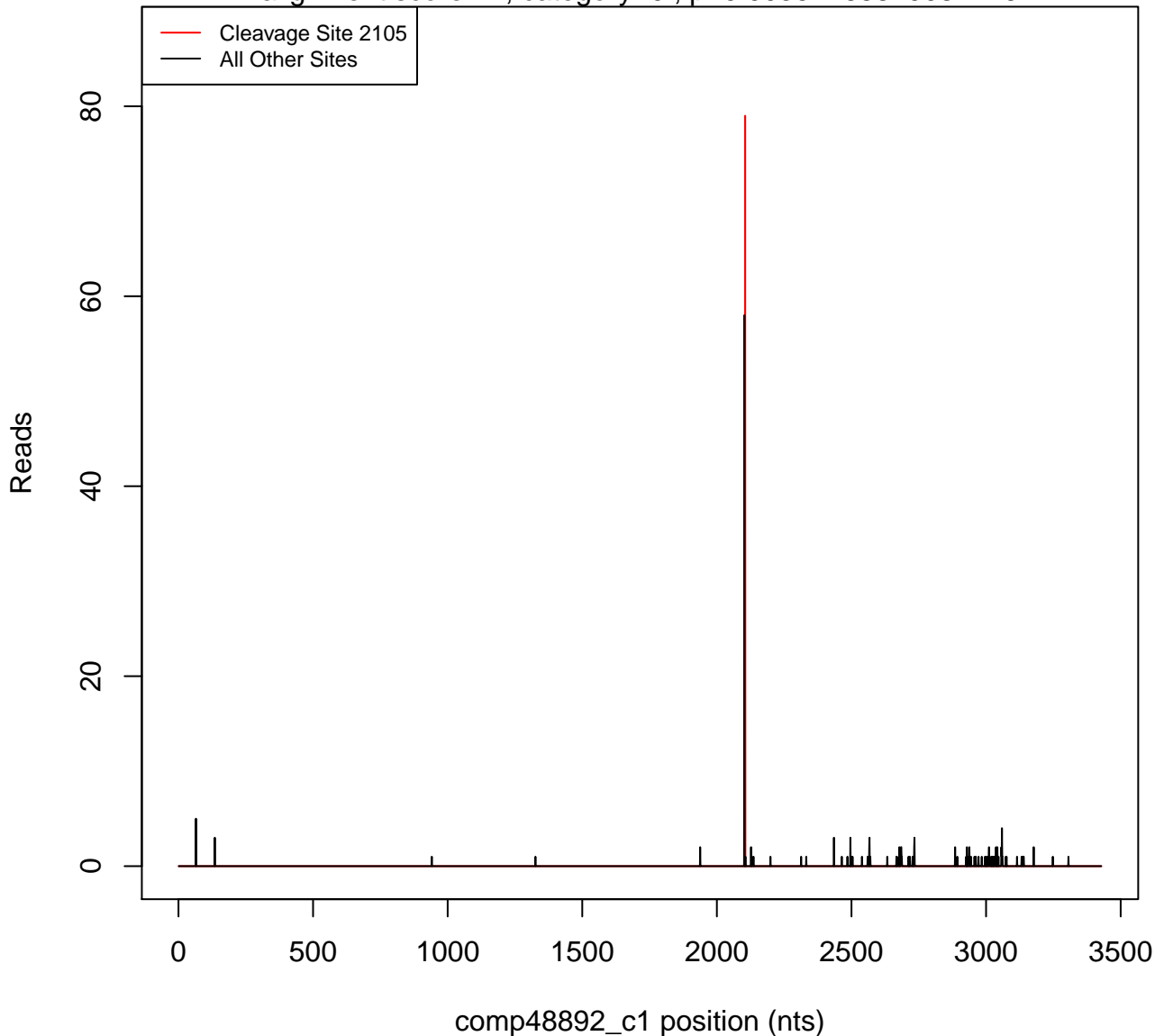

Supplement: S8 File — (ZIP) [file pone.0186500.s014.zip › S8 t-plot of miRNA-target/comp48892_c1--2105--aqc-miR171a_degradome.pdf]

# ath-miR171a\_2ss12CT21CT slicing comp48892\_c1 at nt 2105

alignment score=1.5 , category=0 , p=0.0032983202388529

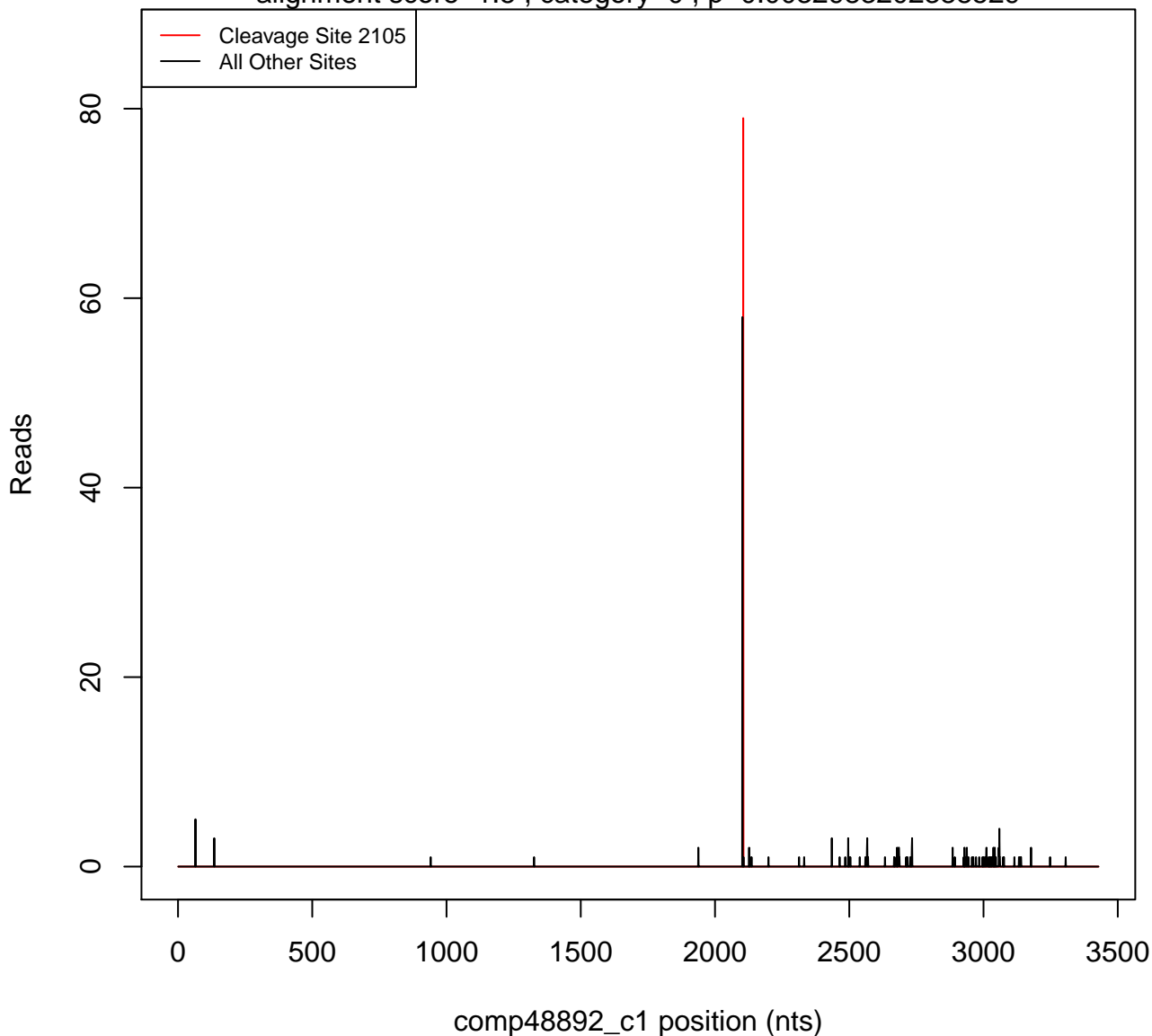

Supplement: S8 File — (ZIP) [file pone.0186500.s014.zip › S8 t-plot of miRNA-target/comp48892_c1--2105--ath-miR171a_2ss12CT21CT_degradome.pdf]

# ath-miR166a\_1ss20CT slicing comp51129\_c0 at nt 1532

alignment score=2.5 , category=0 , p=0.00274935665012399

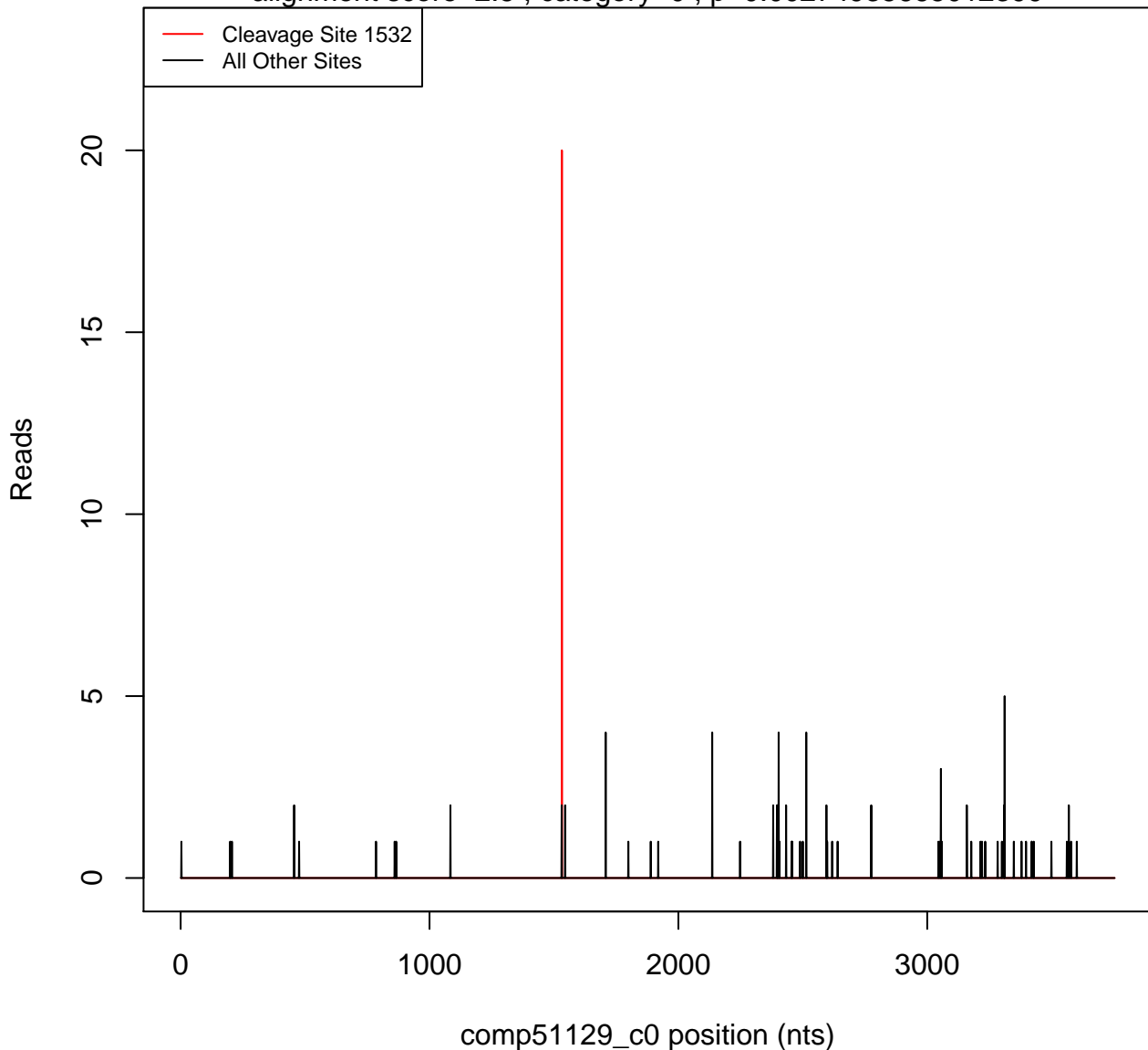

Supplement: S8 File — (ZIP) [file pone.0186500.s014.zip › S8 t-plot of miRNA-target/comp51129_c0--1532--ath-miR166a_1ss20CT_degradome.pdf]

# cme-MIR166i-p3\_1ss18CT slicing comp51129\_c0 at nt 1532

alignment score=1.5 , category=0 , p=0.0032983202388529

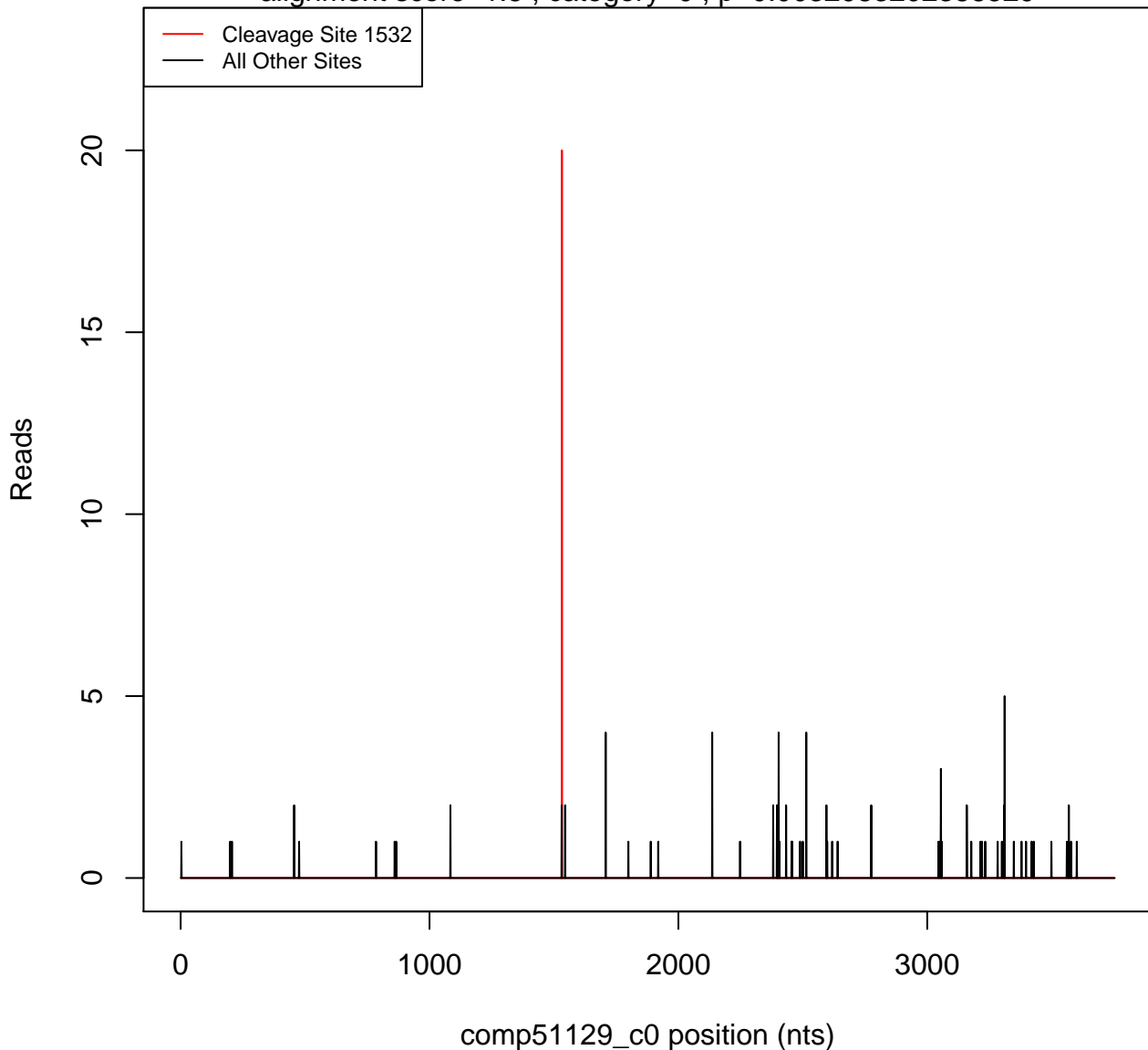

Supplement: S8 File — (ZIP) [file pone.0186500.s014.zip › S8 t-plot of miRNA-target/comp51129_c0--1532--cme-MIR166i-p3_1ss18CT_degradome.pdf]

# ath-miR162a slicing comp51493\_c0 at nt 2516

alignment score=2 , category=2 , p=0.0778478023024114

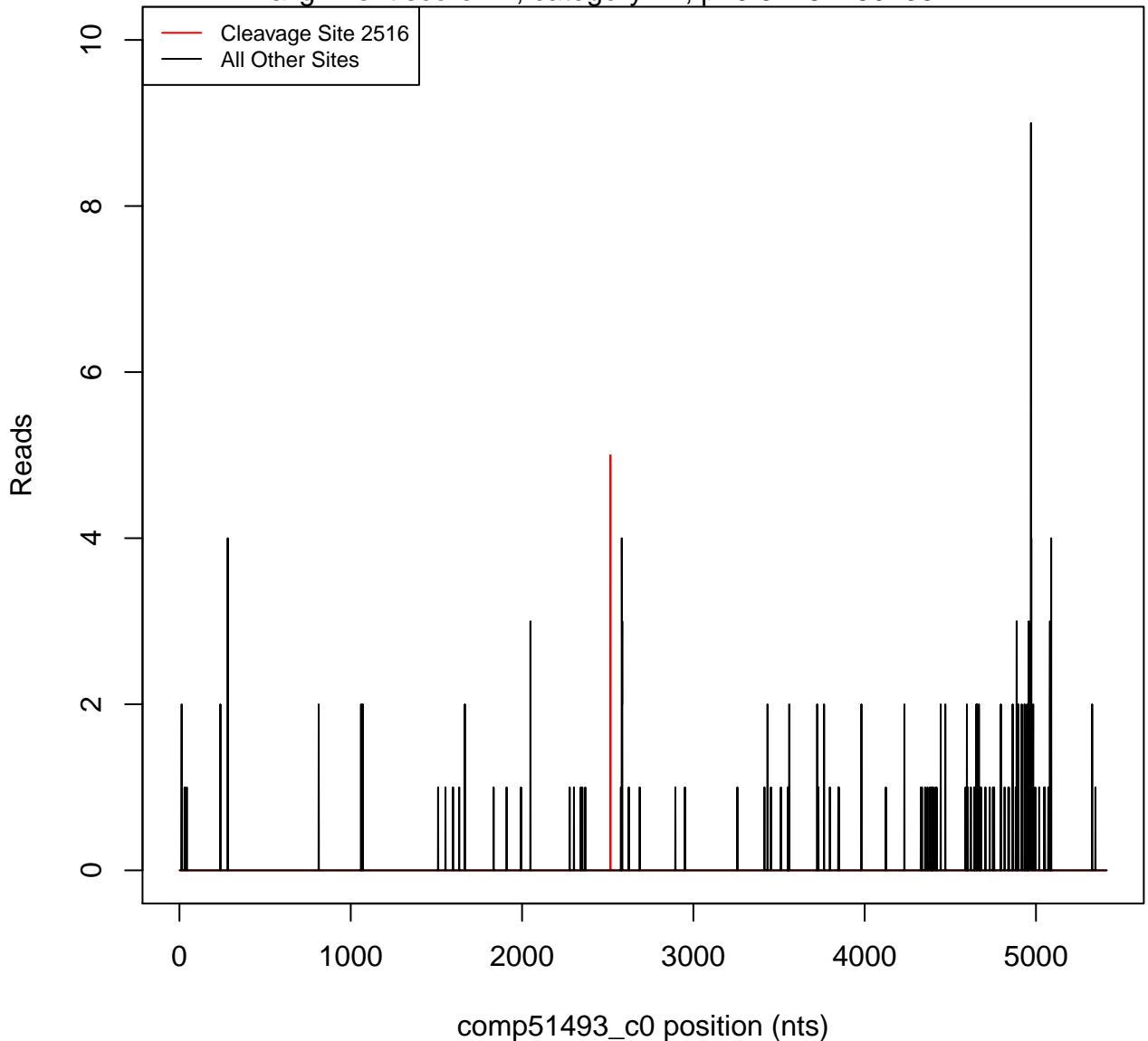

Supplement: S8 File — (ZIP) [file pone.0186500.s014.zip › S8 t-plot of miRNA-target/comp51493_c0--2516--ath-miR162a_degradome.pdf]

# PC-3p-207948\_11 slicing comp9730\_c0 at nt 49

alignment score=1.5 , category=0 , p=0.0032983202388529

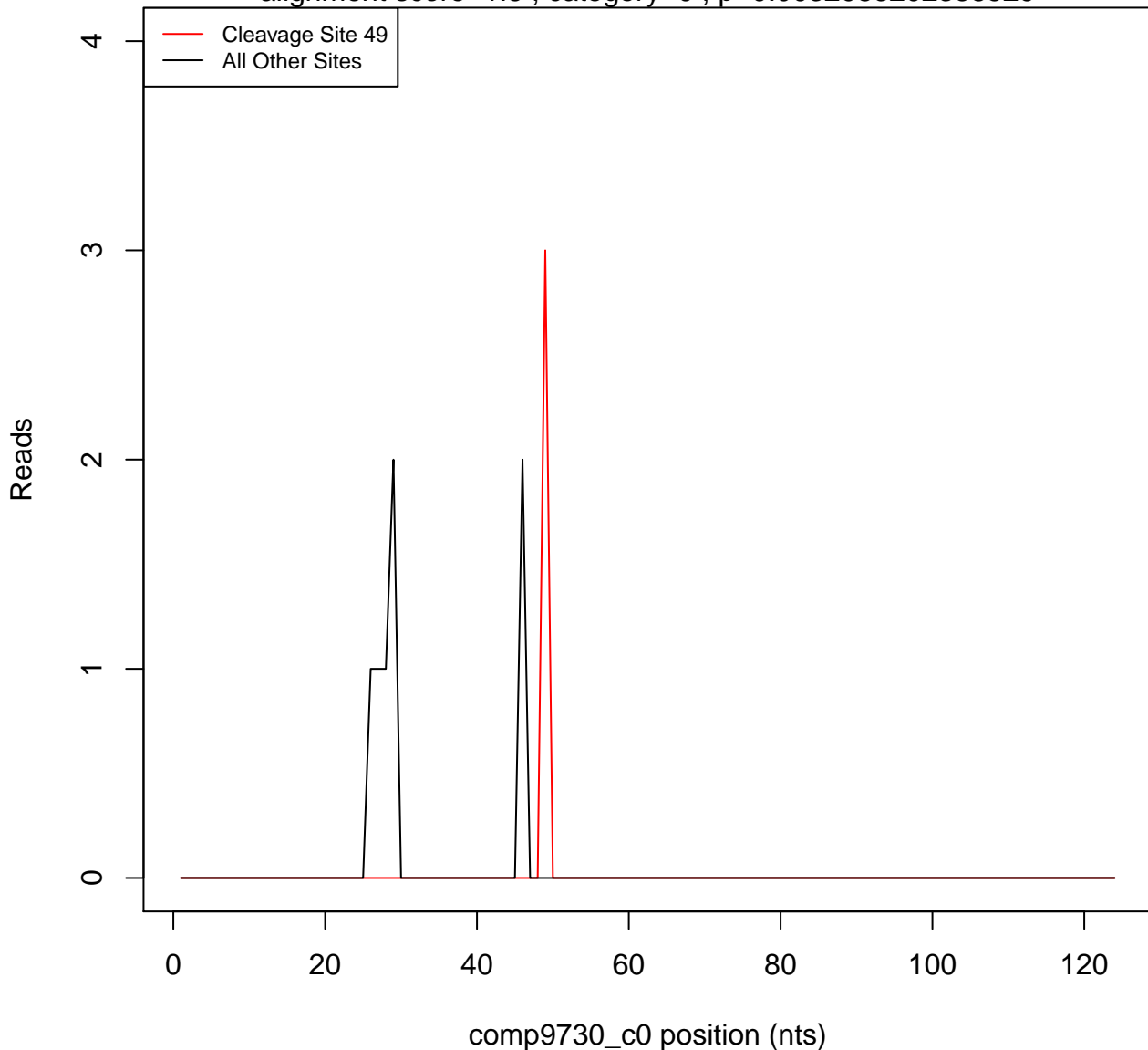

Supplement: S8 File — (ZIP) [file pone.0186500.s014.zip › S8 t-plot of miRNA-target/comp9730_c0--49--PC-3p-207948_11_degradome.pdf]

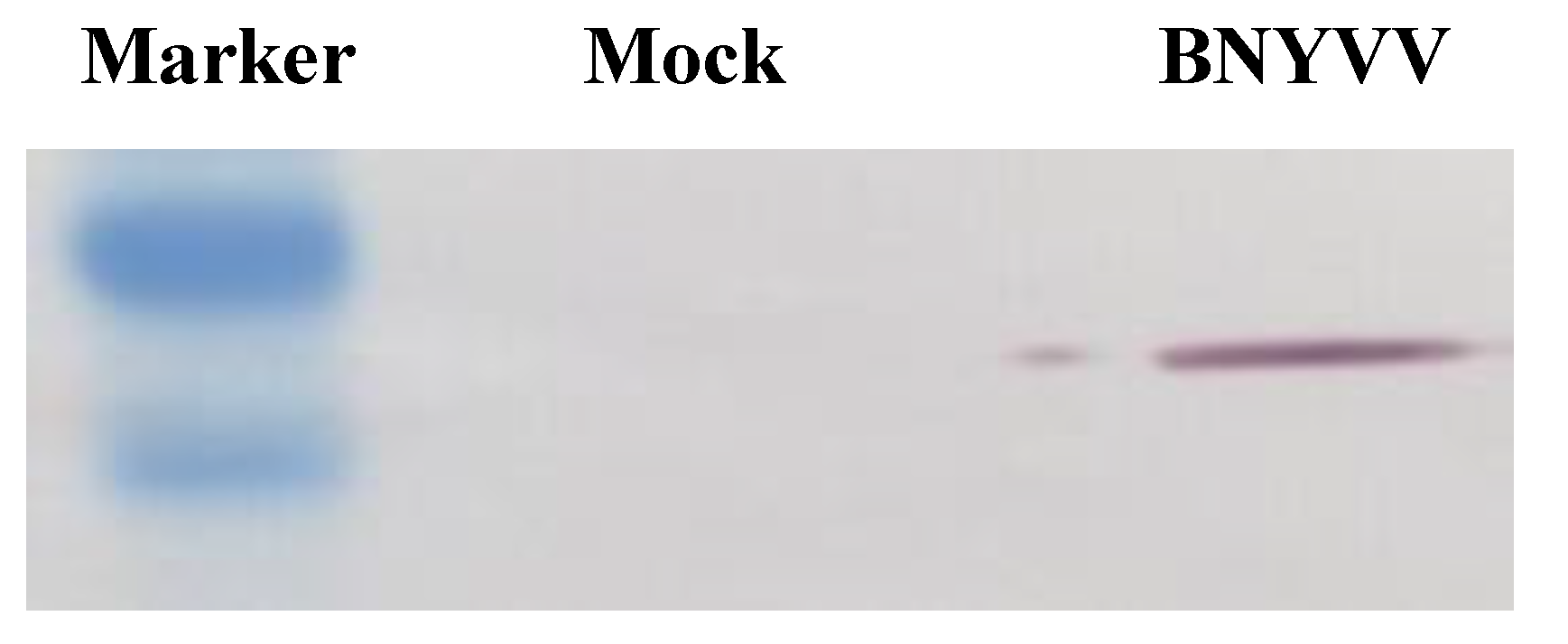

Supplement: S1 Fig — Mock indicates healthy B. macrocarpa inoculated with buffer only. (TIFF) [file pone.0186500.s015.tiff]
