# Supplementary material for: Characterization of microRNAs of Beta macrocarpa and their responses to Beet necrotic yellow vein virus infection
Source: PLoS One. 2017 Oct 16;12(10):e0186500. doi: 10.1371/journal.pone.0186500 (PMC5643120; doi:10.1371/journal.pone.0186500)
Supplement: S6 File — (PDF) [file pone.0186500.s012.pdf]

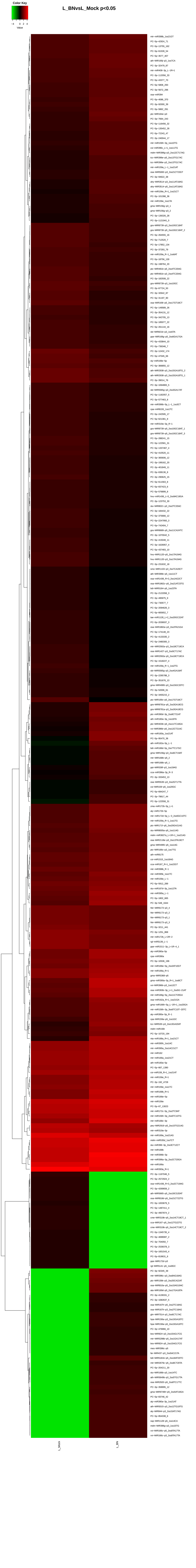
Color Key
-4 0 2 4
Value
L_BNvsL_Mock p<0.05
L_Mock
L_BN
